# Identification of Hub Genes for Colorectal Cancer with Liver Metastasis Using miRNA-mRNA Network

**DOI:** 10.1155/2023/2295788

**Published:** 2023-02-07

**Authors:** Si-ming Zhang, Cheng Shen, Jing Li, Xing-dan Wang, Si-qiu Zhai, Ling-ling Shi, Dan-li Lu, Xiao-hui Jiang, Junlan Qiu

**Affiliations:** ^1^Cancer Research Center Nantong, Nantong Tumor Hospital and Affiliated Tumor Hospital of Nantong University, Nantong, China; ^2^Department of Computer Science and Engineering, Tandon School of Engineering, New York University, Brooklyn, USA; ^3^Department of General Surgery, Nantong Tumor Hospital and Affiliated Tumor Hospital of Nantong University, Nantong, China; ^4^Department of Oncology and Hematology, The Affiliated Suzhou Science and Technology Town Hospital of Nanjing Medical University, Suzhou, Jiangsu Province, China

## Abstract

**Background:**

Liver metastasis is an important cause of death in patients with colorectal cancer (CRC). Increasing evidence indicates that microRNAs (miRNAs) are involved in the pathogenesis of colorectal cancer liver metastasis (CRLM). This study is aimed at exploring the potential miRNA-mRNA regulatory network.

**Methods:**

From the GEO database, we downloaded the microarray datasets GSE56350 and GSE73178. GEO2R was used to conduct differentially expressed miRNAs (DEMs) between CRC and CRLM using the GEO2R tool. Then, GO and KEGG pathway analysis for differentially expressed genes (DEGs) performed via DAVID. A protein-protein interaction (PPI) network was constructed by the STRING and identified by Cytoscape. Hub genes were identified by miRNA-mRNA network. Finally, the expression of the hub gene expression was assessed in the GSE81558.

**Results:**

The four DEMs (hsa-miR-204-5p, hsa-miR-122-5p, hsa-miR-95-3p, and hsa-miR-552-3p) were identified as common DEMs in GSE56350 and GSE73178 datasets. The SP1 was likely to adjust the upregulated DEMs; however, the YY1 could regulate both the upregulated and downregulated DEMs. A total of 3925 genes (3447 upregulated DEM genes and 478 downregulated DEM genes) were screened. These predicted genes were mainly linked to Platinum drug resistance, Cellular senescence, and ErbB signaling pathway. Through the gene network construction, most of the hub genes were found to be modulated by hsa-miR-204-5p, hsa-miR-122-5p, hsa-miR-95-3p, and hsa-miR-552-3p. Among the top 20 hub genes, the expression of CREB1, RHOA, and EGFR was significantly different in the GSE81558 dataset.

**Conclusion:**

In this study, miRNA-mRNA networks in CRLM were screened between CRC patients and CRLM patients to provide a new method to predict for the pathogenesis and development of CRC.

## 1. Introduction

Colorectal cancer (CRC) is a common malignant tumor [1, 2]. Metastasis is a major contributor to resulting in the mortality of patients with CRC, especially, liver metastasis, which has been shown as one of the leading causes of death in patients with CRC [3, 4]. Despite advances in hepatectomy and adjuvant therapy, the 5-year survival rate for colorectal cancer liver metastasis (CRLM) is still only 25-50% [5]. Hence, it is necessary to study the molecular mechanism regulating CRLM, providing evidence for the prevention to improve prognosis of patients.

MicroRNAs (miRNAs) are a class of noncoding RNAs composed of 20-24 nucleotides. They specifically bind to the 3′ untranslated regions of target genes through the principle of base complementary pairing, block the transcription of mRNA, and inhibit protein synthesis, thereby participating in the regulation of biological functions of target genes [6, 7]. Studies believed that miRNAs are closely related to tumor regulation, including CRLM [8–10]. miR-623 inhibits interleukin-8- (IL-8-) induced epithelial interstitial transformation of pancreatic cancer cells by inhibiting extracellular regulatory protein kinase (ERK) phosphorylation, demonstrating the important role of miR-623 in inhibiting *in vitro* migration and invasion of pancreatic cancer cells and *in vivo* metastasis [11].

In this study, we screened DEMs in CRLM compared to CRC without liver metastasis by analyzing two datasets (GSE5635 and GSE73178) from the GEO database. We verified the DEMs, identified the differential expression profile of miRNAs with a gradual increasing trend in transcription factor-DEM-target gene, and analyzed these target genes and hub gene network. The risk of colorectal cancer was analyzed using the demographic data and clinicopathological characteristics of patients with colorectal cancer and colorectal adenoma. Moreover, the expression of hub genes in combination with GSE81558 dataset was further confirmed and used to construct a relationship with miRNA-mRNA to improve the diagnosis and treatment of CRLM.

## 2. Methods

### 2.1. miRNA Microarray and DEG Identification

GEO [12] (http://www.ncbi.nlm.nih.gov/geo) is an international public functional dataset including high-throughput microarray and sequence-based data. The miRNA expression profiles of GSE56350 and GSE73178 of between CRC and CRLM were screened. The detailed dataset information is shown in Table 1. DEMs between CRC and CRLM across different GEO datasets. |log2FC| > 1 and a *P* value of <0.05 are considered significantly by the GEO2R tool. Then, the overlap of DEMs in the two datasets was identified by a Venn diagram (GSE56350 and GSE73178). All methods were carried out in accordance with relevant guidelines and regulations.

### 2.2. Predicting of Target Genes

miRNet (https://www.mirnet.ca/) analyzed the miRNA target interactions and displayed correlations in the network to predict downstream target genes of DEMs.

### 2.3. GO and KEGG Analysis

The Database for Annotation, Visualization and Integrated Discovery (DAVID) is an online bioinformatics tool that can provide GO and KEGG pathway enrichment analysis [13–15]. DAVID showed the unique biology of common DEGs and analyze the DEGs (*P* < 0.05).

### 2.4. Construction of Protein-Protein Interaction (PPI) and miRNA Hub Genes Network

The protein interaction network of target genes was constructed using STRING and Cytoscape tools in the version of 3.7.2. Hub genes were considered to be the top 30 genes using the Maximal Clique Centrality (MCC) method. The miRNA hub genes network was constructed by the Cytoscape software.

### 2.5. Evaluation of DEGs by GSE81558 Dataset

As there was no other data on mRNA expression between CRC and CRLM, we selected GSE81558 in the GEO database to analyze the DEGs. The dataset analyzed gene expression data, and we selected 23 cases of CRC and 19 cases of CRLM. Student's *t*-test was used to identify the DEGs between CRC and CRLM.

## 3. Results

### 3.1. Identification of DEMs in CRC and CRLM

**Figure 1:**
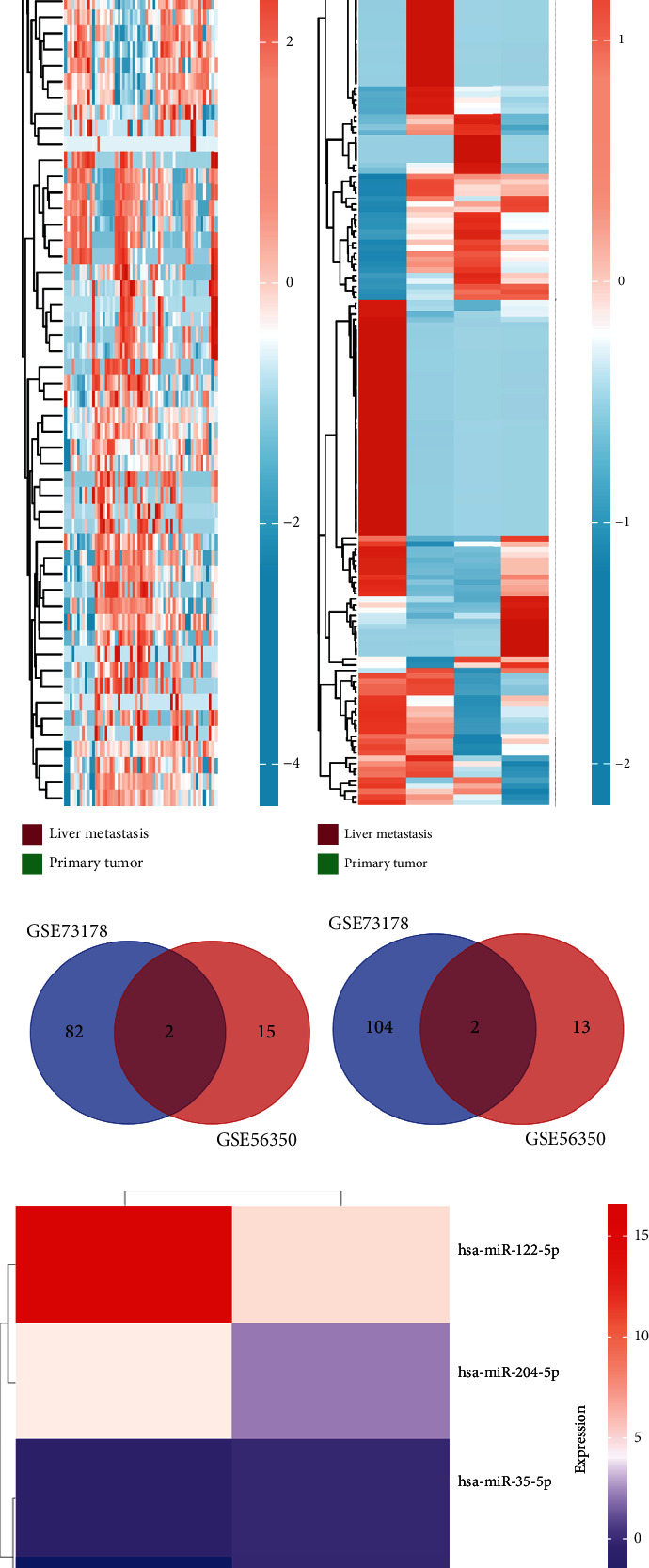
Identification of the potential DEMs. (a) DEMs in the GSE56350 dataset; (b) DEMs in the GSE73178 dataset; (c) Venn diagram for upregulated DEMs in the GSE56350 dataset and GSE73178 dataset; (d) for downregulated DEMs in the GSE56350 dataset and GSE73178 dataset; and (e) Heat map of the common DEMs in between the GSE56350 dataset and GSE73178 dataset.

As mentioned above, we found 32 DEMs (17 upregulated and 15 downregulated) that were identified in GSE56350 and 190 DEMs (84 upregulated and 106 downregulated) that were identified in the GSE73178 dataset (Figures 1(a) and 1(b)); the specific information is shown in Supplementary Tables S1–2. Between two DEMs, two upregulated (hsa-miR-204-5p and hsa-miR-122-5p) and two downregulated (hsa-miR-95-3p and hsa-miR-552-3p) DEMs were screened (Figures 1(c)–1(e)).

### 3.2. Target Prediction and Analysis of Downstream Genes of DEMs

**Figure 2:**
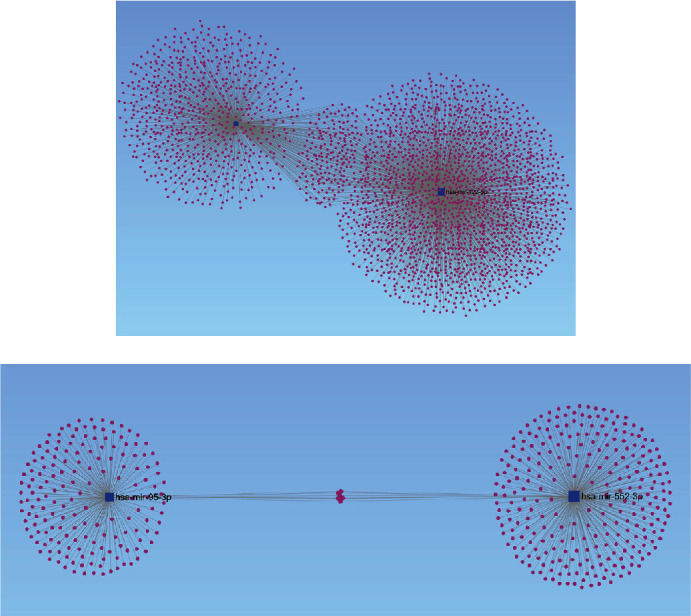
The predicted potential target genes by miRNet. (a) For upregulated DEMs and (b) for downregulated DEMs.

The miRNet database predicted a total of 3925 target genes as candidate DEMs, among which 3447 target genes are upregulated and 478 target genes are downregulated. For a better visualization, the DEMs and its target genes are depicted in Figures 2(a) and 2(b). We counted the genes shown in Table 2 and plotted the target genes presented (Supplementary Table S3).

### 3.3. GO and KEGG Analysis

**Figure 3:**
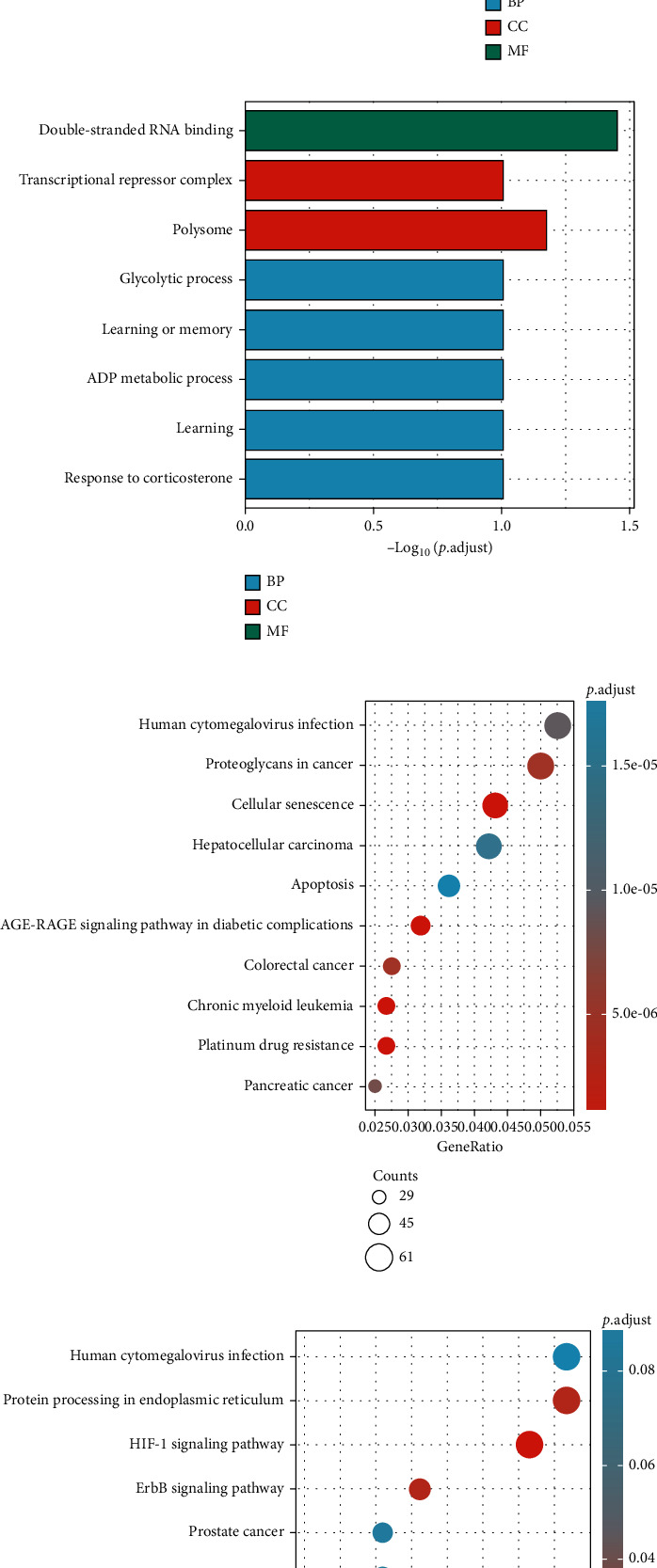
GO and KEGG pathway analysis. GO analysis of (a) upregulated DEMs and (b) downregulated genes; KEGG pathway analysis of (c) upregulated DEMs and (d) downregulated genes.

Then, the 3925 target genes were used for GO analysis and KEGG pathway enrichment analysis. For GO analysis, considering biological process (BP), upregulated genes were found to be enriched in positive regulation of chromosome organization, covalent chromatin modification, histone modification, and peptidyl-lysine modification. Upregulated DEM target genes' cellular component (CC) concentrated on focal adhesion, cell-substrate junction, and cell-substrate adherence junction. The molecular function (MF) analysis demonstrated that upregulated DEM target genes were significantly concentrated in cadherin binding, cell adhesion molecule binding, and DNA-binding transcription activator activity (Figure 3(a)). Moreover, downregulated genes were enriched in the double-stranded RNA binding, polysome, and glycolytic process (Figure 3(b)).

Furthermore, upregulated genes pathway analysis indicated that they are mainly enriched in platinum drug resistance, cellular senescence, AGE-RAGE signaling pathway in diabetic complications, chronic myeloid leukemia, colorectal cancer, and proteoglycans in cancer (Figure 3(c)). Moreover, downregulated genes were significantly enriched in HIF-1 signaling pathway, ErbB signaling pathway, and protein processing in endoplasmic reticulum (Figure 3(d)).

### 3.4. Identification of 20 Hub Genes

**Figure 4:**
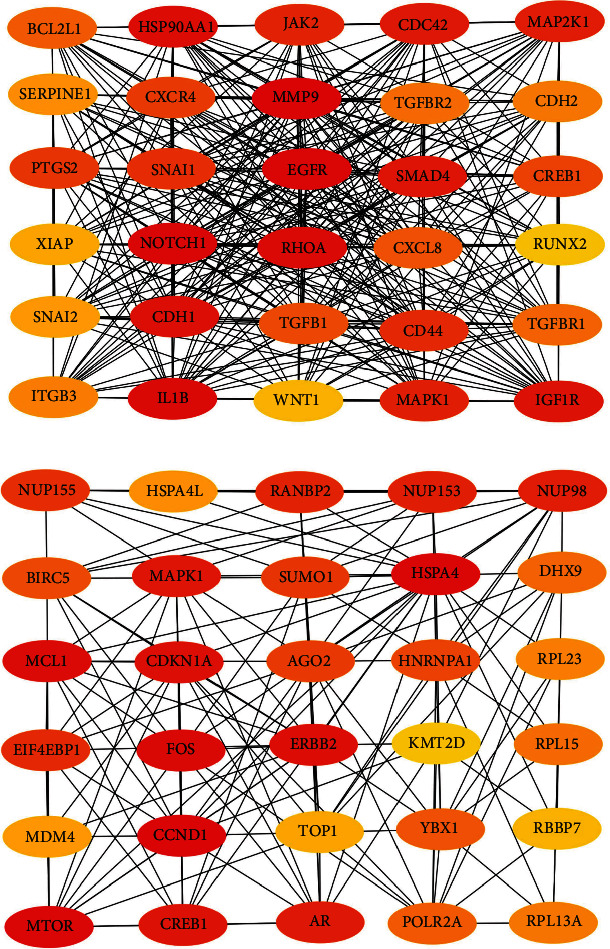
The top 30 hub genes in PPI network. (a) For upregulated DEM-hub genes and (b) for downregulated DEM-hub genes.

To confirm the relationship between DEGs and DEMs, we established a PPI network through the STRING database. At the same time, we uploaded the above-mentioned PPI network and imported in Cytoscape. The top 30 upregulated genes and 30 downregulated genes are shown in Figures 4(a) and 4(b).

**Figure 5:**
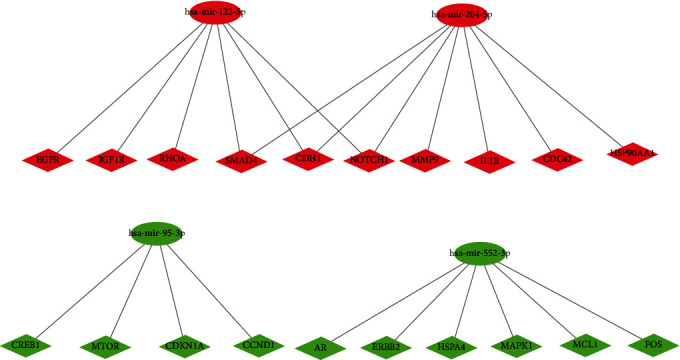
Identified potential miRNA-hub genes network.

Next, we constructed a regulatory network between upregulated DEMs and hub genes. hsa-mir-204-5p interacted with seven hub genes, including MMP9, NOTCH1, IL1B, HSP90AA1, CDH1, SMAD4, and CDC42, and hsa-mir-122-5p interacted with six hub genes, including EGFR, NOTCH1, RHOA, CDH1, IGF1R, and SMAD4. The correlation between DEMs of downregulated and hub genes were detected. Specifically, hsa-mir-552-3p was linked with six hub genes, including HSPA4, FOS, MCL1, ERBB2, MAPK1, and AR, and hsa-mir-95-3p was associated with CCND1, MTOR, CDKN1A, and CREB1 (Figure 5).

Based on the above results, the top 10 upregulated genes were MMP9, EGFR, NOTCH1, IL1B, RHOA, HSP90AA1, CDH1, IGF1R, SMAD4, and CDC42. The top 10 downregulated genes were CCND1, MTOR, HSPA4, FOS, MCL1, ERBB2, CDKN1A, CREB1, MAPK1, and AR (Table 3).

### 3.5. Identification of Hub Gene Expression

**Figure 6:**
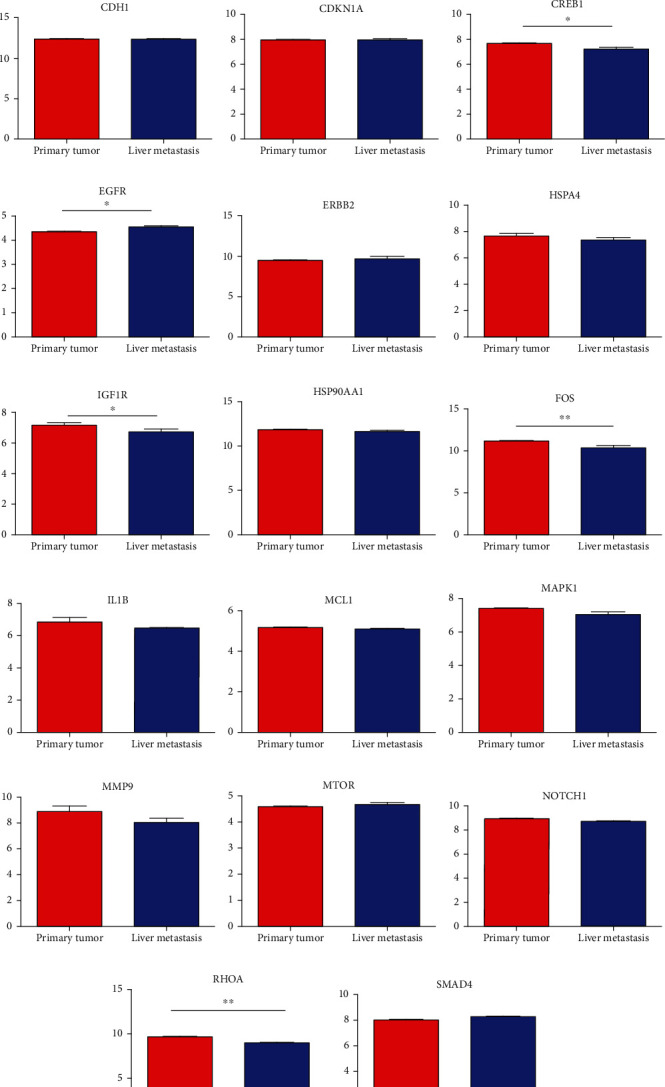
The expression of the top 20 target genes in primary tumor compared to liver metastasis of colorectal tumor. (a) AR, (b) CCND1, (c) CDC42, (d) CDH1, (e) CDKN1A, (f) CREB1, (g) EGFR, (h) ERBB2, (i) HSPA4, (j) IGF1R, (k) HSP90AA1, (l) FOS, (m) IL1B, (n) MCL1, (o) MAPK1, (p) MMP9, (q) MTOR, (r) NOTCH1, (s) RHOA, and (t) SMAD4.

Hub genes (MMP9, EGFR, NOTCH1, IL1B, RHOA, HSP90AA1, CDH1, IGF1R, SMAD4, CDC42, CCND1, MTOR, HSPA4,FOS, MCL1, ERBB2, CDKN1A, CREB1, MAPK1, and AR) were then from the GSE81558 dataset. For these hub genes, compared with CRC, the expressions of CREB1 and RHOA were decreased in CRLM; however, only the EGFR expression was increased in CRLM (Figures 6(a)–6(t)).

## 4. Discussion

In recent years, increasing studies in CRLM have been reported. However, the prognosis of the CRC patients is still poor because of the liver metastasis. Recently, with the development of the microarray technology, genetic alterations have been found during the progression of various diseases. In this study, the GSE56350 and GSE73178 datasets were used to identify DEMs between primary colorectal tumor and colorectal liver metastasis. Two upregulated DEMs (hsa-mir-204-5p and hsa-mir-122-5p) and two downregulated DEMs (hsa-mir-552-3p and hsa-mir-95-3p), which were significantly changed, were selected as candidate DEMs. Besides, from the upregulated DEMs, hsa-mir-204-5p could effectively restrain cancer cell proliferation [16]. miR-204-5p can inhibit cell proliferation, promote apoptosis, and enhance drug sensitivity by downregulating RAB22A expression in CRC [17]. The literature reports that miR-122-5p regulated CDC25A expression in CRC cells [18]. For downregulated DEMs, miR-552-3p was highly expressed in various types of tumor cells and can be used as a specific molecular marker, especially in CRC. A large number of bioinformatics studies also showed that miR-552-3p can be used as a biomarker for diagnosis and treatment of CRC [19]. Using RNA sequencing analysis (RNA-seq), miR-95-3p has been found to be linked to cisplatin resistance in gastric cancer via increasing the PI3K/Akt pathway [20, 21]. Notably, to better understand the mechanisms of these miRNAs involved, further studies for their roles in CRLM were needed.

miRNA expression is abnormal in almost all malignant tumors, which acts as oncogenes or tumor suppressor genes and is regulated by transcription factors [22–24]. SP1 has been detected in CRC [25–27]. ADEM10, EPHB2, HDAC4, and SEPP1 in CRC inhibit cell migration, invasion, tumor growth, and liver metastasis through the SP1 [27]. YY1 regulated the expressions of both upregulated and downregulated DEMs. In the study, YY1 as a member of the PcG protein family can be widely expressed in a variety of tissues and cells and is involved in cell tissue differentiation, chromatin remodeling, and tumor genesis and progression [28–31]. YY1 is closely associated with tumor including metastatic breast cancer [32, 33], colon cancer [34], gastric cancer [35], and prostate cancer [36]. In CRC, through the NF-*κ*B/YY1 axis, LINC01578 enhanced its promoter activity.

Triptolide regulates E2F activity by potentially inducing G1 cell cycle [37]. Apart from SP4, EGR1, ARID3A, and NKX6-1, the remaining transcription factors have been reported in colorectal cancer [38–42], which supports the importance of these candidate DEMs in the mechanism of CRC tumor.

GO and KEGG pathway enrichment analysis were conducted via DAVID. GO analysis revealed that these DEGs were particularly enriched in the positive regulation of chromosome organization, covalent chromatin modification, histone modification, and peptidyl-lysine modification and regulation of chromosome organization. For the molecular function, these genes were also significantly enriched in cadherin binding, cell adhesion molecule binding, DNA-binding transcription activator activity, RNA polymerase II-specific, protein serine/threonine kinase activity, and enhancer binding.

KEGG analysis showed that the hub genes mainly focused on the platinum drug resistance, cellular senescence, AGE-RAGE signaling pathway in diabetic complications, HIF-1 signaling pathway, ErbB signaling pathway, and protein processing in endoplasmic reticulum.

In previous studies, drug resistance has been documented to be associated with liver metastasis of CRC. A study showed that a lack of E-cadherin promotes CRC cell growth, invasion, and drug resistance, contributing to CRC progression and metastatic dissemination [43]. Cellular senescence is also an important factor in tumor metastasis. Cancer stem cells could change their phenotypic and functional appearance. These changes are caused by chemotherapy and radiation, leading to changes in the tumor microenvironment [44]. Research reported that HIF-1*α* (hypoxia inducible factor-1*α*) expression in liver metastasis determines poor prognosis of CRC liver metastasis patients [45]. Therefore, drug resistance, cellular senescence, and HIF-1 signaling pathway may represent and be developed as a novel therapeutic strategy for treating patients with CRC liver metastasis. However, many of the target genes were downregulated without difference in GO analysis, suggesting that upregulated DEMs may play a more critical role in the liver metastasis of CRC.

To screen the DEM-hub genes of CRLM, we found that these genes could be potentially targeted by hsa-miR-204-5p, hsa-miR-122-5p, hsa-miR-95-3p, and hsa-miR-552-3p. Among the top 20 hub genes, the expression of three genes (CREB1, RHOA, and EGFR) was significantly different in the GSE81558 dataset. CREB1 binds to the conserved cAMP response element (CRE) on the promoter to promote gene transcription and activate phosphorylation [46, 47]. The literature reports that CREB1 could suppress CRC proliferation, invasion, and metastasis [48]. Li et al. showed that knockdown of CREB1 exerts effects on proliferation, migration, and invasion of CRC cells [49]. RHOA, a member of the ras homolog gene family, might affect the domain of E-cadherin and endocytosis [50]. Epidermal growth factor receptor (EGFR) was associated with the FOXK2 and mediated CRC metastasis. Moreover, CRC metastasis was inhibited significantly via the EGFR monoclonal antibody cetuximab [51].

In the present study, we investigated the potential miRNA-mRNA regulatory network in CRLM. But there are still limitations. Firstly, we targeted to miRNA-mRNA network between CRC and CRLM; however, some of these underlying mechanisms of CRLM should be further confirmed. Secondly, compared with the number of the sample usually required for biomarker analysis, the sample size of the current article was small. Thirdly, the miRNA-mRNA network was only associated with public databases, and experiments in vivo and in vitro were required to validate our analysis.

## 5. Conclusion

In summary, through the GEO database and bioinformatics analysis, we identified 4 DEMs and 20 hub genes using PPI analysis, potential miRNA-mRNA network in CRLM, hoping that these findings will contribute to improving the prognosis of patients with CRLM.

## Figures and Tables

**Table 1:** Details for GEO COPD data.

Accession	Sample	Primary colorectal tumor	Colorectal liver metastasis	Gene/microRNA
GSE536350	Tumor	46	15	microRNA
GSE73178	Tumor	2	2	microRNA
GSE81558	Tumor	23	19	Gene

**Table 2:** Potential genes of the upregulated and downregulated DEMs.

Upregulated miRNA	Number	Downregulated miRNA	Number
hsa-mir-204-5p	985	hsa-mir-552-3p	291
hsa-mir-122-5p	2462	hsa-mir-95-3p	187
Total	3447	Total	478

**Table 3:** The top 10 hub genes of the significantly upregulated and downregulated DEMs by MCC.

Upregulated		Downregulated	
Gene	Score	Gene	Score
MMP9	3.29E + 09	CCND1	6732
EGFR	3.28E + 09	MTOR	6489
NOTCH1	3.17E + 09	HSPA4	6422
IL1B	3.04E + 09	FOS	6047
RHOA	2.89E + 09	MCL1	3675
HSP90AA1	2.70E + 09	ERBB2	3510
CDH1	2.64E + 09	CDKN1A	3420
IGF1R	2.47E + 09	CREB1	3335
SMAD4	1.97E + 09	MAPK1	3288
CDC42	1.93E + 09	AR	3029

## Data Availability

All data were obtained from the public database described in Materials and Methods and carried out in accordance with relevant guidelines and regulations.

## Abbreviations

CRC: Colorectal cancer
MiRNAs: MicroRNAs
CRLM: Colorectal cancer liver metastasis
DEMs: Differentially expressed miRNAs
DEGs: Differentially expressed genes
DAVID: Visualization and Integrated Discovery.

## Supplementary Materials

Supplementary Materials Table S1. DEMs between primary colorectal tumor and colorectal liver metastasis from the GSE56350 dataset. Table S2. DEMs between primary colorectal tumor and colorectal liver metastasis from the GSE73178 dataset. Table S3. Target genes of DEMs predicted by miRNet.
