# Supplementary material for: Identification of Hub Genes for Colorectal Cancer with Liver Metastasis Using miRNA-mRNA Network
Source: Dis Markers. 2023 Feb 7;2023:2295788. doi: 10.1155/2023/2295788 (PMC9928517; doi:10.1155/2023/2295788)
Supplement: Supplementary Materials — Table S1. DEMs between primary colorectal tumor and colorectal liver metastasis from the GSE56350 dataset. Table S2. DEMs between primary colorectal tumor and colorectal liver metastasis from the GSE73178 dataset. Table S3. Target genes of DEMs predicted by miRNet. [file 2295788.f1.zip › Language certificate.pdf]

Bullet Edits

EDITORIAL CERTIFICATE

This document certifies that the paper listed below was edited and proofread for proper English language, grammar, punctuation, spelling, and overall style by one or more than one highly qualified native speakers at Bullet Edits. All of the suggested amendments were tracked with the Microsoft Word "Track Changes" feature. Therefore, the author had the option to reject or accept each change individually.

Manuscript Title:

Identification of hub genes for colorectal cancer with liver metastasis using miRNA-mRNA network

Anti-counterfeiting Code:

125b551c3f10529153e0b9f59bb58840

Date Issued:

2022-10-16

Bullet Edits is a registered company headquartered in the UK with a global presence. We offer a range of editing, proofreading services to authors. Our Ph.D. editors are all native English speakers from the USA and UK. Authors who work with Bullet Edits are guaranteed excellent language quality and timely delivery.

BULLET EDITS LIMITED COMPANY NO. 13345261 SEAL ENGLAND AND WALES

Address: BULLET EDITS LIMITED, 85 Great Portland Street, London,UK
Tel: UK. (+44)20457 70286 / US.(+1)312-313-9179
Web: www.bulletedits.cn/Email: info@bulletedits.cn
VAT: GB 378 9316 43
