# Supplementary material for: Identification of Hub Genes for Colorectal Cancer with Liver Metastasis Using miRNA-mRNA Network
Source: Dis Markers. 2023 Feb 7;2023:2295788. doi: 10.1155/2023/2295788 (PMC9928517; doi:10.1155/2023/2295788)
Supplement: Supplementary Materials — Table S1. DEMs between primary colorectal tumor and colorectal liver metastasis from the GSE56350 dataset. Table S2. DEMs between primary colorectal tumor and colorectal liver metastasis from the GSE73178 dataset. Table S3. Target genes of DEMs predicted by miRNet. [file 2295788.f1.zip › Supplementary tables 1-3.docx]

**Table S1** DEMs between primary colorectal tumor and colorectal liver metastasis from GSE56350 dataset.

| ID | adj.*P*.Value | *P*.Value | t | B | logFC |
| --- | --- | --- | --- | --- | --- |
| hsa-miR-122 | 1.98E-18 | 2.52E-21 | 14.578118 | 36.9233 | 5.0060249 |
| hsa-miR-609 | 5.81E-06 | 1.48E-08 | 6.551517 | 9.4564 | 2.8965874 |
| hsa-miR-151-3p | 5.81E-06 | 2.22E-08 | 6.44717 | 9.0703 | 2.2925548 |
| hsa-miR-409-3p | 1.94E-02 | 9.89E-05 | 4.173427 | 1.1199 | 1.9865587 |
| hsa-miR-125a-5p | 3.64E-01 | 2.09E-02 | -2.372476 | -3.7594 | -1.3631425 |
| hsa-miR-133a | 2.84E-01 | 1.30E-02 | -2.559776 | -3.3454 | -1.5943374 |
| hsa-miR-424 | 3.94E-02 | 3.89E-04 | 3.760183 | -0.1553 | 0.5927613 |
| hsa-miR-133b | 4.83E-01 | 3.33E-02 | -2.179064 | -4.1595 | -1.3293521 |
| hsa-miR-143 | 3.94E-02 | 4.38E-04 | -3.723518 | -0.2647 | -2.1913772 |
| hsa-miR-425* | 8.80E-02 | 1.12E-03 | 3.423636 | -1.1337 | 1.0617063 |
| HUMTRF:CONTROL | 1.34E-01 | 1.88E-03 | 3.253486 | -1.6054 | 0.7700645 |
| hsa-miR-520e | 1.66E-01 | 2.53E-03 | -3.152452 | -1.8776 | -0.8861943 |
| hsa-mir-604 | 1.69E-01 | 2.80E-03 | -3.118106 | -1.9688 | -0.7137881 |
| hsa-miR-145 | 2.40E-01 | 8.58E-03 | -2.718107 | -2.976 | -1.0822196 |
| hsa-miR-640 | 1.78E-01 | 3.41E-03 | 3.049805 | -2.1479 | 1.5390822 |
| hsa-mir-196b | 1.82E-01 | 3.72E-03 | -3.019428 | -2.2267 | -0.5793299 |
| hsa-miR-185 | 1.83E-01 | 3.97E-03 | -2.997126 | -2.2842 | -0.9060839 |
| hsa-miR-147b | 2.39E-01 | 7.35E-03 | -2.775646 | -2.8376 | -1.3424703 |
| hsa-miR-105* | 2.11E-01 | 5.11E-03 | 2.907632 | -2.5116 | 0.7515068 |
| hsa-mir-204 | 2.14E-01 | 5.62E-03 | 2.873323 | -2.5974 | 1.57763 |
| hsa-mir-563 | 2.14E-01 | 5.73E-03 | -2.866557 | -2.6142 | -0.8113014 |
| hsa-miR-371-3p | 2.25E-01 | 6.33E-03 | -2.830343 | -2.7039 | -0.563145 |
| hsa-mir-431 | 2.39E-01 | 7.33E-03 | -2.776377 | -2.8358 | -0.9216708 |
| hsa-miR-198 | 3.94E-02 | 4.53E-04 | -3.713124 | -0.2956 | -1.2482531 |
| hsa-miR-570 | 2.39E-01 | 7.63E-03 | 2.761564 | -2.8717 | 0.7279436 |
| hsa-miR-122* | 2.40E-01 | 7.94E-03 | 2.746907 | -2.907 | 0.4629804 |
| hsa-miR-199b-5p | 3.06E-01 | 1.44E-02 | -2.519632 | -3.4363 | -1.0162007 |
| hsa-miR-337-3p | 1.99E-01 | 4.57E-03 | -2.947367 | -2.4112 | -1.1208196 |
| hsa-miR-181c* | 2.40E-01 | 9.11E-03 | -2.695864 | -3.029 | -0.890542 |
| hsa-miR-378 | 2.40E-01 | 9.18E-03 | -2.69278 | -3.0363 | -1.1166759 |
| hsa-miR-93* | 2.52E-01 | 1.01E-02 | -2.656767 | -3.1212 | -0.3499998 |
| hsa-miR-548b-3p | 1.78E-01 | 3.28E-03 | -3.063325 | -2.1127 | -1.197373 |
| hsa-miR-92a-1* | 2.58E-01 | 1.09E-02 | -2.629447 | -3.185 | -0.7190372 |
| hsa-miR-29a | 2.63E-01 | 1.14E-02 | 2.610646 | -3.2286 | 1.6694598 |
| hsa-miR-146b-5p | 2.84E-01 | 1.30E-02 | 2.561833 | -3.3407 | 1.7327004 |
| hsa-mir-552 | 3.94E-02 | 3.12E-04 | -3.828083 | 0.0489 | -1.3609605 |
| hsa-mir-571 | 2.40E-01 | 8.90E-03 | -2.70466 | -3.0081 | -1.2361658 |
| hsa-miR-550* | 3.32E-01 | 1.63E-02 | 2.47313 | -3.5401 | 0.1227264 |
| hsa-miR-409-3p | 3.32E-01 | 1.65E-02 | 2.466832 | -3.554 | 0.2710805 |
| hsa-miR-192 | 3.42E-01 | 1.78E-02 | 2.436952 | -3.6197 | 1.7030978 |
| hsa-miR-602 | 3.42E-01 | 1.80E-02 | 2.433725 | -3.6268 | 0.738311 |
| hsa-miR-768-3p | 3.42E-01 | 1.86E-02 | 2.420017 | -3.6567 | 1.6476607 |
| hsa-miR-191* | 3.42E-01 | 1.87E-02 | -2.416515 | -3.6643 | -0.5257243 |
| hsa-miR-214 | 3.52E-01 | 1.98E-02 | 2.395023 | -3.7109 | 0.5280451 |
| hsa-miR-576-5p | 2.52E-01 | 1.03E-02 | -2.650739 | -3.1353 | -1.0578227 |
| hsa-miR-557 | 3.79E-01 | 2.22E-02 | -2.347952 | -3.8117 | -0.76024 |
| hsa-miR-769-3p | 3.88E-01 | 2.33E-02 | 2.329081 | -3.8516 | 0.5482349 |
| HSTRNL:CONTROL | 4.02E-01 | 2.46E-02 | 2.305441 | -3.9012 | 0.8204021 |
| hsa-miR-768-5p | 4.32E-01 | 2.70E-02 | 2.267627 | -3.9798 | 1.2034446 |
| hsa-miR-422a | 4.38E-01 | 2.80E-02 | 2.25294 | -4.01 | 0.342071 |
| hsa-miR-638 | 4.46E-01 | 2.90E-02 | 2.236882 | -4.0428 | 1.0653145 |
| hsa-miR-34b | 4.72E-01 | 3.15E-02 | -2.20238 | -4.1127 | -0.7167167 |
| hsa-miR-487b | 4.72E-01 | 3.19E-02 | -2.197514 | -4.1225 | -0.5830285 |
| hsa-miR-95 | 3.94E-02 | 3.35E-04 | -3.805951 | -0.0179 | -1.3341066 |
| hsa-miR-30b | 4.98E-01 | 3.61E-02 | 2.144468 | -4.228 | 1.4869424 |
| hsa-miR-192* | 4.98E-01 | 3.66E-02 | -2.138313 | -4.2401 | -0.4785829 |
| hsa-mir-206 | 4.98E-01 | 3.70E-02 | 2.133253 | -4.25 | 0.3389845 |
| hsa-let-7a | 4.98E-01 | 3.89E-02 | 2.112347 | -4.2908 | 1.2346643 |
| hsa-miR-149 | 4.98E-01 | 3.99E-02 | -2.100317 | -4.3141 | -0.6855682 |
| hsa-mir-551a | 4.98E-01 | 4.08E-02 | 2.091249 | -4.3316 | 0.6788779 |
| hsa-miR-556-3p | 4.98E-01 | 4.41E-02 | -2.056406 | -4.3982 | -0.528161 |
| hsa-miR-521 | 4.98E-01 | 4.60E-02 | 2.037784 | -4.4334 | 0.143658 |
| hsa-miR-672 | 4.98E-01 | 4.67E-02 | -2.031088 | -4.446 | -0.3378746 |
| hsa-miR-181a-2* | 4.98E-01 | 4.70E-02 | -2.028093 | -4.4517 | -0.6929463 |
| hsa-mir-636 | 4.98E-01 | 4.74E-02 | 2.024352 | -4.4587 | 0.675532 |
| hsa-miR-500* | 4.98E-01 | 4.75E-02 | 2.02347 | -4.4603 | 1.124529 |
| hsa-miR-518a-3p | 4.98E-01 | 4.82E-02 | -2.017108 | -4.4722 | -0.5260876 |
| hsa-miR-126* | 4.98E-01 | 4.84E-02 | 2.015607 | -4.475 | 1.6315477 |
| hsa-mir-326 | 4.98E-01 | 5.15E-02 | 1.987613 | -4.5269 | 0.3421093 |
| hsa-miR-429 | 4.98E-01 | 5.15E-02 | 1.986955 | -4.5281 | 0.469486 |
| hsa-miR-625* | 4.98E-01 | 5.25E-02 | -1.978648 | -4.5434 | -0.4185916 |
| hsa-miR-496 | 4.98E-01 | 5.33E-02 | 1.971922 | -4.5557 | 1.5036158 |
| hsa-miR-708* | 4.98E-01 | 5.52E-02 | -1.956022 | -4.5847 | -0.1735862 |
| hsa-miR-135b | 4.98E-01 | 5.64E-02 | -1.946063 | -4.6028 | -0.7590229 |
| hsa-miR-562 | 4.98E-01 | 5.72E-02 | -1.939109 | -4.6154 | -0.7778452 |
| hsa-miR-138 | 4.98E-01 | 5.79E-02 | -1.933372 | -4.6257 | -0.2616602 |
| hsa-miR-193a-5p | 4.98E-01 | 5.80E-02 | 1.933064 | -4.6262 | 0.7687569 |
| hsa-miR-301a | 4.98E-01 | 5.91E-02 | -1.923948 | -4.6426 | -0.1198068 |
| hsa-miR-103 | 4.98E-01 | 5.94E-02 | 1.921625 | -4.6468 | 1.6439439 |
| hsa-miR-128a | 4.98E-01 | 6.07E-02 | -1.912169 | -4.6636 | -0.3956371 |
| hsa-miR-382 | 4.98E-01 | 6.15E-02 | -1.905892 | -4.6748 | -0.6425767 |
| hsa-miR-215 | 4.98E-01 | 6.17E-02 | 1.904655 | -4.677 | 1.8636379 |
| hsa-miR-342-3p | 4.98E-01 | 6.18E-02 | -1.903367 | -4.6793 | -0.3510092 |
| hsa-mir-196a-1 | 4.98E-01 | 6.34E-02 | -1.891957 | -4.6995 | -0.7147735 |
| hsa-miR-144 | 4.98E-01 | 6.51E-02 | 1.879326 | -4.7217 | 0.1430409 |
| hsa-miR-26a | 4.98E-01 | 6.55E-02 | 1.876598 | -4.7265 | 1.6415879 |
| hsa-mir-573 | 4.98E-01 | 6.65E-02 | -1.869481 | -4.7389 | -0.130831 |
| hsa-miR-107 | 4.98E-01 | 6.78E-02 | 1.859961 | -4.7555 | 1.5922544 |
| hsa-mir-581 | 4.98E-01 | 6.92E-02 | -1.850268 | -4.7723 | -0.7721955 |
| hsa-miR-34b* | 4.98E-01 | 6.94E-02 | -1.849212 | -4.7742 | -0.4835283 |
| hsa-miR-148a* | 4.98E-01 | 7.02E-02 | -1.843965 | -4.7832 | -0.2313071 |
| hsa-miR-373* | 4.98E-01 | 7.02E-02 | -1.843735 | -4.7836 | -0.2597781 |
| hsa-miR-18a | 4.98E-01 | 7.03E-02 | -1.842706 | -4.7854 | -0.1199276 |
| hsa-miR-9 | 4.98E-01 | 7.11E-02 | 1.837735 | -4.794 | 0.8768985 |
| hsa-mir-562 | 4.98E-01 | 7.11E-02 | -1.837435 | -4.7945 | -0.9267923 |
| hsa-mir-647 | 4.98E-01 | 7.18E-02 | -1.833054 | -4.802 | -0.1170337 |
| hsa-miR-645 | 4.98E-01 | 7.19E-02 | -1.8322 | -4.8035 | -0.5504813 |
| hsa-miR-379 | 4.98E-01 | 7.26E-02 | 1.827426 | -4.8116 | 0.2157387 |
| hsa-let-7e | 4.98E-01 | 7.30E-02 | 1.825341 | -4.8152 | 0.8346485 |
| hsa-mir-557 | 4.98E-01 | 7.30E-02 | 1.824975 | -4.8158 | 0.209558 |
| hsa-miR-20a | 4.98E-01 | 7.31E-02 | 1.824597 | -4.8165 | 1.4818904 |
| hsa-miR-608 | 4.98E-01 | 7.43E-02 | -1.816889 | -4.8296 | -0.7013604 |
| hsa-miR-193b | 4.98E-01 | 7.46E-02 | 1.814822 | -4.8331 | 0.3422693 |
| hsa-miR-124 | 4.98E-01 | 7.47E-02 | 1.814039 | -4.8345 | 0.1440765 |
| hsa-mir-668 | 4.98E-01 | 7.52E-02 | 1.81093 | -4.8398 | 0.5194478 |
| hsa-miR-220 | 4.98E-01 | 7.55E-02 | -1.808995 | -4.843 | -0.6598802 |
| hsa-miR-24-1* | 4.98E-01 | 7.55E-02 | 1.80879 | -4.8434 | 0.1324782 |
| hsa-miR-571 | 4.98E-01 | 7.71E-02 | 1.799065 | -4.8598 | 0.1055099 |
| hsa-miR-542-3p | 4.98E-01 | 7.71E-02 | 1.799042 | -4.8599 | 0.2084358 |
| hsa-miR-520a-5p | 4.98E-01 | 7.71E-02 | 1.798892 | -4.8601 | 0.1206439 |
| hsa-miR-378* | 4.98E-01 | 7.72E-02 | 1.79828 | -4.8612 | 0.1537512 |
| hsa-miR-18b | 4.98E-01 | 7.73E-02 | 1.797852 | -4.8619 | 0.1762042 |
| hsa-miR-643 | 4.98E-01 | 7.74E-02 | 1.797047 | -4.8632 | 0.2252233 |
| hsa-miR-302d* | 4.98E-01 | 7.76E-02 | -1.795793 | -4.8654 | -0.1619623 |
| hsa-miR-549 | 4.98E-01 | 7.80E-02 | 1.793094 | -4.8699 | 0.0512584 |
| hsa-miR-378 | 4.98E-01 | 7.83E-02 | 1.791203 | -4.8731 | 0.480355 |
| hsa-miR-384 | 4.98E-01 | 7.85E-02 | 1.789962 | -4.8752 | 0.1901206 |
| hsa-miR-582-3p | 4.98E-01 | 7.95E-02 | 1.78385 | -4.8854 | 0.2705107 |
| hsa-miR-145* | 4.98E-01 | 7.96E-02 | -1.783448 | -4.8861 | -0.5706247 |
| hsa-mir-596 | 4.98E-01 | 7.98E-02 | 1.782398 | -4.8878 | 0.1210594 |
| hsa-miR-630 | 4.98E-01 | 8.07E-02 | -1.776636 | -4.8975 | -0.1164716 |
| hsa-miR-144* | 4.98E-01 | 8.10E-02 | -1.77516 | -4.8999 | -0.1087131 |
| hsa-miR-526a | 4.98E-01 | 8.13E-02 | 1.773202 | -4.9032 | 0.2674986 |
| hsa-miR-137 | 4.98E-01 | 8.27E-02 | -1.764858 | -4.9171 | -0.0765663 |
| hsa-miR-631 | 4.98E-01 | 8.28E-02 | 1.764493 | -4.9177 | 0.6202393 |
| hsa-miR-153 | 4.98E-01 | 8.32E-02 | 1.761892 | -4.922 | 0.1402695 |
| hsa-mir-216a | 4.98E-01 | 8.35E-02 | -1.760086 | -4.925 | -0.7262817 |
| hsa-miR-206 | 4.98E-01 | 8.37E-02 | -1.759262 | -4.9263 | -0.6793721 |
| hsa-miR-29c* | 4.98E-01 | 8.39E-02 | -1.757628 | -4.929 | -0.3811411 |
| hsa-miR-335 | 4.98E-01 | 8.40E-02 | 1.757162 | -4.9298 | 0.2915614 |
| hsa-miR-96* | 4.98E-01 | 8.45E-02 | -1.754191 | -4.9347 | -0.1326538 |
| hsa-miR-374a* | 4.98E-01 | 8.49E-02 | 1.751816 | -4.9386 | 0.5341404 |
| hsa-mir-222 | 4.98E-01 | 8.56E-02 | -1.748214 | -4.9446 | -0.1047981 |
| hsa-mir-619 | 4.98E-01 | 8.58E-02 | -1.74681 | -4.9469 | -0.7615786 |
| hsa-miR-601 | 4.98E-01 | 8.65E-02 | -1.74313 | -4.9529 | -0.3738407 |
| hsa-miR-140-3p | 4.98E-01 | 8.80E-02 | -1.734643 | -4.9668 | -0.4477243 |
| hsa-let-7d | 4.98E-01 | 8.82E-02 | 1.733236 | -4.9691 | 0.762656 |
| hsa-miR-616 | 4.98E-01 | 9.04E-02 | -1.721402 | -4.9884 | -0.7988273 |
| hsa-mir-565 | 4.98E-01 | 9.06E-02 | 1.720347 | -4.9901 | 0.9138942 |
| hsa-miR-876-5p | 4.98E-01 | 9.06E-02 | -1.720229 | -4.9903 | -0.4027448 |
| hsa-miR-513-3p | 4.98E-01 | 9.10E-02 | -1.717794 | -4.9942 | -0.0481434 |
| hsa-miR-543 | 4.98E-01 | 9.10E-02 | -1.7177 | -4.9944 | -0.2534927 |
| hsa-miR-147 | 4.98E-01 | 9.13E-02 | 1.716311 | -4.9966 | 0.0168811 |
| hsa-miR-196a | 4.98E-01 | 9.14E-02 | -1.715833 | -4.9974 | -0.7845253 |
| hsa-miR-190 | 5.06E-01 | 9.38E-02 | 1.70281 | -5.0184 | 0.020124 |
| hsa-miR-636 | 5.06E-01 | 9.43E-02 | 1.700345 | -5.0223 | 0.3549369 |
| hsa-miR-548d-3p | 5.14E-01 | 9.74E-02 | -1.684139 | -5.0482 | -0.731481 |
| HUMTRS:CONTROL | 5.14E-01 | 9.81E-02 | 1.680683 | -5.0537 | 0.4999081 |
| hsa-miR-25 | 5.14E-01 | 9.84E-02 | 1.679103 | -5.0562 | 0.9497303 |
| hsa-miR-17* | 5.14E-01 | 9.90E-02 | 1.675928 | -5.0612 | 0.2757889 |
| hsa-miR-495 | 5.14E-01 | 1.00E-01 | -1.66986 | -5.0708 | -0.1937843 |
| hsa-miR-17 | 5.14E-01 | 1.01E-01 | 1.66816 | -5.0735 | 1.3824353 |
| hsa-mir-626 | 5.14E-01 | 1.02E-01 | -1.662089 | -5.0831 | -0.7295775 |
| hsa-mir-514-3 | 5.14E-01 | 1.02E-01 | -1.660091 | -5.0862 | -0.0258691 |
| hsa-mir-651 | 5.14E-01 | 1.02E-01 | -1.660091 | -5.0862 | -0.0258691 |
| hsa-miR-520g | 5.14E-01 | 1.02E-01 | 1.659349 | -5.0874 | 0.0170155 |
| hsa-miR-15b* | 5.17E-01 | 1.04E-01 | 1.648735 | -5.104 | 0.0164375 |
| hsa-mir-663 | 5.17E-01 | 1.05E-01 | 1.646336 | -5.1077 | 0.2742337 |
| hsa-miR-517* | 5.17E-01 | 1.06E-01 | 1.642181 | -5.1142 | 0.1321606 |
| hsa-miR-29b-2* | 5.17E-01 | 1.06E-01 | -1.64044 | -5.1169 | -0.2162615 |
| hsa-miR-325 | 5.17E-01 | 1.06E-01 | -1.640016 | -5.1175 | -0.2445365 |
| hsa-miR-146a | 5.24E-01 | 1.08E-01 | 1.630558 | -5.1322 | 1.2336138 |
| hsa-miR-760 | 5.40E-01 | 1.13E-01 | -1.609258 | -5.1649 | -0.3932777 |
| hsa-miR-596 | 5.40E-01 | 1.13E-01 | -1.607073 | -5.1682 | -0.3547415 |
| hsa-miR-138-1* | 5.40E-01 | 1.14E-01 | -1.605791 | -5.1701 | -0.1983771 |
| hsa-mir-646 | 5.46E-01 | 1.16E-01 | -1.595823 | -5.1853 | -0.4011676 |
| hsa-mir-487a | 5.46E-01 | 1.16E-01 | -1.593806 | -5.1883 | -0.3639529 |
| hsa-miR-29c | 5.47E-01 | 1.17E-01 | 1.589259 | -5.1952 | 0.8726431 |
| hsa-miR-302a | 5.49E-01 | 1.19E-01 | -1.580022 | -5.2091 | -0.3079124 |
| hsa-miR-19b | 5.49E-01 | 1.20E-01 | 1.578304 | -5.2117 | 0.0906799 |
| HSHELA01:CONTROL | 5.49E-01 | 1.20E-01 | 1.576285 | -5.2147 | 0.4531317 |
| hsa-mir-608 | 5.49E-01 | 1.21E-01 | -1.575113 | -5.2164 | -0.1804719 |
| hsa-mir-591 | 5.50E-01 | 1.22E-01 | -1.566721 | -5.229 | -0.2048451 |
| hsa-miR-362-5p | 5.50E-01 | 1.22E-01 | 1.566621 | -5.2291 | 0.0259451 |
| hsa-miR-548c-3p | 5.50E-01 | 1.24E-01 | -1.559354 | -5.2399 | -0.6745031 |
| hsa-miR-637 | 5.50E-01 | 1.25E-01 | -1.555326 | -5.2459 | -0.1806077 |
| hsa-miR-31* | 5.50E-01 | 1.25E-01 | -1.554419 | -5.2472 | -0.5079981 |
| hsa-miR-26b | 5.50E-01 | 1.26E-01 | 1.552052 | -5.2507 | 1.5724751 |
| hsa-mir-554 | 5.50E-01 | 1.27E-01 | 1.549012 | -5.2552 | 0.6330511 |
| hsa-miR-30a | 5.50E-01 | 1.27E-01 | 1.548588 | -5.2558 | 0.730878 |
| hsa-miR-346 | 5.50E-01 | 1.27E-01 | 1.547845 | -5.2569 | 0.4375675 |
| hsa-mir-448 | 5.50E-01 | 1.28E-01 | -1.544359 | -5.262 | -0.0938529 |
| hsa-mir-496 | 5.54E-01 | 1.30E-01 | -1.535485 | -5.275 | -0.3309631 |
| hsa-miR-196a* | 5.54E-01 | 1.31E-01 | -1.532739 | -5.2791 | -0.0280684 |
| hsa-miR-194 | 5.54E-01 | 1.31E-01 | 1.529577 | -5.2837 | 0.8001723 |
| hsa-miR-513-5p | 5.54E-01 | 1.31E-01 | -1.52944 | -5.2839 | -0.4860395 |
| hsa-miR-603 | 5.54E-01 | 1.32E-01 | -1.526268 | -5.2885 | -0.6591031 |
| hsa-miR-617 | 5.57E-01 | 1.34E-01 | -1.520461 | -5.2969 | -0.1112896 |
| hsa-miR-190b | 5.62E-01 | 1.35E-01 | -1.513733 | -5.3066 | -0.3468865 |
| hsa-miR-145 | 5.64E-01 | 1.37E-01 | 1.508148 | -5.3147 | 0.19259 |
| hsa-miR-96 | 5.66E-01 | 1.38E-01 | -1.502713 | -5.3225 | -0.5932272 |
| hsa-mir-595 | 5.66E-01 | 1.39E-01 | -1.50135 | -5.3244 | -0.4207444 |
| hsa-mir-634 | 5.70E-01 | 1.41E-01 | -1.493102 | -5.3362 | -0.5786666 |
| hsa-miR-208b | 5.70E-01 | 1.42E-01 | 1.48975 | -5.341 | 0.1989598 |
| hsa-miR-551a | 5.70E-01 | 1.42E-01 | 1.487906 | -5.3436 | 0.5882677 |
| hsa-miR-139-5p | 5.70E-01 | 1.43E-01 | 1.483993 | -5.3491 | 0.1709393 |
| hsa-miR-101 | 5.70E-01 | 1.43E-01 | 1.483158 | -5.3503 | 0.2591926 |
| hsa-miR-607 | 5.79E-01 | 1.46E-01 | 1.471878 | -5.3662 | 0.5675002 |
| hsa-miR-519e* | 5.86E-01 | 1.49E-01 | 1.462768 | -5.379 | 0.5320041 |
| HUMTRV1A:CONTROL | 5.87E-01 | 1.50E-01 | 1.459003 | -5.3842 | 0.3618942 |
| hsa-mir-184 | 5.95E-01 | 1.53E-01 | -1.448407 | -5.3989 | -0.2206074 |
| hsa-miR-526b* | 5.95E-01 | 1.54E-01 | 1.44521 | -5.4034 | 0.2142188 |
| HUMTRMI-No1:CONTROL | 5.95E-01 | 1.54E-01 | 1.443801 | -5.4053 | 0.3301579 |
| hsa-miR-184 | 5.98E-01 | 1.56E-01 | -1.437995 | -5.4133 | -0.144 |
| hsa-mir-133b | 5.98E-01 | 1.56E-01 | 1.435623 | -5.4166 | 0.1662983 |
| hsa-mir-217 | 5.99E-01 | 1.58E-01 | 1.430142 | -5.4241 | 0.0486898 |
| hsa-miR-411* | 5.99E-01 | 1.59E-01 | -1.426602 | -5.4289 | -0.0882156 |
| hsa-miR-16-2* | 5.99E-01 | 1.60E-01 | -1.424124 | -5.4323 | -0.3919947 |
| hsa-miR-519b-3p | 5.99E-01 | 1.60E-01 | 1.423886 | -5.4326 | 0.1322608 |
| hsa-miR-379* | 6.06E-01 | 1.63E-01 | 1.41122 | -5.4498 | 0.1072304 |
| hsa-miR-92b | 6.06E-01 | 1.63E-01 | 1.410863 | -5.4503 | 0.327184 |
| hsa-miR-106a | 6.06E-01 | 1.64E-01 | 1.409384 | -5.4523 | 0.9474676 |
| hsa-miR-126 | 6.06E-01 | 1.65E-01 | 1.406498 | -5.4562 | 0.379298 |
| hsa-mir-372 | 6.07E-01 | 1.66E-01 | -1.403126 | -5.4607 | -0.0254437 |
| hsa-mir-617 | 6.13E-01 | 1.68E-01 | -1.395327 | -5.4712 | -0.0911308 |
| hsa-mir-365-2 | 6.23E-01 | 1.72E-01 | 1.383512 | -5.4869 | 0.3686051 |
| hsa-miR-361-5p | 6.23E-01 | 1.73E-01 | 1.380385 | -5.491 | 0.9572755 |
| hsa-miR-662 | 6.23E-01 | 1.73E-01 | 1.378309 | -5.4938 | 0.517513 |
| hsa-miR-497 | 6.29E-01 | 1.76E-01 | 1.370484 | -5.5041 | 0.5302476 |
| hsa-miR-605 | 6.31E-01 | 1.77E-01 | -1.365068 | -5.5112 | -0.332743 |
| hsa-mir-640 | 6.31E-01 | 1.78E-01 | -1.363571 | -5.5132 | -0.5599129 |
| hsa-miR-132* | 6.51E-01 | 1.84E-01 | -1.343422 | -5.5394 | -0.4864246 |
| hsa-miR-484 | 6.56E-01 | 1.87E-01 | -1.333545 | -5.5521 | -0.1506496 |
| hsa-miR-15a | 6.56E-01 | 1.88E-01 | -1.332843 | -5.553 | -0.5076056 |
| hsa-miR-381 | 6.56E-01 | 1.89E-01 | -1.328532 | -5.5585 | -0.0791489 |
| hsa-miR-10b | 6.56E-01 | 1.89E-01 | -1.328225 | -5.5589 | -0.9746366 |
| hsa-miR-136 | 6.60E-01 | 1.92E-01 | 1.318287 | -5.5715 | 0.4756197 |
| hsa-miR-515-5p | 6.60E-01 | 1.94E-01 | 1.31281 | -5.5784 | 0.2348739 |
| hsa-miR-330-3p | 6.60E-01 | 1.94E-01 | -1.312695 | -5.5786 | -0.5412042 |
| hsa-miR-20b | 6.60E-01 | 1.94E-01 | 1.312362 | -5.579 | 0.9518049 |
| hsa-miR-23a* | 6.60E-01 | 1.95E-01 | 1.310125 | -5.5818 | 0.1403583 |
| hsa-mir-606 | 6.60E-01 | 1.95E-01 | -1.309993 | -5.582 | -0.2992513 |
| hsa-let-7c | 6.64E-01 | 1.97E-01 | 1.304076 | -5.5894 | 0.8480733 |
| hsa-miR-33b | 6.77E-01 | 2.02E-01 | 1.289362 | -5.6078 | 0.5903648 |
| hsa-miR-532-5p | 6.77E-01 | 2.03E-01 | -1.287737 | -5.6098 | -0.3671244 |
| hsa-miR-1 | 6.90E-01 | 2.08E-01 | -1.273763 | -5.627 | -0.4508954 |
| hsa-miR-34c-3p | 6.90E-01 | 2.09E-01 | -1.270829 | -5.6306 | -0.3000834 |
| hsa-miR-502-3p | 6.90E-01 | 2.10E-01 | 1.26699 | -5.6353 | 0.4180188 |
| hsa-miR-502-5p | 6.90E-01 | 2.11E-01 | -1.264556 | -5.6383 | -0.2660646 |
| HUMTRMI-No2:CONTROL | 6.90E-01 | 2.12E-01 | 1.262222 | -5.6411 | 0.5305789 |
| hsa-miR-520d-3p | 6.90E-01 | 2.12E-01 | -1.26138 | -5.6422 | -0.3370104 |
| hsa-let-7b* | 6.90E-01 | 2.15E-01 | -1.254295 | -5.6508 | -0.1476608 |
| hsa-miR-324-5p | 6.90E-01 | 2.15E-01 | 1.254279 | -5.6508 | 0.1734588 |
| hsa-let-7i | 6.90E-01 | 2.15E-01 | -1.253597 | -5.6516 | -0.2209377 |
| hsa-miR-615-5p | 6.90E-01 | 2.17E-01 | 1.248011 | -5.6583 | 0.3516275 |
| hsa-miR-520a-3p | 6.90E-01 | 2.18E-01 | -1.245251 | -5.6617 | -0.3876261 |
| hsa-miR-449a | 6.90E-01 | 2.18E-01 | 1.245076 | -5.6619 | 0.3219419 |
| hsa-miR-30d | 6.90E-01 | 2.18E-01 | 1.243941 | -5.6632 | 0.5383602 |
| hsa-miR-650 | 6.92E-01 | 2.20E-01 | -1.240378 | -5.6675 | -0.6039619 |
| hsa-miR-26a-2* | 6.94E-01 | 2.21E-01 | -1.236341 | -5.6723 | -0.5418035 |
| hsa-mir-329-2 | 6.98E-01 | 2.26E-01 | 1.223864 | -5.6871 | 0.2965937 |
| hsa-miR-200c* | 6.98E-01 | 2.26E-01 | 1.223716 | -5.6873 | 0.4114345 |
| hsa-mir-194-1 | 6.98E-01 | 2.26E-01 | -1.223131 | -5.688 | -0.1245291 |
| hsa-miR-595 | 6.98E-01 | 2.27E-01 | 1.220427 | -5.6912 | 0.2058264 |
| hsa-miR-19a | 6.98E-01 | 2.28E-01 | -1.21893 | -5.693 | -0.5095193 |
| hsa-mir-128a | 6.98E-01 | 2.28E-01 | 1.218228 | -5.6938 | 0.2817282 |
| hsa-miR-652 | 7.10E-01 | 2.33E-01 | -1.205793 | -5.7083 | -0.5659997 |
| hsa-miR-302c | 7.11E-01 | 2.34E-01 | -1.202281 | -5.7124 | -0.00747 |
| hsa-miR-197 | 7.11E-01 | 2.37E-01 | -1.195496 | -5.7203 | -0.2925556 |
| hsa-miR-512-5p | 7.11E-01 | 2.38E-01 | 1.192854 | -5.7233 | 0.1862559 |
| hsa-miR-132* | 7.11E-01 | 2.39E-01 | -1.189243 | -5.7275 | -0.4870041 |
| hsa-mir-370 | 7.11E-01 | 2.40E-01 | 1.187319 | -5.7297 | 0.2445071 |
| hsa-miR-28-5p | 7.11E-01 | 2.40E-01 | -1.186056 | -5.7312 | -0.1179903 |
| hsa-miR-132 | 7.11E-01 | 2.40E-01 | 1.185899 | -5.7313 | 0.1741306 |
| hsa-miR-642 | 7.11E-01 | 2.42E-01 | 1.181693 | -5.7361 | 0.1495426 |
| hsa-miR-30c | 7.11E-01 | 2.43E-01 | 1.178898 | -5.7393 | 0.6950445 |
| hsa-mir-635 | 7.11E-01 | 2.43E-01 | -1.178366 | -5.7399 | -0.3582454 |
| hsa-miR-223 | 7.11E-01 | 2.44E-01 | 1.176765 | -5.7418 | 0.8422138 |
| hsa-miR-574-3p | 7.11E-01 | 2.44E-01 | -1.175514 | -5.7432 | -0.1552703 |
| hsa-miR-92b* | 7.11E-01 | 2.45E-01 | 1.174832 | -5.744 | 0.395928 |
| hsa-miR-495 | 7.15E-01 | 2.47E-01 | -1.168513 | -5.7511 | -0.2448057 |
| hsa-miR-130a | 7.15E-01 | 2.50E-01 | 1.162272 | -5.7582 | 0.9265398 |
| hsa-miR-340 | 7.15E-01 | 2.50E-01 | 1.16075 | -5.7599 | 0.591067 |
| hsa-miR-186 | 7.15E-01 | 2.51E-01 | -1.159928 | -5.7608 | -0.3816412 |
| hsa-miR-671-5p | 7.15E-01 | 2.51E-01 | -1.159262 | -5.7615 | -0.4140764 |
| hsa-miR-139-3p | 7.17E-01 | 2.52E-01 | -1.15566 | -5.7656 | -0.5019319 |
| hsa-miR-767-5p | 7.20E-01 | 2.55E-01 | 1.149976 | -5.7719 | 0.1479799 |
| hsa-miR-29b | 7.20E-01 | 2.55E-01 | 1.14859 | -5.7735 | 0.7466718 |
| hsa-mir-564 | 7.22E-01 | 2.58E-01 | 1.143176 | -5.7795 | 0.5453114 |
| hsa-miR-501-3p | 7.22E-01 | 2.58E-01 | -1.141447 | -5.7814 | -0.2899624 |
| hsa-miR-618 | 7.22E-01 | 2.59E-01 | -1.140274 | -5.7827 | -0.4195247 |
| hsa-miR-593* | 7.25E-01 | 2.62E-01 | -1.132955 | -5.7907 | -0.3518129 |
| hsa-let-7b | 7.25E-01 | 2.63E-01 | 1.130267 | -5.7937 | 0.2939304 |
| hsa-mir-612 | 7.25E-01 | 2.64E-01 | 1.127933 | -5.7962 | 0.4383756 |
| hsa-miR-448 | 7.25E-01 | 2.64E-01 | 1.127057 | -5.7972 | 0.4869474 |
| hsa-miR-616* | 7.25E-01 | 2.65E-01 | -1.126066 | -5.7983 | -0.4617934 |
| hsa-miR-554 | 7.26E-01 | 2.66E-01 | 1.123652 | -5.8009 | 0.144589 |
| hsa-mir-623 | 7.26E-01 | 2.67E-01 | 1.121423 | -5.8033 | 0.0952024 |
| hsa-mir-578 | 7.28E-01 | 2.68E-01 | 1.117505 | -5.8076 | 0.378188 |
| hsa-miR-483-3p | 7.28E-01 | 2.70E-01 | 1.113836 | -5.8116 | 0.2290035 |
| hsa-miR-323-5p | 7.28E-01 | 2.70E-01 | 1.113199 | -5.8123 | 0.154253 |
| hsa-miR-216b | 7.28E-01 | 2.71E-01 | -1.110555 | -5.8151 | -0.3448655 |
| hsa-miR-138 | 7.31E-01 | 2.73E-01 | -1.105978 | -5.82 | -0.4885792 |
| hsa-miR-454 | 7.35E-01 | 2.75E-01 | 1.100684 | -5.8257 | 0.0065743 |
| hsa-miR-22* | 7.36E-01 | 2.77E-01 | 1.09768 | -5.8289 | 0.441291 |
| hsa-miR-371-5p | 7.43E-01 | 2.80E-01 | -1.089284 | -5.8378 | -0.3647599 |
| hsa-miR-455-5p | 7.49E-01 | 2.84E-01 | -1.081398 | -5.8461 | -0.0593449 |
| hsa-mir-365-1 | 7.49E-01 | 2.86E-01 | -1.076668 | -5.8511 | -0.2922324 |
| hsa-miR-320 | 7.49E-01 | 2.87E-01 | -1.074994 | -5.8528 | -0.2425223 |
| hsa-mir-656 | 7.49E-01 | 2.87E-01 | -1.074833 | -5.853 | -0.4123032 |
| hsa-miR-18b* | 7.50E-01 | 2.88E-01 | -1.071454 | -5.8565 | -0.0439758 |
| hsa-miR-614 | 7.50E-01 | 2.90E-01 | 1.067266 | -5.8608 | 0.1960553 |
| hsa-miR-376a | 7.50E-01 | 2.91E-01 | -1.065694 | -5.8625 | -0.1219455 |
| hsa-miR-376a* | 7.50E-01 | 2.91E-01 | 1.064435 | -5.8638 | 0.0059291 |
| hsa-miR-130b* | 7.50E-01 | 2.92E-01 | 1.063975 | -5.8643 | 0.3512322 |
| hsa-miR-516a-5p | 7.53E-01 | 2.94E-01 | 1.059153 | -5.8692 | 0.4492386 |
| HUMTRAB:CONTROL | 7.54E-01 | 2.96E-01 | -1.055341 | -5.8731 | -0.259604 |
| hsa-miR-498 | 7.54E-01 | 2.96E-01 | -1.05376 | -5.8748 | -0.1362746 |
| hsa-miR-15b | 7.57E-01 | 2.99E-01 | -1.047523 | -5.8811 | -0.2259058 |
| hsa-miR-526b | 7.57E-01 | 2.99E-01 | -1.046975 | -5.8817 | -0.1525545 |
| hsa-miR-330-5p | 7.58E-01 | 3.02E-01 | -1.041851 | -5.8869 | -0.3836504 |
| hsa-mir-215 | 7.58E-01 | 3.02E-01 | -1.041758 | -5.887 | -0.1647473 |
| hsa-miR-411 | 7.58E-01 | 3.04E-01 | 1.036939 | -5.8919 | 0.2307808 |
| hsa-miR-326 | 7.58E-01 | 3.04E-01 | 1.036313 | -5.8925 | 0.3985104 |
| hsa-miR-191 | 7.58E-01 | 3.06E-01 | 1.032168 | -5.8967 | 0.5824455 |
| hsa-miR-575 | 7.58E-01 | 3.09E-01 | -1.026959 | -5.9019 | -0.0874869 |
| hsa-miR-99b* | 7.58E-01 | 3.09E-01 | -1.026924 | -5.9019 | -0.1950988 |
| hsa-mir-609 | 7.58E-01 | 3.10E-01 | -1.024447 | -5.9044 | -0.2634076 |
| hsa-miR-769-5p | 7.58E-01 | 3.10E-01 | -1.023269 | -5.9056 | -0.4650286 |
| hsa-miR-155 | 7.58E-01 | 3.11E-01 | 1.02218 | -5.9066 | 0.3137861 |
| hsa-mir-487b | 7.58E-01 | 3.11E-01 | -1.021158 | -5.9077 | -0.3953115 |
| hsa-miR-34a | 7.58E-01 | 3.12E-01 | -1.019438 | -5.9094 | -0.3884116 |
| hsa-mir-320 | 7.58E-01 | 3.12E-01 | -1.019084 | -5.9097 | -0.1184218 |
| hsa-mir-453 | 7.62E-01 | 3.15E-01 | -1.013645 | -5.9151 | -0.1308697 |
| hsa-miR-150 | 7.63E-01 | 3.16E-01 | 1.010367 | -5.9183 | 0.157604 |
| hsa-mir-301a | 7.71E-01 | 3.21E-01 | 1.000507 | -5.928 | 0.4044874 |
| hsa-miR-452* | 7.71E-01 | 3.22E-01 | 0.999285 | -5.9292 | 0.1078266 |
| hsa-miR-26b* | 7.74E-01 | 3.24E-01 | -0.994766 | -5.9335 | -0.3225308 |
| hsa-miR-544 | 7.75E-01 | 3.26E-01 | -0.990276 | -5.9379 | -0.13744 |
| hsa-miR-30e* | 7.75E-01 | 3.26E-01 | -0.989781 | -5.9384 | -0.2814509 |
| hsa-miR-7-2* | 7.78E-01 | 3.29E-01 | -0.983965 | -5.9439 | -0.1527414 |
| hsa-miR-30e | 7.78E-01 | 3.30E-01 | 0.98267 | -5.9452 | 0.5599783 |
| hsa-miR-22 | 7.78E-01 | 3.31E-01 | 0.98098 | -5.9468 | 0.2660276 |
| hsa-miR-647 | 7.81E-01 | 3.34E-01 | -0.973407 | -5.954 | -0.0766476 |
| hsa-miR-375 | 7.81E-01 | 3.34E-01 | -0.973231 | -5.9542 | -0.3990258 |
| hsa-miR-598 | 7.81E-01 | 3.36E-01 | -0.97072 | -5.9566 | -0.1411373 |
| hsa-miR-301a | 7.81E-01 | 3.36E-01 | -0.969367 | -5.9578 | -0.1165525 |
| hsa-miR-555 | 7.81E-01 | 3.37E-01 | 0.96692 | -5.9601 | 0.0507014 |
| Human GAPDH:CONTROL | 7.81E-01 | 3.39E-01 | 0.964305 | -5.9626 | 0.347155 |
| hsa-miR-432 | 7.81E-01 | 3.39E-01 | -0.963755 | -5.9631 | -0.0621588 |
| hsa-mir-325 | 7.81E-01 | 3.40E-01 | -0.962159 | -5.9646 | -0.0298061 |
| hsa-miR-421 | 7.87E-01 | 3.44E-01 | -0.954784 | -5.9715 | -0.1412926 |
| hsa-miR-99b | 7.87E-01 | 3.45E-01 | -0.952142 | -5.974 | -0.5427235 |
| hsa-miR-148a | 7.87E-01 | 3.45E-01 | 0.951143 | -5.9749 | 0.1945227 |
| hsa-miR-24 | 7.87E-01 | 3.46E-01 | 0.949374 | -5.9765 | 0.5661842 |
| hsa-miR-133a | 7.92E-01 | 3.49E-01 | -0.94325 | -5.9822 | -0.1703681 |
| hsa-miR-200a | 7.92E-01 | 3.51E-01 | 0.940833 | -5.9844 | 0.5391289 |
| hsa-mir-637 | 7.93E-01 | 3.52E-01 | -0.937682 | -5.9873 | -0.3905841 |
| hsa-miR-125b-1* | 7.94E-01 | 3.56E-01 | -0.931161 | -5.9932 | -0.1672188 |
| hsa-miR-199a-5p | 7.94E-01 | 3.57E-01 | -0.928723 | -5.9955 | -0.4261296 |
| hsa-miR-453 | 7.94E-01 | 3.58E-01 | -0.926906 | -5.9971 | -0.380837 |
| hsa-miR-593 | 7.94E-01 | 3.59E-01 | -0.923965 | -5.9998 | -0.1510457 |
| hsa-miR-765 | 7.94E-01 | 3.60E-01 | -0.922476 | -6.0011 | -0.0155819 |
| hsa-mir-650 | 7.94E-01 | 3.60E-01 | -0.922228 | -6.0013 | -0.1433268 |
| hsa-miR-361-3p | 7.94E-01 | 3.60E-01 | -0.921868 | -6.0016 | -0.2247055 |
| hsa-miR-222 | 7.94E-01 | 3.60E-01 | -0.92179 | -6.0017 | -0.3335073 |
| hsa-let-7g | 7.94E-01 | 3.62E-01 | 0.919082 | -6.0041 | 0.0338502 |
| hsa-miR-410 | 7.94E-01 | 3.63E-01 | -0.917469 | -6.0056 | -0.3520676 |
| hsa-miR-211 | 7.97E-01 | 3.66E-01 | 0.910993 | -6.0114 | 0.040594 |
| hsa-miR-488* | 7.97E-01 | 3.66E-01 | -0.910209 | -6.0121 | -0.116418 |
| hsa-miR-24 | 7.97E-01 | 3.67E-01 | -0.908568 | -6.0135 | -0.2602081 |
| hsa-miR-219-5p | 7.97E-01 | 3.68E-01 | 0.907372 | -6.0146 | 0.2634257 |
| hsa-miR-520d-5p | 7.98E-01 | 3.69E-01 | -0.904313 | -6.0173 | -0.1063003 |
| hsa-mir-600 | 8.02E-01 | 3.74E-01 | -0.896128 | -6.0245 | -0.0981796 |
| hsa-miR-218 | 8.02E-01 | 3.74E-01 | -0.894945 | -6.0255 | -0.0842949 |
| hsa-miR-204 | 8.02E-01 | 3.76E-01 | -0.892867 | -6.0273 | -0.0994776 |
| hsa-miR-515-3p | 8.02E-01 | 3.76E-01 | 0.892028 | -6.0281 | 0.2087199 |
| hsa-miR-516b | 8.02E-01 | 3.77E-01 | -0.889829 | -6.03 | -0.1985449 |
| hsa-mir-504 | 8.02E-01 | 3.77E-01 | 0.889435 | -6.0303 | 0.3815013 |
| hsa-miR-328 | 8.03E-01 | 3.79E-01 | -0.885868 | -6.0334 | -0.2400093 |
| hsa-mir-618 | 8.03E-01 | 3.81E-01 | 0.883445 | -6.0355 | 0.0979821 |
| hsa-mir-421 | 8.03E-01 | 3.81E-01 | 0.882885 | -6.036 | 0.0947378 |
| hsa-mir-181d | 8.03E-01 | 3.82E-01 | 0.880043 | -6.0384 | 0.1065706 |
| hsa-miR-452 | 8.03E-01 | 3.83E-01 | -0.878629 | -6.0397 | -0.2537992 |
| hsa-mir-801 | 8.10E-01 | 3.88E-01 | -0.870358 | -6.0467 | -0.2630668 |
| hsa-miR-758 | 8.11E-01 | 3.92E-01 | -0.86248 | -6.0534 | -0.0740678 |
| hsa-miR-610 | 8.11E-01 | 3.92E-01 | -0.861571 | -6.0542 | -0.0713952 |
| hsa-miR-193a-3p | 8.11E-01 | 3.94E-01 | 0.858015 | -6.0572 | 0.2113867 |
| hsa-miR-202* | 8.11E-01 | 3.96E-01 | 0.854803 | -6.0599 | 0.1741299 |
| hsa-miR-511 | 8.11E-01 | 3.96E-01 | 0.854708 | -6.0599 | 0.0671818 |
| hsa-miR-362-3p | 8.11E-01 | 3.97E-01 | 0.853759 | -6.0607 | 0.4352657 |
| hsa-miR-581 | 8.11E-01 | 3.98E-01 | 0.851724 | -6.0624 | 0.0559822 |
| hsa-miR-483-5p | 8.11E-01 | 3.99E-01 | -0.850118 | -6.0638 | -0.0787992 |
| hsa-miR-660 | 8.11E-01 | 4.00E-01 | 0.847882 | -6.0656 | 0.4017159 |
| hsa-miR-548a-3p | 8.11E-01 | 4.00E-01 | -0.847696 | -6.0658 | -0.3203048 |
| hsa-mir-613 | 8.11E-01 | 4.01E-01 | 0.846323 | -6.0669 | 0.1720666 |
| hsa-miR-539 | 8.11E-01 | 4.01E-01 | -0.846251 | -6.067 | -0.1412407 |
| hsa-miR-34b* | 8.11E-01 | 4.02E-01 | -0.844889 | -6.0681 | -0.2220834 |
| hsa-miR-611 | 8.13E-01 | 4.03E-01 | -0.841664 | -6.0708 | -0.1277473 |
| hsa-miR-101* | 8.15E-01 | 4.05E-01 | 0.837898 | -6.0738 | 0.0290711 |
| hsa-miR-363* | 8.15E-01 | 4.07E-01 | 0.835851 | -6.0755 | 0.2989252 |
| hsa-miR-613 | 8.15E-01 | 4.07E-01 | -0.83448 | -6.0766 | -0.287227 |
| hsa-miR-34b | 8.19E-01 | 4.11E-01 | -0.828687 | -6.0814 | -0.211951 |
| hsa-miR-374b | 8.25E-01 | 4.14E-01 | -0.821944 | -6.0868 | -0.120577 |
| hsa-miR-492 | 8.25E-01 | 4.16E-01 | -0.819516 | -6.0888 | -0.1033603 |
| hsa-miR-551b | 8.30E-01 | 4.19E-01 | 0.813309 | -6.0937 | 0.0912242 |
| hsa-miR-138-1* | 8.32E-01 | 4.22E-01 | -0.808431 | -6.0976 | -0.136541 |
| hsa-miR-674 | 8.32E-01 | 4.23E-01 | -0.807478 | -6.0983 | -0.1289692 |
| hsa-miR-185* | 8.32E-01 | 4.23E-01 | -0.806012 | -6.0995 | -0.2095189 |
| hsa-miR-545* | 8.33E-01 | 4.25E-01 | -0.803533 | -6.1015 | -0.0911958 |
| hsa-miR-338-5p | 8.39E-01 | 4.30E-01 | -0.794307 | -6.1087 | -0.1576064 |
| hsa-miR-518c* | 8.39E-01 | 4.32E-01 | 0.7912 | -6.1111 | 0.096519 |
| hsa-miR-183 | 8.39E-01 | 4.32E-01 | -0.790756 | -6.1114 | -0.1582389 |
| hsa-miR-409-5p | 8.39E-01 | 4.32E-01 | -0.790331 | -6.1118 | -0.0757865 |
| hsa-miR-376b | 8.39E-01 | 4.33E-01 | -0.788603 | -6.1131 | -0.0990798 |
| hsa-mir-544 | 8.39E-01 | 4.35E-01 | 0.786628 | -6.1146 | 0.0040079 |
| hsa-miR-182 | 8.39E-01 | 4.35E-01 | -0.785308 | -6.1156 | -0.1068639 |
| hsa-miR-30c-1* | 8.42E-01 | 4.38E-01 | -0.780185 | -6.1196 | -0.2987374 |
| hsa-miR-423-5p | 8.44E-01 | 4.40E-01 | 0.776542 | -6.1224 | 0.1571477 |
| hsa-miR-507 | 8.51E-01 | 4.45E-01 | -0.768545 | -6.1284 | -0.0602119 |
| hsa-mir-766 | 8.56E-01 | 4.49E-01 | 0.762461 | -6.133 | 0.3137721 |
| hsa-miR-486-5p | 8.63E-01 | 4.54E-01 | -0.754094 | -6.1392 | -0.2766636 |
| hsa-miR-766 | 8.65E-01 | 4.57E-01 | -0.747863 | -6.1438 | -0.1013857 |
| hsa-miR-23a | 8.65E-01 | 4.58E-01 | 0.746907 | -6.1445 | 0.6516368 |
| hsa-miR-214* | 8.65E-01 | 4.59E-01 | 0.745036 | -6.1459 | 0.1494294 |
| hsa-miR-592 | 8.65E-01 | 4.60E-01 | -0.744072 | -6.1466 | -0.25576 |
| hsa-miR-494 | 8.65E-01 | 4.61E-01 | 0.742504 | -6.1477 | 0.233931 |
| hsa-miR-27a* | 8.65E-01 | 4.66E-01 | -0.733892 | -6.1539 | -0.0380749 |
| hsa-miR-491-3p | 8.65E-01 | 4.66E-01 | 0.732992 | -6.1546 | 0.1981209 |
| hsa-miR-451 | 8.65E-01 | 4.68E-01 | 0.730461 | -6.1564 | 0.5202571 |
| HUMTRN:CONTROL | 8.65E-01 | 4.68E-01 | 0.729776 | -6.1569 | 0.2250206 |
| hsa-miR-33a | 8.65E-01 | 4.70E-01 | 0.726814 | -6.159 | 0.0555149 |
| hsa-miR-767-3p | 8.65E-01 | 4.70E-01 | 0.726319 | -6.1594 | 0.1037627 |
| hsa-miR-487a | 8.65E-01 | 4.73E-01 | -0.721427 | -6.1628 | -0.1502099 |
| hsa-miR-649 | 8.65E-01 | 4.74E-01 | -0.720687 | -6.1634 | -0.2415528 |
| hsa-miR-25* | 8.65E-01 | 4.74E-01 | -0.719995 | -6.1639 | -0.2809827 |
| hsa-miR-100 | 8.65E-01 | 4.75E-01 | 0.719556 | -6.1642 | 0.4459161 |
| hsa-mir-489 | 8.65E-01 | 4.76E-01 | 0.717416 | -6.1657 | 0.3108164 |
| hsa-miR-330-5p | 8.65E-01 | 4.76E-01 | 0.71701 | -6.166 | 0.152338 |
| hsa-miR-24-2* | 8.65E-01 | 4.77E-01 | -0.715146 | -6.1673 | -0.0668514 |
| hsa-miR-559 | 8.65E-01 | 4.79E-01 | -0.713118 | -6.1687 | -0.0649714 |
| hsa-mir-583 | 8.65E-01 | 4.79E-01 | -0.712994 | -6.1688 | -0.0358068 |
| hsa-mir-375 | 8.65E-01 | 4.85E-01 | 0.702788 | -6.1759 | 0.1774981 |
| hsa-miR-99a | 8.65E-01 | 4.86E-01 | -0.701251 | -6.1769 | -0.4116012 |
| hsa-miR-558 | 8.65E-01 | 4.87E-01 | -0.699904 | -6.1779 | -0.2404837 |
| hsa-miR-16-1* | 8.65E-01 | 4.88E-01 | -0.698342 | -6.1789 | -0.1068475 |
| hsa-miR-135a | 8.65E-01 | 4.88E-01 | -0.697803 | -6.1793 | -0.2970261 |
| hsa-mir-495 | 8.65E-01 | 4.89E-01 | -0.696859 | -6.1799 | -0.0961111 |
| hsa-miR-181a | 8.65E-01 | 4.89E-01 | -0.695739 | -6.1807 | -0.4072675 |
| hsa-miR-801 | 8.65E-01 | 4.93E-01 | 0.689149 | -6.1852 | 0.3104848 |
| hsa-mir-224 | 8.65E-01 | 4.94E-01 | -0.688575 | -6.1856 | -0.0662973 |
| hsa-miR-181b | 8.65E-01 | 4.94E-01 | -0.687843 | -6.1861 | -0.5106657 |
| hsa-mir-653 | 8.65E-01 | 4.95E-01 | 0.687149 | -6.1865 | 0.0669443 |
| hsa-miR-129-5p | 8.65E-01 | 4.96E-01 | -0.685206 | -6.1879 | -0.0892154 |
| hsa-miR-671-3p | 8.65E-01 | 4.98E-01 | 0.681558 | -6.1903 | 0.2568229 |
| hsa-miR-29b-1* | 8.65E-01 | 5.02E-01 | -0.675673 | -6.1942 | -0.1500589 |
| hsa-miR-433 | 8.65E-01 | 5.04E-01 | -0.672147 | -6.1966 | -0.2918884 |
| hsa-miR-493* | 8.65E-01 | 5.04E-01 | -0.671646 | -6.1969 | -0.2201514 |
| hsa-mir-376a-2 | 8.65E-01 | 5.07E-01 | -0.667992 | -6.1993 | -0.0765292 |
| hsa-mir-575 | 8.65E-01 | 5.07E-01 | -0.667262 | -6.1998 | -0.076786 |
| hsa-miR-579 | 8.65E-01 | 5.07E-01 | 0.667207 | -6.1998 | 0.119675 |
| hsa-miR-656 | 8.65E-01 | 5.07E-01 | -0.667002 | -6.1999 | -0.0506456 |
| hsa-miR-374b* | 8.65E-01 | 5.14E-01 | 0.656451 | -6.2068 | 0.0620696 |
| hsa-miR-518a-5p | 8.65E-01 | 5.14E-01 | 0.656267 | -6.2069 | 0.1182726 |
| hsa-miR-136* | 8.65E-01 | 5.14E-01 | -0.656135 | -6.207 | -0.0252679 |
| hsa-miR-342-5p | 8.65E-01 | 5.15E-01 | 0.65526 | -6.2076 | 0.2029196 |
| hsa-miR-140-5p | 8.65E-01 | 5.15E-01 | 0.654809 | -6.2079 | 0.0052152 |
| hsa-miR-519c-5p | 8.65E-01 | 5.18E-01 | 0.64982 | -6.2111 | 0.0054764 |
| hsa-mir-376b | 8.65E-01 | 5.19E-01 | 0.648044 | -6.2122 | 0.0487709 |
| hsa-miR-126 | 8.65E-01 | 5.22E-01 | 0.644113 | -6.2147 | 0.2752771 |
| hsa-miR-338-3p | 8.65E-01 | 5.29E-01 | -0.633851 | -6.2211 | -0.1617519 |
| hsa-miR-21* | 8.65E-01 | 5.29E-01 | 0.633653 | -6.2213 | 0.1989491 |
| hsa-mir-329-1 | 8.65E-01 | 5.32E-01 | 0.628639 | -6.2244 | 0.1742161 |
| hsa-miR-335* | 8.65E-01 | 5.34E-01 | -0.626165 | -6.2259 | -0.0579672 |
| hsa-mir-630 | 8.65E-01 | 5.35E-01 | -0.623535 | -6.2275 | -0.0825651 |
| hsa-miR-142-5p | 8.65E-01 | 5.36E-01 | -0.622818 | -6.228 | -0.1546577 |
| hsa-mir-134 | 8.65E-01 | 5.37E-01 | -0.621081 | -6.229 | -0.1253016 |
| hsa-mir-566 | 8.65E-01 | 5.40E-01 | 0.616903 | -6.2316 | 0.2022655 |
| hsa-mir-346 | 8.65E-01 | 5.40E-01 | -0.616774 | -6.2317 | -0.0709688 |
| hsa-miR-29a* | 8.65E-01 | 5.40E-01 | -0.616758 | -6.2317 | -0.3388769 |
| hsa-mir-657 | 8.65E-01 | 5.40E-01 | 0.616391 | -6.2319 | 0.2915256 |
| hsa-miR-100* | 8.65E-01 | 5.45E-01 | -0.608362 | -6.2367 | -0.1202305 |
| hsa-miR-504 | 8.65E-01 | 5.47E-01 | 0.605154 | -6.2386 | 0.3958229 |
| hsa-miR-708 | 8.65E-01 | 5.48E-01 | 0.603968 | -6.2393 | 0.0386972 |
| hsa-miR-19a* | 8.65E-01 | 5.49E-01 | 0.603158 | -6.2398 | 0.1103053 |
| hsa-mir-602 | 8.65E-01 | 5.50E-01 | 0.601524 | -6.2408 | 0.1725443 |
| hsa-mir-621 | 8.65E-01 | 5.50E-01 | 0.601461 | -6.2408 | 0.2288545 |
| hsa-miR-20b* | 8.65E-01 | 5.56E-01 | 0.591411 | -6.2467 | 0.0389147 |
| hsa-mir-560 | 8.65E-01 | 5.59E-01 | 0.587571 | -6.249 | 0.2911658 |
| hsa-miR-552 | 8.65E-01 | 5.60E-01 | -0.586856 | -6.2494 | -0.029353 |
| hsa-miR-590-5p | 8.65E-01 | 5.60E-01 | 0.585909 | -6.2499 | 0.0385546 |
| hsa-miR-106a* | 8.65E-01 | 5.60E-01 | 0.585804 | -6.25 | 0.0102503 |
| hsa-mir-449a | 8.65E-01 | 5.61E-01 | 0.58523 | -6.2503 | 0.0167423 |
| hsa-miR-583 | 8.65E-01 | 5.61E-01 | -0.584984 | -6.2505 | -0.0909773 |
| hsa-miR-340* | 8.65E-01 | 5.62E-01 | 0.582495 | -6.2519 | 0.0242028 |
| hsa-let-7f | 8.65E-01 | 5.63E-01 | -0.581856 | -6.2522 | -0.0792518 |
| hsa-miR-497* | 8.65E-01 | 5.64E-01 | 0.580837 | -6.2528 | 0.0299585 |
| hsa-miR-587 | 8.65E-01 | 5.64E-01 | 0.579595 | -6.2535 | 0.0355158 |
| hsa-miR-588 | 8.65E-01 | 5.64E-01 | 0.579595 | -6.2535 | 0.0355158 |
| hsa-miR-324-3p | 8.65E-01 | 5.64E-01 | -0.579577 | -6.2536 | -0.1834023 |
| hsa-miR-653 | 8.65E-01 | 5.64E-01 | -0.579471 | -6.2536 | -0.0333506 |
| hsa-miR-522 | 8.65E-01 | 5.65E-01 | 0.579199 | -6.2538 | 0.0376081 |
| hsa-mir-627 | 8.65E-01 | 5.65E-01 | 0.579108 | -6.2538 | 0.0381143 |
| hsa-mir-217 | 8.65E-01 | 5.65E-01 | 0.57905 | -6.2539 | 0.038443 |
| hsa-mir-128b | 8.65E-01 | 5.65E-01 | 0.578993 | -6.2539 | 0.0387717 |
| hsa-miR-200a* | 8.65E-01 | 5.66E-01 | 0.57731 | -6.2548 | 0.0645527 |
| hsa-miR-217 | 8.65E-01 | 5.69E-01 | 0.57336 | -6.2571 | 0.0052563 |
| hsa-miR-524-5p | 8.65E-01 | 5.70E-01 | 0.571131 | -6.2583 | 0.0061322 |
| hsa-miR-363 | 8.65E-01 | 5.71E-01 | -0.570129 | -6.2589 | -0.0570613 |
| hsa-mir-598 | 8.65E-01 | 5.71E-01 | -0.56964 | -6.2592 | -0.1817774 |
| hsa-miR-519c-3p | 8.65E-01 | 5.72E-01 | 0.568484 | -6.2598 | 0.0348555 |
| hsa-miR-299-3p | 8.65E-01 | 5.73E-01 | -0.566896 | -6.2607 | -0.013317 |
| hsa-miR-565 | 8.65E-01 | 5.75E-01 | -0.564462 | -6.2621 | -0.1039603 |
| hsa-miR-454* | 8.65E-01 | 5.75E-01 | -0.563968 | -6.2623 | -0.0859564 |
| hsa-miR-591 | 8.65E-01 | 5.75E-01 | -0.563905 | -6.2624 | -0.0562506 |
| hsa-miR-130a* | 8.65E-01 | 5.75E-01 | 0.563559 | -6.2626 | 0.1959054 |
| hsa-mir-376c | 8.65E-01 | 5.76E-01 | -0.562958 | -6.2629 | -0.050044 |
| hsa-miR-148b | 8.65E-01 | 5.76E-01 | -0.562891 | -6.2629 | -0.0927971 |
| hsa-mir-662 | 8.65E-01 | 5.76E-01 | -0.56268 | -6.2631 | -0.0484938 |
| hsa-mir-568 | 8.65E-01 | 5.76E-01 | -0.562606 | -6.2631 | -0.0480946 |
| hsa-miR-569 | 8.65E-01 | 5.76E-01 | -0.56208 | -6.2634 | -0.0454677 |
| hsa-miR-875-5p | 8.65E-01 | 5.76E-01 | -0.561881 | -6.2635 | -0.0445529 |
| hsa-miR-586 | 8.65E-01 | 5.80E-01 | -0.556604 | -6.2664 | -0.0294643 |
| hsa-miR-553 | 8.65E-01 | 5.80E-01 | -0.555995 | -6.2667 | -0.1237088 |
| hsa-miR-561 | 8.65E-01 | 5.81E-01 | -0.55468 | -6.2675 | -0.0263633 |
| hsa-miR-373 | 8.65E-01 | 5.81E-01 | -0.554544 | -6.2675 | -0.0783873 |
| hsa-miR-506 | 8.65E-01 | 5.82E-01 | -0.553695 | -6.268 | -0.0250364 |
| hsa-miR-299-5p | 8.65E-01 | 5.83E-01 | -0.551378 | -6.2693 | -0.0510004 |
| hsa-miR-224 | 8.65E-01 | 5.84E-01 | 0.55119 | -6.2694 | 0.1349426 |
| hsa-miR-619 | 8.65E-01 | 5.84E-01 | -0.551145 | -6.2694 | -0.0951244 |
| hsa-miR-205 | 8.65E-01 | 5.84E-01 | -0.550071 | -6.27 | -0.1692422 |
| hsa-miR-431 | 8.65E-01 | 5.85E-01 | -0.549427 | -6.2703 | -0.0140744 |
| hsa-miR-509-5p | 8.65E-01 | 5.85E-01 | -0.548442 | -6.2708 | -0.0198786 |
| hsa-miR-648 | 8.65E-01 | 5.86E-01 | -0.54824 | -6.271 | -0.0197261 |
| hsa-miR-488 | 8.65E-01 | 5.86E-01 | 0.548123 | -6.271 | 0.0356789 |
| hsa-miR-376c | 8.65E-01 | 5.87E-01 | -0.546441 | -6.2719 | -0.0184777 |
| hsa-miR-193b* | 8.65E-01 | 5.87E-01 | -0.546271 | -6.272 | -0.0704457 |
| hsa-let-7d* | 8.65E-01 | 5.87E-01 | -0.545563 | -6.2724 | -0.2733573 |
| hsa-miR-432* | 8.65E-01 | 5.88E-01 | -0.545331 | -6.2725 | -0.0177914 |
| hsa-miR-518f* | 8.65E-01 | 5.88E-01 | -0.544407 | -6.273 | -0.017262 |
| hsa-miR-369-5p | 8.65E-01 | 5.90E-01 | -0.542307 | -6.2741 | -0.0161803 |
| hsa-miR-135b* | 8.65E-01 | 5.90E-01 | -0.541876 | -6.2744 | -0.0472052 |
| hsa-miR-629 | 8.65E-01 | 5.92E-01 | 0.539407 | -6.2757 | 0.2166167 |
| hsa-miR-585 | 8.65E-01 | 5.92E-01 | 0.539205 | -6.2758 | 0.2251386 |
| hsa-miR-490-3p | 8.65E-01 | 5.94E-01 | -0.536312 | -6.2773 | -0.0290275 |
| hsa-miR-655 | 8.65E-01 | 5.94E-01 | -0.535314 | -6.2779 | -0.0134577 |
| hsa-miR-509-3p | 8.65E-01 | 5.96E-01 | -0.532778 | -6.2792 | -0.0127042 |
| hsa-miR-519e | 8.65E-01 | 5.97E-01 | -0.531786 | -6.2797 | -0.0124342 |
| hsa-mir-433 | 8.65E-01 | 5.97E-01 | -0.531571 | -6.2798 | -0.0123776 |
| hsa-miR-182* | 8.65E-01 | 5.98E-01 | 0.530035 | -6.2806 | 0.0359959 |
| hsa-miR-330-3p | 8.65E-01 | 5.98E-01 | 0.529445 | -6.2809 | 0.203546 |
| hsa-miR-129-3p | 8.65E-01 | 6.02E-01 | 0.524024 | -6.2837 | 0.1781712 |
| hsa-miR-130b | 8.65E-01 | 6.03E-01 | -0.52259 | -6.2845 | -0.2788925 |
| hsa-mir-569 | 8.65E-01 | 6.03E-01 | -0.522484 | -6.2845 | -0.0210218 |
| hsa-miR-411* | 8.65E-01 | 6.04E-01 | -0.521777 | -6.2849 | -0.0135493 |
| hsa-miR-582-5p | 8.65E-01 | 6.04E-01 | -0.520933 | -6.2853 | -0.0095372 |
| hsa-miR-542-5p | 8.65E-01 | 6.04E-01 | -0.520722 | -6.2854 | -0.0101034 |
| hsa-mir-599 | 8.65E-01 | 6.05E-01 | -0.520562 | -6.2855 | -0.0906678 |
| hsa-miR-200b | 8.65E-01 | 6.06E-01 | 0.51869 | -6.2865 | 0.2330761 |
| hsa-miR-19b-1* | 8.65E-01 | 6.07E-01 | 0.517818 | -6.2869 | 0.0819094 |
| hsa-miR-210 | 8.68E-01 | 6.11E-01 | 0.510806 | -6.2905 | 0.1241972 |
| hsa-mir-648 | 8.68E-01 | 6.12E-01 | -0.510236 | -6.2908 | -0.0086257 |
| hsa-miR-21 | 8.68E-01 | 6.13E-01 | 0.50903 | -6.2914 | 0.4404375 |
| hsa-miR-27a | 8.68E-01 | 6.14E-01 | 0.507491 | -6.2922 | 0.3171036 |
| hsa-miR-499-3p | 8.69E-01 | 6.16E-01 | -0.504757 | -6.2935 | -0.2168554 |
| hsa-miR-32* | 8.69E-01 | 6.17E-01 | 0.50288 | -6.2945 | 0.1177325 |
| hsa-miR-135a* | 8.69E-01 | 6.19E-01 | -0.500517 | -6.2956 | -0.0174783 |
| hsa-miR-372 | 8.69E-01 | 6.19E-01 | -0.500249 | -6.2958 | -0.1672518 |
| hsa-miR-600 | 8.69E-01 | 6.20E-01 | 0.498066 | -6.2968 | 0.0033472 |
| hsa-mir-203 | 8.69E-01 | 6.21E-01 | 0.497662 | -6.297 | 0.1377567 |
| hsa-mir-614 | 8.69E-01 | 6.22E-01 | -0.496132 | -6.2978 | -0.0072381 |
| hsa-miR-194* | 8.74E-01 | 6.26E-01 | -0.489243 | -6.3011 | -0.0416917 |
| hsa-miR-302b* | 8.74E-01 | 6.28E-01 | -0.487255 | -6.3021 | -0.0510529 |
| hsa-miR-323-3p | 8.76E-01 | 6.31E-01 | -0.482434 | -6.3044 | -0.0125319 |
| hsa-miR-369-3p | 8.76E-01 | 6.31E-01 | -0.482338 | -6.3044 | -0.0705093 |
| hsa-miR-219-2-3p | 8.86E-01 | 6.41E-01 | -0.468518 | -6.3109 | -0.1668573 |
| hsa-miR-106b | 8.86E-01 | 6.42E-01 | 0.466861 | -6.3117 | 0.2858732 |
| hsa-miR-199b-3p | 8.86E-01 | 6.43E-01 | -0.466347 | -6.3119 | -0.2958831 |
| hsa-miR-563 | 8.86E-01 | 6.43E-01 | 0.465278 | -6.3124 | 0.0297549 |
| hsa-mir-492 | 8.86E-01 | 6.44E-01 | -0.464661 | -6.3127 | -0.0426705 |
| hsa-miR-512-3p | 8.87E-01 | 6.46E-01 | -0.461162 | -6.3143 | -0.0051977 |
| hsa-miR-195 | 8.90E-01 | 6.49E-01 | 0.457064 | -6.3161 | 0.1859888 |
| hsa-mir-638 | 8.90E-01 | 6.50E-01 | 0.455434 | -6.3169 | 0.00274 |
| hsa-miR-551b* | 8.90E-01 | 6.52E-01 | 0.453408 | -6.3178 | 0.2382555 |
| hsa-miR-499-5p | 8.91E-01 | 6.55E-01 | -0.449531 | -6.3195 | -0.1453047 |
| hsa-mir-658 | 8.91E-01 | 6.55E-01 | 0.448891 | -6.3198 | 0.071004 |
| hsa-miR-654-5p | 8.91E-01 | 6.56E-01 | -0.44712 | -6.3206 | -0.2049793 |
| hsa-miR-365 | 8.91E-01 | 6.57E-01 | 0.446475 | -6.3209 | 0.0022882 |
| hsa-miR-9* | 8.98E-01 | 6.63E-01 | -0.438173 | -6.3245 | -0.0070884 |
| hsa-let-7g | 9.01E-01 | 6.69E-01 | 0.42991 | -6.328 | 0.1406181 |
| hsa-miR-632 | 9.01E-01 | 6.69E-01 | -0.4291 | -6.3284 | -0.0616988 |
| hsa-miR-516b* | 9.01E-01 | 6.70E-01 | -0.428389 | -6.3287 | -0.0171722 |
| hsa-miR-744* | 9.01E-01 | 6.70E-01 | 0.427836 | -6.3289 | 0.1696906 |
| hsa-miR-34a* | 9.01E-01 | 6.75E-01 | -0.421944 | -6.3314 | -0.0200848 |
| hsa-miR-30a* | 9.01E-01 | 6.75E-01 | -0.421346 | -6.3316 | -0.114418 |
| hsa-mir-555 | 9.01E-01 | 6.76E-01 | 0.419788 | -6.3323 | 0.0324486 |
| hsa-mir-770 | 9.01E-01 | 6.76E-01 | 0.419317 | -6.3325 | 0.2067585 |
| hsa-mir-631 | 9.01E-01 | 6.77E-01 | -0.418855 | -6.3327 | -0.1848397 |
| hsa-miR-331-3p | 9.01E-01 | 6.78E-01 | -0.416635 | -6.3336 | -0.0319366 |
| hsa-miR-181c | 9.01E-01 | 6.79E-01 | 0.41617 | -6.3338 | 0.2235885 |
| hsa-miR-802 | 9.01E-01 | 6.80E-01 | 0.415133 | -6.3342 | 0.0029839 |
| hsa-miR-302b | 9.08E-01 | 6.86E-01 | -0.406643 | -6.3376 | -0.0115192 |
| hsa-miR-383 | 9.12E-01 | 6.90E-01 | 0.40121 | -6.3398 | 0.1666278 |
| hsa-miR-134 | 9.17E-01 | 6.95E-01 | 0.393997 | -6.3426 | 0.005827 |
| hsa-miR-221 | 9.18E-01 | 6.97E-01 | -0.390759 | -6.3439 | -0.2192514 |
| hsa-miR-621 | 9.18E-01 | 6.99E-01 | 0.388565 | -6.3447 | 0.0651594 |
| hsa-miR-214 | 9.18E-01 | 6.99E-01 | -0.388033 | -6.3449 | -0.1344154 |
| hsa-miR-627 | 9.19E-01 | 7.01E-01 | -0.385447 | -6.3459 | -0.2130071 |
| hsa-mir-649 | 9.20E-01 | 7.03E-01 | -0.38308 | -6.3468 | -0.0302343 |
| hsa-miR-604 | 9.20E-01 | 7.05E-01 | 0.380325 | -6.3479 | 0.0596323 |
| hsa-let-7i | 9.20E-01 | 7.05E-01 | -0.379859 | -6.348 | -0.0538435 |
| hsa-miR-132 | 9.24E-01 | 7.09E-01 | -0.374749 | -6.3499 | -0.1439448 |
| hsa-miR-10a* | 9.28E-01 | 7.14E-01 | 0.368734 | -6.3522 | 0.1218721 |
| hsa-miR-663 | 9.30E-01 | 7.17E-01 | -0.364694 | -6.3536 | -0.1966665 |
| hsa-miR-380* | 9.31E-01 | 7.18E-01 | -0.362693 | -6.3543 | -0.0346203 |
| hsa-miR-566 | 9.34E-01 | 7.23E-01 | -0.356168 | -6.3567 | -0.1354793 |
| hsa-miR-668 | 9.34E-01 | 7.23E-01 | 0.355654 | -6.3568 | 0.1176228 |
| hsa-miR-519a | 9.38E-01 | 7.29E-01 | 0.348131 | -6.3594 | 0.0227341 |
| hsa-miR-98 | 9.38E-01 | 7.30E-01 | -0.347076 | -6.3598 | -0.0509885 |
| hsa-miR-424* | 9.38E-01 | 7.30E-01 | 0.346585 | -6.36 | 0.1075672 |
| hsa-miR-105 | 9.44E-01 | 7.38E-01 | 0.336377 | -6.3634 | 0.0789335 |
| hsa-miR-560 | 9.44E-01 | 7.39E-01 | 0.335219 | -6.3638 | 0.1971947 |
| hsa-miR-125b | 9.44E-01 | 7.39E-01 | -0.334418 | -6.3641 | -0.2165243 |
| hsa-mir-597 | 9.44E-01 | 7.41E-01 | 0.332699 | -6.3646 | 0.0320381 |
| hsa-miR-181a* | 9.44E-01 | 7.41E-01 | -0.332533 | -6.3647 | -0.0066115 |
| hsa-miR-425 | 9.45E-01 | 7.43E-01 | -0.329903 | -6.3656 | -0.1245598 |
| hsa-miR-142-3p | 9.45E-01 | 7.49E-01 | -0.321102 | -6.3684 | -0.0528828 |
| hsa-mir-345 | 9.45E-01 | 7.50E-01 | 0.320114 | -6.3687 | 0.0495466 |
| hsa-miR-16 | 9.45E-01 | 7.54E-01 | 0.31427 | -6.3705 | 0.1971622 |
| hsa-let-7e* | 9.45E-01 | 7.64E-01 | -0.302014 | -6.3743 | -0.0874137 |
| hsa-miR-378* | 9.45E-01 | 7.64E-01 | 0.301408 | -6.3744 | 0.0563725 |
| hsa-miR-518b | 9.45E-01 | 7.65E-01 | -0.30026 | -6.3748 | -0.0282905 |
| hsa-miR-578 | 9.45E-01 | 7.66E-01 | -0.299371 | -6.3751 | -0.0229118 |
| hsa-miR-634 | 9.45E-01 | 7.67E-01 | -0.297509 | -6.3756 | -0.0368213 |
| hsa-miR-302d | 9.45E-01 | 7.68E-01 | 0.296532 | -6.3759 | 0.0014412 |
| hsa-miR-486-5p | 9.45E-01 | 7.71E-01 | 0.293009 | -6.3769 | 0.1267325 |
| hsa-miR-125b-2* | 9.45E-01 | 7.74E-01 | 0.288037 | -6.3783 | 0.0506818 |
| hsa-mir-622 | 9.45E-01 | 7.75E-01 | 0.287439 | -6.3785 | 0.0634095 |
| hsa-miR-216a | 9.45E-01 | 7.76E-01 | 0.285173 | -6.3792 | 0.0169229 |
| hsa-miR-503 | 9.45E-01 | 7.77E-01 | 0.284501 | -6.3793 | 0.0338253 |
| hsa-miR-545 | 9.45E-01 | 7.77E-01 | -0.284162 | -6.3794 | -0.0218713 |
| hsa-mir-660 | 9.45E-01 | 7.79E-01 | 0.281438 | -6.3802 | 0.0529986 |
| hsa-miR-639 | 9.45E-01 | 7.83E-01 | 0.276311 | -6.3816 | 0.0240374 |
| hsa-miR-374a | 9.45E-01 | 7.85E-01 | -0.274688 | -6.3821 | -0.0717602 |
| hsa-miR-345 | 9.45E-01 | 7.86E-01 | -0.273116 | -6.3825 | -0.1240882 |
| hsa-miR-532-3p | 9.45E-01 | 7.86E-01 | 0.273072 | -6.3825 | 0.0918044 |
| hsa-miR-7-1* | 9.45E-01 | 7.88E-01 | 0.270701 | -6.3831 | 0.0193388 |
| hsa-miR-651 | 9.45E-01 | 7.88E-01 | 0.270448 | -6.3832 | 0.1172544 |
| hsa-let-7a* | 9.45E-01 | 7.88E-01 | -0.270317 | -6.3832 | -0.0013184 |
| hsa-miR-183* | 9.45E-01 | 7.90E-01 | -0.267647 | -6.3839 | -0.025687 |
| hsa-miR-744 | 9.45E-01 | 7.91E-01 | 0.266217 | -6.3843 | 0.1218837 |
| hsa-miR-615-3p | 9.45E-01 | 7.96E-01 | -0.259862 | -6.386 | -0.091416 |
| hsa-miR-302a* | 9.45E-01 | 7.97E-01 | 0.258135 | -6.3864 | 0.018976 |
| hsa-miR-339-5p | 9.45E-01 | 7.98E-01 | 0.257621 | -6.3866 | 0.1177062 |
| hsa-miR-152 | 9.45E-01 | 7.99E-01 | 0.25516 | -6.3872 | 0.0495162 |
| hsa-miR-92a | 9.45E-01 | 8.00E-01 | -0.254592 | -6.3873 | -0.0663415 |
| hsa-miR-489 | 9.45E-01 | 8.00E-01 | 0.254239 | -6.3874 | 0.0246303 |
| hsa-miR-525-5p | 9.45E-01 | 8.05E-01 | 0.248463 | -6.3888 | 0.051044 |
| hsa-miR-589* | 9.45E-01 | 8.05E-01 | 0.247442 | -6.3891 | 0.0783322 |
| hsa-miR-519d | 9.45E-01 | 8.13E-01 | -0.237153 | -6.3916 | -0.0406962 |
| hsa-miR-450a | 9.45E-01 | 8.16E-01 | 0.23394 | -6.3923 | 0.0574123 |
| hsa-miR-27b | 9.45E-01 | 8.17E-01 | 0.232813 | -6.3926 | 0.1295491 |
| hsa-miR-10a | 9.45E-01 | 8.19E-01 | -0.230234 | -6.3932 | -0.1152753 |
| hsa-mir-211 | 9.45E-01 | 8.20E-01 | -0.228893 | -6.3935 | -0.0392446 |
| hsa-miR-196b | 9.45E-01 | 8.22E-01 | -0.225715 | -6.3942 | -0.0052713 |
| hsa-miR-431* | 9.45E-01 | 8.25E-01 | 0.221678 | -6.3951 | 0.0191439 |
| hsa-miR-208 | 9.45E-01 | 8.26E-01 | 0.22125 | -6.3952 | 0.0010653 |
| hsa-miR-141 | 9.45E-01 | 8.27E-01 | -0.219375 | -6.3956 | -0.1028019 |
| hsa-miR-490-5p | 9.45E-01 | 8.28E-01 | -0.218357 | -6.3958 | -0.0048725 |
| hsa-mir-7-3 | 9.45E-01 | 8.28E-01 | -0.217799 | -6.3959 | -0.0876255 |
| hsa-miR-524-3p | 9.45E-01 | 8.30E-01 | -0.215523 | -6.3964 | -0.0308854 |
| hsa-miR-370 | 9.45E-01 | 8.30E-01 | -0.215365 | -6.3964 | -0.0917267 |
| hsa-miR-485-3p | 9.45E-01 | 8.34E-01 | 0.21101 | -6.3974 | 0.0701738 |
| hsa-miR-661 | 9.45E-01 | 8.36E-01 | 0.207788 | -6.398 | 0.0670763 |
| hsa-miR-31 | 9.45E-01 | 8.37E-01 | 0.206265 | -6.3983 | 0.0762794 |
| hsa-mir-661 | 9.45E-01 | 8.42E-01 | -0.200282 | -6.3995 | -0.1079637 |
| hsa-miR-200b* | 9.45E-01 | 8.43E-01 | 0.199135 | -6.3998 | 0.0153138 |
| hsa-miR-455-3p | 9.45E-01 | 8.43E-01 | -0.198628 | -6.3999 | -0.0307395 |
| hsa-miR-654-3p | 9.45E-01 | 8.43E-01 | -0.198285 | -6.3999 | -0.0622325 |
| hsa-miR-520f | 9.45E-01 | 8.45E-01 | 0.196186 | -6.4003 | 0.0145945 |
| hsa-mir-212 | 9.45E-01 | 8.46E-01 | -0.195075 | -6.4006 | -0.0545742 |
| hsa-mir-633 | 9.45E-01 | 8.46E-01 | 0.194926 | -6.4006 | 0.0590626 |
| hsa-miR-501-5p | 9.45E-01 | 8.47E-01 | 0.19433 | -6.4007 | 0.01882 |
| hsa-miR-564 | 9.45E-01 | 8.48E-01 | 0.191929 | -6.4012 | 0.0393165 |
| hsa-miR-622 | 9.45E-01 | 8.56E-01 | -0.182425 | -6.4029 | -0.0903634 |
| hsa-miR-18a* | 9.45E-01 | 8.56E-01 | 0.182147 | -6.403 | 0.0124691 |
| hsa-mir-135a-2 | 9.45E-01 | 8.59E-01 | 0.178208 | -6.4037 | 0.0008502 |
| hsa-miR-30d* | 9.45E-01 | 8.60E-01 | -0.176653 | -6.4039 | -0.0171053 |
| hsa-miR-409-5p | 9.45E-01 | 8.61E-01 | -0.176241 | -6.404 | -0.0009746 |
| hsa-miR-628-3p | 9.45E-01 | 8.61E-01 | -0.176241 | -6.404 | -0.0009746 |
| hsa-miR-200c | 9.45E-01 | 8.63E-01 | -0.173282 | -6.4045 | -0.1018573 |
| hsa-miR-329 | 9.45E-01 | 8.69E-01 | -0.165963 | -6.4058 | -0.0009215 |
| hsa-miR-423-3p | 9.45E-01 | 8.71E-01 | -0.162947 | -6.4063 | -0.0455217 |
| hsa-miR-212 | 9.45E-01 | 8.71E-01 | -0.162928 | -6.4063 | -0.0263975 |
| hsa-miR-15a* | 9.45E-01 | 8.71E-01 | 0.162921 | -6.4063 | 0.0117896 |
| hsa-miR-296-3p | 9.45E-01 | 8.71E-01 | 0.162763 | -6.4063 | 0.0160362 |
| hsa-miR-612 | 9.45E-01 | 8.74E-01 | -0.159883 | -6.4067 | -0.0317974 |
| hsa-miR-10b* | 9.45E-01 | 8.77E-01 | 0.155815 | -6.4074 | 0.0294947 |
| hsa-miR-629* | 9.45E-01 | 8.77E-01 | -0.1557 | -6.4074 | -0.0229821 |
| hsa-miR-626 | 9.45E-01 | 8.80E-01 | 0.152213 | -6.4079 | 0.0319888 |
| hsa-miR-449b | 9.45E-01 | 8.80E-01 | -0.151573 | -6.408 | -0.0008177 |
| hsa-miR-181d | 9.45E-01 | 8.80E-01 | 0.15153 | -6.408 | 0.089524 |
| hsa-miR-644 | 9.45E-01 | 8.81E-01 | 0.150955 | -6.4081 | 0.017887 |
| hsa-miR-517c | 9.45E-01 | 8.84E-01 | -0.146387 | -6.4088 | -0.0171194 |
| hsa-miR-599 | 9.45E-01 | 8.86E-01 | -0.143981 | -6.4091 | -0.0152641 |
| hsa-miR-187 | 9.45E-01 | 8.89E-01 | -0.139563 | -6.4098 | -0.0506238 |
| hsa-miR-659 | 9.45E-01 | 8.91E-01 | -0.138006 | -6.41 | -0.0028953 |
| hsa-let-7f-1* | 9.45E-01 | 8.92E-01 | 0.136198 | -6.4102 | 0.0006477 |
| hsa-miR-126* | 9.45E-01 | 8.92E-01 | 0.136198 | -6.4102 | 0.0006477 |
| hsa-miR-133b | 9.45E-01 | 8.92E-01 | 0.136198 | -6.4102 | 0.0006477 |
| hsa-miR-154* | 9.45E-01 | 8.92E-01 | 0.136198 | -6.4102 | 0.0006477 |
| hsa-miR-217 | 9.45E-01 | 8.92E-01 | 0.136198 | -6.4102 | 0.0006477 |
| hsa-miR-302c* | 9.45E-01 | 8.92E-01 | 0.136198 | -6.4102 | 0.0006477 |
| hsa-miR-34c-5p | 9.45E-01 | 8.92E-01 | 0.136198 | -6.4102 | 0.0006477 |
| hsa-miR-367* | 9.45E-01 | 8.92E-01 | 0.136198 | -6.4102 | 0.0006477 |
| hsa-miR-367 | 9.45E-01 | 8.92E-01 | 0.136198 | -6.4102 | 0.0006477 |
| hsa-miR-380 | 9.45E-01 | 8.92E-01 | 0.136198 | -6.4102 | 0.0006477 |
| hsa-miR-411 | 9.45E-01 | 8.92E-01 | 0.136198 | -6.4102 | 0.0006477 |
| hsa-miR-488* | 9.45E-01 | 8.92E-01 | 0.136198 | -6.4102 | 0.0006477 |
| hsa-miR-491-5p | 9.45E-01 | 8.92E-01 | 0.136198 | -6.4102 | 0.0006477 |
| hsa-mir-506 | 9.45E-01 | 8.92E-01 | 0.136198 | -6.4102 | 0.0006477 |
| hsa-mir-507 | 9.45E-01 | 8.92E-01 | 0.136198 | -6.4102 | 0.0006477 |
| hsa-miR-508-5p | 9.45E-01 | 8.92E-01 | 0.136198 | -6.4102 | 0.0006477 |
| hsa-miR-510 | 9.45E-01 | 8.92E-01 | 0.136198 | -6.4102 | 0.0006477 |
| hsa-miR-514 | 9.45E-01 | 8.92E-01 | 0.136198 | -6.4102 | 0.0006477 |
| hsa-miR-517b | 9.45E-01 | 8.92E-01 | 0.136198 | -6.4102 | 0.0006477 |
| hsa-miR-518c | 9.45E-01 | 8.92E-01 | 0.136198 | -6.4102 | 0.0006477 |
| hsa-miR-518d-3p | 9.45E-01 | 8.92E-01 | 0.136198 | -6.4102 | 0.0006477 |
| hsa-miR-518f | 9.45E-01 | 8.92E-01 | 0.136198 | -6.4102 | 0.0006477 |
| hsa-miR-520c-3p | 9.45E-01 | 8.92E-01 | 0.136198 | -6.4102 | 0.0006477 |
| hsa-miR-520h | 9.45E-01 | 8.92E-01 | 0.136198 | -6.4102 | 0.0006477 |
| hsa-miR-523 | 9.45E-01 | 8.92E-01 | 0.136198 | -6.4102 | 0.0006477 |
| hsa-miR-525-3p | 9.45E-01 | 8.92E-01 | 0.136198 | -6.4102 | 0.0006477 |
| hsa-miR-556-5p | 9.45E-01 | 8.92E-01 | 0.136198 | -6.4102 | 0.0006477 |
| hsa-miR-559 | 9.45E-01 | 8.92E-01 | 0.136198 | -6.4102 | 0.0006477 |
| hsa-miR-567 | 9.45E-01 | 8.92E-01 | 0.136198 | -6.4102 | 0.0006477 |
| hsa-miR-568 | 9.45E-01 | 8.92E-01 | 0.136198 | -6.4102 | 0.0006477 |
| hsa-miR-572 | 9.45E-01 | 8.92E-01 | 0.136198 | -6.4102 | 0.0006477 |
| hsa-miR-580 | 9.45E-01 | 8.92E-01 | 0.136198 | -6.4102 | 0.0006477 |
| hsa-mir-584 | 9.45E-01 | 8.92E-01 | 0.136198 | -6.4102 | 0.0006477 |
| hsa-mir-592 | 9.45E-01 | 8.92E-01 | 0.136198 | -6.4102 | 0.0006477 |
| hsa-miR-597 | 9.45E-01 | 8.92E-01 | 0.136198 | -6.4102 | 0.0006477 |
| hsa-miR-606 | 9.45E-01 | 8.92E-01 | 0.136198 | -6.4102 | 0.0006477 |
| hsa-miR-620 | 9.45E-01 | 8.92E-01 | 0.136198 | -6.4102 | 0.0006477 |
| hsa-miR-623 | 9.45E-01 | 8.92E-01 | 0.136198 | -6.4102 | 0.0006477 |
| hsa-miR-624* | 9.45E-01 | 8.92E-01 | 0.136198 | -6.4102 | 0.0006477 |
| hsa-miR-625* | 9.45E-01 | 8.92E-01 | 0.136198 | -6.4102 | 0.0006477 |
| hsa-miR-633 | 9.45E-01 | 8.92E-01 | 0.136198 | -6.4102 | 0.0006477 |
| hsa-miR-641 | 9.45E-01 | 8.92E-01 | 0.136198 | -6.4102 | 0.0006477 |
| hsa-miR-876-3p | 9.45E-01 | 8.92E-01 | 0.136198 | -6.4102 | 0.0006477 |
| hsa-miR-485-5p | 9.48E-01 | 8.96E-01 | 0.130775 | -6.4109 | 0.0434847 |
| hsa-miR-23b | 9.48E-01 | 8.98E-01 | -0.12917 | -6.4111 | -0.054285 |
| hsa-miR-635 | 9.56E-01 | 9.06E-01 | -0.118725 | -6.4124 | -0.0328273 |
| hsa-miR-488 | 9.61E-01 | 9.12E-01 | -0.110473 | -6.4134 | -0.0065778 |
| hsa-miR-518e | 9.64E-01 | 9.17E-01 | 0.104564 | -6.414 | 0.0088183 |
| hsa-miR-127-3p | 9.64E-01 | 9.18E-01 | -0.103898 | -6.4141 | -0.0203603 |
| hsa-miR-584 | 9.70E-01 | 9.24E-01 | -0.095685 | -6.4149 | -0.0443629 |
| hsa-miR-188-5p | 9.70E-01 | 9.26E-01 | -0.093651 | -6.4151 | -0.0256293 |
| hsa-miR-203 | 9.72E-01 | 9.29E-01 | 0.089625 | -6.4154 | 0.0688915 |
| hsa-miR-577 | 9.73E-01 | 9.31E-01 | -0.086733 | -6.4157 | -0.0004439 |
| hsa-miR-573 | 9.74E-01 | 9.35E-01 | 0.081899 | -6.4161 | 0.0089826 |
| hsa-miR-646 | 9.74E-01 | 9.35E-01 | 0.081289 | -6.4161 | 0.0117473 |
| hsa-miR-412 | 9.74E-01 | 9.36E-01 | -0.080886 | -6.4162 | -0.0460001 |
| hsa-mir-765 | 9.75E-01 | 9.37E-01 | 0.079089 | -6.4163 | 0.0234449 |
| hsa-miR-216b | 9.76E-01 | 9.40E-01 | 0.075122 | -6.4166 | 0.0131488 |
| hsa-miR-26a-1* | 9.76E-01 | 9.42E-01 | -0.072679 | -6.4168 | -0.0129771 |
| hsa-mir-611 | 9.76E-01 | 9.42E-01 | -0.072468 | -6.4168 | -0.0179931 |
| hsa-miR-202 | 9.76E-01 | 9.45E-01 | -0.069815 | -6.417 | -0.0171857 |
| hsa-miR-770-5p | 9.76E-01 | 9.45E-01 | 0.068995 | -6.417 | 0.0175636 |
| hsa-miR-377 | 9.76E-01 | 9.46E-01 | -0.068251 | -6.4171 | -0.0165511 |
| hsa-mir-133b | 9.78E-01 | 9.49E-01 | -0.06394 | -6.4174 | -0.0036199 |
| hsa-miR-124* | 9.78E-01 | 9.50E-01 | -0.062495 | -6.4175 | -0.021227 |
| hsa-miR-493 | 9.79E-01 | 9.53E-01 | -0.059605 | -6.4176 | -0.0235044 |
| hsa-miR-21 | 9.79E-01 | 9.54E-01 | -0.057406 | -6.4178 | -0.0045948 |
| hsa-miR-125a-3p | 9.81E-01 | 9.57E-01 | -0.054448 | -6.4179 | -0.0149162 |
| hsa-mir-107 | 9.81E-01 | 9.58E-01 | 0.052432 | -6.418 | 0.0051956 |
| hsa-miR-657 | 9.85E-01 | 9.63E-01 | 0.046067 | -6.4183 | 0.0168178 |
| hsa-mir-181b-2 | 9.89E-01 | 9.69E-01 | -0.039137 | -6.4186 | -0.0104721 |
| hsa-miR-30c-2* | 9.90E-01 | 9.71E-01 | 0.036146 | -6.4188 | 0.0092653 |
| hsa-mir-553 | 9.91E-01 | 9.73E-01 | 0.033397 | -6.4188 | 0.0069054 |
| hsa-miR-106b* | 9.97E-01 | 9.82E-01 | -0.022884 | -6.4191 | -0.0076597 |
| hsa-miR-30b* | 9.97E-01 | 9.86E-01 | -0.018101 | -6.4192 | -0.0063313 |
| hsa-miR-377* | 9.97E-01 | 9.86E-01 | 0.017537 | -6.4192 | 0.002891 |
| hsa-miR-32 | 9.97E-01 | 9.86E-01 | -0.017484 | -6.4192 | -0.0046699 |
| hsa-miR-154 | 9.97E-01 | 9.87E-01 | 0.016876 | -6.4193 | 0.0000814 |
| hsa-miR-152 | 9.97E-01 | 9.89E-01 | 0.014201 | -6.4193 | 0.0024344 |
| hsa-miR-7 | 9.97E-01 | 9.90E-01 | 0.013119 | -6.4193 | 0.0078652 |
| hsa-miR-505 | 9.97E-01 | 9.92E-01 | 0.010673 | -6.4193 | 0.0040094 |
| hsa-let-7f-2* | 9.97E-01 | 9.92E-01 | -0.010654 | -6.4193 | -0.0000523 |
| hsa-mir-577 | 9.97E-01 | 9.92E-01 | -0.010609 | -6.4193 | -0.0036994 |
| hsa-mir-422a | 9.99E-01 | 9.95E-01 | -0.006352 | -6.4194 | -0.0020842 |
| hsa-mir-450a-2 | 1 | 9.99E-01 | 0.001455 | -6.4194 | 0.0001425 |
| hsa-miR-93 | 1 | 9.99E-01 | 0.001234 | -6.4194 | 0.0007684 |
| hsa-miR-508-3p | 1 | 1 | 0.000326 | -6.4194 | 0.0000304 |

**Table S2** DEMs between primary colorectal tumor and colorectal liver metastasis from GSE73178 dataset.

| ID | adj.*P*.Value | *P*.Value | t | B | logFC |
| --- | --- | --- | --- | --- | --- |
| hsa-miR-122-5p | 6.22E-40 | 2.44E-43 | 13.93382 | 85.9596 | 12.942528 |
| hsa-miR-122-3p | 9.51E-11 | 7.46E-14 | 7.50038 | 20.5929 | 6.966781 |
| hsa-miR-30a-3p | 9.79E-11 | 1.15E-13 | 7.442661 | 20.1841 | 6.913169 |
| hsa-miR-582-5p | 1.88E-08 | 2.96E-11 | -6.663508 | 14.9741 | -6.189447 |
| hsa-miR-3180-3p | 2.35E-08 | 4.60E-11 | -6.597522 | 14.5593 | -6.128155 |
| hsa-miR-144-3p | 7.37E-08 | 1.74E-10 | -6.39617 | 13.3191 | -5.941128 |
| hsa-miR-550a-3p | 1.98E-07 | 5.44E-10 | -6.217885 | 12.253 | -5.775527 |
| hsa-miR-493-5p | 2.78E-07 | 8.73E-10 | 6.14267 | 11.8123 | 5.705662 |
| hsa-miR-141-5p | 6.09E-07 | 2.15E-09 | -5.996972 | 10.9739 | -5.57033 |
| hsa-miR-379-5p | 1.23E-06 | 4.81E-09 | 5.86383 | 10.2253 | 5.44666 |
| hsa-miR-18a-5p | 1.49E-06 | 6.42E-09 | -5.815337 | 9.9569 | -5.401617 |
| hsa-miR-650 | 2.89E-06 | 1.36E-08 | -5.687663 | 9.2607 | -5.283026 |
| hsa-miR-592 | 3.16E-06 | 1.61E-08 | -5.658525 | 9.104 | -5.255961 |
| hsa-miR-4488 | 7.95E-06 | 4.36E-08 | -5.483744 | 8.1808 | -5.093614 |
| hsa-miR-1307-5p | 1.47E-05 | 8.64E-08 | -5.361057 | 7.5501 | -4.979656 |
| hsa-miR-135b-5p | 1.66E-05 | 1.04E-07 | -5.327256 | 7.3789 | -4.948259 |
| hsa-miR-204-5p | 6.74E-05 | 4.49E-07 | 5.05338 | 6.0312 | 4.693868 |
| hsa-miR-1271-5p | 8.73E-05 | 6.16E-07 | 4.992355 | 5.7406 | 4.637184 |
| hsa-miR-18b-5p | 1.38E-04 | 1.03E-06 | -4.892077 | 5.2707 | -4.544041 |
| hsa-miR-181a-2-3p | 1.99E-04 | 1.56E-06 | 4.809318 | 4.8901 | 4.467169 |
| hsa-miR-552-3p | 2.52E-04 | 2.08E-06 | -4.751048 | 4.6261 | -4.413045 |
| hsa-miR-19b-1-5p | 3.05E-04 | 2.64E-06 | -4.702282 | 4.4075 | -4.367748 |
| hsa-miR-214-5p | 3.05E-04 | 2.86E-06 | 4.685964 | 4.3349 | 4.352591 |
| hsa-miR-301a-3p | 3.05E-04 | 2.87E-06 | -4.685029 | 4.3308 | -4.351722 |
| hsa-miR-7-1-3p | 9.80E-04 | 9.61E-06 | -4.430298 | 3.2301 | -4.115113 |
| hsa-miR-370-3p | 2.11E-03 | 2.15E-05 | 4.252273 | 2.4974 | 3.949754 |
| hsa-miR-4522 | 1.49E-02 | 1.58E-04 | 3.780926 | 0.7025 | 3.51194 |
| hsa-miR-452-5p | 1.90E-02 | 2.09E-04 | -3.710387 | 0.452 | -3.446419 |
| hsa-miR-224-3p | 2.04E-02 | 2.37E-04 | -3.678203 | 0.3393 | -3.416525 |
| hsa-miR-885-5p | 2.04E-02 | 2.40E-04 | 3.675153 | 0.3287 | 3.413692 |
| hsa-miR-4783-3p | 2.06E-02 | 2.51E-04 | -3.664169 | 0.2904 | -3.403489 |
| hsa-miR-936 | 2.27E-02 | 2.85E-04 | 3.631221 | 0.1765 | 3.372886 |
| hsa-miR-139-3p | 2.73E-02 | 3.55E-04 | 3.574216 | -0.0182 | 3.319936 |
| hsa-miR-6516-3p | 2.73E-02 | 3.65E-04 | -3.566902 | -0.043 | -3.313142 |
| hsa-miR-542-5p | 3.61E-02 | 4.96E-04 | 3.485338 | -0.3156 | 3.23738 |
| hsa-miR-7159-5p | 5.17E-02 | 7.31E-04 | 3.379805 | -0.6591 | 3.139356 |
| hsa-miR-5010-5p | 5.65E-02 | 8.20E-04 | 3.347919 | -0.7608 | 3.109738 |
| hsa-miR-182-5p | 5.85E-02 | 8.73E-04 | -3.330609 | -0.8156 | -3.09366 |
| hsa-miR-20a-3p | 6.45E-02 | 9.86E-04 | -3.296298 | -0.9233 | -3.06179 |
| hsa-miR-6500-5p | 6.59E-02 | 1.08E-03 | -3.26976 | -1.0059 | -3.037139 |
| hsa-miR-1910-3p | 6.59E-02 | 1.09E-03 | -3.268248 | -1.0106 | -3.035735 |
| hsa-miR-421 | 6.59E-02 | 1.11E-03 | -3.263003 | -1.0269 | -3.030863 |
| hsa-miR-19a-3p | 6.59E-02 | 1.11E-03 | -3.26232 | -1.029 | -3.030229 |
| hsa-miR-432-5p | 6.61E-02 | 1.14E-03 | 3.254619 | -1.0528 | 3.023076 |
| hsa-miR-125b-2-3p | 6.61E-02 | 1.19E-03 | 3.243086 | -1.0883 | 3.012364 |
| hsa-miR-4513 | 6.61E-02 | 1.21E-03 | -3.238916 | -1.1011 | -3.00849 |
| hsa-miR-4746-5p | 6.61E-02 | 1.22E-03 | 3.236117 | -1.1097 | 3.00589 |
| hsa-miR-1299 | 7.14E-02 | 1.35E-03 | -3.206118 | -1.2013 | -2.978026 |
| hsa-miR-1237-3p | 7.14E-02 | 1.37E-03 | 3.201979 | -1.2139 | 2.974181 |
| hsa-miR-4450 | 7.21E-02 | 1.41E-03 | 3.19338 | -1.24 | 2.966193 |
| hsa-miR-6763-3p | 7.54E-02 | 1.51E-03 | 3.173762 | -1.2992 | 2.947971 |
| hsa-miR-410-3p | 7.54E-02 | 1.54E-03 | 3.168333 | -1.3155 | 2.942928 |
| hsa-miR-181a-3p | 7.54E-02 | 1.57E-03 | 3.163182 | -1.3309 | 2.938144 |
| hsa-miR-6880-3p | 7.54E-02 | 1.60E-03 | 3.158033 | -1.3464 | 2.933361 |
| hsa-miR-4482-3p | 7.62E-02 | 1.64E-03 | -3.149615 | -1.3715 | -2.925542 |
| hsa-miR-34a-3p | 7.95E-02 | 1.77E-03 | 3.12745 | -1.4375 | 2.904954 |
| hsa-miR-365b-5p | 7.95E-02 | 1.78E-03 | 3.126561 | -1.4401 | 2.904128 |
| hsa-miR-125b-1-3p | 7.97E-02 | 1.81E-03 | 3.120874 | -1.457 | 2.898846 |
| hsa-miR-7-5p | 8.14E-02 | 1.91E-03 | -3.104968 | -1.5039 | -2.884072 |
| hsa-miR-208a-5p | 8.14E-02 | 1.92E-03 | 3.104611 | -1.505 | 2.88374 |
| hsa-miR-221-5p | 8.20E-02 | 1.96E-03 | -3.097227 | -1.5267 | -2.876881 |
| hsa-miR-605-5p | 8.20E-02 | 2.03E-03 | 3.087046 | -1.5565 | 2.867424 |
| hsa-miR-645 | 8.20E-02 | 2.03E-03 | 3.08669 | -1.5576 | 2.867094 |
| hsa-miR-6877-3p | 8.20E-02 | 2.06E-03 | -3.083065 | -1.5682 | -2.863727 |
| hsa-miR-6872-3p | 8.32E-02 | 2.12E-03 | -3.074356 | -1.5936 | -2.855638 |
| hsa-miR-629-3p | 8.54E-02 | 2.21E-03 | -3.06175 | -1.6302 | -2.843928 |
| hsa-miR-1909-5p | 8.58E-02 | 2.25E-03 | -3.056069 | -1.6467 | -2.838651 |
| hsa-miR-16-2-3p | 8.71E-02 | 2.32E-03 | -3.047031 | -1.6729 | -2.830257 |
| hsa-miR-769-3p | 8.94E-02 | 2.42E-03 | 3.034566 | -1.7088 | 2.818679 |
| hsa-miR-31-3p | 9.31E-02 | 2.56E-03 | -3.01809 | -1.7561 | -2.803374 |
| hsa-miR-99a-5p | 1.00E-01 | 2.80E-03 | 2.990878 | -1.8336 | 2.778098 |
| hsa-miR-4472 | 1.08E-01 | 3.04E-03 | 2.965174 | -1.9062 | 2.754223 |
| hsa-miR-8087 | 1.08E-01 | 3.10E-03 | -2.959407 | -1.9224 | -2.748866 |
| hsa-miR-429 | 1.13E-01 | 3.27E-03 | -2.942334 | -1.9701 | -2.733007 |
| hsa-miR-4709-3p | 1.14E-01 | 3.34E-03 | 2.935532 | -1.9891 | 2.72669 |
| hsa-miR-196b-3p | 1.14E-01 | 3.40E-03 | -2.930268 | -2.0037 | -2.7218 |
| hsa-miR-33a-5p | 1.17E-01 | 3.53E-03 | -2.918315 | -2.0368 | -2.710697 |
| hsa-miR-6793-5p | 1.22E-01 | 3.80E-03 | 2.895491 | -2.0997 | 2.689498 |
| hsa-miR-1321 | 1.22E-01 | 3.83E-03 | 2.892929 | -2.1068 | 2.687117 |
| hsa-miR-654-5p | 1.22E-01 | 3.83E-03 | 2.892914 | -2.1068 | 2.687104 |
| hsa-miR-4649-3p | 1.22E-01 | 3.88E-03 | -2.889476 | -2.1162 | -2.683911 |
| hsa-miR-181c-3p | 1.34E-01 | 4.32E-03 | -2.855029 | -2.21 | -2.651914 |
| hsa-miR-6765-3p | 1.43E-01 | 4.65E-03 | -2.831344 | -2.2739 | -2.629914 |
| hsa-miR-23b-5p | 1.46E-01 | 4.86E-03 | 2.817531 | -2.3109 | 2.617083 |
| hsa-miR-6889-3p | 1.46E-01 | 4.88E-03 | 2.816209 | -2.3144 | 2.615856 |
| hsa-miR-153-3p | 1.50E-01 | 5.05E-03 | -2.805321 | -2.3434 | -2.605742 |
| hsa-miR-3151-3p | 1.56E-01 | 5.32E-03 | -2.788096 | -2.3891 | -2.589742 |
| hsa-miR-6788-5p | 1.60E-01 | 5.55E-03 | -2.77442 | -2.4252 | -2.57704 |
| hsa-miR-3185 | 1.60E-01 | 5.60E-03 | -2.771363 | -2.4332 | -2.5742 |
| hsa-miR-1908-3p | 1.62E-01 | 5.76E-03 | 2.762521 | -2.4564 | 2.565987 |
| hsa-miR-141-3p | 1.62E-01 | 5.83E-03 | -2.758223 | -2.4676 | -2.561995 |
| hsa-miR-4506 | 1.62E-01 | 5.86E-03 | -2.756768 | -2.4714 | -2.560644 |
| hsa-miR-3680-3p | 1.68E-01 | 6.11E-03 | -2.742824 | -2.5078 | -2.547692 |
| hsa-miR-5196-5p | 1.71E-01 | 6.49E-03 | 2.722896 | -2.5594 | 2.529181 |
| hsa-miR-24-1-5p | 1.71E-01 | 6.52E-03 | -2.721664 | -2.5626 | -2.528037 |
| hsa-miR-200a-5p | 1.71E-01 | 6.52E-03 | -2.721503 | -2.563 | -2.527887 |
| hsa-miR-6511a-5p | 1.71E-01 | 6.52E-03 | -2.721297 | -2.5635 | -2.527696 |
| hsa-miR-1290 | 1.73E-01 | 6.67E-03 | -2.713979 | -2.5824 | -2.520899 |
| hsa-miR-4740-5p | 1.74E-01 | 6.79E-03 | 2.707988 | -2.5978 | 2.515334 |
| hsa-miR-3607-5p | 1.74E-01 | 6.83E-03 | -2.706322 | -2.6021 | -2.513786 |
| hsa-miR-378g | 1.75E-01 | 6.95E-03 | -2.700536 | -2.6169 | -2.508412 |
| hsa-miR-4754 | 1.75E-01 | 7.07E-03 | 2.694809 | -2.6316 | 2.503092 |
| hsa-miR-3609 | 1.75E-01 | 7.09E-03 | -2.693462 | -2.635 | -2.501841 |
| hsa-miR-3621 | 1.75E-01 | 7.14E-03 | 2.691296 | -2.6405 | 2.49983 |
| hsa-miR-200a-3p | 1.81E-01 | 7.45E-03 | -2.677152 | -2.6765 | -2.486691 |
| hsa-miR-3912-5p | 1.84E-01 | 7.64E-03 | 2.668445 | -2.6986 | 2.478604 |
| hsa-miR-6804-5p | 1.86E-01 | 7.82E-03 | 2.660759 | -2.718 | 2.471465 |
| hsa-miR-6798-3p | 1.87E-01 | 7.93E-03 | 2.656177 | -2.7295 | 2.467209 |
| hsa-miR-1273d | 1.95E-01 | 8.38E-03 | 2.637538 | -2.7763 | 2.449895 |
| hsa-miR-6736-5p | 1.95E-01 | 8.41E-03 | 2.636059 | -2.78 | 2.448522 |
| hsa-miR-1273h-5p | 1.95E-01 | 8.63E-03 | 2.627527 | -2.8013 | 2.440597 |
| hsa-miR-6889-5p | 1.95E-01 | 8.65E-03 | 2.626538 | -2.8038 | 2.439678 |
| hsa-miR-4633-5p | 1.95E-01 | 8.66E-03 | -2.626279 | -2.8044 | -2.439438 |
| hsa-miR-6848-3p | 1.95E-01 | 8.78E-03 | 2.621682 | -2.8158 | 2.435168 |
| hsa-miR-498 | 1.95E-01 | 8.80E-03 | 2.620713 | -2.8183 | 2.434267 |
| hsa-miR-4755-3p | 2.02E-01 | 9.26E-03 | 2.603142 | -2.8618 | 2.417947 |
| hsa-miR-1285-3p | 2.02E-01 | 9.33E-03 | 2.600817 | -2.8675 | 2.415787 |
| hsa-miR-1208 | 2.02E-01 | 9.44E-03 | -2.596511 | -2.8781 | -2.411787 |
| hsa-miR-4657 | 2.02E-01 | 9.66E-03 | -2.588811 | -2.897 | -2.404636 |
| hsa-miR-660-3p | 2.02E-01 | 9.71E-03 | -2.587041 | -2.9014 | -2.402991 |
| hsa-miR-3622b-5p | 2.02E-01 | 9.72E-03 | 2.58676 | -2.9021 | 2.40273 |
| hsa-miR-6730-5p | 2.02E-01 | 9.82E-03 | 2.582933 | -2.9114 | 2.399176 |
| hsa-miR-373-5p | 2.02E-01 | 9.86E-03 | -2.581802 | -2.9142 | -2.398125 |
| hsa-miR-3687 | 2.02E-01 | 9.87E-03 | -2.581407 | -2.9152 | -2.397758 |
| hsa-miR-7111-3p | 2.02E-01 | 9.92E-03 | -2.579482 | -2.9199 | -2.39597 |
| hsa-miR-4538 | 2.03E-01 | 1.01E-02 | -2.574087 | -2.9331 | -2.390959 |
| hsa-miR-4725-5p | 2.03E-01 | 1.01E-02 | -2.573482 | -2.9345 | -2.390397 |
| hsa-miR-7854-3p | 2.04E-01 | 1.03E-02 | -2.56647 | -2.9516 | -2.383883 |
| hsa-miR-4323 | 2.04E-01 | 1.03E-02 | 2.565747 | -2.9534 | 2.383212 |
| hsa-miR-192-3p | 2.04E-01 | 1.04E-02 | -2.561893 | -2.9628 | -2.379632 |
| hsa-miR-4494 | 2.04E-01 | 1.05E-02 | -2.560683 | -2.9657 | -2.378509 |
| hsa-miR-598-5p | 2.06E-01 | 1.06E-02 | -2.555066 | -2.9793 | -2.373291 |
| hsa-miR-363-3p | 2.06E-01 | 1.07E-02 | 2.552018 | -2.9867 | 2.37046 |
| hsa-miR-4769-3p | 2.09E-01 | 1.10E-02 | 2.544656 | -3.0045 | 2.363622 |
| hsa-miR-4451 | 2.09E-01 | 1.11E-02 | 2.540329 | -3.0149 | 2.359602 |
| hsa-miR-3132 | 2.09E-01 | 1.13E-02 | 2.534419 | -3.0291 | 2.354113 |
| hsa-miR-191-5p | 2.09E-01 | 1.13E-02 | -2.534358 | -3.0293 | -2.354057 |
| hsa-miR-6832-5p | 2.09E-01 | 1.13E-02 | 2.533372 | -3.0316 | 2.353141 |
| hsa-miR-4768-3p | 2.09E-01 | 1.14E-02 | 2.531785 | -3.0355 | 2.351666 |
| hsa-miR-193b-5p | 2.13E-01 | 1.18E-02 | 2.517697 | -3.0692 | 2.338581 |
| hsa-miR-3660 | 2.13E-01 | 1.19E-02 | 2.517444 | -3.0698 | 2.338346 |
| hsa-miR-132-5p | 2.13E-01 | 1.19E-02 | -2.517246 | -3.0703 | -2.338161 |
| hsa-miR-3131 | 2.19E-01 | 1.23E-02 | 2.504504 | -3.1006 | 2.326326 |
| hsa-miR-4274 | 2.19E-01 | 1.24E-02 | -2.501624 | -3.1074 | -2.323651 |
| hsa-miR-194-3p | 2.22E-01 | 1.27E-02 | -2.494325 | -3.1247 | -2.316871 |
| hsa-miR-369-5p | 2.26E-01 | 1.30E-02 | 2.484557 | -3.1478 | 2.307798 |
| hsa-miR-1307-3p | 2.26E-01 | 1.30E-02 | -2.483625 | -3.15 | -2.306933 |
| hsa-miR-203a-3p | 2.31E-01 | 1.34E-02 | -2.473331 | -3.1741 | -2.297371 |
| hsa-miR-10a-3p | 2.31E-01 | 1.35E-02 | 2.470921 | -3.1798 | 2.295132 |
| hsa-miR-6508-5p | 2.36E-01 | 1.39E-02 | -2.461742 | -3.2013 | -2.286606 |
| hsa-miR-100-5p | 2.43E-01 | 1.44E-02 | 2.448496 | -3.2321 | 2.274303 |
| hsa-miR-19b-3p | 2.57E-01 | 1.54E-02 | -2.423947 | -3.2888 | -2.2515 |
| hsa-miR-6075 | 2.57E-01 | 1.54E-02 | -2.423467 | -3.2899 | -2.251054 |
| hsa-miR-301b-3p | 2.66E-01 | 1.61E-02 | -2.408484 | -3.3242 | -2.237138 |
| hsa-miR-148a-3p | 2.68E-01 | 1.63E-02 | -2.403292 | -3.336 | -2.232315 |
| hsa-miR-575 | 2.78E-01 | 1.70E-02 | 2.386683 | -3.3738 | 2.216887 |
| hsa-miR-196a-5p | 2.83E-01 | 1.75E-02 | -2.377532 | -3.3944 | -2.208387 |
| hsa-miR-3654 | 2.83E-01 | 1.75E-02 | 2.376056 | -3.3978 | 2.207016 |
| hsa-miR-934 | 2.88E-01 | 1.80E-02 | 2.365709 | -3.421 | 2.197406 |
| hsa-miR-23a-5p | 2.88E-01 | 1.81E-02 | -2.364823 | -3.423 | -2.196583 |
| hsa-miR-1246 | 2.90E-01 | 1.83E-02 | -2.359311 | -3.4353 | -2.191463 |
| hsa-miR-4324 | 3.31E-01 | 2.11E-02 | 2.306996 | -3.5511 | 2.14287 |
| hsa-miR-215-5p | 3.31E-01 | 2.11E-02 | -2.30603 | -3.5532 | -2.141972 |
| hsa-miR-194-5p | 3.37E-01 | 2.17E-02 | -2.297095 | -3.5727 | -2.133673 |
| hsa-miR-4776-5p | 3.76E-01 | 2.43E-02 | 2.252329 | -3.6692 | 2.092091 |
| hsa-miR-423-3p | 3.98E-01 | 2.60E-02 | -2.227385 | -3.7222 | -2.068923 |
| hsa-miR-200b-3p | 3.98E-01 | 2.61E-02 | -2.225767 | -3.7256 | -2.06742 |
| hsa-miR-6777-5p | 4.06E-01 | 2.69E-02 | 2.213599 | -3.7512 | 2.056117 |
| hsa-miR-125b-5p | 4.06E-01 | 2.69E-02 | 2.212935 | -3.7526 | 2.055501 |
| hsa-miR-345-5p | 4.13E-01 | 2.75E-02 | 2.204724 | -3.7698 | 2.047874 |
| hsa-miR-32-5p | 4.29E-01 | 2.88E-02 | -2.187284 | -3.8061 | -2.031675 |
| hsa-miR-148a-5p | 4.35E-01 | 2.93E-02 | -2.179775 | -3.8216 | -2.0247 |
| hsa-miR-192-5p | 4.42E-01 | 3.01E-02 | -2.169019 | -3.8438 | -2.014709 |
| hsa-miR-93-3p | 4.42E-01 | 3.02E-02 | -2.168173 | -3.8456 | -2.013923 |
| hsa-miR-15b-3p | 4.99E-01 | 3.43E-02 | -2.117307 | -3.9489 | -1.966675 |
| hsa-miR-183-3p | 5.01E-01 | 3.46E-02 | -2.114044 | -3.9554 | -1.963645 |
| hsa-miR-199a-5p | 5.09E-01 | 3.54E-02 | 2.10462 | -3.9743 | 1.954891 |
| hsa-miR-218-5p | 5.16E-01 | 3.60E-02 | 2.097368 | -3.9887 | 1.948155 |
| hsa-miR-542-3p | 5.17E-01 | 3.63E-02 | 2.094427 | -3.9946 | 1.945423 |
| hsa-miR-95-3p | 5.22E-01 | 3.68E-02 | -2.088097 | -4.0071 | -1.939544 |
| hsa-miR-214-3p | 5.31E-01 | 3.77E-02 | 2.078273 | -4.0265 | 1.930419 |
| hsa-miR-921 | 5.34E-01 | 3.81E-02 | -2.074294 | -4.0343 | -1.926722 |
| hsa-miR-629-5p | 5.68E-01 | 4.08E-02 | -2.046583 | -4.0885 | -1.900983 |
| hsa-miR-662 | 5.96E-01 | 4.31E-02 | -2.023661 | -4.1327 | -1.879692 |
| hsa-miR-1343-5p | 5.96E-01 | 4.32E-02 | -2.022069 | -4.1358 | -1.878213 |
| hsa-miR-431-3p | 5.98E-01 | 4.37E-02 | -2.017896 | -4.1438 | -1.874337 |
| hsa-miR-96-5p | 6.33E-01 | 4.65E-02 | -1.991728 | -4.1935 | -1.85003 |
| hsa-miR-20b-5p | 6.46E-01 | 4.76E-02 | -1.981078 | -4.2135 | -1.840139 |
| hsa-miR-20a-5p | 6.50E-01 | 4.82E-02 | -1.976108 | -4.2229 | -1.835522 |
| hsa-miR-375 | 6.62E-01 | 4.93E-02 | -1.96621 | -4.2414 | -1.826328 |
| hsa-miR-17-5p | 6.70E-01 | 5.02E-02 | -1.958453 | -4.2558 | -1.819123 |
| hsa-miR-374a-5p | 7.19E-01 | 5.44E-02 | -1.923822 | -4.3195 | -1.786956 |
| hsa-miR-196b-5p | 7.19E-01 | 5.47E-02 | -1.921913 | -4.323 | -1.785183 |
| hsa-miR-17-3p | 7.19E-01 | 5.47E-02 | -1.921602 | -4.3236 | -1.784894 |
| hsa-miR-202-3p | 7.37E-01 | 5.63E-02 | 1.908799 | -4.3468 | 1.773001 |
| hsa-miR-5699-5p | 7.72E-01 | 5.94E-02 | -1.88577 | -4.3882 | -1.751611 |
| hsa-miR-6777-3p | 8.40E-01 | 6.50E-02 | -1.845999 | -4.4586 | -1.714669 |
| hsa-miR-760 | 8.50E-01 | 6.61E-02 | -1.838459 | -4.4718 | -1.707666 |
| hsa-miR-362-3p | 9.12E-01 | 7.12E-02 | -1.804418 | -4.5305 | -1.676046 |
| hsa-miR-200c-3p | 9.53E-01 | 7.48E-02 | -1.782274 | -4.5682 | -1.655478 |
| hsa-miR-106b-5p | 9.89E-01 | 8.29E-02 | -1.734287 | -4.6482 | -1.610904 |
| hsa-miR-200b-5p | 9.89E-01 | 8.44E-02 | -1.725787 | -4.6621 | -1.603009 |
| hsa-miR-197-5p | 9.89E-01 | 9.25E-02 | -1.682485 | -4.7321 | -1.562788 |
| hsa-miR-1-3p | 9.89E-01 | 9.35E-02 | -1.677405 | -4.7402 | -1.55807 |
| hsa-miR-4687-3p | 9.89E-01 | 9.40E-02 | 1.674906 | -4.7442 | 1.555749 |
| hsa-miR-342-5p | 9.89E-01 | 9.44E-02 | 1.672953 | -4.7473 | 1.553935 |
| hsa-miR-362-5p | 9.89E-01 | 9.48E-02 | -1.670895 | -4.7505 | -1.552023 |
| hsa-miR-149-5p | 9.89E-01 | 9.52E-02 | 1.668742 | -4.7539 | 1.550023 |
| hsa-miR-424-3p | 9.89E-01 | 9.57E-02 | -1.666149 | -4.758 | -1.547614 |
| hsa-miR-501-5p | 9.89E-01 | 9.86E-02 | -1.652055 | -4.7802 | -1.534523 |
| hsa-miR-584-5p | 9.89E-01 | 1.04E-01 | 1.624843 | -4.8225 | 1.509247 |
| hsa-miR-198 | 9.89E-01 | 1.06E-01 | 1.616122 | -4.8359 | 1.501146 |
| hsa-miR-4739 | 9.89E-01 | 1.13E-01 | 1.586351 | -4.8811 | 1.473493 |
| hsa-miR-4673 | 9.89E-01 | 1.17E-01 | 1.56743 | -4.9093 | 1.455919 |
| hsa-miR-502-5p | 9.89E-01 | 1.17E-01 | -1.567023 | -4.9099 | -1.45554 |
| hsa-miR-7977 | 9.89E-01 | 1.20E-01 | -1.555623 | -4.9268 | -1.444951 |
| hsa-miR-127-3p | 9.89E-01 | 1.32E-01 | 1.506714 | -4.9978 | 1.399522 |
| hsa-miR-7107-5p | 9.89E-01 | 1.42E-01 | 1.468498 | -5.0517 | 1.364025 |
| hsa-miR-183-5p | 9.89E-01 | 1.45E-01 | -1.457003 | -5.0677 | -1.353348 |
| hsa-miR-193a-5p | 9.89E-01 | 1.46E-01 | 1.452363 | -5.0741 | 1.349037 |
| hsa-miR-338-3p | 9.89E-01 | 1.51E-01 | -1.43688 | -5.0953 | -1.334656 |
| hsa-miR-487b-3p | 9.89E-01 | 1.51E-01 | 1.434566 | -5.0984 | 1.332506 |
| hsa-miR-4284 | 9.89E-01 | 1.54E-01 | -1.425776 | -5.1103 | -1.324342 |
| hsa-miR-30c-1-3p | 9.89E-01 | 1.56E-01 | -1.420184 | -5.1179 | -1.319148 |
| hsa-miR-154-5p | 9.89E-01 | 1.57E-01 | 1.416727 | -5.1225 | 1.315937 |
| hsa-miR-342-3p | 9.89E-01 | 1.60E-01 | 1.404351 | -5.1391 | 1.304441 |
| hsa-miR-30a-5p | 9.89E-01 | 1.68E-01 | 1.379375 | -5.172 | 1.281242 |
| hsa-miR-590-5p | 9.89E-01 | 1.68E-01 | -1.377413 | -5.1746 | -1.27942 |
| hsa-miR-5703 | 9.89E-01 | 1.71E-01 | 1.369774 | -5.1845 | 1.272324 |
| hsa-miR-6786-5p | 9.89E-01 | 1.75E-01 | 1.356546 | -5.2016 | 1.260037 |
| hsa-miR-501-3p | 9.89E-01 | 1.90E-01 | -1.31145 | -5.2587 | -1.21815 |
| hsa-miR-199a-3p | 9.89E-01 | 1.90E-01 | 1.310035 | -5.2604 | 1.216835 |
| hsa-miR-335-5p | 9.89E-01 | 1.90E-01 | -1.309522 | -5.2611 | -1.216359 |
| hsa-miR-93-5p | 9.89E-01 | 1.92E-01 | -1.30408 | -5.2678 | -1.211304 |
| hsa-miR-224-5p | 9.89E-01 | 1.95E-01 | -1.296412 | -5.2773 | -1.204181 |
| hsa-miR-454-3p | 9.89E-01 | 1.95E-01 | -1.295866 | -5.2779 | -1.203674 |
| hsa-miR-6090 | 9.89E-01 | 2.01E-01 | 1.279256 | -5.2982 | 1.188246 |
| hsa-miR-382-5p | 9.89E-01 | 2.04E-01 | 1.269191 | -5.3104 | 1.178897 |
| hsa-miR-29b-3p | 9.89E-01 | 2.06E-01 | -1.263971 | -5.3166 | -1.174048 |
| hsa-miR-374c-5p | 9.89E-01 | 2.08E-01 | -1.259148 | -5.3224 | -1.169569 |
| hsa-miR-142-5p | 9.89E-01 | 2.09E-01 | -1.255881 | -5.3263 | -1.166534 |
| hsa-miR-1244 | 9.89E-01 | 2.10E-01 | -1.253693 | -5.3289 | -1.164502 |
| hsa-miR-22-5p | 9.89E-01 | 2.16E-01 | 1.236459 | -5.3492 | 1.148493 |
| hsa-miR-6825-5p | 9.89E-01 | 2.16E-01 | 1.236285 | -5.3494 | 1.148332 |
| hsa-miR-1202 | 9.89E-01 | 2.16E-01 | 1.236285 | -5.3494 | 1.148332 |
| hsa-miR-532-5p | 9.89E-01 | 2.18E-01 | -1.232671 | -5.3537 | -1.144975 |
| hsa-miR-1207-5p | 9.89E-01 | 2.20E-01 | 1.226232 | -5.3612 | 1.138995 |
| hsa-miR-10b-5p | 9.89E-01 | 2.22E-01 | -1.221968 | -5.3661 | -1.135034 |
| hsa-miR-31-5p | 9.89E-01 | 2.24E-01 | -1.215441 | -5.3737 | -1.128971 |
| hsa-miR-6749-5p | 9.89E-01 | 2.25E-01 | 1.21391 | -5.3754 | 1.127549 |
| hsa-miR-630 | 9.89E-01 | 2.26E-01 | 1.209704 | -5.3803 | 1.123642 |
| hsa-miR-142-3p | 9.89E-01 | 2.30E-01 | -1.20106 | -5.3901 | -1.115613 |
| hsa-miR-29c-5p | 9.89E-01 | 2.35E-01 | 1.187797 | -5.4052 | 1.103293 |
| hsa-miR-551b-3p | 9.89E-01 | 2.35E-01 | 1.186769 | -5.4063 | 1.102339 |
| hsa-miR-4788 | 9.89E-01 | 2.44E-01 | -1.164915 | -5.4307 | -1.08204 |
| hsa-miR-335-3p | 9.89E-01 | 2.50E-01 | -1.150765 | -5.4462 | -1.068896 |
| hsa-miR-500a-5p | 9.89E-01 | 2.54E-01 | -1.139811 | -5.4581 | -1.058722 |
| hsa-miR-3652 | 9.89E-01 | 2.57E-01 | 1.133691 | -5.4647 | 1.053037 |
| hsa-miR-4763-3p | 9.89E-01 | 2.58E-01 | 1.132364 | -5.4661 | 1.051804 |
| hsa-miR-221-3p | 9.89E-01 | 2.61E-01 | -1.124284 | -5.4748 | -1.044299 |
| hsa-miR-4665-5p | 9.89E-01 | 2.61E-01 | -1.123611 | -5.4755 | -1.043674 |
| hsa-miR-6794-5p | 9.89E-01 | 2.66E-01 | 1.113542 | -5.4862 | 1.034322 |
| hsa-miR-660-5p | 9.89E-01 | 2.67E-01 | -1.109759 | -5.4902 | -1.030808 |
| hsa-miR-299-5p | 9.89E-01 | 2.70E-01 | 1.102905 | -5.4973 | 1.024441 |
| hsa-miR-376c-3p | 9.89E-01 | 2.71E-01 | 1.10135 | -5.499 | 1.022997 |
| hsa-miR-654-3p | 9.89E-01 | 2.71E-01 | 1.101139 | -5.4992 | 1.022801 |
| hsa-miR-92a-3p | 9.89E-01 | 2.77E-01 | -1.086875 | -5.514 | -1.009552 |
| hsa-miR-130b-3p | 9.89E-01 | 2.80E-01 | -1.081443 | -5.5196 | -1.004506 |
| hsa-miR-381-3p | 9.89E-01 | 2.82E-01 | 1.076669 | -5.5245 | 1.000072 |
| hsa-miR-6126 | 9.89E-01 | 2.84E-01 | -1.070562 | -5.5307 | -0.994399 |
| hsa-miR-3653-3p | 9.89E-01 | 2.86E-01 | 1.067775 | -5.5335 | 0.991811 |
| hsa-miR-34a-5p | 9.89E-01 | 2.86E-01 | 1.066343 | -5.5349 | 0.99048 |
| hsa-miR-22-3p | 9.89E-01 | 2.90E-01 | 1.05884 | -5.5425 | 0.983511 |
| hsa-miR-602 | 9.89E-01 | 2.96E-01 | -1.04471 | -5.5566 | -0.970387 |
| hsa-let-7c-5p | 9.89E-01 | 2.98E-01 | 1.040747 | -5.5605 | 0.966705 |
| hsa-miR-376a-3p | 9.89E-01 | 3.04E-01 | 1.028411 | -5.5726 | 0.955247 |
| hsa-miR-4270 | 9.89E-01 | 3.04E-01 | 1.026997 | -5.574 | 0.953933 |
| hsa-miR-4672 | 9.89E-01 | 3.06E-01 | -1.024726 | -5.5762 | -0.951824 |
| hsa-miR-5096 | 9.89E-01 | 3.13E-01 | 1.009831 | -5.5906 | 0.937989 |
| hsa-miR-4655-3p | 9.89E-01 | 3.14E-01 | 1.006728 | -5.5935 | 0.935106 |
| hsa-miR-1249-5p | 9.89E-01 | 3.16E-01 | -1.003809 | -5.5963 | -0.932395 |
| hsa-miR-15b-5p | 9.89E-01 | 3.16E-01 | -1.002092 | -5.5979 | -0.9308 |
| hsa-miR-4462 | 9.89E-01 | 3.20E-01 | 0.993922 | -5.6057 | 0.923212 |
| hsa-miR-3198 | 9.89E-01 | 3.23E-01 | 0.988524 | -5.6107 | 0.918198 |
| hsa-miR-455-5p | 9.89E-01 | 3.29E-01 | -0.976276 | -5.6222 | -0.906821 |
| hsa-miR-500a-3p | 9.89E-01 | 3.33E-01 | -0.967207 | -5.6305 | -0.898397 |
| hsa-miR-378a-5p | 9.89E-01 | 3.35E-01 | -0.964524 | -5.633 | -0.895905 |
| hsa-miR-1225-5p | 9.89E-01 | 3.36E-01 | 0.961522 | -5.6357 | 0.893116 |
| hsa-miR-337-5p | 9.89E-01 | 3.40E-01 | 0.953428 | -5.6431 | 0.885599 |
| hsa-miR-532-3p | 9.89E-01 | 3.42E-01 | -0.949558 | -5.6465 | -0.882003 |
| hsa-miR-1247-3p | 9.89E-01 | 3.44E-01 | -0.946457 | -5.6493 | -0.879123 |
| hsa-miR-409-3p | 9.89E-01 | 3.46E-01 | 0.943202 | -5.6522 | 0.8761 |
| hsa-miR-374b-5p | 9.89E-01 | 3.46E-01 | -0.941913 | -5.6534 | -0.874903 |
| hsa-miR-425-5p | 9.89E-01 | 3.46E-01 | -0.941674 | -5.6536 | -0.874681 |
| hsa-miR-4734 | 9.89E-01 | 3.47E-01 | -0.940517 | -5.6546 | -0.873606 |
| hsa-miR-5787 | 9.89E-01 | 3.50E-01 | 0.934223 | -5.6602 | 0.867759 |
| hsa-miR-4707-5p | 9.89E-01 | 3.51E-01 | 0.933051 | -5.6613 | 0.866671 |
| hsa-miR-140-3p | 9.89E-01 | 3.53E-01 | 0.929677 | -5.6643 | 0.863537 |
| hsa-miR-181a-5p | 9.89E-01 | 3.53E-01 | 0.928498 | -5.6653 | 0.862442 |
| hsa-miR-29b-1-5p | 9.89E-01 | 3.53E-01 | -0.928272 | -5.6655 | -0.862232 |
| hsa-miR-6763-5p | 9.89E-01 | 3.54E-01 | 0.927339 | -5.6663 | 0.861366 |
| hsa-miR-8071 | 9.89E-01 | 3.56E-01 | -0.923542 | -5.6696 | -0.857838 |
| hsa-miR-30e-5p | 9.89E-01 | 3.65E-01 | -0.906523 | -5.6844 | -0.84203 |
| hsa-miR-133b | 9.89E-01 | 3.66E-01 | -0.904445 | -5.6862 | -0.8401 |
| hsa-miR-6785-5p | 9.89E-01 | 3.66E-01 | 0.904225 | -5.6864 | 0.839896 |
| hsa-miR-6085 | 9.89E-01 | 3.67E-01 | 0.901327 | -5.6889 | 0.837204 |
| hsa-miR-126-5p | 9.89E-01 | 3.68E-01 | -0.901068 | -5.6891 | -0.836963 |
| hsa-miR-25-3p | 9.89E-01 | 3.69E-01 | -0.899338 | -5.6906 | -0.835357 |
| hsa-miR-6862-5p | 9.89E-01 | 3.69E-01 | -0.89752 | -5.6921 | -0.833668 |
| hsa-miR-34b-5p | 9.89E-01 | 3.76E-01 | 0.885025 | -5.7027 | 0.822061 |
| hsa-miR-6790-5p | 9.89E-01 | 3.80E-01 | 0.87866 | -5.708 | 0.816149 |
| hsa-miR-4741 | 9.89E-01 | 3.80E-01 | 0.877583 | -5.7089 | 0.815149 |
| hsa-miR-210-3p | 9.89E-01 | 3.88E-01 | -0.863356 | -5.7206 | -0.801935 |
| hsa-miR-6727-5p | 9.89E-01 | 3.93E-01 | 0.854739 | -5.7276 | 0.79393 |
| hsa-miR-648 | 9.89E-01 | 3.93E-01 | 0.854386 | -5.7279 | 0.793602 |
| hsa-miR-762 | 9.89E-01 | 3.94E-01 | 0.852821 | -5.7292 | 0.792148 |
| hsa-miR-101-3p | 9.89E-01 | 3.95E-01 | -0.850941 | -5.7307 | -0.790402 |
| hsa-miR-30b-5p | 9.89E-01 | 3.95E-01 | -0.849961 | -5.7315 | -0.789492 |
| hsa-miR-3682-3p | 9.89E-01 | 3.98E-01 | 0.845852 | -5.7348 | 0.785676 |
| hsa-miR-21-3p | 9.89E-01 | 3.99E-01 | -0.84312 | -5.737 | -0.783138 |
| hsa-miR-6516-5p | 9.89E-01 | 4.00E-01 | -0.842172 | -5.7377 | -0.782258 |
| hsa-miR-3156-5p | 9.89E-01 | 4.02E-01 | -0.83866 | -5.7405 | -0.778996 |
| hsa-miR-3646 | 9.89E-01 | 4.02E-01 | -0.837871 | -5.7412 | -0.778262 |
| hsa-miR-146b-5p | 9.89E-01 | 4.09E-01 | 0.825652 | -5.7508 | 0.766912 |
| hsa-miR-151a-3p | 9.89E-01 | 4.14E-01 | -0.816775 | -5.7577 | -0.758667 |
| hsa-miR-6769b-5p | 9.89E-01 | 4.16E-01 | 0.81374 | -5.7601 | 0.755848 |
| hsa-miR-451a | 9.89E-01 | 4.16E-01 | -0.812962 | -5.7607 | -0.755126 |
| hsa-miR-6792-5p | 9.89E-01 | 4.16E-01 | -0.812682 | -5.7609 | -0.754866 |
| hsa-miR-4697-5p | 9.89E-01 | 4.18E-01 | -0.809672 | -5.7632 | -0.752069 |
| hsa-miR-3614-5p | 9.89E-01 | 4.20E-01 | -0.807233 | -5.7651 | -0.749804 |
| hsa-miR-1260a | 9.89E-01 | 4.20E-01 | -0.80705 | -5.7652 | -0.749634 |
| hsa-miR-378d | 9.89E-01 | 4.24E-01 | -0.799721 | -5.7708 | -0.742826 |
| hsa-miR-3196 | 9.89E-01 | 4.26E-01 | 0.795868 | -5.7737 | 0.739248 |
| hsa-miR-663b | 9.89E-01 | 4.27E-01 | -0.794851 | -5.7745 | -0.738303 |
| hsa-miR-4286 | 9.89E-01 | 4.27E-01 | -0.794408 | -5.7748 | -0.737892 |
| hsa-miR-191-3p | 9.89E-01 | 4.27E-01 | 0.794139 | -5.775 | 0.737642 |
| hsa-miR-6753-5p | 9.89E-01 | 4.27E-01 | -0.793781 | -5.7753 | -0.737309 |
| hsa-miR-4530 | 9.89E-01 | 4.29E-01 | 0.791114 | -5.7773 | 0.734832 |
| hsa-miR-3651 | 9.89E-01 | 4.29E-01 | -0.790739 | -5.7776 | -0.734483 |
| hsa-miR-769-5p | 9.89E-01 | 4.30E-01 | -0.788989 | -5.7789 | -0.732858 |
| hsa-miR-6860 | 9.89E-01 | 4.31E-01 | 0.78712 | -5.7803 | 0.731122 |
| hsa-miR-4731-3p | 9.89E-01 | 4.32E-01 | 0.785283 | -5.7816 | 0.729416 |
| hsa-miR-146a-5p | 9.89E-01 | 4.36E-01 | -0.778721 | -5.7865 | -0.72332 |
| hsa-miR-4465 | 9.89E-01 | 4.38E-01 | -0.775539 | -5.7888 | -0.720365 |
| hsa-miR-7975 | 9.89E-01 | 4.41E-01 | -0.769846 | -5.793 | -0.715077 |
| hsa-miR-718 | 9.89E-01 | 4.49E-01 | 0.757464 | -5.802 | 0.703576 |
| hsa-let-7b-5p | 9.89E-01 | 4.49E-01 | 0.757387 | -5.802 | 0.703505 |
| hsa-miR-939-5p | 9.89E-01 | 4.51E-01 | 0.753987 | -5.8045 | 0.700346 |
| hsa-miR-15a-5p | 9.89E-01 | 4.60E-01 | -0.738913 | -5.8151 | -0.686345 |
| hsa-miR-6857-5p | 9.89E-01 | 4.64E-01 | 0.732208 | -5.8198 | 0.680117 |
| hsa-miR-340-3p | 9.89E-01 | 4.66E-01 | 0.729853 | -5.8214 | 0.677929 |
| hsa-miR-6807-5p | 9.89E-01 | 4.66E-01 | 0.728948 | -5.8221 | 0.677088 |
| hsa-miR-155-5p | 9.89E-01 | 4.68E-01 | 0.726397 | -5.8238 | 0.674719 |
| hsa-miR-150-5p | 9.89E-01 | 4.77E-01 | 0.711704 | -5.8338 | 0.661071 |
| hsa-miR-222-3p | 9.89E-01 | 4.87E-01 | -0.69483 | -5.8451 | -0.645398 |
| hsa-miR-1288-3p | 9.89E-01 | 4.89E-01 | -0.692659 | -5.8465 | -0.643381 |
| hsa-miR-1229-5p | 9.89E-01 | 4.90E-01 | 0.689817 | -5.8484 | 0.640741 |
| hsa-miR-6779-5p | 9.89E-01 | 4.91E-01 | 0.689484 | -5.8486 | 0.640432 |
| hsa-miR-6133 | 9.89E-01 | 4.94E-01 | 0.684575 | -5.8518 | 0.635872 |
| hsa-miR-130a-3p | 9.89E-01 | 4.97E-01 | 0.679874 | -5.8548 | 0.631506 |
| hsa-miR-3161 | 9.89E-01 | 5.03E-01 | 0.669732 | -5.8613 | 0.622085 |
| hsa-miR-3907 | 9.89E-01 | 5.08E-01 | -0.661475 | -5.8665 | -0.614415 |
| hsa-miR-4261 | 9.89E-01 | 5.10E-01 | -0.659338 | -5.8679 | -0.612431 |
| hsa-miR-6800-3p | 9.89E-01 | 5.10E-01 | -0.659216 | -5.868 | -0.612317 |
| hsa-miR-4534 | 9.89E-01 | 5.11E-01 | 0.656658 | -5.8696 | 0.609941 |
| hsa-miR-378i | 9.89E-01 | 5.19E-01 | -0.644752 | -5.8769 | -0.598882 |
| hsa-miR-128-3p | 9.89E-01 | 5.20E-01 | -0.643311 | -5.8778 | -0.597544 |
| hsa-miR-6088 | 9.89E-01 | 5.21E-01 | 0.642184 | -5.8785 | 0.596497 |
| hsa-miR-3945 | 9.89E-01 | 5.21E-01 | 0.641207 | -5.8791 | 0.59559 |
| hsa-miR-4429 | 9.89E-01 | 5.23E-01 | 0.639432 | -5.8801 | 0.593941 |
| hsa-miR-16-5p | 9.89E-01 | 5.27E-01 | -0.631937 | -5.8847 | -0.58698 |
| hsa-miR-3935 | 9.89E-01 | 5.29E-01 | -0.629956 | -5.8858 | -0.585139 |
| hsa-miR-185-5p | 9.89E-01 | 5.31E-01 | 0.62719 | -5.8875 | 0.58257 |
| hsa-miR-6127 | 9.89E-01 | 5.33E-01 | -0.623576 | -5.8896 | -0.579213 |
| hsa-miR-4306 | 9.89E-01 | 5.37E-01 | 0.617505 | -5.8932 | 0.573574 |
| hsa-miR-892b | 9.89E-01 | 5.37E-01 | 0.616662 | -5.8937 | 0.572791 |
| hsa-miR-502-3p | 9.89E-01 | 5.38E-01 | -0.61531 | -5.8945 | -0.571535 |
| hsa-miR-4313 | 9.89E-01 | 5.42E-01 | 0.610345 | -5.8974 | 0.566923 |
| hsa-miR-642a-3p | 9.89E-01 | 5.45E-01 | 0.605199 | -5.9003 | 0.562143 |
| hsa-miR-484 | 9.89E-01 | 5.45E-01 | -0.605139 | -5.9004 | -0.562088 |
| hsa-miR-3934-5p | 9.89E-01 | 5.45E-01 | -0.60456 | -5.9007 | -0.56155 |
| hsa-miR-145-5p | 9.89E-01 | 5.46E-01 | 0.603477 | -5.9013 | 0.560544 |
| hsa-miR-6766-3p | 9.89E-01 | 5.50E-01 | 0.597214 | -5.9049 | 0.554727 |
| hsa-miR-6165 | 9.89E-01 | 5.51E-01 | 0.596439 | -5.9053 | 0.554006 |
| hsa-miR-152-3p | 9.89E-01 | 5.54E-01 | 0.591648 | -5.908 | 0.549556 |
| hsa-miR-4433b-3p | 9.89E-01 | 5.55E-01 | 0.590655 | -5.9086 | 0.548634 |
| hsa-miR-151b | 9.89E-01 | 5.56E-01 | -0.589364 | -5.9093 | -0.547435 |
| hsa-miR-1238-5p | 9.89E-01 | 5.57E-01 | -0.587964 | -5.9101 | -0.546135 |
| hsa-miR-28-5p | 9.89E-01 | 5.59E-01 | -0.583912 | -5.9123 | -0.542371 |
| hsa-miR-378a-3p | 9.89E-01 | 5.60E-01 | -0.582274 | -5.9132 | -0.540849 |
| hsa-miR-99b-5p | 9.89E-01 | 5.65E-01 | 0.575767 | -5.9168 | 0.534805 |
| hsa-miR-149-3p | 9.89E-01 | 5.66E-01 | 0.573775 | -5.9179 | 0.532955 |
| hsa-miR-4459 | 9.89E-01 | 5.66E-01 | 0.573356 | -5.9181 | 0.532565 |
| hsa-miR-1249-3p | 9.89E-01 | 5.67E-01 | 0.571928 | -5.9189 | 0.531239 |
| hsa-miR-7150 | 9.89E-01 | 5.67E-01 | 0.571826 | -5.919 | 0.531145 |
| hsa-miR-1268b | 9.89E-01 | 5.69E-01 | 0.570215 | -5.9198 | 0.529648 |
| hsa-miR-4753-5p | 9.89E-01 | 5.69E-01 | 0.569486 | -5.9202 | 0.528971 |
| hsa-miR-6826-5p | 9.89E-01 | 5.69E-01 | -0.568966 | -5.9205 | -0.528488 |
| hsa-miR-4281 | 9.89E-01 | 5.70E-01 | 0.567754 | -5.9212 | 0.527363 |
| hsa-miR-181b-5p | 9.89E-01 | 5.71E-01 | 0.566568 | -5.9218 | 0.526261 |
| hsa-miR-6132 | 9.89E-01 | 5.71E-01 | 0.56623 | -5.922 | 0.525947 |
| hsa-miR-4532 | 9.89E-01 | 5.72E-01 | -0.565558 | -5.9223 | -0.525322 |
| hsa-miR-23a-3p | 9.89E-01 | 5.72E-01 | 0.564854 | -5.9227 | 0.524668 |
| hsa-miR-1304-3p | 9.89E-01 | 5.78E-01 | 0.556843 | -5.927 | 0.517228 |
| hsa-miR-6867-5p | 9.89E-01 | 5.78E-01 | 0.556077 | -5.9274 | 0.516516 |
| hsa-miR-7114-5p | 9.89E-01 | 5.78E-01 | 0.55603 | -5.9274 | 0.516473 |
| hsa-miR-499a-5p | 9.89E-01 | 5.82E-01 | 0.550696 | -5.9302 | 0.511518 |
| hsa-miR-188-5p | 9.89E-01 | 5.85E-01 | 0.545719 | -5.9328 | 0.506895 |
| hsa-miR-6809-5p | 9.89E-01 | 5.87E-01 | 0.542903 | -5.9342 | 0.50428 |
| hsa-miR-6124 | 9.89E-01 | 5.89E-01 | 0.540861 | -5.9353 | 0.502383 |
| hsa-miR-9500 | 9.89E-01 | 5.90E-01 | 0.538479 | -5.9365 | 0.50017 |
| hsa-miR-4322 | 9.89E-01 | 5.92E-01 | -0.535997 | -5.9378 | -0.497864 |
| hsa-miR-6732-5p | 9.89E-01 | 5.96E-01 | -0.530113 | -5.9407 | -0.4924 |
| hsa-miR-1471 | 9.89E-01 | 5.97E-01 | 0.528777 | -5.9414 | 0.491159 |
| hsa-miR-619-5p | 9.89E-01 | 6.00E-01 | -0.52454 | -5.9435 | -0.487223 |
| hsa-miR-652-3p | 9.89E-01 | 6.00E-01 | -0.524162 | -5.9437 | -0.486872 |
| hsa-miR-6728-5p | 9.89E-01 | 6.02E-01 | 0.521073 | -5.9452 | 0.484002 |
| hsa-miR-151a-5p | 9.89E-01 | 6.07E-01 | -0.514917 | -5.9483 | -0.478285 |
| hsa-miR-6850-5p | 9.89E-01 | 6.07E-01 | 0.51468 | -5.9484 | 0.478064 |
| hsa-miR-4441 | 9.89E-01 | 6.09E-01 | 0.511879 | -5.9498 | 0.475462 |
| hsa-miR-98-5p | 9.89E-01 | 6.10E-01 | -0.510132 | -5.9506 | -0.47384 |
| hsa-miR-6831-5p | 9.89E-01 | 6.12E-01 | 0.507131 | -5.952 | 0.471053 |
| hsa-miR-5100 | 9.89E-01 | 6.12E-01 | -0.506799 | -5.9522 | -0.470744 |
| hsa-miR-26b-5p | 9.89E-01 | 6.14E-01 | -0.50389 | -5.9536 | -0.468042 |
| hsa-miR-4721 | 9.89E-01 | 6.14E-01 | 0.50377 | -5.9537 | 0.467931 |
| hsa-miR-6768-5p | 9.89E-01 | 6.16E-01 | 0.501309 | -5.9548 | 0.465644 |
| hsa-miR-4701-3p | 9.89E-01 | 6.16E-01 | 0.50126 | -5.9549 | 0.465599 |
| hsa-miR-33b-3p | 9.89E-01 | 6.20E-01 | -0.496567 | -5.9571 | -0.461239 |
| hsa-miR-3656 | 9.89E-01 | 6.22E-01 | 0.493343 | -5.9586 | 0.458245 |
| hsa-miR-1291 | 9.89E-01 | 6.24E-01 | -0.490877 | -5.9597 | -0.455954 |
| hsa-miR-6789-5p | 9.89E-01 | 6.26E-01 | 0.486862 | -5.9616 | 0.452225 |
| hsa-miR-1228-3p | 9.89E-01 | 6.29E-01 | 0.482605 | -5.9636 | 0.448271 |
| hsa-miR-514b-5p | 9.89E-01 | 6.30E-01 | 0.482132 | -5.9638 | 0.447832 |
| hsa-miR-140-5p | 9.89E-01 | 6.33E-01 | 0.477812 | -5.9657 | 0.44382 |
| hsa-miR-4449 | 9.89E-01 | 6.36E-01 | -0.472977 | -5.9679 | -0.439328 |
| hsa-miR-940 | 9.89E-01 | 6.38E-01 | -0.470977 | -5.9688 | -0.43747 |
| hsa-miR-4481 | 9.89E-01 | 6.39E-01 | 0.468498 | -5.9699 | 0.435168 |
| hsa-miR-186-5p | 9.89E-01 | 6.41E-01 | -0.466598 | -5.9708 | -0.433403 |
| hsa-miR-598-3p | 9.89E-01 | 6.41E-01 | -0.465681 | -5.9712 | -0.432551 |
| hsa-miR-6515-3p | 9.89E-01 | 6.41E-01 | 0.465631 | -5.9712 | 0.432505 |
| hsa-miR-7110-5p | 9.89E-01 | 6.42E-01 | 0.465219 | -5.9714 | 0.432122 |
| hsa-miR-4327 | 9.89E-01 | 6.43E-01 | -0.464062 | -5.9719 | -0.431048 |
| hsa-miR-623 | 9.89E-01 | 6.43E-01 | 0.463877 | -5.972 | 0.430875 |
| hsa-miR-1587 | 9.89E-01 | 6.43E-01 | 0.463656 | -5.9721 | 0.43067 |
| hsa-miR-4515 | 9.89E-01 | 6.43E-01 | -0.462916 | -5.9724 | -0.429983 |
| hsa-miR-4514 | 9.89E-01 | 6.45E-01 | -0.460792 | -5.9733 | -0.42801 |
| hsa-miR-134-5p | 9.89E-01 | 6.46E-01 | 0.459021 | -5.9741 | 0.426365 |
| hsa-miR-6787-5p | 9.89E-01 | 6.47E-01 | -0.458018 | -5.9745 | -0.425434 |
| hsa-miR-6724-5p | 9.89E-01 | 6.49E-01 | 0.454938 | -5.9758 | 0.422573 |
| hsa-miR-877-5p | 9.89E-01 | 6.50E-01 | 0.454229 | -5.9762 | 0.421914 |
| hsa-miR-4417 | 9.89E-01 | 6.51E-01 | 0.452459 | -5.9769 | 0.42027 |
| hsa-let-7e-5p | 9.89E-01 | 6.52E-01 | 0.450381 | -5.9778 | 0.418339 |
| hsa-miR-6073 | 9.89E-01 | 6.53E-01 | 0.450274 | -5.9778 | 0.41824 |
| hsa-miR-6865-5p | 9.89E-01 | 6.53E-01 | 0.449749 | -5.9781 | 0.417752 |
| hsa-miR-1469 | 9.89E-01 | 6.53E-01 | -0.449634 | -5.9781 | -0.417646 |
| hsa-miR-3679-5p | 9.89E-01 | 6.55E-01 | 0.446942 | -5.9793 | 0.415145 |
| hsa-miR-6848-5p | 9.89E-01 | 6.59E-01 | 0.442005 | -5.9813 | 0.41056 |
| hsa-miR-6893-5p | 9.89E-01 | 6.60E-01 | 0.439775 | -5.9823 | 0.408488 |
| hsa-miR-483-5p | 9.89E-01 | 6.61E-01 | 0.438361 | -5.9829 | 0.407175 |
| hsa-miR-7162-3p | 9.89E-01 | 6.63E-01 | -0.435966 | -5.9839 | -0.40495 |
| hsa-miR-4468 | 9.89E-01 | 6.64E-01 | 0.434949 | -5.9843 | 0.404005 |
| hsa-miR-28-3p | 9.89E-01 | 6.65E-01 | -0.433402 | -5.9849 | -0.402569 |
| hsa-miR-494-3p | 9.89E-01 | 6.65E-01 | 0.43288 | -5.9851 | 0.402083 |
| hsa-miR-5195-3p | 9.89E-01 | 6.67E-01 | 0.430143 | -5.9862 | 0.399541 |
| hsa-miR-6745 | 9.89E-01 | 6.69E-01 | -0.427332 | -5.9874 | -0.39693 |
| hsa-miR-6840-3p | 9.89E-01 | 6.71E-01 | 0.424924 | -5.9884 | 0.394694 |
| hsa-miR-223-3p | 9.89E-01 | 6.72E-01 | -0.423289 | -5.989 | -0.393175 |
| hsa-miR-4507 | 9.89E-01 | 6.74E-01 | 0.421153 | -5.9899 | 0.391191 |
| hsa-miR-5739 | 9.89E-01 | 6.74E-01 | 0.420784 | -5.99 | 0.390848 |
| hsa-miR-3667-5p | 9.89E-01 | 6.74E-01 | 0.420294 | -5.9902 | 0.390393 |
| hsa-miR-6821-5p | 9.89E-01 | 6.75E-01 | 0.419871 | -5.9904 | 0.39 |
| hsa-miR-6086 | 9.89E-01 | 6.75E-01 | 0.419084 | -5.9907 | 0.389269 |
| hsa-miR-6129 | 9.89E-01 | 6.77E-01 | 0.416319 | -5.9918 | 0.386701 |
| hsa-miR-3679-3p | 9.89E-01 | 6.82E-01 | 0.40997 | -5.9943 | 0.380803 |
| hsa-miR-6856-5p | 9.89E-01 | 6.83E-01 | 0.408937 | -5.9947 | 0.379844 |
| hsa-miR-503-5p | 9.89E-01 | 6.84E-01 | -0.407379 | -5.9953 | -0.378397 |
| hsa-miR-4497 | 9.89E-01 | 6.87E-01 | -0.403129 | -5.9969 | -0.374449 |
| hsa-miR-3960 | 9.89E-01 | 6.89E-01 | 0.400225 | -5.998 | 0.371752 |
| hsa-miR-4632-5p | 9.89E-01 | 6.93E-01 | 0.394694 | -6.0001 | 0.366614 |
| hsa-miR-7109-5p | 9.89E-01 | 6.94E-01 | -0.393942 | -6.0004 | -0.365916 |
| hsa-miR-1275 | 9.89E-01 | 6.94E-01 | 0.393296 | -6.0006 | 0.365316 |
| hsa-miR-642b-3p | 9.89E-01 | 6.94E-01 | -0.393209 | -6.0007 | -0.365235 |
| hsa-miR-6752-5p | 9.89E-01 | 6.97E-01 | 0.38949 | -6.002 | 0.36178 |
| hsa-miR-3158-5p | 9.89E-01 | 6.97E-01 | 0.389267 | -6.0021 | 0.361573 |
| hsa-miR-6758-5p | 9.89E-01 | 6.98E-01 | 0.387839 | -6.0027 | 0.360247 |
| hsa-miR-548q | 9.89E-01 | 6.99E-01 | 0.387029 | -6.003 | 0.359495 |
| hsa-miR-6729-5p | 9.89E-01 | 7.02E-01 | -0.383312 | -6.0043 | -0.356042 |
| hsa-miR-665 | 9.89E-01 | 7.06E-01 | 0.377422 | -6.0064 | 0.350571 |
| hsa-miR-6760-5p | 9.89E-01 | 7.09E-01 | 0.372573 | -6.0082 | 0.346067 |
| hsa-miR-1268a | 9.89E-01 | 7.10E-01 | -0.371395 | -6.0086 | -0.344973 |
| hsa-miR-377-3p | 9.89E-01 | 7.11E-01 | -0.370825 | -6.0088 | -0.344443 |
| hsa-miR-4743-5p | 9.89E-01 | 7.11E-01 | 0.370103 | -6.009 | 0.343772 |
| hsa-miR-4317 | 9.89E-01 | 7.12E-01 | 0.369847 | -6.0091 | 0.343535 |
| hsa-miR-4728-5p | 9.89E-01 | 7.12E-01 | 0.368758 | -6.0095 | 0.342524 |
| hsa-miR-574-3p | 9.89E-01 | 7.15E-01 | 0.365047 | -6.0108 | 0.339076 |
| hsa-miR-371b-5p | 9.89E-01 | 7.18E-01 | -0.360705 | -6.0123 | -0.335043 |
| hsa-miR-4433a-5p | 9.89E-01 | 7.19E-01 | 0.360066 | -6.0125 | 0.33445 |
| hsa-miR-361-3p | 9.89E-01 | 7.19E-01 | 0.359842 | -6.0126 | 0.334242 |
| hsa-miR-135a-3p | 9.89E-01 | 7.19E-01 | 0.359792 | -6.0126 | 0.334195 |
| hsa-miR-8485 | 9.89E-01 | 7.23E-01 | 0.35457 | -6.0144 | 0.329345 |
| hsa-miR-145-3p | 9.89E-01 | 7.23E-01 | 0.35406 | -6.0145 | 0.328871 |
| hsa-miR-6797-5p | 9.89E-01 | 7.24E-01 | -0.352647 | -6.015 | -0.327558 |
| hsa-miR-601 | 9.89E-01 | 7.24E-01 | 0.352625 | -6.015 | 0.327538 |
| hsa-miR-3620-5p | 9.89E-01 | 7.25E-01 | 0.351866 | -6.0153 | 0.326833 |
| hsa-miR-3648 | 9.89E-01 | 7.27E-01 | -0.34977 | -6.016 | -0.324886 |
| hsa-miR-29a-3p | 9.89E-01 | 7.27E-01 | -0.349554 | -6.016 | -0.324686 |
| hsa-miR-212-3p | 9.89E-01 | 7.27E-01 | -0.349259 | -6.0161 | -0.324412 |
| hsa-let-7g-5p | 9.89E-01 | 7.28E-01 | -0.347165 | -6.0168 | -0.322467 |
| hsa-miR-1260b | 9.89E-01 | 7.29E-01 | 0.347002 | -6.0169 | 0.322315 |
| hsa-miR-6722-3p | 9.89E-01 | 7.29E-01 | 0.346833 | -6.0169 | 0.322159 |
| hsa-miR-495-3p | 9.89E-01 | 7.29E-01 | 0.346833 | -6.0169 | 0.322158 |
| hsa-let-7i-5p | 9.89E-01 | 7.29E-01 | 0.346067 | -6.0172 | 0.321447 |
| hsa-miR-4647 | 9.89E-01 | 7.31E-01 | -0.344307 | -6.0178 | -0.319812 |
| hsa-miR-8072 | 9.89E-01 | 7.31E-01 | 0.343648 | -6.018 | 0.3192 |
| hsa-miR-6778-5p | 9.89E-01 | 7.33E-01 | 0.341672 | -6.0186 | 0.317364 |
| hsa-miR-6851-5p | 9.89E-01 | 7.33E-01 | 0.341387 | -6.0187 | 0.3171 |
| hsa-miR-4787-5p | 9.89E-01 | 7.35E-01 | -0.338216 | -6.0197 | -0.314154 |
| hsa-miR-6812-5p | 9.89E-01 | 7.36E-01 | 0.336896 | -6.0202 | 0.312929 |
| hsa-miR-638 | 9.89E-01 | 7.39E-01 | 0.332538 | -6.0215 | 0.308881 |
| hsa-miR-874-3p | 9.89E-01 | 7.40E-01 | 0.33128 | -6.0219 | 0.307712 |
| hsa-miR-30c-2-3p | 9.89E-01 | 7.43E-01 | 0.328141 | -6.0229 | 0.304797 |
| hsa-miR-8085 | 9.89E-01 | 7.44E-01 | 0.326634 | -6.0234 | 0.303396 |
| hsa-miR-187-5p | 9.89E-01 | 7.45E-01 | 0.325536 | -6.0237 | 0.302377 |
| hsa-miR-3605-5p | 9.89E-01 | 7.45E-01 | -0.325287 | -6.0238 | -0.302146 |
| hsa-miR-5008-5p | 9.89E-01 | 7.45E-01 | 0.324695 | -6.024 | 0.301595 |
| hsa-miR-211-3p | 9.89E-01 | 7.48E-01 | 0.321799 | -6.0249 | 0.298906 |
| hsa-miR-4443 | 9.89E-01 | 7.49E-01 | 0.320101 | -6.0254 | 0.297328 |
| hsa-miR-1234-3p | 9.89E-01 | 7.49E-01 | 0.31968 | -6.0255 | 0.296937 |
| hsa-miR-765 | 9.89E-01 | 7.51E-01 | 0.316681 | -6.0264 | 0.294151 |
| hsa-miR-6752-3p | 9.89E-01 | 7.52E-01 | 0.315416 | -6.0268 | 0.292976 |
| hsa-miR-6802-5p | 9.89E-01 | 7.55E-01 | 0.312385 | -6.0277 | 0.290161 |
| hsa-miR-4539 | 9.89E-01 | 7.55E-01 | 0.311591 | -6.0279 | 0.289424 |
| hsa-miR-937-5p | 9.89E-01 | 7.55E-01 | -0.31157 | -6.0279 | -0.289404 |
| hsa-miR-365a-5p | 9.89E-01 | 7.56E-01 | 0.31054 | -6.0282 | 0.288448 |
| hsa-miR-6775-5p | 9.89E-01 | 7.57E-01 | 0.310076 | -6.0284 | 0.288016 |
| hsa-miR-3194-5p | 9.89E-01 | 7.58E-01 | 0.308284 | -6.0289 | 0.286352 |
| hsa-miR-6846-5p | 9.89E-01 | 7.60E-01 | 0.305342 | -6.0298 | 0.283619 |
| hsa-miR-1224-5p | 9.89E-01 | 7.60E-01 | -0.305246 | -6.0298 | -0.28353 |
| hsa-miR-4713-3p | 9.89E-01 | 7.62E-01 | -0.3031 | -6.0304 | -0.281536 |
| hsa-miR-664b-5p | 9.89E-01 | 7.63E-01 | -0.301699 | -6.0308 | -0.280236 |
| hsa-miR-320e | 9.89E-01 | 7.64E-01 | -0.299957 | -6.0313 | -0.278618 |
| hsa-miR-1185-2-3p | 9.89E-01 | 7.69E-01 | -0.293347 | -6.0332 | -0.272477 |
| hsa-miR-1182 | 9.89E-01 | 7.69E-01 | 0.293229 | -6.0332 | 0.272368 |
| hsa-miR-26a-5p | 9.89E-01 | 7.70E-01 | 0.292758 | -6.0333 | 0.27193 |
| hsa-miR-320d | 9.89E-01 | 7.70E-01 | -0.2926 | -6.0334 | -0.271784 |
| hsa-miR-6801-5p | 9.89E-01 | 7.70E-01 | 0.292329 | -6.0334 | 0.271532 |
| hsa-miR-4298 | 9.89E-01 | 7.72E-01 | 0.290089 | -6.0341 | 0.269451 |
| hsa-miR-4535 | 9.89E-01 | 7.72E-01 | -0.289869 | -6.0341 | -0.269247 |
| hsa-miR-1247-5p | 9.89E-01 | 7.74E-01 | -0.287003 | -6.0349 | -0.266585 |
| hsa-miR-6849-5p | 9.89E-01 | 7.75E-01 | -0.2855 | -6.0353 | -0.265188 |
| hsa-miR-143-5p | 9.89E-01 | 7.76E-01 | 0.284575 | -6.0356 | 0.264329 |
| hsa-miR-6746-5p | 9.89E-01 | 7.81E-01 | 0.277972 | -6.0373 | 0.258197 |
| hsa-miR-143-3p | 9.89E-01 | 7.85E-01 | -0.272904 | -6.0387 | -0.253489 |
| hsa-miR-6781-5p | 9.89E-01 | 7.85E-01 | -0.272873 | -6.0387 | -0.25346 |
| hsa-miR-5684 | 9.89E-01 | 7.86E-01 | 0.272045 | -6.0389 | 0.252691 |
| hsa-miR-1273e | 9.89E-01 | 7.88E-01 | 0.269339 | -6.0396 | 0.250177 |
| hsa-miR-125a-5p | 9.89E-01 | 7.89E-01 | 0.268017 | -6.0399 | 0.24895 |
| hsa-miR-3138 | 9.89E-01 | 7.89E-01 | 0.267323 | -6.0401 | 0.248305 |
| hsa-miR-6800-5p | 9.89E-01 | 7.90E-01 | 0.266355 | -6.0403 | 0.247406 |
| hsa-miR-378f | 9.89E-01 | 7.92E-01 | -0.26421 | -6.0409 | -0.245413 |
| hsa-miR-4800-5p | 9.89E-01 | 7.93E-01 | -0.26229 | -6.0413 | -0.24363 |
| hsa-miR-2467-3p | 9.89E-01 | 7.96E-01 | 0.258171 | -6.0424 | 0.239804 |
| hsa-miR-3162-5p | 9.89E-01 | 7.97E-01 | 0.257083 | -6.0426 | 0.238793 |
| hsa-miR-3610 | 9.89E-01 | 7.98E-01 | -0.25618 | -6.0428 | -0.237954 |
| hsa-miR-6747-5p | 9.89E-01 | 7.98E-01 | 0.255959 | -6.0429 | 0.237749 |
| hsa-miR-6798-5p | 9.89E-01 | 7.99E-01 | 0.254916 | -6.0432 | 0.236781 |
| hsa-miR-4463 | 9.89E-01 | 7.99E-01 | 0.254748 | -6.0432 | 0.236624 |
| hsa-miR-365a-3p | 9.89E-01 | 8.00E-01 | 0.253936 | -6.0434 | 0.23587 |
| hsa-miR-4778-5p | 9.89E-01 | 8.01E-01 | 0.252267 | -6.0438 | 0.23432 |
| hsa-miR-504-3p | 9.89E-01 | 8.02E-01 | 0.25018 | -6.0443 | 0.232382 |
| hsa-miR-4496 | 9.89E-01 | 8.03E-01 | 0.248988 | -6.0446 | 0.231274 |
| hsa-miR-550a-5p | 9.89E-01 | 8.04E-01 | -0.248299 | -6.0447 | -0.230634 |
| hsa-miR-486-5p | 9.89E-01 | 8.04E-01 | -0.248051 | -6.0448 | -0.230403 |
| hsa-miR-6512-5p | 9.89E-01 | 8.05E-01 | -0.247381 | -6.0449 | -0.229781 |
| hsa-miR-6819-5p | 9.89E-01 | 8.07E-01 | -0.243961 | -6.0457 | -0.226605 |
| hsa-miR-5580-3p | 9.89E-01 | 8.08E-01 | -0.243208 | -6.0459 | -0.225906 |
| hsa-miR-4640-5p | 9.89E-01 | 8.09E-01 | -0.241176 | -6.0464 | -0.224018 |
| hsa-miR-7641 | 9.89E-01 | 8.11E-01 | -0.239745 | -6.0467 | -0.222689 |
| hsa-miR-6880-5p | 9.89E-01 | 8.11E-01 | 0.239714 | -6.0467 | 0.22266 |
| hsa-miR-4505 | 9.89E-01 | 8.12E-01 | 0.237338 | -6.0473 | 0.220453 |
| hsa-miR-566 | 9.89E-01 | 8.13E-01 | 0.237145 | -6.0473 | 0.220274 |
| hsa-miR-1972 | 9.89E-01 | 8.13E-01 | 0.237074 | -6.0473 | 0.220208 |
| hsa-miR-4651 | 9.89E-01 | 8.14E-01 | 0.235828 | -6.0476 | 0.219051 |
| hsa-miR-550a-3-5p | 9.89E-01 | 8.15E-01 | -0.233542 | -6.0481 | -0.216927 |
| hsa-miR-664a-5p | 9.89E-01 | 8.16E-01 | -0.232185 | -6.0484 | -0.215666 |
| hsa-miR-5088-5p | 9.89E-01 | 8.19E-01 | -0.229352 | -6.049 | -0.213035 |
| hsa-miR-4749-5p | 9.89E-01 | 8.19E-01 | -0.228864 | -6.0491 | -0.212582 |
| hsa-miR-6891-5p | 9.89E-01 | 8.21E-01 | 0.226526 | -6.0496 | 0.210411 |
| hsa-miR-6131 | 9.89E-01 | 8.23E-01 | -0.223679 | -6.0502 | -0.207766 |
| hsa-miR-6769a-5p | 9.89E-01 | 8.24E-01 | 0.222948 | -6.0504 | 0.207087 |
| hsa-miR-4749-3p | 9.89E-01 | 8.24E-01 | 0.222776 | -6.0504 | 0.206927 |
| hsa-miR-1180-3p | 9.89E-01 | 8.24E-01 | 0.222772 | -6.0504 | 0.206923 |
| hsa-miR-3149 | 9.89E-01 | 8.24E-01 | -0.222597 | -6.0505 | -0.20676 |
| hsa-miR-32-3p | 9.89E-01 | 8.24E-01 | -0.222048 | -6.0506 | -0.206251 |
| hsa-miR-6772-5p | 9.89E-01 | 8.25E-01 | -0.221531 | -6.0507 | -0.20577 |
| hsa-miR-557 | 9.89E-01 | 8.25E-01 | 0.220728 | -6.0509 | 0.205025 |
| hsa-miR-2861 | 9.89E-01 | 8.26E-01 | 0.220088 | -6.051 | 0.20443 |
| hsa-miR-2392 | 9.89E-01 | 8.27E-01 | 0.218841 | -6.0513 | 0.203272 |
| hsa-miR-1281 | 9.89E-01 | 8.27E-01 | -0.218835 | -6.0513 | -0.203267 |
| hsa-let-7f-5p | 9.89E-01 | 8.27E-01 | -0.217987 | -6.0514 | -0.202478 |
| hsa-miR-652-5p | 9.89E-01 | 8.28E-01 | -0.217799 | -6.0515 | -0.202304 |
| hsa-miR-6748-5p | 9.89E-01 | 8.29E-01 | -0.216407 | -6.0518 | -0.201011 |
| hsa-miR-6875-5p | 9.89E-01 | 8.29E-01 | -0.215879 | -6.0519 | -0.200521 |
| hsa-miR-4784 | 9.89E-01 | 8.29E-01 | -0.215727 | -6.0519 | -0.20038 |
| hsa-miR-513a-5p | 9.89E-01 | 8.30E-01 | 0.214428 | -6.0522 | 0.199173 |
| hsa-miR-3174 | 9.89E-01 | 8.31E-01 | -0.213885 | -6.0523 | -0.198668 |
| hsa-miR-107 | 9.89E-01 | 8.31E-01 | -0.213481 | -6.0524 | -0.198293 |
| hsa-miR-3940-5p | 9.89E-01 | 8.32E-01 | 0.212062 | -6.0526 | 0.196975 |
| hsa-miR-148b-3p | 9.89E-01 | 8.33E-01 | -0.211251 | -6.0528 | -0.196222 |
| hsa-miR-497-5p | 9.89E-01 | 8.33E-01 | -0.211245 | -6.0528 | -0.196216 |
| hsa-miR-181c-5p | 9.89E-01 | 8.33E-01 | 0.210388 | -6.053 | 0.195421 |
| hsa-miR-758-5p | 9.89E-01 | 8.33E-01 | -0.210252 | -6.053 | -0.195294 |
| hsa-miR-330-3p | 9.89E-01 | 8.34E-01 | -0.210033 | -6.053 | -0.195091 |
| hsa-miR-7108-5p | 9.89E-01 | 8.35E-01 | 0.208308 | -6.0534 | 0.193488 |
| hsa-miR-4769-5p | 9.89E-01 | 8.35E-01 | 0.207672 | -6.0535 | 0.192897 |
| hsa-miR-6741-5p | 9.89E-01 | 8.36E-01 | 0.206595 | -6.0537 | 0.191897 |
| hsa-miR-8060 | 9.89E-01 | 8.37E-01 | -0.206175 | -6.0538 | -0.191507 |
| hsa-miR-4419b | 9.89E-01 | 8.37E-01 | -0.205895 | -6.0539 | -0.191247 |
| hsa-miR-8073 | 9.89E-01 | 8.37E-01 | -0.205104 | -6.054 | -0.190513 |
| hsa-miR-664a-3p | 9.89E-01 | 8.39E-01 | 0.202885 | -6.0544 | 0.188451 |
| hsa-miR-3154 | 9.89E-01 | 8.40E-01 | -0.202252 | -6.0546 | -0.187864 |
| hsa-miR-4430 | 9.89E-01 | 8.40E-01 | 0.20166 | -6.0547 | 0.187313 |
| hsa-miR-1305 | 9.89E-01 | 8.41E-01 | -0.200352 | -6.0549 | -0.186098 |
| hsa-miR-6797-3p | 9.89E-01 | 8.42E-01 | 0.199891 | -6.055 | 0.18567 |
| hsa-miR-3147 | 9.89E-01 | 8.42E-01 | 0.199851 | -6.055 | 0.185633 |
| hsa-miR-4442 | 9.89E-01 | 8.42E-01 | -0.199663 | -6.0551 | -0.185458 |
| hsa-miR-6796-5p | 9.89E-01 | 8.42E-01 | 0.198843 | -6.0552 | 0.184697 |
| hsa-miR-455-3p | 9.89E-01 | 8.43E-01 | 0.198207 | -6.0553 | 0.184106 |
| hsa-miR-505-3p | 9.89E-01 | 8.43E-01 | -0.197803 | -6.0554 | -0.18373 |
| hsa-miR-3137 | 9.89E-01 | 8.44E-01 | 0.196803 | -6.0556 | 0.182802 |
| hsa-miR-1226-5p | 9.89E-01 | 8.45E-01 | -0.195502 | -6.0558 | -0.181593 |
| hsa-miR-4726-5p | 9.89E-01 | 8.46E-01 | -0.193898 | -6.0561 | -0.180103 |
| hsa-miR-6089 | 9.89E-01 | 8.47E-01 | -0.192899 | -6.0563 | -0.179175 |
| hsa-miR-6510-5p | 9.89E-01 | 8.47E-01 | 0.192385 | -6.0564 | 0.178698 |
| hsa-miR-30d-5p | 9.89E-01 | 8.48E-01 | -0.191044 | -6.0567 | -0.177453 |
| hsa-let-7a-5p | 9.89E-01 | 8.49E-01 | 0.189863 | -6.0569 | 0.176355 |
| hsa-miR-4484 | 9.89E-01 | 8.52E-01 | -0.186663 | -6.0574 | -0.173383 |
| hsa-miR-6830-5p | 9.89E-01 | 8.52E-01 | -0.186295 | -6.0575 | -0.173042 |
| hsa-miR-4444 | 9.89E-01 | 8.53E-01 | -0.18483 | -6.0578 | -0.171681 |
| hsa-miR-4260 | 9.89E-01 | 8.55E-01 | 0.182757 | -6.0581 | 0.169755 |
| hsa-miR-8089 | 9.89E-01 | 8.56E-01 | 0.181829 | -6.0583 | 0.168893 |
| hsa-miR-5001-5p | 9.89E-01 | 8.56E-01 | -0.181506 | -6.0583 | -0.168593 |
| hsa-miR-27a-3p | 9.89E-01 | 8.57E-01 | 0.180727 | -6.0585 | 0.16787 |
| hsa-miR-324-3p | 9.89E-01 | 8.57E-01 | -0.180076 | -6.0586 | -0.167265 |
| hsa-miR-3135b | 9.89E-01 | 8.57E-01 | 0.180061 | -6.0586 | 0.167251 |
| hsa-miR-6799-5p | 9.89E-01 | 8.59E-01 | -0.177386 | -6.059 | -0.164766 |
| hsa-miR-1183 | 9.89E-01 | 8.59E-01 | -0.177074 | -6.0591 | -0.164476 |
| hsa-miR-6776-5p | 9.89E-01 | 8.60E-01 | 0.176451 | -6.0592 | 0.163897 |
| hsa-miR-340-5p | 9.89E-01 | 8.60E-01 | -0.175756 | -6.0593 | -0.163252 |
| hsa-miR-6511b-5p | 9.89E-01 | 8.61E-01 | -0.175424 | -6.0594 | -0.162944 |
| hsa-miR-3917 | 9.89E-01 | 8.62E-01 | -0.174297 | -6.0596 | -0.161897 |
| hsa-miR-6881-5p | 9.89E-01 | 8.62E-01 | 0.174067 | -6.0596 | 0.161683 |
| hsa-miR-766-3p | 9.89E-01 | 8.63E-01 | -0.173124 | -6.0597 | -0.160808 |
| hsa-miR-4485-5p | 9.89E-01 | 8.63E-01 | 0.172925 | -6.0598 | 0.160622 |
| hsa-miR-526b-5p | 9.89E-01 | 8.63E-01 | 0.172889 | -6.0598 | 0.160589 |
| hsa-miR-6125 | 9.89E-01 | 8.63E-01 | -0.172547 | -6.0598 | -0.160271 |
| hsa-miR-6509-5p | 9.89E-01 | 8.64E-01 | -0.171449 | -6.06 | -0.159252 |
| hsa-miR-30b-3p | 9.89E-01 | 8.65E-01 | -0.170365 | -6.0602 | -0.158245 |
| hsa-miR-193b-3p | 9.89E-01 | 8.66E-01 | -0.168858 | -6.0604 | -0.156845 |
| hsa-miR-4466 | 9.89E-01 | 8.67E-01 | 0.167971 | -6.0606 | 0.156021 |
| hsa-miR-4486 | 9.89E-01 | 8.67E-01 | -0.167866 | -6.0606 | -0.155924 |
| hsa-miR-331-3p | 9.89E-01 | 8.67E-01 | 0.167119 | -6.0607 | 0.15523 |
| hsa-miR-6717-5p | 9.89E-01 | 8.68E-01 | -0.166184 | -6.0609 | -0.154361 |
| hsa-miR-5189-5p | 9.89E-01 | 8.69E-01 | -0.164334 | -6.0612 | -0.152643 |
| hsa-miR-6774-5p | 9.89E-01 | 8.70E-01 | -0.163289 | -6.0613 | -0.151672 |
| hsa-miR-6882-5p | 9.89E-01 | 8.71E-01 | -0.162051 | -6.0615 | -0.150522 |
| hsa-miR-1181 | 9.89E-01 | 8.73E-01 | -0.1594 | -6.0619 | -0.14806 |
| hsa-miR-671-5p | 9.89E-01 | 8.74E-01 | 0.158538 | -6.062 | 0.14726 |
| hsa-miR-320a | 9.89E-01 | 8.75E-01 | -0.157783 | -6.0622 | -0.146558 |
| hsa-miR-3188 | 9.89E-01 | 8.75E-01 | 0.157311 | -6.0622 | 0.146119 |
| hsa-miR-30c-5p | 9.89E-01 | 8.76E-01 | -0.156442 | -6.0624 | -0.145312 |
| hsa-miR-6869-5p | 9.89E-01 | 8.76E-01 | 0.155852 | -6.0624 | 0.144764 |
| hsa-miR-6813-3p | 9.89E-01 | 8.76E-01 | -0.155793 | -6.0625 | -0.144709 |
| hsa-miR-3195 | 9.89E-01 | 8.77E-01 | 0.155331 | -6.0625 | 0.144281 |
| hsa-miR-4446-3p | 9.89E-01 | 8.77E-01 | 0.155257 | -6.0625 | 0.144212 |
| hsa-miR-6833-5p | 9.89E-01 | 8.80E-01 | 0.151529 | -6.0631 | 0.140749 |
| hsa-miR-324-5p | 9.89E-01 | 8.81E-01 | 0.14971 | -6.0633 | 0.139059 |
| hsa-miR-320b | 9.89E-01 | 8.81E-01 | -0.149083 | -6.0634 | -0.138477 |
| hsa-miR-193a-3p | 9.89E-01 | 8.83E-01 | 0.147751 | -6.0636 | 0.137239 |
| hsa-miR-6757-5p | 9.89E-01 | 8.83E-01 | 0.147468 | -6.0636 | 0.136977 |
| hsa-miR-181d-5p | 9.89E-01 | 8.83E-01 | -0.146869 | -6.0637 | -0.136421 |
| hsa-miR-6861-5p | 9.89E-01 | 8.84E-01 | 0.146495 | -6.0638 | 0.136073 |
| hsa-miR-4646-5p | 9.89E-01 | 8.84E-01 | -0.145789 | -6.0639 | -0.135417 |
| hsa-miR-1238-3p | 9.89E-01 | 8.84E-01 | 0.145337 | -6.0639 | 0.134998 |
| hsa-miR-132-3p | 9.89E-01 | 8.85E-01 | -0.145024 | -6.064 | -0.134707 |
| hsa-miR-361-5p | 9.89E-01 | 8.85E-01 | -0.144892 | -6.064 | -0.134584 |
| hsa-miR-3937 | 9.89E-01 | 8.86E-01 | 0.143729 | -6.0642 | 0.133504 |
| hsa-miR-8063 | 9.89E-01 | 8.86E-01 | -0.143541 | -6.0642 | -0.133329 |
| hsa-miR-4710 | 9.89E-01 | 8.87E-01 | 0.14219 | -6.0644 | 0.132074 |
| hsa-miR-4689 | 9.89E-01 | 8.88E-01 | 0.14072 | -6.0646 | 0.130709 |
| hsa-miR-513c-5p | 9.89E-01 | 8.89E-01 | 0.139023 | -6.0648 | 0.129133 |
| hsa-miR-4667-5p | 9.89E-01 | 8.91E-01 | -0.13724 | -6.065 | -0.127476 |
| hsa-miR-610 | 9.89E-01 | 8.91E-01 | -0.136837 | -6.0651 | -0.127102 |
| hsa-miR-3162-3p | 9.89E-01 | 8.92E-01 | -0.135609 | -6.0652 | -0.125961 |
| hsa-miR-574-5p | 9.89E-01 | 8.92E-01 | 0.135571 | -6.0652 | 0.125926 |
| hsa-miR-6795-5p | 9.89E-01 | 8.93E-01 | -0.134213 | -6.0654 | -0.124665 |
| hsa-miR-27b-3p | 9.89E-01 | 8.94E-01 | -0.133504 | -6.0655 | -0.124006 |
| hsa-miR-6815-5p | 9.89E-01 | 8.94E-01 | -0.132988 | -6.0656 | -0.123527 |
| hsa-miR-197-3p | 9.89E-01 | 8.94E-01 | 0.132721 | -6.0656 | 0.123279 |
| hsa-miR-6780a-5p | 9.89E-01 | 8.95E-01 | -0.132157 | -6.0657 | -0.122755 |
| hsa-miR-24-3p | 9.89E-01 | 8.96E-01 | 0.131294 | -6.0658 | 0.121954 |
| hsa-miR-4291 | 9.89E-01 | 8.96E-01 | 0.130872 | -6.0658 | 0.121562 |
| hsa-miR-6069 | 9.89E-01 | 8.96E-01 | 0.130282 | -6.0659 | 0.121014 |
| hsa-miR-4655-5p | 9.89E-01 | 8.98E-01 | 0.128474 | -6.0661 | 0.119334 |
| hsa-miR-6820-5p | 9.89E-01 | 8.98E-01 | -0.128262 | -6.0662 | -0.119137 |
| hsa-miR-378c | 9.89E-01 | 8.98E-01 | 0.127755 | -6.0662 | 0.118666 |
| hsa-miR-663a | 9.89E-01 | 8.99E-01 | 0.127392 | -6.0663 | 0.118329 |
| hsa-miR-4436b-3p | 9.89E-01 | 9.00E-01 | -0.126194 | -6.0664 | -0.117216 |
| hsa-miR-1915-3p | 9.89E-01 | 9.00E-01 | 0.126078 | -6.0664 | 0.117108 |
| hsa-miR-6740-5p | 9.89E-01 | 9.00E-01 | 0.125766 | -6.0665 | 0.116818 |
| hsa-miR-6894-5p | 9.89E-01 | 9.00E-01 | 0.125613 | -6.0665 | 0.116677 |
| hsa-miR-7152-3p | 9.89E-01 | 9.03E-01 | 0.122277 | -6.0669 | 0.113578 |
| hsa-miR-10a-5p | 9.89E-01 | 9.03E-01 | -0.121453 | -6.067 | -0.112813 |
| hsa-miR-4419a | 9.89E-01 | 9.04E-01 | 0.120263 | -6.0671 | 0.111707 |
| hsa-miR-572 | 9.89E-01 | 9.05E-01 | 0.118853 | -6.0673 | 0.110398 |
| hsa-miR-4516 | 9.89E-01 | 9.06E-01 | -0.118437 | -6.0673 | -0.110011 |
| hsa-miR-4732-5p | 9.89E-01 | 9.07E-01 | 0.117245 | -6.0674 | 0.108903 |
| hsa-miR-4257 | 9.89E-01 | 9.07E-01 | -0.116993 | -6.0675 | -0.10867 |
| hsa-miR-450a-5p | 9.89E-01 | 9.07E-01 | 0.116396 | -6.0675 | 0.108116 |
| hsa-miR-1227-5p | 9.89E-01 | 9.08E-01 | 0.115125 | -6.0677 | 0.106934 |
| hsa-miR-7515 | 9.89E-01 | 9.11E-01 | -0.11183 | -6.068 | -0.103875 |
| hsa-miR-513b-5p | 9.89E-01 | 9.11E-01 | 0.111477 | -6.0681 | 0.103546 |
| hsa-miR-4688 | 9.89E-01 | 9.12E-01 | -0.110112 | -6.0682 | -0.102278 |
| hsa-miR-326 | 9.89E-01 | 9.12E-01 | 0.110021 | -6.0682 | 0.102194 |
| hsa-miR-664b-3p | 9.89E-01 | 9.13E-01 | -0.109706 | -6.0683 | -0.101901 |
| hsa-miR-6784-5p | 9.89E-01 | 9.14E-01 | -0.108083 | -6.0684 | -0.100394 |
| hsa-miR-3976 | 9.89E-01 | 9.15E-01 | -0.107261 | -6.0685 | -0.09963 |
| hsa-miR-6076 | 9.89E-01 | 9.15E-01 | 0.106435 | -6.0686 | 0.098863 |
| hsa-miR-1273f | 9.89E-01 | 9.16E-01 | -0.105492 | -6.0687 | -0.097987 |
| hsa-miR-339-3p | 9.89E-01 | 9.16E-01 | -0.105344 | -6.0687 | -0.09785 |
| hsa-miR-328-5p | 9.89E-01 | 9.17E-01 | -0.104508 | -6.0688 | -0.097073 |
| hsa-miR-6845-5p | 9.89E-01 | 9.17E-01 | -0.104158 | -6.0688 | -0.096748 |
| hsa-miR-4716-3p | 9.89E-01 | 9.17E-01 | -0.104139 | -6.0688 | -0.09673 |
| hsa-miR-4299 | 9.89E-01 | 9.17E-01 | 0.103797 | -6.0688 | 0.096412 |
| hsa-miR-6887-5p | 9.89E-01 | 9.18E-01 | -0.10357 | -6.0689 | -0.096201 |
| hsa-miR-4669 | 9.89E-01 | 9.18E-01 | 0.103214 | -6.0689 | 0.095871 |
| hsa-miR-150-3p | 9.89E-01 | 9.19E-01 | -0.101696 | -6.0691 | -0.094461 |
| hsa-miR-1233-5p | 9.89E-01 | 9.19E-01 | -0.101624 | -6.0691 | -0.094394 |
| hsa-miR-8064 | 9.89E-01 | 9.20E-01 | -0.100071 | -6.0692 | -0.092952 |
| hsa-miR-4787-3p | 9.89E-01 | 9.21E-01 | -0.099793 | -6.0692 | -0.092693 |
| hsa-miR-659-3p | 9.89E-01 | 9.21E-01 | -0.099152 | -6.0693 | -0.092098 |
| hsa-miR-6734-5p | 9.89E-01 | 9.22E-01 | 0.098062 | -6.0694 | 0.091086 |
| hsa-miR-125a-3p | 9.89E-01 | 9.22E-01 | -0.097872 | -6.0694 | -0.090909 |
| hsa-miR-21-5p | 9.89E-01 | 9.23E-01 | -0.097019 | -6.0695 | -0.090117 |
| hsa-miR-6087 | 9.89E-01 | 9.23E-01 | -0.096653 | -6.0695 | -0.089777 |
| hsa-miR-103a-3p | 9.89E-01 | 9.23E-01 | 0.096267 | -6.0696 | 0.089418 |
| hsa-miR-195-5p | 9.89E-01 | 9.23E-01 | -0.096134 | -6.0696 | -0.089295 |
| hsa-miR-7845-5p | 9.89E-01 | 9.25E-01 | 0.094451 | -6.0697 | 0.087732 |
| hsa-miR-6886-5p | 9.89E-01 | 9.25E-01 | -0.09393 | -6.0698 | -0.087248 |
| hsa-miR-6890-5p | 9.89E-01 | 9.25E-01 | 0.09374 | -6.0698 | 0.087071 |
| hsa-miR-4690-5p | 9.89E-01 | 9.26E-01 | 0.093359 | -6.0698 | 0.086717 |
| hsa-miR-29c-3p | 9.89E-01 | 9.26E-01 | 0.092942 | -6.0699 | 0.086329 |
| hsa-miR-5581-5p | 9.89E-01 | 9.27E-01 | -0.092101 | -6.0699 | -0.085548 |
| hsa-miR-625-5p | 9.89E-01 | 9.28E-01 | -0.090824 | -6.07 | -0.084363 |
| hsa-miR-1914-3p | 9.89E-01 | 9.30E-01 | -0.088434 | -6.0702 | -0.082142 |
| hsa-miR-3663-3p | 9.89E-01 | 9.30E-01 | -0.087911 | -6.0703 | -0.081657 |
| hsa-miR-4634 | 9.89E-01 | 9.31E-01 | 0.086534 | -6.0704 | 0.080378 |
| hsa-miR-6723-5p | 9.89E-01 | 9.32E-01 | -0.08531 | -6.0705 | -0.079241 |
| hsa-miR-4253 | 9.89E-01 | 9.32E-01 | 0.085044 | -6.0705 | 0.078994 |
| hsa-miR-199b-5p | 9.89E-01 | 9.33E-01 | -0.084078 | -6.0706 | -0.078097 |
| hsa-miR-4660 | 9.89E-01 | 9.33E-01 | 0.083591 | -6.0706 | 0.077644 |
| hsa-miR-23b-3p | 9.89E-01 | 9.34E-01 | 0.083197 | -6.0707 | 0.077278 |
| hsa-miR-136-5p | 9.89E-01 | 9.34E-01 | -0.082809 | -6.0707 | -0.076918 |
| hsa-miR-3202 | 9.89E-01 | 9.34E-01 | 0.082227 | -6.0708 | 0.076377 |
| hsa-miR-887-3p | 9.89E-01 | 9.35E-01 | 0.081578 | -6.0708 | 0.075775 |
| hsa-miR-6737-3p | 9.89E-01 | 9.35E-01 | 0.081559 | -6.0708 | 0.075756 |
| hsa-miR-296-5p | 9.89E-01 | 9.36E-01 | 0.080925 | -6.0709 | 0.075168 |
| hsa-miR-6824-5p | 9.89E-01 | 9.37E-01 | -0.079423 | -6.071 | -0.073773 |
| hsa-miR-5006-5p | 9.89E-01 | 9.37E-01 | 0.079157 | -6.071 | 0.073526 |
| hsa-miR-595 | 9.89E-01 | 9.37E-01 | 0.079115 | -6.071 | 0.073487 |
| hsa-miR-4758-3p | 9.89E-01 | 9.37E-01 | 0.078727 | -6.071 | 0.073126 |
| hsa-miR-5585-3p | 9.89E-01 | 9.41E-01 | -0.073549 | -6.0714 | -0.068316 |
| hsa-miR-1185-1-3p | 9.89E-01 | 9.42E-01 | -0.073349 | -6.0714 | -0.068131 |
| hsa-miR-4685-5p | 9.89E-01 | 9.42E-01 | 0.072391 | -6.0715 | 0.067241 |
| hsa-miR-3127-5p | 9.89E-01 | 9.42E-01 | -0.072199 | -6.0715 | -0.067062 |
| hsa-miR-6872-5p | 9.89E-01 | 9.42E-01 | -0.072189 | -6.0715 | -0.067053 |
| hsa-miR-4294 | 9.89E-01 | 9.44E-01 | -0.069967 | -6.0716 | -0.064989 |
| hsa-miR-3125 | 9.89E-01 | 9.45E-01 | -0.069439 | -6.0717 | -0.064499 |
| hsa-miR-6765-5p | 9.89E-01 | 9.45E-01 | 0.069062 | -6.0717 | 0.064149 |
| hsa-miR-6083 | 9.89E-01 | 9.45E-01 | -0.06852 | -6.0717 | -0.063645 |
| hsa-miR-6877-5p | 9.89E-01 | 9.48E-01 | 0.065633 | -6.0719 | 0.060964 |
| hsa-miR-4793-5p | 9.89E-01 | 9.48E-01 | 0.064756 | -6.072 | 0.060149 |
| hsa-miR-4746-3p | 9.89E-01 | 9.50E-01 | 0.0631 | -6.0721 | 0.058611 |
| hsa-miR-4259 | 9.89E-01 | 9.50E-01 | -0.062885 | -6.0721 | -0.058411 |
| hsa-miR-7106-5p | 9.89E-01 | 9.50E-01 | 0.062129 | -6.0721 | 0.057709 |
| hsa-miR-6879-5p | 9.89E-01 | 9.51E-01 | 0.060937 | -6.0722 | 0.056602 |
| hsa-miR-345-3p | 9.89E-01 | 9.52E-01 | 0.060806 | -6.0722 | 0.05648 |
| hsa-miR-6847-5p | 9.89E-01 | 9.52E-01 | -0.060108 | -6.0722 | -0.055832 |
| hsa-miR-320c | 9.89E-01 | 9.52E-01 | -0.059765 | -6.0723 | -0.055513 |
| hsa-miR-711 | 9.89E-01 | 9.53E-01 | -0.058679 | -6.0723 | -0.054504 |
| hsa-miR-7704 | 9.89E-01 | 9.53E-01 | -0.058484 | -6.0723 | -0.054323 |
| hsa-miR-4470 | 9.89E-01 | 9.54E-01 | -0.057661 | -6.0724 | -0.053559 |
| hsa-miR-5194 | 9.89E-01 | 9.54E-01 | 0.057525 | -6.0724 | 0.053433 |
| hsa-miR-30e-3p | 9.89E-01 | 9.55E-01 | 0.056633 | -6.0724 | 0.052604 |
| hsa-miR-6753-3p | 9.89E-01 | 9.56E-01 | -0.055793 | -6.0725 | -0.051824 |
| hsa-miR-4436b-5p | 9.89E-01 | 9.56E-01 | -0.055251 | -6.0725 | -0.051321 |
| hsa-miR-3173-3p | 9.89E-01 | 9.56E-01 | -0.05507 | -6.0725 | -0.051152 |
| hsa-miR-126-3p | 9.89E-01 | 9.57E-01 | -0.05415 | -6.0726 | -0.050297 |
| hsa-miR-6829-5p | 9.89E-01 | 9.57E-01 | 0.05362 | -6.0726 | 0.049806 |
| hsa-miR-6803-5p | 9.89E-01 | 9.58E-01 | 0.052879 | -6.0726 | 0.049117 |
| hsa-miR-8069 | 9.89E-01 | 9.58E-01 | -0.05258 | -6.0726 | -0.04884 |
| hsa-miR-4478 | 9.89E-01 | 9.59E-01 | 0.051972 | -6.0727 | 0.048275 |
| hsa-miR-378b | 9.89E-01 | 9.60E-01 | -0.050167 | -6.0728 | -0.046598 |
| hsa-miR-6819-3p | 9.89E-01 | 9.61E-01 | 0.048658 | -6.0728 | 0.045197 |
| hsa-miR-4455 | 9.89E-01 | 9.62E-01 | 0.047934 | -6.0729 | 0.044523 |
| hsa-miR-3665 | 9.89E-01 | 9.62E-01 | 0.047551 | -6.0729 | 0.044168 |
| hsa-miR-4653-3p | 9.89E-01 | 9.62E-01 | 0.047153 | -6.0729 | 0.043798 |
| hsa-miR-6791-5p | 9.89E-01 | 9.62E-01 | -0.047063 | -6.0729 | -0.043715 |
| hsa-miR-4750-5p | 9.89E-01 | 9.64E-01 | 0.045551 | -6.073 | 0.04231 |
| hsa-miR-7846-3p | 9.89E-01 | 9.64E-01 | 0.045268 | -6.073 | 0.042048 |
| hsa-miR-4271 | 9.89E-01 | 9.66E-01 | 0.042611 | -6.0731 | 0.03958 |
| hsa-miR-4758-5p | 9.89E-01 | 9.67E-01 | -0.041466 | -6.0731 | -0.038516 |
| hsa-miR-7847-3p | 9.89E-01 | 9.67E-01 | 0.041346 | -6.0731 | 0.038404 |
| hsa-miR-6767-5p | 9.89E-01 | 9.68E-01 | -0.039588 | -6.0732 | -0.036772 |
| hsa-miR-4433a-3p | 9.89E-01 | 9.69E-01 | 0.039404 | -6.0732 | 0.036601 |
| hsa-miR-6808-5p | 9.89E-01 | 9.70E-01 | 0.038138 | -6.0733 | 0.035425 |
| hsa-miR-744-5p | 9.89E-01 | 9.70E-01 | 0.037314 | -6.0733 | 0.034659 |
| hsa-miR-371a-5p | 9.89E-01 | 9.70E-01 | -0.037205 | -6.0733 | -0.034558 |
| hsa-miR-7851-3p | 9.89E-01 | 9.70E-01 | 0.037173 | -6.0733 | 0.034528 |
| hsa-let-7a-3p | 9.89E-01 | 9.72E-01 | -0.035388 | -6.0734 | -0.03287 |
| hsa-let-7b-3p | 9.89E-01 | 9.72E-01 | -0.035388 | -6.0734 | -0.03287 |
| hsa-let-7c-3p | 9.89E-01 | 9.72E-01 | -0.035388 | -6.0734 | -0.03287 |
| hsa-let-7d-3p | 9.89E-01 | 9.72E-01 | -0.035388 | -6.0734 | -0.03287 |
| hsa-let-7e-3p | 9.89E-01 | 9.72E-01 | -0.035388 | -6.0734 | -0.03287 |
| hsa-let-7f-1-3p | 9.89E-01 | 9.72E-01 | -0.035388 | -6.0734 | -0.03287 |
| hsa-let-7f-2-3p | 9.89E-01 | 9.72E-01 | -0.035388 | -6.0734 | -0.03287 |
| hsa-let-7g-3p | 9.89E-01 | 9.72E-01 | -0.035388 | -6.0734 | -0.03287 |
| hsa-let-7i-3p | 9.89E-01 | 9.72E-01 | -0.035388 | -6.0734 | -0.03287 |
| hsa-miR-1-5p | 9.89E-01 | 9.72E-01 | -0.035388 | -6.0734 | -0.03287 |
| hsa-miR-100-3p | 9.89E-01 | 9.72E-01 | -0.035388 | -6.0734 | -0.03287 |
| hsa-miR-101-5p | 9.89E-01 | 9.72E-01 | -0.035388 | -6.0734 | -0.03287 |
| hsa-miR-103a-2-5p | 9.89E-01 | 9.72E-01 | -0.035388 | -6.0734 | -0.03287 |
| hsa-miR-103b | 9.89E-01 | 9.72E-01 | -0.035388 | -6.0734 | -0.03287 |
| hsa-miR-105-3p | 9.89E-01 | 9.72E-01 | -0.035388 | -6.0734 | -0.03287 |
| hsa-miR-105-5p | 9.89E-01 | 9.72E-01 | -0.035388 | -6.0734 | -0.03287 |
| hsa-miR-106a-3p | 9.89E-01 | 9.72E-01 | -0.035388 | -6.0734 | -0.03287 |
| hsa-miR-106b-3p | 9.89E-01 | 9.72E-01 | -0.035388 | -6.0734 | -0.03287 |
| hsa-miR-10b-3p | 9.89E-01 | 9.72E-01 | -0.035388 | -6.0734 | -0.03287 |
| hsa-miR-1178-3p | 9.89E-01 | 9.72E-01 | -0.035388 | -6.0734 | -0.03287 |
| hsa-miR-1178-5p | 9.89E-01 | 9.72E-01 | -0.035388 | -6.0734 | -0.03287 |
| hsa-miR-1179 | 9.89E-01 | 9.72E-01 | -0.035388 | -6.0734 | -0.03287 |
| hsa-miR-1180-5p | 9.89E-01 | 9.72E-01 | -0.035388 | -6.0734 | -0.03287 |
| hsa-miR-1184 | 9.89E-01 | 9.72E-01 | -0.035388 | -6.0734 | -0.03287 |
| hsa-miR-1185-5p | 9.89E-01 | 9.72E-01 | -0.035388 | -6.0734 | -0.03287 |
| hsa-miR-1193 | 9.89E-01 | 9.72E-01 | -0.035388 | -6.0734 | -0.03287 |
| hsa-miR-1197 | 9.89E-01 | 9.72E-01 | -0.035388 | -6.0734 | -0.03287 |
| hsa-miR-1199-3p | 9.89E-01 | 9.72E-01 | -0.035388 | -6.0734 | -0.03287 |
| hsa-miR-1199-5p | 9.89E-01 | 9.72E-01 | -0.035388 | -6.0734 | -0.03287 |
| hsa-miR-1200 | 9.89E-01 | 9.72E-01 | -0.035388 | -6.0734 | -0.03287 |
| hsa-miR-1203 | 9.89E-01 | 9.72E-01 | -0.035388 | -6.0734 | -0.03287 |
| hsa-miR-1204 | 9.89E-01 | 9.72E-01 | -0.035388 | -6.0734 | -0.03287 |
| hsa-miR-1205 | 9.89E-01 | 9.72E-01 | -0.035388 | -6.0734 | -0.03287 |
| hsa-miR-1206 | 9.89E-01 | 9.72E-01 | -0.035388 | -6.0734 | -0.03287 |
| hsa-miR-1207-3p | 9.89E-01 | 9.72E-01 | -0.035388 | -6.0734 | -0.03287 |
| hsa-miR-1224-3p | 9.89E-01 | 9.72E-01 | -0.035388 | -6.0734 | -0.03287 |
| hsa-miR-1226-3p | 9.89E-01 | 9.72E-01 | -0.035388 | -6.0734 | -0.03287 |
| hsa-miR-1227-3p | 9.89E-01 | 9.72E-01 | -0.035388 | -6.0734 | -0.03287 |
| hsa-miR-1229-3p | 9.89E-01 | 9.72E-01 | -0.035388 | -6.0734 | -0.03287 |
| hsa-miR-1231 | 9.89E-01 | 9.72E-01 | -0.035388 | -6.0734 | -0.03287 |
| hsa-miR-1233-3p | 9.89E-01 | 9.72E-01 | -0.035388 | -6.0734 | -0.03287 |
| hsa-miR-1236-3p | 9.89E-01 | 9.72E-01 | -0.035388 | -6.0734 | -0.03287 |
| hsa-miR-1237-5p | 9.89E-01 | 9.72E-01 | -0.035388 | -6.0734 | -0.03287 |
| hsa-miR-124-3p | 9.89E-01 | 9.72E-01 | -0.035388 | -6.0734 | -0.03287 |
| hsa-miR-124-5p | 9.89E-01 | 9.72E-01 | -0.035388 | -6.0734 | -0.03287 |
| hsa-miR-1243 | 9.89E-01 | 9.72E-01 | -0.035388 | -6.0734 | -0.03287 |
| hsa-miR-1245a | 9.89E-01 | 9.72E-01 | -0.035388 | -6.0734 | -0.03287 |
| hsa-miR-1245b-3p | 9.89E-01 | 9.72E-01 | -0.035388 | -6.0734 | -0.03287 |
| hsa-miR-1245b-5p | 9.89E-01 | 9.72E-01 | -0.035388 | -6.0734 | -0.03287 |
| hsa-miR-1248 | 9.89E-01 | 9.72E-01 | -0.035388 | -6.0734 | -0.03287 |
| hsa-miR-1250-3p | 9.89E-01 | 9.72E-01 | -0.035388 | -6.0734 | -0.03287 |
| hsa-miR-1250-5p | 9.89E-01 | 9.72E-01 | -0.035388 | -6.0734 | -0.03287 |
| hsa-miR-1251-3p | 9.89E-01 | 9.72E-01 | -0.035388 | -6.0734 | -0.03287 |
| hsa-miR-1251-5p | 9.89E-01 | 9.72E-01 | -0.035388 | -6.0734 | -0.03287 |
| hsa-miR-1252-3p | 9.89E-01 | 9.72E-01 | -0.035388 | -6.0734 | -0.03287 |
| hsa-miR-1252-5p | 9.89E-01 | 9.72E-01 | -0.035388 | -6.0734 | -0.03287 |
| hsa-miR-1253 | 9.89E-01 | 9.72E-01 | -0.035388 | -6.0734 | -0.03287 |
| hsa-miR-1254 | 9.89E-01 | 9.72E-01 | -0.035388 | -6.0734 | -0.03287 |
| hsa-miR-1255a | 9.89E-01 | 9.72E-01 | -0.035388 | -6.0734 | -0.03287 |
| hsa-miR-1255b-2-3p | 9.89E-01 | 9.72E-01 | -0.035388 | -6.0734 | -0.03287 |
| hsa-miR-1255b-5p | 9.89E-01 | 9.72E-01 | -0.035388 | -6.0734 | -0.03287 |
| hsa-miR-1256 | 9.89E-01 | 9.72E-01 | -0.035388 | -6.0734 | -0.03287 |
| hsa-miR-1257 | 9.89E-01 | 9.72E-01 | -0.035388 | -6.0734 | -0.03287 |
| hsa-miR-1258 | 9.89E-01 | 9.72E-01 | -0.035388 | -6.0734 | -0.03287 |
| hsa-miR-1261 | 9.89E-01 | 9.72E-01 | -0.035388 | -6.0734 | -0.03287 |
| hsa-miR-1262 | 9.89E-01 | 9.72E-01 | -0.035388 | -6.0734 | -0.03287 |
| hsa-miR-1263 | 9.89E-01 | 9.72E-01 | -0.035388 | -6.0734 | -0.03287 |
| hsa-miR-1264 | 9.89E-01 | 9.72E-01 | -0.035388 | -6.0734 | -0.03287 |
| hsa-miR-1265 | 9.89E-01 | 9.72E-01 | -0.035388 | -6.0734 | -0.03287 |
| hsa-miR-1266-3p | 9.89E-01 | 9.72E-01 | -0.035388 | -6.0734 | -0.03287 |
| hsa-miR-1266-5p | 9.89E-01 | 9.72E-01 | -0.035388 | -6.0734 | -0.03287 |
| hsa-miR-1267 | 9.89E-01 | 9.72E-01 | -0.035388 | -6.0734 | -0.03287 |
| hsa-miR-1269a | 9.89E-01 | 9.72E-01 | -0.035388 | -6.0734 | -0.03287 |
| hsa-miR-1269b | 9.89E-01 | 9.72E-01 | -0.035388 | -6.0734 | -0.03287 |
| hsa-miR-127-5p | 9.89E-01 | 9.72E-01 | -0.035388 | -6.0734 | -0.03287 |
| hsa-miR-1270 | 9.89E-01 | 9.72E-01 | -0.035388 | -6.0734 | -0.03287 |
| hsa-miR-1271-3p | 9.89E-01 | 9.72E-01 | -0.035388 | -6.0734 | -0.03287 |
| hsa-miR-1272 | 9.89E-01 | 9.72E-01 | -0.035388 | -6.0734 | -0.03287 |
| hsa-miR-1273a | 9.89E-01 | 9.72E-01 | -0.035388 | -6.0734 | -0.03287 |
| hsa-miR-1273h-3p | 9.89E-01 | 9.72E-01 | -0.035388 | -6.0734 | -0.03287 |
| hsa-miR-1276 | 9.89E-01 | 9.72E-01 | -0.035388 | -6.0734 | -0.03287 |
| hsa-miR-1277-3p | 9.89E-01 | 9.72E-01 | -0.035388 | -6.0734 | -0.03287 |
| hsa-miR-1278 | 9.89E-01 | 9.72E-01 | -0.035388 | -6.0734 | -0.03287 |
| hsa-miR-1279 | 9.89E-01 | 9.72E-01 | -0.035388 | -6.0734 | -0.03287 |
| hsa-miR-128-1-5p | 9.89E-01 | 9.72E-01 | -0.035388 | -6.0734 | -0.03287 |
| hsa-miR-128-2-5p | 9.89E-01 | 9.72E-01 | -0.035388 | -6.0734 | -0.03287 |
| hsa-miR-1282 | 9.89E-01 | 9.72E-01 | -0.035388 | -6.0734 | -0.03287 |
| hsa-miR-1283 | 9.89E-01 | 9.72E-01 | -0.035388 | -6.0734 | -0.03287 |
| hsa-miR-1284 | 9.89E-01 | 9.72E-01 | -0.035388 | -6.0734 | -0.03287 |
| hsa-miR-1285-5p | 9.89E-01 | 9.72E-01 | -0.035388 | -6.0734 | -0.03287 |
| hsa-miR-1286 | 9.89E-01 | 9.72E-01 | -0.035388 | -6.0734 | -0.03287 |
| hsa-miR-1287-3p | 9.89E-01 | 9.72E-01 | -0.035388 | -6.0734 | -0.03287 |
| hsa-miR-1287-5p | 9.89E-01 | 9.72E-01 | -0.035388 | -6.0734 | -0.03287 |
| hsa-miR-1288-5p | 9.89E-01 | 9.72E-01 | -0.035388 | -6.0734 | -0.03287 |
| hsa-miR-1289 | 9.89E-01 | 9.72E-01 | -0.035388 | -6.0734 | -0.03287 |
| hsa-miR-129-1-3p | 9.89E-01 | 9.72E-01 | -0.035388 | -6.0734 | -0.03287 |
| hsa-miR-129-2-3p | 9.89E-01 | 9.72E-01 | -0.035388 | -6.0734 | -0.03287 |
| hsa-miR-129-5p | 9.89E-01 | 9.72E-01 | -0.035388 | -6.0734 | -0.03287 |
| hsa-miR-1292-3p | 9.89E-01 | 9.72E-01 | -0.035388 | -6.0734 | -0.03287 |
| hsa-miR-1292-5p | 9.89E-01 | 9.72E-01 | -0.035388 | -6.0734 | -0.03287 |
| hsa-miR-1293 | 9.89E-01 | 9.72E-01 | -0.035388 | -6.0734 | -0.03287 |
| hsa-miR-1294 | 9.89E-01 | 9.72E-01 | -0.035388 | -6.0734 | -0.03287 |
| hsa-miR-1295a | 9.89E-01 | 9.72E-01 | -0.035388 | -6.0734 | -0.03287 |
| hsa-miR-1295b-3p | 9.89E-01 | 9.72E-01 | -0.035388 | -6.0734 | -0.03287 |
| hsa-miR-1295b-5p | 9.89E-01 | 9.72E-01 | -0.035388 | -6.0734 | -0.03287 |
| hsa-miR-1296-3p | 9.89E-01 | 9.72E-01 | -0.035388 | -6.0734 | -0.03287 |
| hsa-miR-1296-5p | 9.89E-01 | 9.72E-01 | -0.035388 | -6.0734 | -0.03287 |
| hsa-miR-1297 | 9.89E-01 | 9.72E-01 | -0.035388 | -6.0734 | -0.03287 |
| hsa-miR-1298-3p | 9.89E-01 | 9.72E-01 | -0.035388 | -6.0734 | -0.03287 |
| hsa-miR-1298-5p | 9.89E-01 | 9.72E-01 | -0.035388 | -6.0734 | -0.03287 |
| hsa-miR-1301-3p | 9.89E-01 | 9.72E-01 | -0.035388 | -6.0734 | -0.03287 |
| hsa-miR-1301-5p | 9.89E-01 | 9.72E-01 | -0.035388 | -6.0734 | -0.03287 |
| hsa-miR-1302 | 9.89E-01 | 9.72E-01 | -0.035388 | -6.0734 | -0.03287 |
| hsa-miR-1303 | 9.89E-01 | 9.72E-01 | -0.035388 | -6.0734 | -0.03287 |
| hsa-miR-1304-5p | 9.89E-01 | 9.72E-01 | -0.035388 | -6.0734 | -0.03287 |
| hsa-miR-1306-3p | 9.89E-01 | 9.72E-01 | -0.035388 | -6.0734 | -0.03287 |
| hsa-miR-1306-5p | 9.89E-01 | 9.72E-01 | -0.035388 | -6.0734 | -0.03287 |
| hsa-miR-130a-5p | 9.89E-01 | 9.72E-01 | -0.035388 | -6.0734 | -0.03287 |
| hsa-miR-130b-5p | 9.89E-01 | 9.72E-01 | -0.035388 | -6.0734 | -0.03287 |
| hsa-miR-1322 | 9.89E-01 | 9.72E-01 | -0.035388 | -6.0734 | -0.03287 |
| hsa-miR-1323 | 9.89E-01 | 9.72E-01 | -0.035388 | -6.0734 | -0.03287 |
| hsa-miR-1324 | 9.89E-01 | 9.72E-01 | -0.035388 | -6.0734 | -0.03287 |
| hsa-miR-133a-3p | 9.89E-01 | 9.72E-01 | -0.035388 | -6.0734 | -0.03287 |
| hsa-miR-133a-5p | 9.89E-01 | 9.72E-01 | -0.035388 | -6.0734 | -0.03287 |
| hsa-miR-134-3p | 9.89E-01 | 9.72E-01 | -0.035388 | -6.0734 | -0.03287 |
| hsa-miR-1343-3p | 9.89E-01 | 9.72E-01 | -0.035388 | -6.0734 | -0.03287 |
| hsa-miR-135a-5p | 9.89E-01 | 9.72E-01 | -0.035388 | -6.0734 | -0.03287 |
| hsa-miR-135b-3p | 9.89E-01 | 9.72E-01 | -0.035388 | -6.0734 | -0.03287 |
| hsa-miR-136-3p | 9.89E-01 | 9.72E-01 | -0.035388 | -6.0734 | -0.03287 |
| hsa-miR-137 | 9.89E-01 | 9.72E-01 | -0.035388 | -6.0734 | -0.03287 |
| hsa-miR-138-1-3p | 9.89E-01 | 9.72E-01 | -0.035388 | -6.0734 | -0.03287 |
| hsa-miR-138-2-3p | 9.89E-01 | 9.72E-01 | -0.035388 | -6.0734 | -0.03287 |
| hsa-miR-138-5p | 9.89E-01 | 9.72E-01 | -0.035388 | -6.0734 | -0.03287 |
| hsa-miR-139-5p | 9.89E-01 | 9.72E-01 | -0.035388 | -6.0734 | -0.03287 |
| hsa-miR-144-5p | 9.89E-01 | 9.72E-01 | -0.035388 | -6.0734 | -0.03287 |
| hsa-miR-1468-3p | 9.89E-01 | 9.72E-01 | -0.035388 | -6.0734 | -0.03287 |
| hsa-miR-1468-5p | 9.89E-01 | 9.72E-01 | -0.035388 | -6.0734 | -0.03287 |
| hsa-miR-146a-3p | 9.89E-01 | 9.72E-01 | -0.035388 | -6.0734 | -0.03287 |
| hsa-miR-146b-3p | 9.89E-01 | 9.72E-01 | -0.035388 | -6.0734 | -0.03287 |
| hsa-miR-1470 | 9.89E-01 | 9.72E-01 | -0.035388 | -6.0734 | -0.03287 |
| hsa-miR-147a | 9.89E-01 | 9.72E-01 | -0.035388 | -6.0734 | -0.03287 |
| hsa-miR-147b | 9.89E-01 | 9.72E-01 | -0.035388 | -6.0734 | -0.03287 |
| hsa-miR-148b-5p | 9.89E-01 | 9.72E-01 | -0.035388 | -6.0734 | -0.03287 |
| hsa-miR-152-5p | 9.89E-01 | 9.72E-01 | -0.035388 | -6.0734 | -0.03287 |
| hsa-miR-153-5p | 9.89E-01 | 9.72E-01 | -0.035388 | -6.0734 | -0.03287 |
| hsa-miR-1537-3p | 9.89E-01 | 9.72E-01 | -0.035388 | -6.0734 | -0.03287 |
| hsa-miR-1537-5p | 9.89E-01 | 9.72E-01 | -0.035388 | -6.0734 | -0.03287 |
| hsa-miR-1538 | 9.89E-01 | 9.72E-01 | -0.035388 | -6.0734 | -0.03287 |
| hsa-miR-1539 | 9.89E-01 | 9.72E-01 | -0.035388 | -6.0734 | -0.03287 |
| hsa-miR-154-3p | 9.89E-01 | 9.72E-01 | -0.035388 | -6.0734 | -0.03287 |
| hsa-miR-155-3p | 9.89E-01 | 9.72E-01 | -0.035388 | -6.0734 | -0.03287 |
| hsa-miR-15a-3p | 9.89E-01 | 9.72E-01 | -0.035388 | -6.0734 | -0.03287 |
| hsa-miR-16-1-3p | 9.89E-01 | 9.72E-01 | -0.035388 | -6.0734 | -0.03287 |
| hsa-miR-181b-2-3p | 9.89E-01 | 9.72E-01 | -0.035388 | -6.0734 | -0.03287 |
| hsa-miR-181b-3p | 9.89E-01 | 9.72E-01 | -0.035388 | -6.0734 | -0.03287 |
| hsa-miR-181d-3p | 9.89E-01 | 9.72E-01 | -0.035388 | -6.0734 | -0.03287 |
| hsa-miR-182-3p | 9.89E-01 | 9.72E-01 | -0.035388 | -6.0734 | -0.03287 |
| hsa-miR-1825 | 9.89E-01 | 9.72E-01 | -0.035388 | -6.0734 | -0.03287 |
| hsa-miR-1827 | 9.89E-01 | 9.72E-01 | -0.035388 | -6.0734 | -0.03287 |
| hsa-miR-184 | 9.89E-01 | 9.72E-01 | -0.035388 | -6.0734 | -0.03287 |
| hsa-miR-185-3p | 9.89E-01 | 9.72E-01 | -0.035388 | -6.0734 | -0.03287 |
| hsa-miR-186-3p | 9.89E-01 | 9.72E-01 | -0.035388 | -6.0734 | -0.03287 |
| hsa-miR-187-3p | 9.89E-01 | 9.72E-01 | -0.035388 | -6.0734 | -0.03287 |
| hsa-miR-188-3p | 9.89E-01 | 9.72E-01 | -0.035388 | -6.0734 | -0.03287 |
| hsa-miR-18a-3p | 9.89E-01 | 9.72E-01 | -0.035388 | -6.0734 | -0.03287 |
| hsa-miR-18b-3p | 9.89E-01 | 9.72E-01 | -0.035388 | -6.0734 | -0.03287 |
| hsa-miR-1908-5p | 9.89E-01 | 9.72E-01 | -0.035388 | -6.0734 | -0.03287 |
| hsa-miR-1909-3p | 9.89E-01 | 9.72E-01 | -0.035388 | -6.0734 | -0.03287 |
| hsa-miR-190a-3p | 9.89E-01 | 9.72E-01 | -0.035388 | -6.0734 | -0.03287 |
| hsa-miR-190a-5p | 9.89E-01 | 9.72E-01 | -0.035388 | -6.0734 | -0.03287 |
| hsa-miR-190b | 9.89E-01 | 9.72E-01 | -0.035388 | -6.0734 | -0.03287 |
| hsa-miR-1910-5p | 9.89E-01 | 9.72E-01 | -0.035388 | -6.0734 | -0.03287 |
| hsa-miR-1911-3p | 9.89E-01 | 9.72E-01 | -0.035388 | -6.0734 | -0.03287 |
| hsa-miR-1911-5p | 9.89E-01 | 9.72E-01 | -0.035388 | -6.0734 | -0.03287 |
| hsa-miR-1912 | 9.89E-01 | 9.72E-01 | -0.035388 | -6.0734 | -0.03287 |
| hsa-miR-1913 | 9.89E-01 | 9.72E-01 | -0.035388 | -6.0734 | -0.03287 |
| hsa-miR-1914-5p | 9.89E-01 | 9.72E-01 | -0.035388 | -6.0734 | -0.03287 |
| hsa-miR-1915-5p | 9.89E-01 | 9.72E-01 | -0.035388 | -6.0734 | -0.03287 |
| hsa-miR-195-3p | 9.89E-01 | 9.72E-01 | -0.035388 | -6.0734 | -0.03287 |
| hsa-miR-196a-3p | 9.89E-01 | 9.72E-01 | -0.035388 | -6.0734 | -0.03287 |
| hsa-miR-1976 | 9.89E-01 | 9.72E-01 | -0.035388 | -6.0734 | -0.03287 |
| hsa-miR-19a-5p | 9.89E-01 | 9.72E-01 | -0.035388 | -6.0734 | -0.03287 |
| hsa-miR-19b-2-5p | 9.89E-01 | 9.72E-01 | -0.035388 | -6.0734 | -0.03287 |
| hsa-miR-200c-5p | 9.89E-01 | 9.72E-01 | -0.035388 | -6.0734 | -0.03287 |
| hsa-miR-202-5p | 9.89E-01 | 9.72E-01 | -0.035388 | -6.0734 | -0.03287 |
| hsa-miR-203a-5p | 9.89E-01 | 9.72E-01 | -0.035388 | -6.0734 | -0.03287 |
| hsa-miR-203b-3p | 9.89E-01 | 9.72E-01 | -0.035388 | -6.0734 | -0.03287 |
| hsa-miR-203b-5p | 9.89E-01 | 9.72E-01 | -0.035388 | -6.0734 | -0.03287 |
| hsa-miR-204-3p | 9.89E-01 | 9.72E-01 | -0.035388 | -6.0734 | -0.03287 |
| hsa-miR-205-3p | 9.89E-01 | 9.72E-01 | -0.035388 | -6.0734 | -0.03287 |
| hsa-miR-205-5p | 9.89E-01 | 9.72E-01 | -0.035388 | -6.0734 | -0.03287 |
| hsa-miR-2052 | 9.89E-01 | 9.72E-01 | -0.035388 | -6.0734 | -0.03287 |
| hsa-miR-2053 | 9.89E-01 | 9.72E-01 | -0.035388 | -6.0734 | -0.03287 |
| hsa-miR-206 | 9.89E-01 | 9.72E-01 | -0.035388 | -6.0734 | -0.03287 |
| hsa-miR-208a-3p | 9.89E-01 | 9.72E-01 | -0.035388 | -6.0734 | -0.03287 |
| hsa-miR-208b-3p | 9.89E-01 | 9.72E-01 | -0.035388 | -6.0734 | -0.03287 |
| hsa-miR-208b-5p | 9.89E-01 | 9.72E-01 | -0.035388 | -6.0734 | -0.03287 |
| hsa-miR-20b-3p | 9.89E-01 | 9.72E-01 | -0.035388 | -6.0734 | -0.03287 |
| hsa-miR-210-5p | 9.89E-01 | 9.72E-01 | -0.035388 | -6.0734 | -0.03287 |
| hsa-miR-211-5p | 9.89E-01 | 9.72E-01 | -0.035388 | -6.0734 | -0.03287 |
| hsa-miR-2110 | 9.89E-01 | 9.72E-01 | -0.035388 | -6.0734 | -0.03287 |
| hsa-miR-2113 | 9.89E-01 | 9.72E-01 | -0.035388 | -6.0734 | -0.03287 |
| hsa-miR-2114-3p | 9.89E-01 | 9.72E-01 | -0.035388 | -6.0734 | -0.03287 |
| hsa-miR-2114-5p | 9.89E-01 | 9.72E-01 | -0.035388 | -6.0734 | -0.03287 |
| hsa-miR-2115-3p | 9.89E-01 | 9.72E-01 | -0.035388 | -6.0734 | -0.03287 |
| hsa-miR-2115-5p | 9.89E-01 | 9.72E-01 | -0.035388 | -6.0734 | -0.03287 |
| hsa-miR-2116-3p | 9.89E-01 | 9.72E-01 | -0.035388 | -6.0734 | -0.03287 |
| hsa-miR-2116-5p | 9.89E-01 | 9.72E-01 | -0.035388 | -6.0734 | -0.03287 |
| hsa-miR-2117 | 9.89E-01 | 9.72E-01 | -0.035388 | -6.0734 | -0.03287 |
| hsa-miR-212-5p | 9.89E-01 | 9.72E-01 | -0.035388 | -6.0734 | -0.03287 |
| hsa-miR-215-3p | 9.89E-01 | 9.72E-01 | -0.035388 | -6.0734 | -0.03287 |
| hsa-miR-216a-3p | 9.89E-01 | 9.72E-01 | -0.035388 | -6.0734 | -0.03287 |
| hsa-miR-216a-5p | 9.89E-01 | 9.72E-01 | -0.035388 | -6.0734 | -0.03287 |
| hsa-miR-216b-3p | 9.89E-01 | 9.72E-01 | -0.035388 | -6.0734 | -0.03287 |
| hsa-miR-216b-5p | 9.89E-01 | 9.72E-01 | -0.035388 | -6.0734 | -0.03287 |
| hsa-miR-217 | 9.89E-01 | 9.72E-01 | -0.035388 | -6.0734 | -0.03287 |
| hsa-miR-218-1-3p | 9.89E-01 | 9.72E-01 | -0.035388 | -6.0734 | -0.03287 |
| hsa-miR-218-2-3p | 9.89E-01 | 9.72E-01 | -0.035388 | -6.0734 | -0.03287 |
| hsa-miR-219a-1-3p | 9.89E-01 | 9.72E-01 | -0.035388 | -6.0734 | -0.03287 |
| hsa-miR-219a-2-3p | 9.89E-01 | 9.72E-01 | -0.035388 | -6.0734 | -0.03287 |
| hsa-miR-219a-5p | 9.89E-01 | 9.72E-01 | -0.035388 | -6.0734 | -0.03287 |
| hsa-miR-219b-3p | 9.89E-01 | 9.72E-01 | -0.035388 | -6.0734 | -0.03287 |
| hsa-miR-219b-5p | 9.89E-01 | 9.72E-01 | -0.035388 | -6.0734 | -0.03287 |
| hsa-miR-222-5p | 9.89E-01 | 9.72E-01 | -0.035388 | -6.0734 | -0.03287 |
| hsa-miR-223-5p | 9.89E-01 | 9.72E-01 | -0.035388 | -6.0734 | -0.03287 |
| hsa-miR-2276-5p | 9.89E-01 | 9.72E-01 | -0.035388 | -6.0734 | -0.03287 |
| hsa-miR-2277-3p | 9.89E-01 | 9.72E-01 | -0.035388 | -6.0734 | -0.03287 |
| hsa-miR-2277-5p | 9.89E-01 | 9.72E-01 | -0.035388 | -6.0734 | -0.03287 |
| hsa-miR-2278 | 9.89E-01 | 9.72E-01 | -0.035388 | -6.0734 | -0.03287 |
| hsa-miR-2355-3p | 9.89E-01 | 9.72E-01 | -0.035388 | -6.0734 | -0.03287 |
| hsa-miR-2355-5p | 9.89E-01 | 9.72E-01 | -0.035388 | -6.0734 | -0.03287 |
| hsa-miR-23c | 9.89E-01 | 9.72E-01 | -0.035388 | -6.0734 | -0.03287 |
| hsa-miR-24-2-5p | 9.89E-01 | 9.72E-01 | -0.035388 | -6.0734 | -0.03287 |
| hsa-miR-2467-5p | 9.89E-01 | 9.72E-01 | -0.035388 | -6.0734 | -0.03287 |
| hsa-miR-25-5p | 9.89E-01 | 9.72E-01 | -0.035388 | -6.0734 | -0.03287 |
| hsa-miR-2681-3p | 9.89E-01 | 9.72E-01 | -0.035388 | -6.0734 | -0.03287 |
| hsa-miR-2681-5p | 9.89E-01 | 9.72E-01 | -0.035388 | -6.0734 | -0.03287 |
| hsa-miR-2682-3p | 9.89E-01 | 9.72E-01 | -0.035388 | -6.0734 | -0.03287 |
| hsa-miR-2682-5p | 9.89E-01 | 9.72E-01 | -0.035388 | -6.0734 | -0.03287 |
| hsa-miR-26a-1-3p | 9.89E-01 | 9.72E-01 | -0.035388 | -6.0734 | -0.03287 |
| hsa-miR-26a-2-3p | 9.89E-01 | 9.72E-01 | -0.035388 | -6.0734 | -0.03287 |
| hsa-miR-26b-3p | 9.89E-01 | 9.72E-01 | -0.035388 | -6.0734 | -0.03287 |
| hsa-miR-27a-5p | 9.89E-01 | 9.72E-01 | -0.035388 | -6.0734 | -0.03287 |
| hsa-miR-27b-5p | 9.89E-01 | 9.72E-01 | -0.035388 | -6.0734 | -0.03287 |
| hsa-miR-2909 | 9.89E-01 | 9.72E-01 | -0.035388 | -6.0734 | -0.03287 |
| hsa-miR-296-3p | 9.89E-01 | 9.72E-01 | -0.035388 | -6.0734 | -0.03287 |
| hsa-miR-297 | 9.89E-01 | 9.72E-01 | -0.035388 | -6.0734 | -0.03287 |
| hsa-miR-298 | 9.89E-01 | 9.72E-01 | -0.035388 | -6.0734 | -0.03287 |
| hsa-miR-299-3p | 9.89E-01 | 9.72E-01 | -0.035388 | -6.0734 | -0.03287 |
| hsa-miR-29a-5p | 9.89E-01 | 9.72E-01 | -0.035388 | -6.0734 | -0.03287 |
| hsa-miR-29b-2-5p | 9.89E-01 | 9.72E-01 | -0.035388 | -6.0734 | -0.03287 |
| hsa-miR-300 | 9.89E-01 | 9.72E-01 | -0.035388 | -6.0734 | -0.03287 |
| hsa-miR-301a-5p | 9.89E-01 | 9.72E-01 | -0.035388 | -6.0734 | -0.03287 |
| hsa-miR-301b-5p | 9.89E-01 | 9.72E-01 | -0.035388 | -6.0734 | -0.03287 |
| hsa-miR-302a-3p | 9.89E-01 | 9.72E-01 | -0.035388 | -6.0734 | -0.03287 |
| hsa-miR-302a-5p | 9.89E-01 | 9.72E-01 | -0.035388 | -6.0734 | -0.03287 |
| hsa-miR-302b-3p | 9.89E-01 | 9.72E-01 | -0.035388 | -6.0734 | -0.03287 |
| hsa-miR-302b-5p | 9.89E-01 | 9.72E-01 | -0.035388 | -6.0734 | -0.03287 |
| hsa-miR-302c-3p | 9.89E-01 | 9.72E-01 | -0.035388 | -6.0734 | -0.03287 |
| hsa-miR-302c-5p | 9.89E-01 | 9.72E-01 | -0.035388 | -6.0734 | -0.03287 |
| hsa-miR-302d-3p | 9.89E-01 | 9.72E-01 | -0.035388 | -6.0734 | -0.03287 |
| hsa-miR-302d-5p | 9.89E-01 | 9.72E-01 | -0.035388 | -6.0734 | -0.03287 |
| hsa-miR-302e | 9.89E-01 | 9.72E-01 | -0.035388 | -6.0734 | -0.03287 |
| hsa-miR-302f | 9.89E-01 | 9.72E-01 | -0.035388 | -6.0734 | -0.03287 |
| hsa-miR-3064-3p | 9.89E-01 | 9.72E-01 | -0.035388 | -6.0734 | -0.03287 |
| hsa-miR-3064-5p | 9.89E-01 | 9.72E-01 | -0.035388 | -6.0734 | -0.03287 |
| hsa-miR-3065-3p | 9.89E-01 | 9.72E-01 | -0.035388 | -6.0734 | -0.03287 |
| hsa-miR-3065-5p | 9.89E-01 | 9.72E-01 | -0.035388 | -6.0734 | -0.03287 |
| hsa-miR-3074-3p | 9.89E-01 | 9.72E-01 | -0.035388 | -6.0734 | -0.03287 |
| hsa-miR-3074-5p | 9.89E-01 | 9.72E-01 | -0.035388 | -6.0734 | -0.03287 |
| hsa-miR-30d-3p | 9.89E-01 | 9.72E-01 | -0.035388 | -6.0734 | -0.03287 |
| hsa-miR-3115 | 9.89E-01 | 9.72E-01 | -0.035388 | -6.0734 | -0.03287 |
| hsa-miR-3116 | 9.89E-01 | 9.72E-01 | -0.035388 | -6.0734 | -0.03287 |
| hsa-miR-3117-3p | 9.89E-01 | 9.72E-01 | -0.035388 | -6.0734 | -0.03287 |
| hsa-miR-3117-5p | 9.89E-01 | 9.72E-01 | -0.035388 | -6.0734 | -0.03287 |
| hsa-miR-3118 | 9.89E-01 | 9.72E-01 | -0.035388 | -6.0734 | -0.03287 |
| hsa-miR-3119 | 9.89E-01 | 9.72E-01 | -0.035388 | -6.0734 | -0.03287 |
| hsa-miR-3120-3p | 9.89E-01 | 9.72E-01 | -0.035388 | -6.0734 | -0.03287 |
| hsa-miR-3120-5p | 9.89E-01 | 9.72E-01 | -0.035388 | -6.0734 | -0.03287 |
| hsa-miR-3121-3p | 9.89E-01 | 9.72E-01 | -0.035388 | -6.0734 | -0.03287 |
| hsa-miR-3121-5p | 9.89E-01 | 9.72E-01 | -0.035388 | -6.0734 | -0.03287 |
| hsa-miR-3122 | 9.89E-01 | 9.72E-01 | -0.035388 | -6.0734 | -0.03287 |
| hsa-miR-3123 | 9.89E-01 | 9.72E-01 | -0.035388 | -6.0734 | -0.03287 |
| hsa-miR-3124-3p | 9.89E-01 | 9.72E-01 | -0.035388 | -6.0734 | -0.03287 |
| hsa-miR-3124-5p | 9.89E-01 | 9.72E-01 | -0.035388 | -6.0734 | -0.03287 |
| hsa-miR-3126-3p | 9.89E-01 | 9.72E-01 | -0.035388 | -6.0734 | -0.03287 |
| hsa-miR-3126-5p | 9.89E-01 | 9.72E-01 | -0.035388 | -6.0734 | -0.03287 |
| hsa-miR-3127-3p | 9.89E-01 | 9.72E-01 | -0.035388 | -6.0734 | -0.03287 |
| hsa-miR-3128 | 9.89E-01 | 9.72E-01 | -0.035388 | -6.0734 | -0.03287 |
| hsa-miR-3129-3p | 9.89E-01 | 9.72E-01 | -0.035388 | -6.0734 | -0.03287 |
| hsa-miR-3129-5p | 9.89E-01 | 9.72E-01 | -0.035388 | -6.0734 | -0.03287 |
| hsa-miR-3130-3p | 9.89E-01 | 9.72E-01 | -0.035388 | -6.0734 | -0.03287 |
| hsa-miR-3130-5p | 9.89E-01 | 9.72E-01 | -0.035388 | -6.0734 | -0.03287 |
| hsa-miR-3133 | 9.89E-01 | 9.72E-01 | -0.035388 | -6.0734 | -0.03287 |
| hsa-miR-3134 | 9.89E-01 | 9.72E-01 | -0.035388 | -6.0734 | -0.03287 |
| hsa-miR-3135a | 9.89E-01 | 9.72E-01 | -0.035388 | -6.0734 | -0.03287 |
| hsa-miR-3136-3p | 9.89E-01 | 9.72E-01 | -0.035388 | -6.0734 | -0.03287 |
| hsa-miR-3136-5p | 9.89E-01 | 9.72E-01 | -0.035388 | -6.0734 | -0.03287 |
| hsa-miR-3139 | 9.89E-01 | 9.72E-01 | -0.035388 | -6.0734 | -0.03287 |
| hsa-miR-3140-3p | 9.89E-01 | 9.72E-01 | -0.035388 | -6.0734 | -0.03287 |
| hsa-miR-3140-5p | 9.89E-01 | 9.72E-01 | -0.035388 | -6.0734 | -0.03287 |
| hsa-miR-3142 | 9.89E-01 | 9.72E-01 | -0.035388 | -6.0734 | -0.03287 |
| hsa-miR-3143 | 9.89E-01 | 9.72E-01 | -0.035388 | -6.0734 | -0.03287 |
| hsa-miR-3144-3p | 9.89E-01 | 9.72E-01 | -0.035388 | -6.0734 | -0.03287 |
| hsa-miR-3144-5p | 9.89E-01 | 9.72E-01 | -0.035388 | -6.0734 | -0.03287 |
| hsa-miR-3145-3p | 9.89E-01 | 9.72E-01 | -0.035388 | -6.0734 | -0.03287 |
| hsa-miR-3145-5p | 9.89E-01 | 9.72E-01 | -0.035388 | -6.0734 | -0.03287 |
| hsa-miR-3146 | 9.89E-01 | 9.72E-01 | -0.035388 | -6.0734 | -0.03287 |
| hsa-miR-3148 | 9.89E-01 | 9.72E-01 | -0.035388 | -6.0734 | -0.03287 |
| hsa-miR-3150a-3p | 9.89E-01 | 9.72E-01 | -0.035388 | -6.0734 | -0.03287 |
| hsa-miR-3150a-5p | 9.89E-01 | 9.72E-01 | -0.035388 | -6.0734 | -0.03287 |
| hsa-miR-3150b-3p | 9.89E-01 | 9.72E-01 | -0.035388 | -6.0734 | -0.03287 |
| hsa-miR-3150b-5p | 9.89E-01 | 9.72E-01 | -0.035388 | -6.0734 | -0.03287 |
| hsa-miR-3151-5p | 9.89E-01 | 9.72E-01 | -0.035388 | -6.0734 | -0.03287 |
| hsa-miR-3152-3p | 9.89E-01 | 9.72E-01 | -0.035388 | -6.0734 | -0.03287 |
| hsa-miR-3152-5p | 9.89E-01 | 9.72E-01 | -0.035388 | -6.0734 | -0.03287 |
| hsa-miR-3153 | 9.89E-01 | 9.72E-01 | -0.035388 | -6.0734 | -0.03287 |
| hsa-miR-3155a | 9.89E-01 | 9.72E-01 | -0.035388 | -6.0734 | -0.03287 |
| hsa-miR-3155b | 9.89E-01 | 9.72E-01 | -0.035388 | -6.0734 | -0.03287 |
| hsa-miR-3156-3p | 9.89E-01 | 9.72E-01 | -0.035388 | -6.0734 | -0.03287 |
| hsa-miR-3157-3p | 9.89E-01 | 9.72E-01 | -0.035388 | -6.0734 | -0.03287 |
| hsa-miR-3157-5p | 9.89E-01 | 9.72E-01 | -0.035388 | -6.0734 | -0.03287 |
| hsa-miR-3158-3p | 9.89E-01 | 9.72E-01 | -0.035388 | -6.0734 | -0.03287 |
| hsa-miR-3159 | 9.89E-01 | 9.72E-01 | -0.035388 | -6.0734 | -0.03287 |
| hsa-miR-3160-3p | 9.89E-01 | 9.72E-01 | -0.035388 | -6.0734 | -0.03287 |
| hsa-miR-3160-5p | 9.89E-01 | 9.72E-01 | -0.035388 | -6.0734 | -0.03287 |
| hsa-miR-3163 | 9.89E-01 | 9.72E-01 | -0.035388 | -6.0734 | -0.03287 |
| hsa-miR-3164 | 9.89E-01 | 9.72E-01 | -0.035388 | -6.0734 | -0.03287 |
| hsa-miR-3165 | 9.89E-01 | 9.72E-01 | -0.035388 | -6.0734 | -0.03287 |
| hsa-miR-3166 | 9.89E-01 | 9.72E-01 | -0.035388 | -6.0734 | -0.03287 |
| hsa-miR-3167 | 9.89E-01 | 9.72E-01 | -0.035388 | -6.0734 | -0.03287 |
| hsa-miR-3168 | 9.89E-01 | 9.72E-01 | -0.035388 | -6.0734 | -0.03287 |
| hsa-miR-3169 | 9.89E-01 | 9.72E-01 | -0.035388 | -6.0734 | -0.03287 |
| hsa-miR-3170 | 9.89E-01 | 9.72E-01 | -0.035388 | -6.0734 | -0.03287 |
| hsa-miR-3171 | 9.89E-01 | 9.72E-01 | -0.035388 | -6.0734 | -0.03287 |
| hsa-miR-3173-5p | 9.89E-01 | 9.72E-01 | -0.035388 | -6.0734 | -0.03287 |
| hsa-miR-3175 | 9.89E-01 | 9.72E-01 | -0.035388 | -6.0734 | -0.03287 |
| hsa-miR-3176 | 9.89E-01 | 9.72E-01 | -0.035388 | -6.0734 | -0.03287 |
| hsa-miR-3177-3p | 9.89E-01 | 9.72E-01 | -0.035388 | -6.0734 | -0.03287 |
| hsa-miR-3177-5p | 9.89E-01 | 9.72E-01 | -0.035388 | -6.0734 | -0.03287 |
| hsa-miR-3178 | 9.89E-01 | 9.72E-01 | -0.035388 | -6.0734 | -0.03287 |
| hsa-miR-3179 | 9.89E-01 | 9.72E-01 | -0.035388 | -6.0734 | -0.03287 |
| hsa-miR-3180 | 9.89E-01 | 9.72E-01 | -0.035388 | -6.0734 | -0.03287 |
| hsa-miR-3180-5p | 9.89E-01 | 9.72E-01 | -0.035388 | -6.0734 | -0.03287 |
| hsa-miR-3181 | 9.89E-01 | 9.72E-01 | -0.035388 | -6.0734 | -0.03287 |
| hsa-miR-3182 | 9.89E-01 | 9.72E-01 | -0.035388 | -6.0734 | -0.03287 |
| hsa-miR-3183 | 9.89E-01 | 9.72E-01 | -0.035388 | -6.0734 | -0.03287 |
| hsa-miR-3184-3p | 9.89E-01 | 9.72E-01 | -0.035388 | -6.0734 | -0.03287 |
| hsa-miR-3184-5p | 9.89E-01 | 9.72E-01 | -0.035388 | -6.0734 | -0.03287 |
| hsa-miR-3186-3p | 9.89E-01 | 9.72E-01 | -0.035388 | -6.0734 | -0.03287 |
| hsa-miR-3186-5p | 9.89E-01 | 9.72E-01 | -0.035388 | -6.0734 | -0.03287 |
| hsa-miR-3187-3p | 9.89E-01 | 9.72E-01 | -0.035388 | -6.0734 | -0.03287 |
| hsa-miR-3187-5p | 9.89E-01 | 9.72E-01 | -0.035388 | -6.0734 | -0.03287 |
| hsa-miR-3189-3p | 9.89E-01 | 9.72E-01 | -0.035388 | -6.0734 | -0.03287 |
| hsa-miR-3189-5p | 9.89E-01 | 9.72E-01 | -0.035388 | -6.0734 | -0.03287 |
| hsa-miR-3190-3p | 9.89E-01 | 9.72E-01 | -0.035388 | -6.0734 | -0.03287 |
| hsa-miR-3190-5p | 9.89E-01 | 9.72E-01 | -0.035388 | -6.0734 | -0.03287 |
| hsa-miR-3191-3p | 9.89E-01 | 9.72E-01 | -0.035388 | -6.0734 | -0.03287 |
| hsa-miR-3191-5p | 9.89E-01 | 9.72E-01 | -0.035388 | -6.0734 | -0.03287 |
| hsa-miR-3192-3p | 9.89E-01 | 9.72E-01 | -0.035388 | -6.0734 | -0.03287 |
| hsa-miR-3192-5p | 9.89E-01 | 9.72E-01 | -0.035388 | -6.0734 | -0.03287 |
| hsa-miR-3193 | 9.89E-01 | 9.72E-01 | -0.035388 | -6.0734 | -0.03287 |
| hsa-miR-3194-3p | 9.89E-01 | 9.72E-01 | -0.035388 | -6.0734 | -0.03287 |
| hsa-miR-3197 | 9.89E-01 | 9.72E-01 | -0.035388 | -6.0734 | -0.03287 |
| hsa-miR-3199 | 9.89E-01 | 9.72E-01 | -0.035388 | -6.0734 | -0.03287 |
| hsa-miR-3200-3p | 9.89E-01 | 9.72E-01 | -0.035388 | -6.0734 | -0.03287 |
| hsa-miR-3200-5p | 9.89E-01 | 9.72E-01 | -0.035388 | -6.0734 | -0.03287 |
| hsa-miR-3201 | 9.89E-01 | 9.72E-01 | -0.035388 | -6.0734 | -0.03287 |
| hsa-miR-323a-3p | 9.89E-01 | 9.72E-01 | -0.035388 | -6.0734 | -0.03287 |
| hsa-miR-323a-5p | 9.89E-01 | 9.72E-01 | -0.035388 | -6.0734 | -0.03287 |
| hsa-miR-323b-3p | 9.89E-01 | 9.72E-01 | -0.035388 | -6.0734 | -0.03287 |
| hsa-miR-323b-5p | 9.89E-01 | 9.72E-01 | -0.035388 | -6.0734 | -0.03287 |
| hsa-miR-325 | 9.89E-01 | 9.72E-01 | -0.035388 | -6.0734 | -0.03287 |
| hsa-miR-328-3p | 9.89E-01 | 9.72E-01 | -0.035388 | -6.0734 | -0.03287 |
| hsa-miR-329-3p | 9.89E-01 | 9.72E-01 | -0.035388 | -6.0734 | -0.03287 |
| hsa-miR-329-5p | 9.89E-01 | 9.72E-01 | -0.035388 | -6.0734 | -0.03287 |
| hsa-miR-330-5p | 9.89E-01 | 9.72E-01 | -0.035388 | -6.0734 | -0.03287 |
| hsa-miR-331-5p | 9.89E-01 | 9.72E-01 | -0.035388 | -6.0734 | -0.03287 |
| hsa-miR-337-3p | 9.89E-01 | 9.72E-01 | -0.035388 | -6.0734 | -0.03287 |
| hsa-miR-338-5p | 9.89E-01 | 9.72E-01 | -0.035388 | -6.0734 | -0.03287 |
| hsa-miR-339-5p | 9.89E-01 | 9.72E-01 | -0.035388 | -6.0734 | -0.03287 |
| hsa-miR-33a-3p | 9.89E-01 | 9.72E-01 | -0.035388 | -6.0734 | -0.03287 |
| hsa-miR-33b-5p | 9.89E-01 | 9.72E-01 | -0.035388 | -6.0734 | -0.03287 |
| hsa-miR-346 | 9.89E-01 | 9.72E-01 | -0.035388 | -6.0734 | -0.03287 |
| hsa-miR-34b-3p | 9.89E-01 | 9.72E-01 | -0.035388 | -6.0734 | -0.03287 |
| hsa-miR-34c-3p | 9.89E-01 | 9.72E-01 | -0.035388 | -6.0734 | -0.03287 |
| hsa-miR-34c-5p | 9.89E-01 | 9.72E-01 | -0.035388 | -6.0734 | -0.03287 |
| hsa-miR-3529-3p | 9.89E-01 | 9.72E-01 | -0.035388 | -6.0734 | -0.03287 |
| hsa-miR-3529-5p | 9.89E-01 | 9.72E-01 | -0.035388 | -6.0734 | -0.03287 |
| hsa-miR-3591-3p | 9.89E-01 | 9.72E-01 | -0.035388 | -6.0734 | -0.03287 |
| hsa-miR-3591-5p | 9.89E-01 | 9.72E-01 | -0.035388 | -6.0734 | -0.03287 |
| hsa-miR-3605-3p | 9.89E-01 | 9.72E-01 | -0.035388 | -6.0734 | -0.03287 |
| hsa-miR-3606-3p | 9.89E-01 | 9.72E-01 | -0.035388 | -6.0734 | -0.03287 |
| hsa-miR-3606-5p | 9.89E-01 | 9.72E-01 | -0.035388 | -6.0734 | -0.03287 |
| hsa-miR-3607-3p | 9.89E-01 | 9.72E-01 | -0.035388 | -6.0734 | -0.03287 |
| hsa-miR-3611 | 9.89E-01 | 9.72E-01 | -0.035388 | -6.0734 | -0.03287 |
| hsa-miR-3612 | 9.89E-01 | 9.72E-01 | -0.035388 | -6.0734 | -0.03287 |
| hsa-miR-3613-3p | 9.89E-01 | 9.72E-01 | -0.035388 | -6.0734 | -0.03287 |
| hsa-miR-3613-5p | 9.89E-01 | 9.72E-01 | -0.035388 | -6.0734 | -0.03287 |
| hsa-miR-3614-3p | 9.89E-01 | 9.72E-01 | -0.035388 | -6.0734 | -0.03287 |
| hsa-miR-3615 | 9.89E-01 | 9.72E-01 | -0.035388 | -6.0734 | -0.03287 |
| hsa-miR-3616-3p | 9.89E-01 | 9.72E-01 | -0.035388 | -6.0734 | -0.03287 |
| hsa-miR-3616-5p | 9.89E-01 | 9.72E-01 | -0.035388 | -6.0734 | -0.03287 |
| hsa-miR-3617-3p | 9.89E-01 | 9.72E-01 | -0.035388 | -6.0734 | -0.03287 |
| hsa-miR-3617-5p | 9.89E-01 | 9.72E-01 | -0.035388 | -6.0734 | -0.03287 |
| hsa-miR-3618 | 9.89E-01 | 9.72E-01 | -0.035388 | -6.0734 | -0.03287 |
| hsa-miR-3619-3p | 9.89E-01 | 9.72E-01 | -0.035388 | -6.0734 | -0.03287 |
| hsa-miR-3619-5p | 9.89E-01 | 9.72E-01 | -0.035388 | -6.0734 | -0.03287 |
| hsa-miR-3620-3p | 9.89E-01 | 9.72E-01 | -0.035388 | -6.0734 | -0.03287 |
| hsa-miR-3622a-3p | 9.89E-01 | 9.72E-01 | -0.035388 | -6.0734 | -0.03287 |
| hsa-miR-3622a-5p | 9.89E-01 | 9.72E-01 | -0.035388 | -6.0734 | -0.03287 |
| hsa-miR-3622b-3p | 9.89E-01 | 9.72E-01 | -0.035388 | -6.0734 | -0.03287 |
| hsa-miR-363-5p | 9.89E-01 | 9.72E-01 | -0.035388 | -6.0734 | -0.03287 |
| hsa-miR-3649 | 9.89E-01 | 9.72E-01 | -0.035388 | -6.0734 | -0.03287 |
| hsa-miR-3650 | 9.89E-01 | 9.72E-01 | -0.035388 | -6.0734 | -0.03287 |
| hsa-miR-3653-5p | 9.89E-01 | 9.72E-01 | -0.035388 | -6.0734 | -0.03287 |
| hsa-miR-3655 | 9.89E-01 | 9.72E-01 | -0.035388 | -6.0734 | -0.03287 |
| hsa-miR-3657 | 9.89E-01 | 9.72E-01 | -0.035388 | -6.0734 | -0.03287 |
| hsa-miR-3658 | 9.89E-01 | 9.72E-01 | -0.035388 | -6.0734 | -0.03287 |
| hsa-miR-3659 | 9.89E-01 | 9.72E-01 | -0.035388 | -6.0734 | -0.03287 |
| hsa-miR-3661 | 9.89E-01 | 9.72E-01 | -0.035388 | -6.0734 | -0.03287 |
| hsa-miR-3662 | 9.89E-01 | 9.72E-01 | -0.035388 | -6.0734 | -0.03287 |
| hsa-miR-3663-5p | 9.89E-01 | 9.72E-01 | -0.035388 | -6.0734 | -0.03287 |
| hsa-miR-3664-3p | 9.89E-01 | 9.72E-01 | -0.035388 | -6.0734 | -0.03287 |
| hsa-miR-3664-5p | 9.89E-01 | 9.72E-01 | -0.035388 | -6.0734 | -0.03287 |
| hsa-miR-3666 | 9.89E-01 | 9.72E-01 | -0.035388 | -6.0734 | -0.03287 |
| hsa-miR-3667-3p | 9.89E-01 | 9.72E-01 | -0.035388 | -6.0734 | -0.03287 |
| hsa-miR-3668 | 9.89E-01 | 9.72E-01 | -0.035388 | -6.0734 | -0.03287 |
| hsa-miR-367-3p | 9.89E-01 | 9.72E-01 | -0.035388 | -6.0734 | -0.03287 |
| hsa-miR-367-5p | 9.89E-01 | 9.72E-01 | -0.035388 | -6.0734 | -0.03287 |
| hsa-miR-3670 | 9.89E-01 | 9.72E-01 | -0.035388 | -6.0734 | -0.03287 |
| hsa-miR-3671 | 9.89E-01 | 9.72E-01 | -0.035388 | -6.0734 | -0.03287 |
| hsa-miR-3672 | 9.89E-01 | 9.72E-01 | -0.035388 | -6.0734 | -0.03287 |
| hsa-miR-3674 | 9.89E-01 | 9.72E-01 | -0.035388 | -6.0734 | -0.03287 |
| hsa-miR-3675-3p | 9.89E-01 | 9.72E-01 | -0.035388 | -6.0734 | -0.03287 |
| hsa-miR-3675-5p | 9.89E-01 | 9.72E-01 | -0.035388 | -6.0734 | -0.03287 |
| hsa-miR-3677-3p | 9.89E-01 | 9.72E-01 | -0.035388 | -6.0734 | -0.03287 |
| hsa-miR-3677-5p | 9.89E-01 | 9.72E-01 | -0.035388 | -6.0734 | -0.03287 |
| hsa-miR-3678-3p | 9.89E-01 | 9.72E-01 | -0.035388 | -6.0734 | -0.03287 |
| hsa-miR-3678-5p | 9.89E-01 | 9.72E-01 | -0.035388 | -6.0734 | -0.03287 |
| hsa-miR-3680-5p | 9.89E-01 | 9.72E-01 | -0.035388 | -6.0734 | -0.03287 |
| hsa-miR-3681-3p | 9.89E-01 | 9.72E-01 | -0.035388 | -6.0734 | -0.03287 |
| hsa-miR-3681-5p | 9.89E-01 | 9.72E-01 | -0.035388 | -6.0734 | -0.03287 |
| hsa-miR-3682-5p | 9.89E-01 | 9.72E-01 | -0.035388 | -6.0734 | -0.03287 |
| hsa-miR-3683 | 9.89E-01 | 9.72E-01 | -0.035388 | -6.0734 | -0.03287 |
| hsa-miR-3684 | 9.89E-01 | 9.72E-01 | -0.035388 | -6.0734 | -0.03287 |
| hsa-miR-3685 | 9.89E-01 | 9.72E-01 | -0.035388 | -6.0734 | -0.03287 |
| hsa-miR-3686 | 9.89E-01 | 9.72E-01 | -0.035388 | -6.0734 | -0.03287 |
| hsa-miR-3688-3p | 9.89E-01 | 9.72E-01 | -0.035388 | -6.0734 | -0.03287 |
| hsa-miR-3688-5p | 9.89E-01 | 9.72E-01 | -0.035388 | -6.0734 | -0.03287 |
| hsa-miR-3689a-3p | 9.89E-01 | 9.72E-01 | -0.035388 | -6.0734 | -0.03287 |
| hsa-miR-3689a-5p | 9.89E-01 | 9.72E-01 | -0.035388 | -6.0734 | -0.03287 |
| hsa-miR-3689b-3p | 9.89E-01 | 9.72E-01 | -0.035388 | -6.0734 | -0.03287 |
| hsa-miR-3689d | 9.89E-01 | 9.72E-01 | -0.035388 | -6.0734 | -0.03287 |
| hsa-miR-3689f | 9.89E-01 | 9.72E-01 | -0.035388 | -6.0734 | -0.03287 |
| hsa-miR-369-3p | 9.89E-01 | 9.72E-01 | -0.035388 | -6.0734 | -0.03287 |
| hsa-miR-3690 | 9.89E-01 | 9.72E-01 | -0.035388 | -6.0734 | -0.03287 |
| hsa-miR-3691-3p | 9.89E-01 | 9.72E-01 | -0.035388 | -6.0734 | -0.03287 |
| hsa-miR-3691-5p | 9.89E-01 | 9.72E-01 | -0.035388 | -6.0734 | -0.03287 |
| hsa-miR-3692-3p | 9.89E-01 | 9.72E-01 | -0.035388 | -6.0734 | -0.03287 |
| hsa-miR-3692-5p | 9.89E-01 | 9.72E-01 | -0.035388 | -6.0734 | -0.03287 |
| hsa-miR-370-5p | 9.89E-01 | 9.72E-01 | -0.035388 | -6.0734 | -0.03287 |
| hsa-miR-3713 | 9.89E-01 | 9.72E-01 | -0.035388 | -6.0734 | -0.03287 |
| hsa-miR-3714 | 9.89E-01 | 9.72E-01 | -0.035388 | -6.0734 | -0.03287 |
| hsa-miR-371a-3p | 9.89E-01 | 9.72E-01 | -0.035388 | -6.0734 | -0.03287 |
| hsa-miR-371b-3p | 9.89E-01 | 9.72E-01 | -0.035388 | -6.0734 | -0.03287 |
| hsa-miR-372-3p | 9.89E-01 | 9.72E-01 | -0.035388 | -6.0734 | -0.03287 |
| hsa-miR-372-5p | 9.89E-01 | 9.72E-01 | -0.035388 | -6.0734 | -0.03287 |
| hsa-miR-373-3p | 9.89E-01 | 9.72E-01 | -0.035388 | -6.0734 | -0.03287 |
| hsa-miR-374a-3p | 9.89E-01 | 9.72E-01 | -0.035388 | -6.0734 | -0.03287 |
| hsa-miR-374b-3p | 9.89E-01 | 9.72E-01 | -0.035388 | -6.0734 | -0.03287 |
| hsa-miR-374c-3p | 9.89E-01 | 9.72E-01 | -0.035388 | -6.0734 | -0.03287 |
| hsa-miR-376a-2-5p | 9.89E-01 | 9.72E-01 | -0.035388 | -6.0734 | -0.03287 |
| hsa-miR-376a-5p | 9.89E-01 | 9.72E-01 | -0.035388 | -6.0734 | -0.03287 |
| hsa-miR-376b-3p | 9.89E-01 | 9.72E-01 | -0.035388 | -6.0734 | -0.03287 |
| hsa-miR-376b-5p | 9.89E-01 | 9.72E-01 | -0.035388 | -6.0734 | -0.03287 |
| hsa-miR-376c-5p | 9.89E-01 | 9.72E-01 | -0.035388 | -6.0734 | -0.03287 |
| hsa-miR-377-5p | 9.89E-01 | 9.72E-01 | -0.035388 | -6.0734 | -0.03287 |
| hsa-miR-378e | 9.89E-01 | 9.72E-01 | -0.035388 | -6.0734 | -0.03287 |
| hsa-miR-378h | 9.89E-01 | 9.72E-01 | -0.035388 | -6.0734 | -0.03287 |
| hsa-miR-378j | 9.89E-01 | 9.72E-01 | -0.035388 | -6.0734 | -0.03287 |
| hsa-miR-379-3p | 9.89E-01 | 9.72E-01 | -0.035388 | -6.0734 | -0.03287 |
| hsa-miR-380-3p | 9.89E-01 | 9.72E-01 | -0.035388 | -6.0734 | -0.03287 |
| hsa-miR-380-5p | 9.89E-01 | 9.72E-01 | -0.035388 | -6.0734 | -0.03287 |
| hsa-miR-381-5p | 9.89E-01 | 9.72E-01 | -0.035388 | -6.0734 | -0.03287 |
| hsa-miR-382-3p | 9.89E-01 | 9.72E-01 | -0.035388 | -6.0734 | -0.03287 |
| hsa-miR-383-3p | 9.89E-01 | 9.72E-01 | -0.035388 | -6.0734 | -0.03287 |
| hsa-miR-383-5p | 9.89E-01 | 9.72E-01 | -0.035388 | -6.0734 | -0.03287 |
| hsa-miR-384 | 9.89E-01 | 9.72E-01 | -0.035388 | -6.0734 | -0.03287 |
| hsa-miR-3908 | 9.89E-01 | 9.72E-01 | -0.035388 | -6.0734 | -0.03287 |
| hsa-miR-3909 | 9.89E-01 | 9.72E-01 | -0.035388 | -6.0734 | -0.03287 |
| hsa-miR-3910 | 9.89E-01 | 9.72E-01 | -0.035388 | -6.0734 | -0.03287 |
| hsa-miR-3912-3p | 9.89E-01 | 9.72E-01 | -0.035388 | -6.0734 | -0.03287 |
| hsa-miR-3913-3p | 9.89E-01 | 9.72E-01 | -0.035388 | -6.0734 | -0.03287 |
| hsa-miR-3913-5p | 9.89E-01 | 9.72E-01 | -0.035388 | -6.0734 | -0.03287 |
| hsa-miR-3914 | 9.89E-01 | 9.72E-01 | -0.035388 | -6.0734 | -0.03287 |
| hsa-miR-3915 | 9.89E-01 | 9.72E-01 | -0.035388 | -6.0734 | -0.03287 |
| hsa-miR-3916 | 9.89E-01 | 9.72E-01 | -0.035388 | -6.0734 | -0.03287 |
| hsa-miR-3918 | 9.89E-01 | 9.72E-01 | -0.035388 | -6.0734 | -0.03287 |
| hsa-miR-3919 | 9.89E-01 | 9.72E-01 | -0.035388 | -6.0734 | -0.03287 |
| hsa-miR-3920 | 9.89E-01 | 9.72E-01 | -0.035388 | -6.0734 | -0.03287 |
| hsa-miR-3921 | 9.89E-01 | 9.72E-01 | -0.035388 | -6.0734 | -0.03287 |
| hsa-miR-3922-3p | 9.89E-01 | 9.72E-01 | -0.035388 | -6.0734 | -0.03287 |
| hsa-miR-3922-5p | 9.89E-01 | 9.72E-01 | -0.035388 | -6.0734 | -0.03287 |
| hsa-miR-3923 | 9.89E-01 | 9.72E-01 | -0.035388 | -6.0734 | -0.03287 |
| hsa-miR-3924 | 9.89E-01 | 9.72E-01 | -0.035388 | -6.0734 | -0.03287 |
| hsa-miR-3925-3p | 9.89E-01 | 9.72E-01 | -0.035388 | -6.0734 | -0.03287 |
| hsa-miR-3925-5p | 9.89E-01 | 9.72E-01 | -0.035388 | -6.0734 | -0.03287 |
| hsa-miR-3927-3p | 9.89E-01 | 9.72E-01 | -0.035388 | -6.0734 | -0.03287 |
| hsa-miR-3927-5p | 9.89E-01 | 9.72E-01 | -0.035388 | -6.0734 | -0.03287 |
| hsa-miR-3928-3p | 9.89E-01 | 9.72E-01 | -0.035388 | -6.0734 | -0.03287 |
| hsa-miR-3928-5p | 9.89E-01 | 9.72E-01 | -0.035388 | -6.0734 | -0.03287 |
| hsa-miR-3929 | 9.89E-01 | 9.72E-01 | -0.035388 | -6.0734 | -0.03287 |
| hsa-miR-3934-3p | 9.89E-01 | 9.72E-01 | -0.035388 | -6.0734 | -0.03287 |
| hsa-miR-3936 | 9.89E-01 | 9.72E-01 | -0.035388 | -6.0734 | -0.03287 |
| hsa-miR-3938 | 9.89E-01 | 9.72E-01 | -0.035388 | -6.0734 | -0.03287 |
| hsa-miR-3939 | 9.89E-01 | 9.72E-01 | -0.035388 | -6.0734 | -0.03287 |
| hsa-miR-3940-3p | 9.89E-01 | 9.72E-01 | -0.035388 | -6.0734 | -0.03287 |
| hsa-miR-3941 | 9.89E-01 | 9.72E-01 | -0.035388 | -6.0734 | -0.03287 |
| hsa-miR-3942-3p | 9.89E-01 | 9.72E-01 | -0.035388 | -6.0734 | -0.03287 |
| hsa-miR-3942-5p | 9.89E-01 | 9.72E-01 | -0.035388 | -6.0734 | -0.03287 |
| hsa-miR-3943 | 9.89E-01 | 9.72E-01 | -0.035388 | -6.0734 | -0.03287 |
| hsa-miR-3944-3p | 9.89E-01 | 9.72E-01 | -0.035388 | -6.0734 | -0.03287 |
| hsa-miR-3944-5p | 9.89E-01 | 9.72E-01 | -0.035388 | -6.0734 | -0.03287 |
| hsa-miR-3972 | 9.89E-01 | 9.72E-01 | -0.035388 | -6.0734 | -0.03287 |
| hsa-miR-3973 | 9.89E-01 | 9.72E-01 | -0.035388 | -6.0734 | -0.03287 |
| hsa-miR-3974 | 9.89E-01 | 9.72E-01 | -0.035388 | -6.0734 | -0.03287 |
| hsa-miR-3975 | 9.89E-01 | 9.72E-01 | -0.035388 | -6.0734 | -0.03287 |
| hsa-miR-3977 | 9.89E-01 | 9.72E-01 | -0.035388 | -6.0734 | -0.03287 |
| hsa-miR-3978 | 9.89E-01 | 9.72E-01 | -0.035388 | -6.0734 | -0.03287 |
| hsa-miR-409-5p | 9.89E-01 | 9.72E-01 | -0.035388 | -6.0734 | -0.03287 |
| hsa-miR-410-5p | 9.89E-01 | 9.72E-01 | -0.035388 | -6.0734 | -0.03287 |
| hsa-miR-411-3p | 9.89E-01 | 9.72E-01 | -0.035388 | -6.0734 | -0.03287 |
| hsa-miR-411-5p | 9.89E-01 | 9.72E-01 | -0.035388 | -6.0734 | -0.03287 |
| hsa-miR-412-3p | 9.89E-01 | 9.72E-01 | -0.035388 | -6.0734 | -0.03287 |
| hsa-miR-412-5p | 9.89E-01 | 9.72E-01 | -0.035388 | -6.0734 | -0.03287 |
| hsa-miR-422a | 9.89E-01 | 9.72E-01 | -0.035388 | -6.0734 | -0.03287 |
| hsa-miR-425-3p | 9.89E-01 | 9.72E-01 | -0.035388 | -6.0734 | -0.03287 |
| hsa-miR-4251 | 9.89E-01 | 9.72E-01 | -0.035388 | -6.0734 | -0.03287 |
| hsa-miR-4252 | 9.89E-01 | 9.72E-01 | -0.035388 | -6.0734 | -0.03287 |
| hsa-miR-4254 | 9.89E-01 | 9.72E-01 | -0.035388 | -6.0734 | -0.03287 |
| hsa-miR-4255 | 9.89E-01 | 9.72E-01 | -0.035388 | -6.0734 | -0.03287 |
| hsa-miR-4256 | 9.89E-01 | 9.72E-01 | -0.035388 | -6.0734 | -0.03287 |
| hsa-miR-4258 | 9.89E-01 | 9.72E-01 | -0.035388 | -6.0734 | -0.03287 |
| hsa-miR-4262 | 9.89E-01 | 9.72E-01 | -0.035388 | -6.0734 | -0.03287 |
| hsa-miR-4263 | 9.89E-01 | 9.72E-01 | -0.035388 | -6.0734 | -0.03287 |
| hsa-miR-4264 | 9.89E-01 | 9.72E-01 | -0.035388 | -6.0734 | -0.03287 |
| hsa-miR-4265 | 9.89E-01 | 9.72E-01 | -0.035388 | -6.0734 | -0.03287 |
| hsa-miR-4266 | 9.89E-01 | 9.72E-01 | -0.035388 | -6.0734 | -0.03287 |
| hsa-miR-4267 | 9.89E-01 | 9.72E-01 | -0.035388 | -6.0734 | -0.03287 |
| hsa-miR-4268 | 9.89E-01 | 9.72E-01 | -0.035388 | -6.0734 | -0.03287 |
| hsa-miR-4269 | 9.89E-01 | 9.72E-01 | -0.035388 | -6.0734 | -0.03287 |
| hsa-miR-4272 | 9.89E-01 | 9.72E-01 | -0.035388 | -6.0734 | -0.03287 |
| hsa-miR-4273 | 9.89E-01 | 9.72E-01 | -0.035388 | -6.0734 | -0.03287 |
| hsa-miR-4275 | 9.89E-01 | 9.72E-01 | -0.035388 | -6.0734 | -0.03287 |
| hsa-miR-4276 | 9.89E-01 | 9.72E-01 | -0.035388 | -6.0734 | -0.03287 |
| hsa-miR-4277 | 9.89E-01 | 9.72E-01 | -0.035388 | -6.0734 | -0.03287 |
| hsa-miR-4278 | 9.89E-01 | 9.72E-01 | -0.035388 | -6.0734 | -0.03287 |
| hsa-miR-4279 | 9.89E-01 | 9.72E-01 | -0.035388 | -6.0734 | -0.03287 |
| hsa-miR-4280 | 9.89E-01 | 9.72E-01 | -0.035388 | -6.0734 | -0.03287 |
| hsa-miR-4282 | 9.89E-01 | 9.72E-01 | -0.035388 | -6.0734 | -0.03287 |
| hsa-miR-4283 | 9.89E-01 | 9.72E-01 | -0.035388 | -6.0734 | -0.03287 |
| hsa-miR-4285 | 9.89E-01 | 9.72E-01 | -0.035388 | -6.0734 | -0.03287 |
| hsa-miR-4287 | 9.89E-01 | 9.72E-01 | -0.035388 | -6.0734 | -0.03287 |
| hsa-miR-4288 | 9.89E-01 | 9.72E-01 | -0.035388 | -6.0734 | -0.03287 |
| hsa-miR-4289 | 9.89E-01 | 9.72E-01 | -0.035388 | -6.0734 | -0.03287 |
| hsa-miR-4290 | 9.89E-01 | 9.72E-01 | -0.035388 | -6.0734 | -0.03287 |
| hsa-miR-4292 | 9.89E-01 | 9.72E-01 | -0.035388 | -6.0734 | -0.03287 |
| hsa-miR-4293 | 9.89E-01 | 9.72E-01 | -0.035388 | -6.0734 | -0.03287 |
| hsa-miR-4295 | 9.89E-01 | 9.72E-01 | -0.035388 | -6.0734 | -0.03287 |
| hsa-miR-4296 | 9.89E-01 | 9.72E-01 | -0.035388 | -6.0734 | -0.03287 |
| hsa-miR-4297 | 9.89E-01 | 9.72E-01 | -0.035388 | -6.0734 | -0.03287 |
| hsa-miR-4300 | 9.89E-01 | 9.72E-01 | -0.035388 | -6.0734 | -0.03287 |
| hsa-miR-4301 | 9.89E-01 | 9.72E-01 | -0.035388 | -6.0734 | -0.03287 |
| hsa-miR-4302 | 9.89E-01 | 9.72E-01 | -0.035388 | -6.0734 | -0.03287 |
| hsa-miR-4303 | 9.89E-01 | 9.72E-01 | -0.035388 | -6.0734 | -0.03287 |
| hsa-miR-4304 | 9.89E-01 | 9.72E-01 | -0.035388 | -6.0734 | -0.03287 |
| hsa-miR-4305 | 9.89E-01 | 9.72E-01 | -0.035388 | -6.0734 | -0.03287 |
| hsa-miR-4307 | 9.89E-01 | 9.72E-01 | -0.035388 | -6.0734 | -0.03287 |
| hsa-miR-4308 | 9.89E-01 | 9.72E-01 | -0.035388 | -6.0734 | -0.03287 |
| hsa-miR-4309 | 9.89E-01 | 9.72E-01 | -0.035388 | -6.0734 | -0.03287 |
| hsa-miR-431-5p | 9.89E-01 | 9.72E-01 | -0.035388 | -6.0734 | -0.03287 |
| hsa-miR-4310 | 9.89E-01 | 9.72E-01 | -0.035388 | -6.0734 | -0.03287 |
| hsa-miR-4311 | 9.89E-01 | 9.72E-01 | -0.035388 | -6.0734 | -0.03287 |
| hsa-miR-4312 | 9.89E-01 | 9.72E-01 | -0.035388 | -6.0734 | -0.03287 |
| hsa-miR-4315 | 9.89E-01 | 9.72E-01 | -0.035388 | -6.0734 | -0.03287 |
| hsa-miR-4316 | 9.89E-01 | 9.72E-01 | -0.035388 | -6.0734 | -0.03287 |
| hsa-miR-4318 | 9.89E-01 | 9.72E-01 | -0.035388 | -6.0734 | -0.03287 |
| hsa-miR-4319 | 9.89E-01 | 9.72E-01 | -0.035388 | -6.0734 | -0.03287 |
| hsa-miR-432-3p | 9.89E-01 | 9.72E-01 | -0.035388 | -6.0734 | -0.03287 |
| hsa-miR-4320 | 9.89E-01 | 9.72E-01 | -0.035388 | -6.0734 | -0.03287 |
| hsa-miR-4321 | 9.89E-01 | 9.72E-01 | -0.035388 | -6.0734 | -0.03287 |
| hsa-miR-4325 | 9.89E-01 | 9.72E-01 | -0.035388 | -6.0734 | -0.03287 |
| hsa-miR-4326 | 9.89E-01 | 9.72E-01 | -0.035388 | -6.0734 | -0.03287 |
| hsa-miR-4328 | 9.89E-01 | 9.72E-01 | -0.035388 | -6.0734 | -0.03287 |
| hsa-miR-4329 | 9.89E-01 | 9.72E-01 | -0.035388 | -6.0734 | -0.03287 |
| hsa-miR-433-3p | 9.89E-01 | 9.72E-01 | -0.035388 | -6.0734 | -0.03287 |
| hsa-miR-433-5p | 9.89E-01 | 9.72E-01 | -0.035388 | -6.0734 | -0.03287 |
| hsa-miR-4330 | 9.89E-01 | 9.72E-01 | -0.035388 | -6.0734 | -0.03287 |
| hsa-miR-4418 | 9.89E-01 | 9.72E-01 | -0.035388 | -6.0734 | -0.03287 |
| hsa-miR-4420 | 9.89E-01 | 9.72E-01 | -0.035388 | -6.0734 | -0.03287 |
| hsa-miR-4421 | 9.89E-01 | 9.72E-01 | -0.035388 | -6.0734 | -0.03287 |
| hsa-miR-4422 | 9.89E-01 | 9.72E-01 | -0.035388 | -6.0734 | -0.03287 |
| hsa-miR-4423-3p | 9.89E-01 | 9.72E-01 | -0.035388 | -6.0734 | -0.03287 |
| hsa-miR-4423-5p | 9.89E-01 | 9.72E-01 | -0.035388 | -6.0734 | -0.03287 |
| hsa-miR-4424 | 9.89E-01 | 9.72E-01 | -0.035388 | -6.0734 | -0.03287 |
| hsa-miR-4425 | 9.89E-01 | 9.72E-01 | -0.035388 | -6.0734 | -0.03287 |
| hsa-miR-4426 | 9.89E-01 | 9.72E-01 | -0.035388 | -6.0734 | -0.03287 |
| hsa-miR-4427 | 9.89E-01 | 9.72E-01 | -0.035388 | -6.0734 | -0.03287 |
| hsa-miR-4431 | 9.89E-01 | 9.72E-01 | -0.035388 | -6.0734 | -0.03287 |
| hsa-miR-4432 | 9.89E-01 | 9.72E-01 | -0.035388 | -6.0734 | -0.03287 |
| hsa-miR-4434 | 9.89E-01 | 9.72E-01 | -0.035388 | -6.0734 | -0.03287 |
| hsa-miR-4435 | 9.89E-01 | 9.72E-01 | -0.035388 | -6.0734 | -0.03287 |
| hsa-miR-4436a | 9.89E-01 | 9.72E-01 | -0.035388 | -6.0734 | -0.03287 |
| hsa-miR-4437 | 9.89E-01 | 9.72E-01 | -0.035388 | -6.0734 | -0.03287 |
| hsa-miR-4438 | 9.89E-01 | 9.72E-01 | -0.035388 | -6.0734 | -0.03287 |
| hsa-miR-4439 | 9.89E-01 | 9.72E-01 | -0.035388 | -6.0734 | -0.03287 |
| hsa-miR-4440 | 9.89E-01 | 9.72E-01 | -0.035388 | -6.0734 | -0.03287 |
| hsa-miR-4445-3p | 9.89E-01 | 9.72E-01 | -0.035388 | -6.0734 | -0.03287 |
| hsa-miR-4445-5p | 9.89E-01 | 9.72E-01 | -0.035388 | -6.0734 | -0.03287 |
| hsa-miR-4446-5p | 9.89E-01 | 9.72E-01 | -0.035388 | -6.0734 | -0.03287 |
| hsa-miR-4447 | 9.89E-01 | 9.72E-01 | -0.035388 | -6.0734 | -0.03287 |
| hsa-miR-4448 | 9.89E-01 | 9.72E-01 | -0.035388 | -6.0734 | -0.03287 |
| hsa-miR-4452 | 9.89E-01 | 9.72E-01 | -0.035388 | -6.0734 | -0.03287 |
| hsa-miR-4453 | 9.89E-01 | 9.72E-01 | -0.035388 | -6.0734 | -0.03287 |
| hsa-miR-4456 | 9.89E-01 | 9.72E-01 | -0.035388 | -6.0734 | -0.03287 |
| hsa-miR-4457 | 9.89E-01 | 9.72E-01 | -0.035388 | -6.0734 | -0.03287 |
| hsa-miR-4458 | 9.89E-01 | 9.72E-01 | -0.035388 | -6.0734 | -0.03287 |
| hsa-miR-4460 | 9.89E-01 | 9.72E-01 | -0.035388 | -6.0734 | -0.03287 |
| hsa-miR-4461 | 9.89E-01 | 9.72E-01 | -0.035388 | -6.0734 | -0.03287 |
| hsa-miR-4464 | 9.89E-01 | 9.72E-01 | -0.035388 | -6.0734 | -0.03287 |
| hsa-miR-4467 | 9.89E-01 | 9.72E-01 | -0.035388 | -6.0734 | -0.03287 |
| hsa-miR-4469 | 9.89E-01 | 9.72E-01 | -0.035388 | -6.0734 | -0.03287 |
| hsa-miR-4471 | 9.89E-01 | 9.72E-01 | -0.035388 | -6.0734 | -0.03287 |
| hsa-miR-4473 | 9.89E-01 | 9.72E-01 | -0.035388 | -6.0734 | -0.03287 |
| hsa-miR-4474-3p | 9.89E-01 | 9.72E-01 | -0.035388 | -6.0734 | -0.03287 |
| hsa-miR-4474-5p | 9.89E-01 | 9.72E-01 | -0.035388 | -6.0734 | -0.03287 |
| hsa-miR-4475 | 9.89E-01 | 9.72E-01 | -0.035388 | -6.0734 | -0.03287 |
| hsa-miR-4477a | 9.89E-01 | 9.72E-01 | -0.035388 | -6.0734 | -0.03287 |
| hsa-miR-4477b | 9.89E-01 | 9.72E-01 | -0.035388 | -6.0734 | -0.03287 |
| hsa-miR-4479 | 9.89E-01 | 9.72E-01 | -0.035388 | -6.0734 | -0.03287 |
| hsa-miR-448 | 9.89E-01 | 9.72E-01 | -0.035388 | -6.0734 | -0.03287 |
| hsa-miR-4480 | 9.89E-01 | 9.72E-01 | -0.035388 | -6.0734 | -0.03287 |
| hsa-miR-4482-5p | 9.89E-01 | 9.72E-01 | -0.035388 | -6.0734 | -0.03287 |
| hsa-miR-4483 | 9.89E-01 | 9.72E-01 | -0.035388 | -6.0734 | -0.03287 |
| hsa-miR-4489 | 9.89E-01 | 9.72E-01 | -0.035388 | -6.0734 | -0.03287 |
| hsa-miR-4490 | 9.89E-01 | 9.72E-01 | -0.035388 | -6.0734 | -0.03287 |
| hsa-miR-4491 | 9.89E-01 | 9.72E-01 | -0.035388 | -6.0734 | -0.03287 |
| hsa-miR-4492 | 9.89E-01 | 9.72E-01 | -0.035388 | -6.0734 | -0.03287 |
| hsa-miR-4493 | 9.89E-01 | 9.72E-01 | -0.035388 | -6.0734 | -0.03287 |
| hsa-miR-4495 | 9.89E-01 | 9.72E-01 | -0.035388 | -6.0734 | -0.03287 |
| hsa-miR-4498 | 9.89E-01 | 9.72E-01 | -0.035388 | -6.0734 | -0.03287 |
| hsa-miR-449a | 9.89E-01 | 9.72E-01 | -0.035388 | -6.0734 | -0.03287 |
| hsa-miR-449b-3p | 9.89E-01 | 9.72E-01 | -0.035388 | -6.0734 | -0.03287 |
| hsa-miR-449b-5p | 9.89E-01 | 9.72E-01 | -0.035388 | -6.0734 | -0.03287 |
| hsa-miR-449c-3p | 9.89E-01 | 9.72E-01 | -0.035388 | -6.0734 | -0.03287 |
| hsa-miR-449c-5p | 9.89E-01 | 9.72E-01 | -0.035388 | -6.0734 | -0.03287 |
| hsa-miR-4500 | 9.89E-01 | 9.72E-01 | -0.035388 | -6.0734 | -0.03287 |
| hsa-miR-4501 | 9.89E-01 | 9.72E-01 | -0.035388 | -6.0734 | -0.03287 |
| hsa-miR-4502 | 9.89E-01 | 9.72E-01 | -0.035388 | -6.0734 | -0.03287 |
| hsa-miR-4503 | 9.89E-01 | 9.72E-01 | -0.035388 | -6.0734 | -0.03287 |
| hsa-miR-4504 | 9.89E-01 | 9.72E-01 | -0.035388 | -6.0734 | -0.03287 |
| hsa-miR-4508 | 9.89E-01 | 9.72E-01 | -0.035388 | -6.0734 | -0.03287 |
| hsa-miR-4509 | 9.89E-01 | 9.72E-01 | -0.035388 | -6.0734 | -0.03287 |
| hsa-miR-450a-1-3p | 9.89E-01 | 9.72E-01 | -0.035388 | -6.0734 | -0.03287 |
| hsa-miR-450a-2-3p | 9.89E-01 | 9.72E-01 | -0.035388 | -6.0734 | -0.03287 |
| hsa-miR-450b-3p | 9.89E-01 | 9.72E-01 | -0.035388 | -6.0734 | -0.03287 |
| hsa-miR-450b-5p | 9.89E-01 | 9.72E-01 | -0.035388 | -6.0734 | -0.03287 |
| hsa-miR-4510 | 9.89E-01 | 9.72E-01 | -0.035388 | -6.0734 | -0.03287 |
| hsa-miR-4511 | 9.89E-01 | 9.72E-01 | -0.035388 | -6.0734 | -0.03287 |
| hsa-miR-4512 | 9.89E-01 | 9.72E-01 | -0.035388 | -6.0734 | -0.03287 |
| hsa-miR-4517 | 9.89E-01 | 9.72E-01 | -0.035388 | -6.0734 | -0.03287 |
| hsa-miR-4518 | 9.89E-01 | 9.72E-01 | -0.035388 | -6.0734 | -0.03287 |
| hsa-miR-4519 | 9.89E-01 | 9.72E-01 | -0.035388 | -6.0734 | -0.03287 |
| hsa-miR-451b | 9.89E-01 | 9.72E-01 | -0.035388 | -6.0734 | -0.03287 |
| hsa-miR-452-3p | 9.89E-01 | 9.72E-01 | -0.035388 | -6.0734 | -0.03287 |
| hsa-miR-4520-2-3p | 9.89E-01 | 9.72E-01 | -0.035388 | -6.0734 | -0.03287 |
| hsa-miR-4520-3p | 9.89E-01 | 9.72E-01 | -0.035388 | -6.0734 | -0.03287 |
| hsa-miR-4520-5p | 9.89E-01 | 9.72E-01 | -0.035388 | -6.0734 | -0.03287 |
| hsa-miR-4521 | 9.89E-01 | 9.72E-01 | -0.035388 | -6.0734 | -0.03287 |
| hsa-miR-4523 | 9.89E-01 | 9.72E-01 | -0.035388 | -6.0734 | -0.03287 |
| hsa-miR-4524a-3p | 9.89E-01 | 9.72E-01 | -0.035388 | -6.0734 | -0.03287 |
| hsa-miR-4524a-5p | 9.89E-01 | 9.72E-01 | -0.035388 | -6.0734 | -0.03287 |
| hsa-miR-4524b-3p | 9.89E-01 | 9.72E-01 | -0.035388 | -6.0734 | -0.03287 |
| hsa-miR-4524b-5p | 9.89E-01 | 9.72E-01 | -0.035388 | -6.0734 | -0.03287 |
| hsa-miR-4525 | 9.89E-01 | 9.72E-01 | -0.035388 | -6.0734 | -0.03287 |
| hsa-miR-4526 | 9.89E-01 | 9.72E-01 | -0.035388 | -6.0734 | -0.03287 |
| hsa-miR-4527 | 9.89E-01 | 9.72E-01 | -0.035388 | -6.0734 | -0.03287 |
| hsa-miR-4528 | 9.89E-01 | 9.72E-01 | -0.035388 | -6.0734 | -0.03287 |
| hsa-miR-4529-3p | 9.89E-01 | 9.72E-01 | -0.035388 | -6.0734 | -0.03287 |
| hsa-miR-4529-5p | 9.89E-01 | 9.72E-01 | -0.035388 | -6.0734 | -0.03287 |
| hsa-miR-4531 | 9.89E-01 | 9.72E-01 | -0.035388 | -6.0734 | -0.03287 |
| hsa-miR-4533 | 9.89E-01 | 9.72E-01 | -0.035388 | -6.0734 | -0.03287 |
| hsa-miR-4536-3p | 9.89E-01 | 9.72E-01 | -0.035388 | -6.0734 | -0.03287 |
| hsa-miR-4536-5p | 9.89E-01 | 9.72E-01 | -0.035388 | -6.0734 | -0.03287 |
| hsa-miR-4537 | 9.89E-01 | 9.72E-01 | -0.035388 | -6.0734 | -0.03287 |
| hsa-miR-454-5p | 9.89E-01 | 9.72E-01 | -0.035388 | -6.0734 | -0.03287 |
| hsa-miR-4540 | 9.89E-01 | 9.72E-01 | -0.035388 | -6.0734 | -0.03287 |
| hsa-miR-4632-3p | 9.89E-01 | 9.72E-01 | -0.035388 | -6.0734 | -0.03287 |
| hsa-miR-4633-3p | 9.89E-01 | 9.72E-01 | -0.035388 | -6.0734 | -0.03287 |
| hsa-miR-4635 | 9.89E-01 | 9.72E-01 | -0.035388 | -6.0734 | -0.03287 |
| hsa-miR-4636 | 9.89E-01 | 9.72E-01 | -0.035388 | -6.0734 | -0.03287 |
| hsa-miR-4637 | 9.89E-01 | 9.72E-01 | -0.035388 | -6.0734 | -0.03287 |
| hsa-miR-4638-3p | 9.89E-01 | 9.72E-01 | -0.035388 | -6.0734 | -0.03287 |
| hsa-miR-4638-5p | 9.89E-01 | 9.72E-01 | -0.035388 | -6.0734 | -0.03287 |
| hsa-miR-4639-3p | 9.89E-01 | 9.72E-01 | -0.035388 | -6.0734 | -0.03287 |
| hsa-miR-4639-5p | 9.89E-01 | 9.72E-01 | -0.035388 | -6.0734 | -0.03287 |
| hsa-miR-4640-3p | 9.89E-01 | 9.72E-01 | -0.035388 | -6.0734 | -0.03287 |
| hsa-miR-4641 | 9.89E-01 | 9.72E-01 | -0.035388 | -6.0734 | -0.03287 |
| hsa-miR-4642 | 9.89E-01 | 9.72E-01 | -0.035388 | -6.0734 | -0.03287 |
| hsa-miR-4643 | 9.89E-01 | 9.72E-01 | -0.035388 | -6.0734 | -0.03287 |
| hsa-miR-4645-3p | 9.89E-01 | 9.72E-01 | -0.035388 | -6.0734 | -0.03287 |
| hsa-miR-4645-5p | 9.89E-01 | 9.72E-01 | -0.035388 | -6.0734 | -0.03287 |
| hsa-miR-4646-3p | 9.89E-01 | 9.72E-01 | -0.035388 | -6.0734 | -0.03287 |
| hsa-miR-4648 | 9.89E-01 | 9.72E-01 | -0.035388 | -6.0734 | -0.03287 |
| hsa-miR-4649-5p | 9.89E-01 | 9.72E-01 | -0.035388 | -6.0734 | -0.03287 |
| hsa-miR-4650-3p | 9.89E-01 | 9.72E-01 | -0.035388 | -6.0734 | -0.03287 |
| hsa-miR-4650-5p | 9.89E-01 | 9.72E-01 | -0.035388 | -6.0734 | -0.03287 |
| hsa-miR-4652-3p | 9.89E-01 | 9.72E-01 | -0.035388 | -6.0734 | -0.03287 |
| hsa-miR-4652-5p | 9.89E-01 | 9.72E-01 | -0.035388 | -6.0734 | -0.03287 |
| hsa-miR-4653-5p | 9.89E-01 | 9.72E-01 | -0.035388 | -6.0734 | -0.03287 |
| hsa-miR-4654 | 9.89E-01 | 9.72E-01 | -0.035388 | -6.0734 | -0.03287 |
| hsa-miR-4658 | 9.89E-01 | 9.72E-01 | -0.035388 | -6.0734 | -0.03287 |
| hsa-miR-4659a-3p | 9.89E-01 | 9.72E-01 | -0.035388 | -6.0734 | -0.03287 |
| hsa-miR-4659a-5p | 9.89E-01 | 9.72E-01 | -0.035388 | -6.0734 | -0.03287 |
| hsa-miR-4659b-3p | 9.89E-01 | 9.72E-01 | -0.035388 | -6.0734 | -0.03287 |
| hsa-miR-4659b-5p | 9.89E-01 | 9.72E-01 | -0.035388 | -6.0734 | -0.03287 |
| hsa-miR-466 | 9.89E-01 | 9.72E-01 | -0.035388 | -6.0734 | -0.03287 |
| hsa-miR-4661-3p | 9.89E-01 | 9.72E-01 | -0.035388 | -6.0734 | -0.03287 |
| hsa-miR-4661-5p | 9.89E-01 | 9.72E-01 | -0.035388 | -6.0734 | -0.03287 |
| hsa-miR-4662a-3p | 9.89E-01 | 9.72E-01 | -0.035388 | -6.0734 | -0.03287 |
| hsa-miR-4662a-5p | 9.89E-01 | 9.72E-01 | -0.035388 | -6.0734 | -0.03287 |
| hsa-miR-4662b | 9.89E-01 | 9.72E-01 | -0.035388 | -6.0734 | -0.03287 |
| hsa-miR-4663 | 9.89E-01 | 9.72E-01 | -0.035388 | -6.0734 | -0.03287 |
| hsa-miR-4664-3p | 9.89E-01 | 9.72E-01 | -0.035388 | -6.0734 | -0.03287 |
| hsa-miR-4664-5p | 9.89E-01 | 9.72E-01 | -0.035388 | -6.0734 | -0.03287 |
| hsa-miR-4666a-3p | 9.89E-01 | 9.72E-01 | -0.035388 | -6.0734 | -0.03287 |
| hsa-miR-4666a-5p | 9.89E-01 | 9.72E-01 | -0.035388 | -6.0734 | -0.03287 |
| hsa-miR-4666b | 9.89E-01 | 9.72E-01 | -0.035388 | -6.0734 | -0.03287 |
| hsa-miR-4667-3p | 9.89E-01 | 9.72E-01 | -0.035388 | -6.0734 | -0.03287 |
| hsa-miR-4668-3p | 9.89E-01 | 9.72E-01 | -0.035388 | -6.0734 | -0.03287 |
| hsa-miR-4668-5p | 9.89E-01 | 9.72E-01 | -0.035388 | -6.0734 | -0.03287 |
| hsa-miR-4670-3p | 9.89E-01 | 9.72E-01 | -0.035388 | -6.0734 | -0.03287 |
| hsa-miR-4670-5p | 9.89E-01 | 9.72E-01 | -0.035388 | -6.0734 | -0.03287 |
| hsa-miR-4671-3p | 9.89E-01 | 9.72E-01 | -0.035388 | -6.0734 | -0.03287 |
| hsa-miR-4671-5p | 9.89E-01 | 9.72E-01 | -0.035388 | -6.0734 | -0.03287 |
| hsa-miR-4674 | 9.89E-01 | 9.72E-01 | -0.035388 | -6.0734 | -0.03287 |
| hsa-miR-4675 | 9.89E-01 | 9.72E-01 | -0.035388 | -6.0734 | -0.03287 |
| hsa-miR-4676-3p | 9.89E-01 | 9.72E-01 | -0.035388 | -6.0734 | -0.03287 |
| hsa-miR-4676-5p | 9.89E-01 | 9.72E-01 | -0.035388 | -6.0734 | -0.03287 |
| hsa-miR-4677-3p | 9.89E-01 | 9.72E-01 | -0.035388 | -6.0734 | -0.03287 |
| hsa-miR-4677-5p | 9.89E-01 | 9.72E-01 | -0.035388 | -6.0734 | -0.03287 |
| hsa-miR-4678 | 9.89E-01 | 9.72E-01 | -0.035388 | -6.0734 | -0.03287 |
| hsa-miR-4679 | 9.89E-01 | 9.72E-01 | -0.035388 | -6.0734 | -0.03287 |
| hsa-miR-4680-3p | 9.89E-01 | 9.72E-01 | -0.035388 | -6.0734 | -0.03287 |
| hsa-miR-4680-5p | 9.89E-01 | 9.72E-01 | -0.035388 | -6.0734 | -0.03287 |
| hsa-miR-4681 | 9.89E-01 | 9.72E-01 | -0.035388 | -6.0734 | -0.03287 |
| hsa-miR-4682 | 9.89E-01 | 9.72E-01 | -0.035388 | -6.0734 | -0.03287 |
| hsa-miR-4683 | 9.89E-01 | 9.72E-01 | -0.035388 | -6.0734 | -0.03287 |
| hsa-miR-4684-3p | 9.89E-01 | 9.72E-01 | -0.035388 | -6.0734 | -0.03287 |
| hsa-miR-4684-5p | 9.89E-01 | 9.72E-01 | -0.035388 | -6.0734 | -0.03287 |
| hsa-miR-4685-3p | 9.89E-01 | 9.72E-01 | -0.035388 | -6.0734 | -0.03287 |
| hsa-miR-4686 | 9.89E-01 | 9.72E-01 | -0.035388 | -6.0734 | -0.03287 |
| hsa-miR-4687-5p | 9.89E-01 | 9.72E-01 | -0.035388 | -6.0734 | -0.03287 |
| hsa-miR-4690-3p | 9.89E-01 | 9.72E-01 | -0.035388 | -6.0734 | -0.03287 |
| hsa-miR-4691-3p | 9.89E-01 | 9.72E-01 | -0.035388 | -6.0734 | -0.03287 |
| hsa-miR-4691-5p | 9.89E-01 | 9.72E-01 | -0.035388 | -6.0734 | -0.03287 |
| hsa-miR-4692 | 9.89E-01 | 9.72E-01 | -0.035388 | -6.0734 | -0.03287 |
| hsa-miR-4693-3p | 9.89E-01 | 9.72E-01 | -0.035388 | -6.0734 | -0.03287 |
| hsa-miR-4693-5p | 9.89E-01 | 9.72E-01 | -0.035388 | -6.0734 | -0.03287 |
| hsa-miR-4694-3p | 9.89E-01 | 9.72E-01 | -0.035388 | -6.0734 | -0.03287 |
| hsa-miR-4694-5p | 9.89E-01 | 9.72E-01 | -0.035388 | -6.0734 | -0.03287 |
| hsa-miR-4695-3p | 9.89E-01 | 9.72E-01 | -0.035388 | -6.0734 | -0.03287 |
| hsa-miR-4696 | 9.89E-01 | 9.72E-01 | -0.035388 | -6.0734 | -0.03287 |
| hsa-miR-4697-3p | 9.89E-01 | 9.72E-01 | -0.035388 | -6.0734 | -0.03287 |
| hsa-miR-4699-3p | 9.89E-01 | 9.72E-01 | -0.035388 | -6.0734 | -0.03287 |
| hsa-miR-4699-5p | 9.89E-01 | 9.72E-01 | -0.035388 | -6.0734 | -0.03287 |
| hsa-miR-4700-3p | 9.89E-01 | 9.72E-01 | -0.035388 | -6.0734 | -0.03287 |
| hsa-miR-4700-5p | 9.89E-01 | 9.72E-01 | -0.035388 | -6.0734 | -0.03287 |
| hsa-miR-4701-5p | 9.89E-01 | 9.72E-01 | -0.035388 | -6.0734 | -0.03287 |
| hsa-miR-4703-3p | 9.89E-01 | 9.72E-01 | -0.035388 | -6.0734 | -0.03287 |
| hsa-miR-4703-5p | 9.89E-01 | 9.72E-01 | -0.035388 | -6.0734 | -0.03287 |
| hsa-miR-4704-3p | 9.89E-01 | 9.72E-01 | -0.035388 | -6.0734 | -0.03287 |
| hsa-miR-4704-5p | 9.89E-01 | 9.72E-01 | -0.035388 | -6.0734 | -0.03287 |
| hsa-miR-4705 | 9.89E-01 | 9.72E-01 | -0.035388 | -6.0734 | -0.03287 |
| hsa-miR-4706 | 9.89E-01 | 9.72E-01 | -0.035388 | -6.0734 | -0.03287 |
| hsa-miR-4707-3p | 9.89E-01 | 9.72E-01 | -0.035388 | -6.0734 | -0.03287 |
| hsa-miR-4708-3p | 9.89E-01 | 9.72E-01 | -0.035388 | -6.0734 | -0.03287 |
| hsa-miR-4708-5p | 9.89E-01 | 9.72E-01 | -0.035388 | -6.0734 | -0.03287 |
| hsa-miR-4709-5p | 9.89E-01 | 9.72E-01 | -0.035388 | -6.0734 | -0.03287 |
| hsa-miR-4711-3p | 9.89E-01 | 9.72E-01 | -0.035388 | -6.0734 | -0.03287 |
| hsa-miR-4711-5p | 9.89E-01 | 9.72E-01 | -0.035388 | -6.0734 | -0.03287 |
| hsa-miR-4712-3p | 9.89E-01 | 9.72E-01 | -0.035388 | -6.0734 | -0.03287 |
| hsa-miR-4712-5p | 9.89E-01 | 9.72E-01 | -0.035388 | -6.0734 | -0.03287 |
| hsa-miR-4713-5p | 9.89E-01 | 9.72E-01 | -0.035388 | -6.0734 | -0.03287 |
| hsa-miR-4714-3p | 9.89E-01 | 9.72E-01 | -0.035388 | -6.0734 | -0.03287 |
| hsa-miR-4714-5p | 9.89E-01 | 9.72E-01 | -0.035388 | -6.0734 | -0.03287 |
| hsa-miR-4715-3p | 9.89E-01 | 9.72E-01 | -0.035388 | -6.0734 | -0.03287 |
| hsa-miR-4715-5p | 9.89E-01 | 9.72E-01 | -0.035388 | -6.0734 | -0.03287 |
| hsa-miR-4716-5p | 9.89E-01 | 9.72E-01 | -0.035388 | -6.0734 | -0.03287 |
| hsa-miR-4717-3p | 9.89E-01 | 9.72E-01 | -0.035388 | -6.0734 | -0.03287 |
| hsa-miR-4717-5p | 9.89E-01 | 9.72E-01 | -0.035388 | -6.0734 | -0.03287 |
| hsa-miR-4718 | 9.89E-01 | 9.72E-01 | -0.035388 | -6.0734 | -0.03287 |
| hsa-miR-4719 | 9.89E-01 | 9.72E-01 | -0.035388 | -6.0734 | -0.03287 |
| hsa-miR-4720-3p | 9.89E-01 | 9.72E-01 | -0.035388 | -6.0734 | -0.03287 |
| hsa-miR-4720-5p | 9.89E-01 | 9.72E-01 | -0.035388 | -6.0734 | -0.03287 |
| hsa-miR-4722-3p | 9.89E-01 | 9.72E-01 | -0.035388 | -6.0734 | -0.03287 |
| hsa-miR-4722-5p | 9.89E-01 | 9.72E-01 | -0.035388 | -6.0734 | -0.03287 |
| hsa-miR-4723-3p | 9.89E-01 | 9.72E-01 | -0.035388 | -6.0734 | -0.03287 |
| hsa-miR-4723-5p | 9.89E-01 | 9.72E-01 | -0.035388 | -6.0734 | -0.03287 |
| hsa-miR-4724-3p | 9.89E-01 | 9.72E-01 | -0.035388 | -6.0734 | -0.03287 |
| hsa-miR-4724-5p | 9.89E-01 | 9.72E-01 | -0.035388 | -6.0734 | -0.03287 |
| hsa-miR-4725-3p | 9.89E-01 | 9.72E-01 | -0.035388 | -6.0734 | -0.03287 |
| hsa-miR-4726-3p | 9.89E-01 | 9.72E-01 | -0.035388 | -6.0734 | -0.03287 |
| hsa-miR-4727-3p | 9.89E-01 | 9.72E-01 | -0.035388 | -6.0734 | -0.03287 |
| hsa-miR-4727-5p | 9.89E-01 | 9.72E-01 | -0.035388 | -6.0734 | -0.03287 |
| hsa-miR-4728-3p | 9.89E-01 | 9.72E-01 | -0.035388 | -6.0734 | -0.03287 |
| hsa-miR-4729 | 9.89E-01 | 9.72E-01 | -0.035388 | -6.0734 | -0.03287 |
| hsa-miR-4730 | 9.89E-01 | 9.72E-01 | -0.035388 | -6.0734 | -0.03287 |
| hsa-miR-4731-5p | 9.89E-01 | 9.72E-01 | -0.035388 | -6.0734 | -0.03287 |
| hsa-miR-4732-3p | 9.89E-01 | 9.72E-01 | -0.035388 | -6.0734 | -0.03287 |
| hsa-miR-4733-3p | 9.89E-01 | 9.72E-01 | -0.035388 | -6.0734 | -0.03287 |
| hsa-miR-4733-5p | 9.89E-01 | 9.72E-01 | -0.035388 | -6.0734 | -0.03287 |
| hsa-miR-4735-3p | 9.89E-01 | 9.72E-01 | -0.035388 | -6.0734 | -0.03287 |
| hsa-miR-4735-5p | 9.89E-01 | 9.72E-01 | -0.035388 | -6.0734 | -0.03287 |
| hsa-miR-4736 | 9.89E-01 | 9.72E-01 | -0.035388 | -6.0734 | -0.03287 |
| hsa-miR-4737 | 9.89E-01 | 9.72E-01 | -0.035388 | -6.0734 | -0.03287 |
| hsa-miR-4738-5p | 9.89E-01 | 9.72E-01 | -0.035388 | -6.0734 | -0.03287 |
| hsa-miR-4740-3p | 9.89E-01 | 9.72E-01 | -0.035388 | -6.0734 | -0.03287 |
| hsa-miR-4742-3p | 9.89E-01 | 9.72E-01 | -0.035388 | -6.0734 | -0.03287 |
| hsa-miR-4742-5p | 9.89E-01 | 9.72E-01 | -0.035388 | -6.0734 | -0.03287 |
| hsa-miR-4743-3p | 9.89E-01 | 9.72E-01 | -0.035388 | -6.0734 | -0.03287 |
| hsa-miR-4744 | 9.89E-01 | 9.72E-01 | -0.035388 | -6.0734 | -0.03287 |
| hsa-miR-4745-3p | 9.89E-01 | 9.72E-01 | -0.035388 | -6.0734 | -0.03287 |
| hsa-miR-4747-3p | 9.89E-01 | 9.72E-01 | -0.035388 | -6.0734 | -0.03287 |
| hsa-miR-4747-5p | 9.89E-01 | 9.72E-01 | -0.035388 | -6.0734 | -0.03287 |
| hsa-miR-4748 | 9.89E-01 | 9.72E-01 | -0.035388 | -6.0734 | -0.03287 |
| hsa-miR-4750-3p | 9.89E-01 | 9.72E-01 | -0.035388 | -6.0734 | -0.03287 |
| hsa-miR-4751 | 9.89E-01 | 9.72E-01 | -0.035388 | -6.0734 | -0.03287 |
| hsa-miR-4752 | 9.89E-01 | 9.72E-01 | -0.035388 | -6.0734 | -0.03287 |
| hsa-miR-4753-3p | 9.89E-01 | 9.72E-01 | -0.035388 | -6.0734 | -0.03287 |
| hsa-miR-4755-5p | 9.89E-01 | 9.72E-01 | -0.035388 | -6.0734 | -0.03287 |
| hsa-miR-4756-3p | 9.89E-01 | 9.72E-01 | -0.035388 | -6.0734 | -0.03287 |
| hsa-miR-4756-5p | 9.89E-01 | 9.72E-01 | -0.035388 | -6.0734 | -0.03287 |
| hsa-miR-4757-3p | 9.89E-01 | 9.72E-01 | -0.035388 | -6.0734 | -0.03287 |
| hsa-miR-4757-5p | 9.89E-01 | 9.72E-01 | -0.035388 | -6.0734 | -0.03287 |
| hsa-miR-4759 | 9.89E-01 | 9.72E-01 | -0.035388 | -6.0734 | -0.03287 |
| hsa-miR-4760-3p | 9.89E-01 | 9.72E-01 | -0.035388 | -6.0734 | -0.03287 |
| hsa-miR-4760-5p | 9.89E-01 | 9.72E-01 | -0.035388 | -6.0734 | -0.03287 |
| hsa-miR-4761-3p | 9.89E-01 | 9.72E-01 | -0.035388 | -6.0734 | -0.03287 |
| hsa-miR-4761-5p | 9.89E-01 | 9.72E-01 | -0.035388 | -6.0734 | -0.03287 |
| hsa-miR-4762-3p | 9.89E-01 | 9.72E-01 | -0.035388 | -6.0734 | -0.03287 |
| hsa-miR-4762-5p | 9.89E-01 | 9.72E-01 | -0.035388 | -6.0734 | -0.03287 |
| hsa-miR-4763-5p | 9.89E-01 | 9.72E-01 | -0.035388 | -6.0734 | -0.03287 |
| hsa-miR-4764-3p | 9.89E-01 | 9.72E-01 | -0.035388 | -6.0734 | -0.03287 |
| hsa-miR-4764-5p | 9.89E-01 | 9.72E-01 | -0.035388 | -6.0734 | -0.03287 |
| hsa-miR-4765 | 9.89E-01 | 9.72E-01 | -0.035388 | -6.0734 | -0.03287 |
| hsa-miR-4766-3p | 9.89E-01 | 9.72E-01 | -0.035388 | -6.0734 | -0.03287 |
| hsa-miR-4766-5p | 9.89E-01 | 9.72E-01 | -0.035388 | -6.0734 | -0.03287 |
| hsa-miR-4768-5p | 9.89E-01 | 9.72E-01 | -0.035388 | -6.0734 | -0.03287 |
| hsa-miR-4770 | 9.89E-01 | 9.72E-01 | -0.035388 | -6.0734 | -0.03287 |
| hsa-miR-4771 | 9.89E-01 | 9.72E-01 | -0.035388 | -6.0734 | -0.03287 |
| hsa-miR-4772-3p | 9.89E-01 | 9.72E-01 | -0.035388 | -6.0734 | -0.03287 |
| hsa-miR-4772-5p | 9.89E-01 | 9.72E-01 | -0.035388 | -6.0734 | -0.03287 |
| hsa-miR-4773 | 9.89E-01 | 9.72E-01 | -0.035388 | -6.0734 | -0.03287 |
| hsa-miR-4774-3p | 9.89E-01 | 9.72E-01 | -0.035388 | -6.0734 | -0.03287 |
| hsa-miR-4774-5p | 9.89E-01 | 9.72E-01 | -0.035388 | -6.0734 | -0.03287 |
| hsa-miR-4775 | 9.89E-01 | 9.72E-01 | -0.035388 | -6.0734 | -0.03287 |
| hsa-miR-4776-3p | 9.89E-01 | 9.72E-01 | -0.035388 | -6.0734 | -0.03287 |
| hsa-miR-4777-3p | 9.89E-01 | 9.72E-01 | -0.035388 | -6.0734 | -0.03287 |
| hsa-miR-4777-5p | 9.89E-01 | 9.72E-01 | -0.035388 | -6.0734 | -0.03287 |
| hsa-miR-4778-3p | 9.89E-01 | 9.72E-01 | -0.035388 | -6.0734 | -0.03287 |
| hsa-miR-4779 | 9.89E-01 | 9.72E-01 | -0.035388 | -6.0734 | -0.03287 |
| hsa-miR-4780 | 9.89E-01 | 9.72E-01 | -0.035388 | -6.0734 | -0.03287 |
| hsa-miR-4781-3p | 9.89E-01 | 9.72E-01 | -0.035388 | -6.0734 | -0.03287 |
| hsa-miR-4781-5p | 9.89E-01 | 9.72E-01 | -0.035388 | -6.0734 | -0.03287 |
| hsa-miR-4782-3p | 9.89E-01 | 9.72E-01 | -0.035388 | -6.0734 | -0.03287 |
| hsa-miR-4782-5p | 9.89E-01 | 9.72E-01 | -0.035388 | -6.0734 | -0.03287 |
| hsa-miR-4783-5p | 9.89E-01 | 9.72E-01 | -0.035388 | -6.0734 | -0.03287 |
| hsa-miR-4785 | 9.89E-01 | 9.72E-01 | -0.035388 | -6.0734 | -0.03287 |
| hsa-miR-4786-3p | 9.89E-01 | 9.72E-01 | -0.035388 | -6.0734 | -0.03287 |
| hsa-miR-4786-5p | 9.89E-01 | 9.72E-01 | -0.035388 | -6.0734 | -0.03287 |
| hsa-miR-4789-3p | 9.89E-01 | 9.72E-01 | -0.035388 | -6.0734 | -0.03287 |
| hsa-miR-4789-5p | 9.89E-01 | 9.72E-01 | -0.035388 | -6.0734 | -0.03287 |
| hsa-miR-4790-3p | 9.89E-01 | 9.72E-01 | -0.035388 | -6.0734 | -0.03287 |
| hsa-miR-4790-5p | 9.89E-01 | 9.72E-01 | -0.035388 | -6.0734 | -0.03287 |
| hsa-miR-4791 | 9.89E-01 | 9.72E-01 | -0.035388 | -6.0734 | -0.03287 |
| hsa-miR-4792 | 9.89E-01 | 9.72E-01 | -0.035388 | -6.0734 | -0.03287 |
| hsa-miR-4793-3p | 9.89E-01 | 9.72E-01 | -0.035388 | -6.0734 | -0.03287 |
| hsa-miR-4794 | 9.89E-01 | 9.72E-01 | -0.035388 | -6.0734 | -0.03287 |
| hsa-miR-4795-3p | 9.89E-01 | 9.72E-01 | -0.035388 | -6.0734 | -0.03287 |
| hsa-miR-4795-5p | 9.89E-01 | 9.72E-01 | -0.035388 | -6.0734 | -0.03287 |
| hsa-miR-4796-3p | 9.89E-01 | 9.72E-01 | -0.035388 | -6.0734 | -0.03287 |
| hsa-miR-4796-5p | 9.89E-01 | 9.72E-01 | -0.035388 | -6.0734 | -0.03287 |
| hsa-miR-4797-3p | 9.89E-01 | 9.72E-01 | -0.035388 | -6.0734 | -0.03287 |
| hsa-miR-4797-5p | 9.89E-01 | 9.72E-01 | -0.035388 | -6.0734 | -0.03287 |
| hsa-miR-4798-3p | 9.89E-01 | 9.72E-01 | -0.035388 | -6.0734 | -0.03287 |
| hsa-miR-4798-5p | 9.89E-01 | 9.72E-01 | -0.035388 | -6.0734 | -0.03287 |
| hsa-miR-4799-3p | 9.89E-01 | 9.72E-01 | -0.035388 | -6.0734 | -0.03287 |
| hsa-miR-4799-5p | 9.89E-01 | 9.72E-01 | -0.035388 | -6.0734 | -0.03287 |
| hsa-miR-4800-3p | 9.89E-01 | 9.72E-01 | -0.035388 | -6.0734 | -0.03287 |
| hsa-miR-4801 | 9.89E-01 | 9.72E-01 | -0.035388 | -6.0734 | -0.03287 |
| hsa-miR-4802-3p | 9.89E-01 | 9.72E-01 | -0.035388 | -6.0734 | -0.03287 |
| hsa-miR-4802-5p | 9.89E-01 | 9.72E-01 | -0.035388 | -6.0734 | -0.03287 |
| hsa-miR-4803 | 9.89E-01 | 9.72E-01 | -0.035388 | -6.0734 | -0.03287 |
| hsa-miR-4804-3p | 9.89E-01 | 9.72E-01 | -0.035388 | -6.0734 | -0.03287 |
| hsa-miR-4804-5p | 9.89E-01 | 9.72E-01 | -0.035388 | -6.0734 | -0.03287 |
| hsa-miR-483-3p | 9.89E-01 | 9.72E-01 | -0.035388 | -6.0734 | -0.03287 |
| hsa-miR-485-3p | 9.89E-01 | 9.72E-01 | -0.035388 | -6.0734 | -0.03287 |
| hsa-miR-485-5p | 9.89E-01 | 9.72E-01 | -0.035388 | -6.0734 | -0.03287 |
| hsa-miR-486-3p | 9.89E-01 | 9.72E-01 | -0.035388 | -6.0734 | -0.03287 |
| hsa-miR-487a-3p | 9.89E-01 | 9.72E-01 | -0.035388 | -6.0734 | -0.03287 |
| hsa-miR-487a-5p | 9.89E-01 | 9.72E-01 | -0.035388 | -6.0734 | -0.03287 |
| hsa-miR-487b-5p | 9.89E-01 | 9.72E-01 | -0.035388 | -6.0734 | -0.03287 |
| hsa-miR-488-3p | 9.89E-01 | 9.72E-01 | -0.035388 | -6.0734 | -0.03287 |
| hsa-miR-488-5p | 9.89E-01 | 9.72E-01 | -0.035388 | -6.0734 | -0.03287 |
| hsa-miR-489-3p | 9.89E-01 | 9.72E-01 | -0.035388 | -6.0734 | -0.03287 |
| hsa-miR-489-5p | 9.89E-01 | 9.72E-01 | -0.035388 | -6.0734 | -0.03287 |
| hsa-miR-490-3p | 9.89E-01 | 9.72E-01 | -0.035388 | -6.0734 | -0.03287 |
| hsa-miR-490-5p | 9.89E-01 | 9.72E-01 | -0.035388 | -6.0734 | -0.03287 |
| hsa-miR-491-3p | 9.89E-01 | 9.72E-01 | -0.035388 | -6.0734 | -0.03287 |
| hsa-miR-491-5p | 9.89E-01 | 9.72E-01 | -0.035388 | -6.0734 | -0.03287 |
| hsa-miR-492 | 9.89E-01 | 9.72E-01 | -0.035388 | -6.0734 | -0.03287 |
| hsa-miR-494-5p | 9.89E-01 | 9.72E-01 | -0.035388 | -6.0734 | -0.03287 |
| hsa-miR-495-5p | 9.89E-01 | 9.72E-01 | -0.035388 | -6.0734 | -0.03287 |
| hsa-miR-496 | 9.89E-01 | 9.72E-01 | -0.035388 | -6.0734 | -0.03287 |
| hsa-miR-497-3p | 9.89E-01 | 9.72E-01 | -0.035388 | -6.0734 | -0.03287 |
| hsa-miR-4999-3p | 9.89E-01 | 9.72E-01 | -0.035388 | -6.0734 | -0.03287 |
| hsa-miR-4999-5p | 9.89E-01 | 9.72E-01 | -0.035388 | -6.0734 | -0.03287 |
| hsa-miR-499a-3p | 9.89E-01 | 9.72E-01 | -0.035388 | -6.0734 | -0.03287 |
| hsa-miR-499b-3p | 9.89E-01 | 9.72E-01 | -0.035388 | -6.0734 | -0.03287 |
| hsa-miR-499b-5p | 9.89E-01 | 9.72E-01 | -0.035388 | -6.0734 | -0.03287 |
| hsa-miR-5000-3p | 9.89E-01 | 9.72E-01 | -0.035388 | -6.0734 | -0.03287 |
| hsa-miR-5000-5p | 9.89E-01 | 9.72E-01 | -0.035388 | -6.0734 | -0.03287 |
| hsa-miR-5001-3p | 9.89E-01 | 9.72E-01 | -0.035388 | -6.0734 | -0.03287 |
| hsa-miR-5002-3p | 9.89E-01 | 9.72E-01 | -0.035388 | -6.0734 | -0.03287 |
| hsa-miR-5002-5p | 9.89E-01 | 9.72E-01 | -0.035388 | -6.0734 | -0.03287 |
| hsa-miR-5003-3p | 9.89E-01 | 9.72E-01 | -0.035388 | -6.0734 | -0.03287 |
| hsa-miR-5003-5p | 9.89E-01 | 9.72E-01 | -0.035388 | -6.0734 | -0.03287 |
| hsa-miR-5004-3p | 9.89E-01 | 9.72E-01 | -0.035388 | -6.0734 | -0.03287 |
| hsa-miR-5004-5p | 9.89E-01 | 9.72E-01 | -0.035388 | -6.0734 | -0.03287 |
| hsa-miR-5006-3p | 9.89E-01 | 9.72E-01 | -0.035388 | -6.0734 | -0.03287 |
| hsa-miR-5007-3p | 9.89E-01 | 9.72E-01 | -0.035388 | -6.0734 | -0.03287 |
| hsa-miR-5007-5p | 9.89E-01 | 9.72E-01 | -0.035388 | -6.0734 | -0.03287 |
| hsa-miR-5008-3p | 9.89E-01 | 9.72E-01 | -0.035388 | -6.0734 | -0.03287 |
| hsa-miR-5009-3p | 9.89E-01 | 9.72E-01 | -0.035388 | -6.0734 | -0.03287 |
| hsa-miR-5009-5p | 9.89E-01 | 9.72E-01 | -0.035388 | -6.0734 | -0.03287 |
| hsa-miR-500b-3p | 9.89E-01 | 9.72E-01 | -0.035388 | -6.0734 | -0.03287 |
| hsa-miR-500b-5p | 9.89E-01 | 9.72E-01 | -0.035388 | -6.0734 | -0.03287 |
| hsa-miR-5010-3p | 9.89E-01 | 9.72E-01 | -0.035388 | -6.0734 | -0.03287 |
| hsa-miR-5011-3p | 9.89E-01 | 9.72E-01 | -0.035388 | -6.0734 | -0.03287 |
| hsa-miR-5011-5p | 9.89E-01 | 9.72E-01 | -0.035388 | -6.0734 | -0.03287 |
| hsa-miR-503-3p | 9.89E-01 | 9.72E-01 | -0.035388 | -6.0734 | -0.03287 |
| hsa-miR-504-5p | 9.89E-01 | 9.72E-01 | -0.035388 | -6.0734 | -0.03287 |
| hsa-miR-5047 | 9.89E-01 | 9.72E-01 | -0.035388 | -6.0734 | -0.03287 |
| hsa-miR-506-3p | 9.89E-01 | 9.72E-01 | -0.035388 | -6.0734 | -0.03287 |
| hsa-miR-506-5p | 9.89E-01 | 9.72E-01 | -0.035388 | -6.0734 | -0.03287 |
| hsa-miR-507 | 9.89E-01 | 9.72E-01 | -0.035388 | -6.0734 | -0.03287 |
| hsa-miR-508-3p | 9.89E-01 | 9.72E-01 | -0.035388 | -6.0734 | -0.03287 |
| hsa-miR-508-5p | 9.89E-01 | 9.72E-01 | -0.035388 | -6.0734 | -0.03287 |
| hsa-miR-5087 | 9.89E-01 | 9.72E-01 | -0.035388 | -6.0734 | -0.03287 |
| hsa-miR-5088-3p | 9.89E-01 | 9.72E-01 | -0.035388 | -6.0734 | -0.03287 |
| hsa-miR-5089-3p | 9.89E-01 | 9.72E-01 | -0.035388 | -6.0734 | -0.03287 |
| hsa-miR-5089-5p | 9.89E-01 | 9.72E-01 | -0.035388 | -6.0734 | -0.03287 |
| hsa-miR-509-3-5p | 9.89E-01 | 9.72E-01 | -0.035388 | -6.0734 | -0.03287 |
| hsa-miR-509-3p | 9.89E-01 | 9.72E-01 | -0.035388 | -6.0734 | -0.03287 |
| hsa-miR-509-5p | 9.89E-01 | 9.72E-01 | -0.035388 | -6.0734 | -0.03287 |
| hsa-miR-5090 | 9.89E-01 | 9.72E-01 | -0.035388 | -6.0734 | -0.03287 |
| hsa-miR-5091 | 9.89E-01 | 9.72E-01 | -0.035388 | -6.0734 | -0.03287 |
| hsa-miR-5092 | 9.89E-01 | 9.72E-01 | -0.035388 | -6.0734 | -0.03287 |
| hsa-miR-5093 | 9.89E-01 | 9.72E-01 | -0.035388 | -6.0734 | -0.03287 |
| hsa-miR-5094 | 9.89E-01 | 9.72E-01 | -0.035388 | -6.0734 | -0.03287 |
| hsa-miR-5095 | 9.89E-01 | 9.72E-01 | -0.035388 | -6.0734 | -0.03287 |
| hsa-miR-510-3p | 9.89E-01 | 9.72E-01 | -0.035388 | -6.0734 | -0.03287 |
| hsa-miR-510-5p | 9.89E-01 | 9.72E-01 | -0.035388 | -6.0734 | -0.03287 |
| hsa-miR-511-3p | 9.89E-01 | 9.72E-01 | -0.035388 | -6.0734 | -0.03287 |
| hsa-miR-511-5p | 9.89E-01 | 9.72E-01 | -0.035388 | -6.0734 | -0.03287 |
| hsa-miR-512-3p | 9.89E-01 | 9.72E-01 | -0.035388 | -6.0734 | -0.03287 |
| hsa-miR-512-5p | 9.89E-01 | 9.72E-01 | -0.035388 | -6.0734 | -0.03287 |
| hsa-miR-513a-3p | 9.89E-01 | 9.72E-01 | -0.035388 | -6.0734 | -0.03287 |
| hsa-miR-513b-3p | 9.89E-01 | 9.72E-01 | -0.035388 | -6.0734 | -0.03287 |
| hsa-miR-513c-3p | 9.89E-01 | 9.72E-01 | -0.035388 | -6.0734 | -0.03287 |
| hsa-miR-514a-3p | 9.89E-01 | 9.72E-01 | -0.035388 | -6.0734 | -0.03287 |
| hsa-miR-514a-5p | 9.89E-01 | 9.72E-01 | -0.035388 | -6.0734 | -0.03287 |
| hsa-miR-514b-3p | 9.89E-01 | 9.72E-01 | -0.035388 | -6.0734 | -0.03287 |
| hsa-miR-515-3p | 9.89E-01 | 9.72E-01 | -0.035388 | -6.0734 | -0.03287 |
| hsa-miR-515-5p | 9.89E-01 | 9.72E-01 | -0.035388 | -6.0734 | -0.03287 |
| hsa-miR-516a-3p | 9.89E-01 | 9.72E-01 | -0.035388 | -6.0734 | -0.03287 |
| hsa-miR-516a-5p | 9.89E-01 | 9.72E-01 | -0.035388 | -6.0734 | -0.03287 |
| hsa-miR-516b-5p | 9.89E-01 | 9.72E-01 | -0.035388 | -6.0734 | -0.03287 |
| hsa-miR-517-5p | 9.89E-01 | 9.72E-01 | -0.035388 | -6.0734 | -0.03287 |
| hsa-miR-517a-3p | 9.89E-01 | 9.72E-01 | -0.035388 | -6.0734 | -0.03287 |
| hsa-miR-517c-3p | 9.89E-01 | 9.72E-01 | -0.035388 | -6.0734 | -0.03287 |
| hsa-miR-5186 | 9.89E-01 | 9.72E-01 | -0.035388 | -6.0734 | -0.03287 |
| hsa-miR-5187-3p | 9.89E-01 | 9.72E-01 | -0.035388 | -6.0734 | -0.03287 |
| hsa-miR-5187-5p | 9.89E-01 | 9.72E-01 | -0.035388 | -6.0734 | -0.03287 |
| hsa-miR-5188 | 9.89E-01 | 9.72E-01 | -0.035388 | -6.0734 | -0.03287 |
| hsa-miR-5189-3p | 9.89E-01 | 9.72E-01 | -0.035388 | -6.0734 | -0.03287 |
| hsa-miR-518a-3p | 9.89E-01 | 9.72E-01 | -0.035388 | -6.0734 | -0.03287 |
| hsa-miR-518a-5p | 9.89E-01 | 9.72E-01 | -0.035388 | -6.0734 | -0.03287 |
| hsa-miR-518b | 9.89E-01 | 9.72E-01 | -0.035388 | -6.0734 | -0.03287 |
| hsa-miR-518c-3p | 9.89E-01 | 9.72E-01 | -0.035388 | -6.0734 | -0.03287 |
| hsa-miR-518c-5p | 9.89E-01 | 9.72E-01 | -0.035388 | -6.0734 | -0.03287 |
| hsa-miR-518d-3p | 9.89E-01 | 9.72E-01 | -0.035388 | -6.0734 | -0.03287 |
| hsa-miR-518e-3p | 9.89E-01 | 9.72E-01 | -0.035388 | -6.0734 | -0.03287 |
| hsa-miR-518e-5p | 9.89E-01 | 9.72E-01 | -0.035388 | -6.0734 | -0.03287 |
| hsa-miR-518f-3p | 9.89E-01 | 9.72E-01 | -0.035388 | -6.0734 | -0.03287 |
| hsa-miR-518f-5p | 9.89E-01 | 9.72E-01 | -0.035388 | -6.0734 | -0.03287 |
| hsa-miR-5191 | 9.89E-01 | 9.72E-01 | -0.035388 | -6.0734 | -0.03287 |
| hsa-miR-5192 | 9.89E-01 | 9.72E-01 | -0.035388 | -6.0734 | -0.03287 |
| hsa-miR-5193 | 9.89E-01 | 9.72E-01 | -0.035388 | -6.0734 | -0.03287 |
| hsa-miR-5195-5p | 9.89E-01 | 9.72E-01 | -0.035388 | -6.0734 | -0.03287 |
| hsa-miR-5196-3p | 9.89E-01 | 9.72E-01 | -0.035388 | -6.0734 | -0.03287 |
| hsa-miR-5197-3p | 9.89E-01 | 9.72E-01 | -0.035388 | -6.0734 | -0.03287 |
| hsa-miR-5197-5p | 9.89E-01 | 9.72E-01 | -0.035388 | -6.0734 | -0.03287 |
| hsa-miR-519b-3p | 9.89E-01 | 9.72E-01 | -0.035388 | -6.0734 | -0.03287 |
| hsa-miR-519c-3p | 9.89E-01 | 9.72E-01 | -0.035388 | -6.0734 | -0.03287 |
| hsa-miR-519d-3p | 9.89E-01 | 9.72E-01 | -0.035388 | -6.0734 | -0.03287 |
| hsa-miR-519d-5p | 9.89E-01 | 9.72E-01 | -0.035388 | -6.0734 | -0.03287 |
| hsa-miR-519e-3p | 9.89E-01 | 9.72E-01 | -0.035388 | -6.0734 | -0.03287 |
| hsa-miR-519e-5p | 9.89E-01 | 9.72E-01 | -0.035388 | -6.0734 | -0.03287 |
| hsa-miR-520a-3p | 9.89E-01 | 9.72E-01 | -0.035388 | -6.0734 | -0.03287 |
| hsa-miR-520a-5p | 9.89E-01 | 9.72E-01 | -0.035388 | -6.0734 | -0.03287 |
| hsa-miR-520b | 9.89E-01 | 9.72E-01 | -0.035388 | -6.0734 | -0.03287 |
| hsa-miR-520c-3p | 9.89E-01 | 9.72E-01 | -0.035388 | -6.0734 | -0.03287 |
| hsa-miR-520d-3p | 9.89E-01 | 9.72E-01 | -0.035388 | -6.0734 | -0.03287 |
| hsa-miR-520e | 9.89E-01 | 9.72E-01 | -0.035388 | -6.0734 | -0.03287 |
| hsa-miR-520f-3p | 9.89E-01 | 9.72E-01 | -0.035388 | -6.0734 | -0.03287 |
| hsa-miR-520f-5p | 9.89E-01 | 9.72E-01 | -0.035388 | -6.0734 | -0.03287 |
| hsa-miR-520g-3p | 9.89E-01 | 9.72E-01 | -0.035388 | -6.0734 | -0.03287 |
| hsa-miR-520g-5p | 9.89E-01 | 9.72E-01 | -0.035388 | -6.0734 | -0.03287 |
| hsa-miR-520h | 9.89E-01 | 9.72E-01 | -0.035388 | -6.0734 | -0.03287 |
| hsa-miR-521 | 9.89E-01 | 9.72E-01 | -0.035388 | -6.0734 | -0.03287 |
| hsa-miR-522-3p | 9.89E-01 | 9.72E-01 | -0.035388 | -6.0734 | -0.03287 |
| hsa-miR-523-3p | 9.89E-01 | 9.72E-01 | -0.035388 | -6.0734 | -0.03287 |
| hsa-miR-524-3p | 9.89E-01 | 9.72E-01 | -0.035388 | -6.0734 | -0.03287 |
| hsa-miR-525-3p | 9.89E-01 | 9.72E-01 | -0.035388 | -6.0734 | -0.03287 |
| hsa-miR-525-5p | 9.89E-01 | 9.72E-01 | -0.035388 | -6.0734 | -0.03287 |
| hsa-miR-526b-3p | 9.89E-01 | 9.72E-01 | -0.035388 | -6.0734 | -0.03287 |
| hsa-miR-539-3p | 9.89E-01 | 9.72E-01 | -0.035388 | -6.0734 | -0.03287 |
| hsa-miR-539-5p | 9.89E-01 | 9.72E-01 | -0.035388 | -6.0734 | -0.03287 |
| hsa-miR-541-3p | 9.89E-01 | 9.72E-01 | -0.035388 | -6.0734 | -0.03287 |
| hsa-miR-541-5p | 9.89E-01 | 9.72E-01 | -0.035388 | -6.0734 | -0.03287 |
| hsa-miR-543 | 9.89E-01 | 9.72E-01 | -0.035388 | -6.0734 | -0.03287 |
| hsa-miR-544a | 9.89E-01 | 9.72E-01 | -0.035388 | -6.0734 | -0.03287 |
| hsa-miR-544b | 9.89E-01 | 9.72E-01 | -0.035388 | -6.0734 | -0.03287 |
| hsa-miR-545-3p | 9.89E-01 | 9.72E-01 | -0.035388 | -6.0734 | -0.03287 |
| hsa-miR-545-5p | 9.89E-01 | 9.72E-01 | -0.035388 | -6.0734 | -0.03287 |
| hsa-miR-548a-3p | 9.89E-01 | 9.72E-01 | -0.035388 | -6.0734 | -0.03287 |
| hsa-miR-548a-5p | 9.89E-01 | 9.72E-01 | -0.035388 | -6.0734 | -0.03287 |
| hsa-miR-548aa | 9.89E-01 | 9.72E-01 | -0.035388 | -6.0734 | -0.03287 |
| hsa-miR-548ab | 9.89E-01 | 9.72E-01 | -0.035388 | -6.0734 | -0.03287 |
| hsa-miR-548ac | 9.89E-01 | 9.72E-01 | -0.035388 | -6.0734 | -0.03287 |
| hsa-miR-548ad-3p | 9.89E-01 | 9.72E-01 | -0.035388 | -6.0734 | -0.03287 |
| hsa-miR-548ad-5p | 9.89E-01 | 9.72E-01 | -0.035388 | -6.0734 | -0.03287 |
| hsa-miR-548ae-3p | 9.89E-01 | 9.72E-01 | -0.035388 | -6.0734 | -0.03287 |
| hsa-miR-548ag | 9.89E-01 | 9.72E-01 | -0.035388 | -6.0734 | -0.03287 |
| hsa-miR-548ah-5p | 9.89E-01 | 9.72E-01 | -0.035388 | -6.0734 | -0.03287 |
| hsa-miR-548ai | 9.89E-01 | 9.72E-01 | -0.035388 | -6.0734 | -0.03287 |
| hsa-miR-548aj-3p | 9.89E-01 | 9.72E-01 | -0.035388 | -6.0734 | -0.03287 |
| hsa-miR-548aj-5p | 9.89E-01 | 9.72E-01 | -0.035388 | -6.0734 | -0.03287 |
| hsa-miR-548ak | 9.89E-01 | 9.72E-01 | -0.035388 | -6.0734 | -0.03287 |
| hsa-miR-548al | 9.89E-01 | 9.72E-01 | -0.035388 | -6.0734 | -0.03287 |
| hsa-miR-548am-3p | 9.89E-01 | 9.72E-01 | -0.035388 | -6.0734 | -0.03287 |
| hsa-miR-548am-5p | 9.89E-01 | 9.72E-01 | -0.035388 | -6.0734 | -0.03287 |
| hsa-miR-548an | 9.89E-01 | 9.72E-01 | -0.035388 | -6.0734 | -0.03287 |
| hsa-miR-548ao-3p | 9.89E-01 | 9.72E-01 | -0.035388 | -6.0734 | -0.03287 |
| hsa-miR-548ao-5p | 9.89E-01 | 9.72E-01 | -0.035388 | -6.0734 | -0.03287 |
| hsa-miR-548ap-3p | 9.89E-01 | 9.72E-01 | -0.035388 | -6.0734 | -0.03287 |
| hsa-miR-548ap-5p | 9.89E-01 | 9.72E-01 | -0.035388 | -6.0734 | -0.03287 |
| hsa-miR-548aq-5p | 9.89E-01 | 9.72E-01 | -0.035388 | -6.0734 | -0.03287 |
| hsa-miR-548ar-3p | 9.89E-01 | 9.72E-01 | -0.035388 | -6.0734 | -0.03287 |
| hsa-miR-548ar-5p | 9.89E-01 | 9.72E-01 | -0.035388 | -6.0734 | -0.03287 |
| hsa-miR-548as-3p | 9.89E-01 | 9.72E-01 | -0.035388 | -6.0734 | -0.03287 |
| hsa-miR-548as-5p | 9.89E-01 | 9.72E-01 | -0.035388 | -6.0734 | -0.03287 |
| hsa-miR-548at-3p | 9.89E-01 | 9.72E-01 | -0.035388 | -6.0734 | -0.03287 |
| hsa-miR-548at-5p | 9.89E-01 | 9.72E-01 | -0.035388 | -6.0734 | -0.03287 |
| hsa-miR-548au-3p | 9.89E-01 | 9.72E-01 | -0.035388 | -6.0734 | -0.03287 |
| hsa-miR-548au-5p | 9.89E-01 | 9.72E-01 | -0.035388 | -6.0734 | -0.03287 |
| hsa-miR-548av-3p | 9.89E-01 | 9.72E-01 | -0.035388 | -6.0734 | -0.03287 |
| hsa-miR-548av-5p | 9.89E-01 | 9.72E-01 | -0.035388 | -6.0734 | -0.03287 |
| hsa-miR-548aw | 9.89E-01 | 9.72E-01 | -0.035388 | -6.0734 | -0.03287 |
| hsa-miR-548ax | 9.89E-01 | 9.72E-01 | -0.035388 | -6.0734 | -0.03287 |
| hsa-miR-548ay-3p | 9.89E-01 | 9.72E-01 | -0.035388 | -6.0734 | -0.03287 |
| hsa-miR-548ay-5p | 9.89E-01 | 9.72E-01 | -0.035388 | -6.0734 | -0.03287 |
| hsa-miR-548az-3p | 9.89E-01 | 9.72E-01 | -0.035388 | -6.0734 | -0.03287 |
| hsa-miR-548az-5p | 9.89E-01 | 9.72E-01 | -0.035388 | -6.0734 | -0.03287 |
| hsa-miR-548b-3p | 9.89E-01 | 9.72E-01 | -0.035388 | -6.0734 | -0.03287 |
| hsa-miR-548b-5p | 9.89E-01 | 9.72E-01 | -0.035388 | -6.0734 | -0.03287 |
| hsa-miR-548ba | 9.89E-01 | 9.72E-01 | -0.035388 | -6.0734 | -0.03287 |
| hsa-miR-548bb-3p | 9.89E-01 | 9.72E-01 | -0.035388 | -6.0734 | -0.03287 |
| hsa-miR-548bb-5p | 9.89E-01 | 9.72E-01 | -0.035388 | -6.0734 | -0.03287 |
| hsa-miR-548c-3p | 9.89E-01 | 9.72E-01 | -0.035388 | -6.0734 | -0.03287 |
| hsa-miR-548d-3p | 9.89E-01 | 9.72E-01 | -0.035388 | -6.0734 | -0.03287 |
| hsa-miR-548d-5p | 9.89E-01 | 9.72E-01 | -0.035388 | -6.0734 | -0.03287 |
| hsa-miR-548e-3p | 9.89E-01 | 9.72E-01 | -0.035388 | -6.0734 | -0.03287 |
| hsa-miR-548e-5p | 9.89E-01 | 9.72E-01 | -0.035388 | -6.0734 | -0.03287 |
| hsa-miR-548f-3p | 9.89E-01 | 9.72E-01 | -0.035388 | -6.0734 | -0.03287 |
| hsa-miR-548f-5p | 9.89E-01 | 9.72E-01 | -0.035388 | -6.0734 | -0.03287 |
| hsa-miR-548g-3p | 9.89E-01 | 9.72E-01 | -0.035388 | -6.0734 | -0.03287 |
| hsa-miR-548h-3p | 9.89E-01 | 9.72E-01 | -0.035388 | -6.0734 | -0.03287 |
| hsa-miR-548h-5p | 9.89E-01 | 9.72E-01 | -0.035388 | -6.0734 | -0.03287 |
| hsa-miR-548i | 9.89E-01 | 9.72E-01 | -0.035388 | -6.0734 | -0.03287 |
| hsa-miR-548j-3p | 9.89E-01 | 9.72E-01 | -0.035388 | -6.0734 | -0.03287 |
| hsa-miR-548j-5p | 9.89E-01 | 9.72E-01 | -0.035388 | -6.0734 | -0.03287 |
| hsa-miR-548k | 9.89E-01 | 9.72E-01 | -0.035388 | -6.0734 | -0.03287 |
| hsa-miR-548l | 9.89E-01 | 9.72E-01 | -0.035388 | -6.0734 | -0.03287 |
| hsa-miR-548m | 9.89E-01 | 9.72E-01 | -0.035388 | -6.0734 | -0.03287 |
| hsa-miR-548n | 9.89E-01 | 9.72E-01 | -0.035388 | -6.0734 | -0.03287 |
| hsa-miR-548p | 9.89E-01 | 9.72E-01 | -0.035388 | -6.0734 | -0.03287 |
| hsa-miR-548s | 9.89E-01 | 9.72E-01 | -0.035388 | -6.0734 | -0.03287 |
| hsa-miR-548t-5p | 9.89E-01 | 9.72E-01 | -0.035388 | -6.0734 | -0.03287 |
| hsa-miR-548u | 9.89E-01 | 9.72E-01 | -0.035388 | -6.0734 | -0.03287 |
| hsa-miR-548v | 9.89E-01 | 9.72E-01 | -0.035388 | -6.0734 | -0.03287 |
| hsa-miR-548w | 9.89E-01 | 9.72E-01 | -0.035388 | -6.0734 | -0.03287 |
| hsa-miR-548x-3p | 9.89E-01 | 9.72E-01 | -0.035388 | -6.0734 | -0.03287 |
| hsa-miR-548y | 9.89E-01 | 9.72E-01 | -0.035388 | -6.0734 | -0.03287 |
| hsa-miR-549a | 9.89E-01 | 9.72E-01 | -0.035388 | -6.0734 | -0.03287 |
| hsa-miR-550b-2-5p | 9.89E-01 | 9.72E-01 | -0.035388 | -6.0734 | -0.03287 |
| hsa-miR-550b-3p | 9.89E-01 | 9.72E-01 | -0.035388 | -6.0734 | -0.03287 |
| hsa-miR-551a | 9.89E-01 | 9.72E-01 | -0.035388 | -6.0734 | -0.03287 |
| hsa-miR-551b-5p | 9.89E-01 | 9.72E-01 | -0.035388 | -6.0734 | -0.03287 |
| hsa-miR-552-5p | 9.89E-01 | 9.72E-01 | -0.035388 | -6.0734 | -0.03287 |
| hsa-miR-553 | 9.89E-01 | 9.72E-01 | -0.035388 | -6.0734 | -0.03287 |
| hsa-miR-554 | 9.89E-01 | 9.72E-01 | -0.035388 | -6.0734 | -0.03287 |
| hsa-miR-555 | 9.89E-01 | 9.72E-01 | -0.035388 | -6.0734 | -0.03287 |
| hsa-miR-556-3p | 9.89E-01 | 9.72E-01 | -0.035388 | -6.0734 | -0.03287 |
| hsa-miR-556-5p | 9.89E-01 | 9.72E-01 | -0.035388 | -6.0734 | -0.03287 |
| hsa-miR-5571-3p | 9.89E-01 | 9.72E-01 | -0.035388 | -6.0734 | -0.03287 |
| hsa-miR-5571-5p | 9.89E-01 | 9.72E-01 | -0.035388 | -6.0734 | -0.03287 |
| hsa-miR-5572 | 9.89E-01 | 9.72E-01 | -0.035388 | -6.0734 | -0.03287 |
| hsa-miR-5579-3p | 9.89E-01 | 9.72E-01 | -0.035388 | -6.0734 | -0.03287 |
| hsa-miR-5579-5p | 9.89E-01 | 9.72E-01 | -0.035388 | -6.0734 | -0.03287 |
| hsa-miR-558 | 9.89E-01 | 9.72E-01 | -0.035388 | -6.0734 | -0.03287 |
| hsa-miR-5580-5p | 9.89E-01 | 9.72E-01 | -0.035388 | -6.0734 | -0.03287 |
| hsa-miR-5581-3p | 9.89E-01 | 9.72E-01 | -0.035388 | -6.0734 | -0.03287 |
| hsa-miR-5582-3p | 9.89E-01 | 9.72E-01 | -0.035388 | -6.0734 | -0.03287 |
| hsa-miR-5582-5p | 9.89E-01 | 9.72E-01 | -0.035388 | -6.0734 | -0.03287 |
| hsa-miR-5583-3p | 9.89E-01 | 9.72E-01 | -0.035388 | -6.0734 | -0.03287 |
| hsa-miR-5583-5p | 9.89E-01 | 9.72E-01 | -0.035388 | -6.0734 | -0.03287 |
| hsa-miR-5584-3p | 9.89E-01 | 9.72E-01 | -0.035388 | -6.0734 | -0.03287 |
| hsa-miR-5584-5p | 9.89E-01 | 9.72E-01 | -0.035388 | -6.0734 | -0.03287 |
| hsa-miR-5585-5p | 9.89E-01 | 9.72E-01 | -0.035388 | -6.0734 | -0.03287 |
| hsa-miR-5586-3p | 9.89E-01 | 9.72E-01 | -0.035388 | -6.0734 | -0.03287 |
| hsa-miR-5586-5p | 9.89E-01 | 9.72E-01 | -0.035388 | -6.0734 | -0.03287 |
| hsa-miR-5587-3p | 9.89E-01 | 9.72E-01 | -0.035388 | -6.0734 | -0.03287 |
| hsa-miR-5587-5p | 9.89E-01 | 9.72E-01 | -0.035388 | -6.0734 | -0.03287 |
| hsa-miR-5588-3p | 9.89E-01 | 9.72E-01 | -0.035388 | -6.0734 | -0.03287 |
| hsa-miR-5588-5p | 9.89E-01 | 9.72E-01 | -0.035388 | -6.0734 | -0.03287 |
| hsa-miR-5589-3p | 9.89E-01 | 9.72E-01 | -0.035388 | -6.0734 | -0.03287 |
| hsa-miR-5589-5p | 9.89E-01 | 9.72E-01 | -0.035388 | -6.0734 | -0.03287 |
| hsa-miR-559 | 9.89E-01 | 9.72E-01 | -0.035388 | -6.0734 | -0.03287 |
| hsa-miR-5590-3p | 9.89E-01 | 9.72E-01 | -0.035388 | -6.0734 | -0.03287 |
| hsa-miR-5590-5p | 9.89E-01 | 9.72E-01 | -0.035388 | -6.0734 | -0.03287 |
| hsa-miR-5591-3p | 9.89E-01 | 9.72E-01 | -0.035388 | -6.0734 | -0.03287 |
| hsa-miR-5591-5p | 9.89E-01 | 9.72E-01 | -0.035388 | -6.0734 | -0.03287 |
| hsa-miR-561-3p | 9.89E-01 | 9.72E-01 | -0.035388 | -6.0734 | -0.03287 |
| hsa-miR-561-5p | 9.89E-01 | 9.72E-01 | -0.035388 | -6.0734 | -0.03287 |
| hsa-miR-562 | 9.89E-01 | 9.72E-01 | -0.035388 | -6.0734 | -0.03287 |
| hsa-miR-563 | 9.89E-01 | 9.72E-01 | -0.035388 | -6.0734 | -0.03287 |
| hsa-miR-567 | 9.89E-01 | 9.72E-01 | -0.035388 | -6.0734 | -0.03287 |
| hsa-miR-568 | 9.89E-01 | 9.72E-01 | -0.035388 | -6.0734 | -0.03287 |
| hsa-miR-5680 | 9.89E-01 | 9.72E-01 | -0.035388 | -6.0734 | -0.03287 |
| hsa-miR-5681a | 9.89E-01 | 9.72E-01 | -0.035388 | -6.0734 | -0.03287 |
| hsa-miR-5681b | 9.89E-01 | 9.72E-01 | -0.035388 | -6.0734 | -0.03287 |
| hsa-miR-5682 | 9.89E-01 | 9.72E-01 | -0.035388 | -6.0734 | -0.03287 |
| hsa-miR-5683 | 9.89E-01 | 9.72E-01 | -0.035388 | -6.0734 | -0.03287 |
| hsa-miR-5685 | 9.89E-01 | 9.72E-01 | -0.035388 | -6.0734 | -0.03287 |
| hsa-miR-5687 | 9.89E-01 | 9.72E-01 | -0.035388 | -6.0734 | -0.03287 |
| hsa-miR-5688 | 9.89E-01 | 9.72E-01 | -0.035388 | -6.0734 | -0.03287 |
| hsa-miR-5689 | 9.89E-01 | 9.72E-01 | -0.035388 | -6.0734 | -0.03287 |
| hsa-miR-569 | 9.89E-01 | 9.72E-01 | -0.035388 | -6.0734 | -0.03287 |
| hsa-miR-5690 | 9.89E-01 | 9.72E-01 | -0.035388 | -6.0734 | -0.03287 |
| hsa-miR-5691 | 9.89E-01 | 9.72E-01 | -0.035388 | -6.0734 | -0.03287 |
| hsa-miR-5692a | 9.89E-01 | 9.72E-01 | -0.035388 | -6.0734 | -0.03287 |
| hsa-miR-5692b | 9.89E-01 | 9.72E-01 | -0.035388 | -6.0734 | -0.03287 |
| hsa-miR-5692c | 9.89E-01 | 9.72E-01 | -0.035388 | -6.0734 | -0.03287 |
| hsa-miR-5693 | 9.89E-01 | 9.72E-01 | -0.035388 | -6.0734 | -0.03287 |
| hsa-miR-5694 | 9.89E-01 | 9.72E-01 | -0.035388 | -6.0734 | -0.03287 |
| hsa-miR-5695 | 9.89E-01 | 9.72E-01 | -0.035388 | -6.0734 | -0.03287 |
| hsa-miR-5696 | 9.89E-01 | 9.72E-01 | -0.035388 | -6.0734 | -0.03287 |
| hsa-miR-5697 | 9.89E-01 | 9.72E-01 | -0.035388 | -6.0734 | -0.03287 |
| hsa-miR-5698 | 9.89E-01 | 9.72E-01 | -0.035388 | -6.0734 | -0.03287 |
| hsa-miR-5699-3p | 9.89E-01 | 9.72E-01 | -0.035388 | -6.0734 | -0.03287 |
| hsa-miR-570-3p | 9.89E-01 | 9.72E-01 | -0.035388 | -6.0734 | -0.03287 |
| hsa-miR-5700 | 9.89E-01 | 9.72E-01 | -0.035388 | -6.0734 | -0.03287 |
| hsa-miR-5701 | 9.89E-01 | 9.72E-01 | -0.035388 | -6.0734 | -0.03287 |
| hsa-miR-5702 | 9.89E-01 | 9.72E-01 | -0.035388 | -6.0734 | -0.03287 |
| hsa-miR-5704 | 9.89E-01 | 9.72E-01 | -0.035388 | -6.0734 | -0.03287 |
| hsa-miR-5705 | 9.89E-01 | 9.72E-01 | -0.035388 | -6.0734 | -0.03287 |
| hsa-miR-5706 | 9.89E-01 | 9.72E-01 | -0.035388 | -6.0734 | -0.03287 |
| hsa-miR-5707 | 9.89E-01 | 9.72E-01 | -0.035388 | -6.0734 | -0.03287 |
| hsa-miR-5708 | 9.89E-01 | 9.72E-01 | -0.035388 | -6.0734 | -0.03287 |
| hsa-miR-571 | 9.89E-01 | 9.72E-01 | -0.035388 | -6.0734 | -0.03287 |
| hsa-miR-573 | 9.89E-01 | 9.72E-01 | -0.035388 | -6.0734 | -0.03287 |
| hsa-miR-576-3p | 9.89E-01 | 9.72E-01 | -0.035388 | -6.0734 | -0.03287 |
| hsa-miR-576-5p | 9.89E-01 | 9.72E-01 | -0.035388 | -6.0734 | -0.03287 |
| hsa-miR-577 | 9.89E-01 | 9.72E-01 | -0.035388 | -6.0734 | -0.03287 |
| hsa-miR-578 | 9.89E-01 | 9.72E-01 | -0.035388 | -6.0734 | -0.03287 |
| hsa-miR-579-3p | 9.89E-01 | 9.72E-01 | -0.035388 | -6.0734 | -0.03287 |
| hsa-miR-579-5p | 9.89E-01 | 9.72E-01 | -0.035388 | -6.0734 | -0.03287 |
| hsa-miR-580-3p | 9.89E-01 | 9.72E-01 | -0.035388 | -6.0734 | -0.03287 |
| hsa-miR-580-5p | 9.89E-01 | 9.72E-01 | -0.035388 | -6.0734 | -0.03287 |
| hsa-miR-581 | 9.89E-01 | 9.72E-01 | -0.035388 | -6.0734 | -0.03287 |
| hsa-miR-582-3p | 9.89E-01 | 9.72E-01 | -0.035388 | -6.0734 | -0.03287 |
| hsa-miR-583 | 9.89E-01 | 9.72E-01 | -0.035388 | -6.0734 | -0.03287 |
| hsa-miR-584-3p | 9.89E-01 | 9.72E-01 | -0.035388 | -6.0734 | -0.03287 |
| hsa-miR-585-3p | 9.89E-01 | 9.72E-01 | -0.035388 | -6.0734 | -0.03287 |
| hsa-miR-585-5p | 9.89E-01 | 9.72E-01 | -0.035388 | -6.0734 | -0.03287 |
| hsa-miR-586 | 9.89E-01 | 9.72E-01 | -0.035388 | -6.0734 | -0.03287 |
| hsa-miR-587 | 9.89E-01 | 9.72E-01 | -0.035388 | -6.0734 | -0.03287 |
| hsa-miR-588 | 9.89E-01 | 9.72E-01 | -0.035388 | -6.0734 | -0.03287 |
| hsa-miR-589-3p | 9.89E-01 | 9.72E-01 | -0.035388 | -6.0734 | -0.03287 |
| hsa-miR-589-5p | 9.89E-01 | 9.72E-01 | -0.035388 | -6.0734 | -0.03287 |
| hsa-miR-590-3p | 9.89E-01 | 9.72E-01 | -0.035388 | -6.0734 | -0.03287 |
| hsa-miR-591 | 9.89E-01 | 9.72E-01 | -0.035388 | -6.0734 | -0.03287 |
| hsa-miR-593-3p | 9.89E-01 | 9.72E-01 | -0.035388 | -6.0734 | -0.03287 |
| hsa-miR-593-5p | 9.89E-01 | 9.72E-01 | -0.035388 | -6.0734 | -0.03287 |
| hsa-miR-596 | 9.89E-01 | 9.72E-01 | -0.035388 | -6.0734 | -0.03287 |
| hsa-miR-597-3p | 9.89E-01 | 9.72E-01 | -0.035388 | -6.0734 | -0.03287 |
| hsa-miR-597-5p | 9.89E-01 | 9.72E-01 | -0.035388 | -6.0734 | -0.03287 |
| hsa-miR-599 | 9.89E-01 | 9.72E-01 | -0.035388 | -6.0734 | -0.03287 |
| hsa-miR-600 | 9.89E-01 | 9.72E-01 | -0.035388 | -6.0734 | -0.03287 |
| hsa-miR-603 | 9.89E-01 | 9.72E-01 | -0.035388 | -6.0734 | -0.03287 |
| hsa-miR-604 | 9.89E-01 | 9.72E-01 | -0.035388 | -6.0734 | -0.03287 |
| hsa-miR-605-3p | 9.89E-01 | 9.72E-01 | -0.035388 | -6.0734 | -0.03287 |
| hsa-miR-606 | 9.89E-01 | 9.72E-01 | -0.035388 | -6.0734 | -0.03287 |
| hsa-miR-607 | 9.89E-01 | 9.72E-01 | -0.035388 | -6.0734 | -0.03287 |
| hsa-miR-6070 | 9.89E-01 | 9.72E-01 | -0.035388 | -6.0734 | -0.03287 |
| hsa-miR-6071 | 9.89E-01 | 9.72E-01 | -0.035388 | -6.0734 | -0.03287 |
| hsa-miR-6072 | 9.89E-01 | 9.72E-01 | -0.035388 | -6.0734 | -0.03287 |
| hsa-miR-6074 | 9.89E-01 | 9.72E-01 | -0.035388 | -6.0734 | -0.03287 |
| hsa-miR-6077 | 9.89E-01 | 9.72E-01 | -0.035388 | -6.0734 | -0.03287 |
| hsa-miR-6078 | 9.89E-01 | 9.72E-01 | -0.035388 | -6.0734 | -0.03287 |
| hsa-miR-6079 | 9.89E-01 | 9.72E-01 | -0.035388 | -6.0734 | -0.03287 |
| hsa-miR-608 | 9.89E-01 | 9.72E-01 | -0.035388 | -6.0734 | -0.03287 |
| hsa-miR-6080 | 9.89E-01 | 9.72E-01 | -0.035388 | -6.0734 | -0.03287 |
| hsa-miR-6081 | 9.89E-01 | 9.72E-01 | -0.035388 | -6.0734 | -0.03287 |
| hsa-miR-6082 | 9.89E-01 | 9.72E-01 | -0.035388 | -6.0734 | -0.03287 |
| hsa-miR-6084 | 9.89E-01 | 9.72E-01 | -0.035388 | -6.0734 | -0.03287 |
| hsa-miR-609 | 9.89E-01 | 9.72E-01 | -0.035388 | -6.0734 | -0.03287 |
| hsa-miR-611 | 9.89E-01 | 9.72E-01 | -0.035388 | -6.0734 | -0.03287 |
| hsa-miR-612 | 9.89E-01 | 9.72E-01 | -0.035388 | -6.0734 | -0.03287 |
| hsa-miR-6128 | 9.89E-01 | 9.72E-01 | -0.035388 | -6.0734 | -0.03287 |
| hsa-miR-613 | 9.89E-01 | 9.72E-01 | -0.035388 | -6.0734 | -0.03287 |
| hsa-miR-6130 | 9.89E-01 | 9.72E-01 | -0.035388 | -6.0734 | -0.03287 |
| hsa-miR-6134 | 9.89E-01 | 9.72E-01 | -0.035388 | -6.0734 | -0.03287 |
| hsa-miR-614 | 9.89E-01 | 9.72E-01 | -0.035388 | -6.0734 | -0.03287 |
| hsa-miR-615-3p | 9.89E-01 | 9.72E-01 | -0.035388 | -6.0734 | -0.03287 |
| hsa-miR-615-5p | 9.89E-01 | 9.72E-01 | -0.035388 | -6.0734 | -0.03287 |
| hsa-miR-616-3p | 9.89E-01 | 9.72E-01 | -0.035388 | -6.0734 | -0.03287 |
| hsa-miR-616-5p | 9.89E-01 | 9.72E-01 | -0.035388 | -6.0734 | -0.03287 |
| hsa-miR-617 | 9.89E-01 | 9.72E-01 | -0.035388 | -6.0734 | -0.03287 |
| hsa-miR-618 | 9.89E-01 | 9.72E-01 | -0.035388 | -6.0734 | -0.03287 |
| hsa-miR-619-3p | 9.89E-01 | 9.72E-01 | -0.035388 | -6.0734 | -0.03287 |
| hsa-miR-620 | 9.89E-01 | 9.72E-01 | -0.035388 | -6.0734 | -0.03287 |
| hsa-miR-621 | 9.89E-01 | 9.72E-01 | -0.035388 | -6.0734 | -0.03287 |
| hsa-miR-624-3p | 9.89E-01 | 9.72E-01 | -0.035388 | -6.0734 | -0.03287 |
| hsa-miR-624-5p | 9.89E-01 | 9.72E-01 | -0.035388 | -6.0734 | -0.03287 |
| hsa-miR-625-3p | 9.89E-01 | 9.72E-01 | -0.035388 | -6.0734 | -0.03287 |
| hsa-miR-626 | 9.89E-01 | 9.72E-01 | -0.035388 | -6.0734 | -0.03287 |
| hsa-miR-627-3p | 9.89E-01 | 9.72E-01 | -0.035388 | -6.0734 | -0.03287 |
| hsa-miR-627-5p | 9.89E-01 | 9.72E-01 | -0.035388 | -6.0734 | -0.03287 |
| hsa-miR-628-3p | 9.89E-01 | 9.72E-01 | -0.035388 | -6.0734 | -0.03287 |
| hsa-miR-628-5p | 9.89E-01 | 9.72E-01 | -0.035388 | -6.0734 | -0.03287 |
| hsa-miR-632 | 9.89E-01 | 9.72E-01 | -0.035388 | -6.0734 | -0.03287 |
| hsa-miR-633 | 9.89E-01 | 9.72E-01 | -0.035388 | -6.0734 | -0.03287 |
| hsa-miR-634 | 9.89E-01 | 9.72E-01 | -0.035388 | -6.0734 | -0.03287 |
| hsa-miR-635 | 9.89E-01 | 9.72E-01 | -0.035388 | -6.0734 | -0.03287 |
| hsa-miR-636 | 9.89E-01 | 9.72E-01 | -0.035388 | -6.0734 | -0.03287 |
| hsa-miR-637 | 9.89E-01 | 9.72E-01 | -0.035388 | -6.0734 | -0.03287 |
| hsa-miR-639 | 9.89E-01 | 9.72E-01 | -0.035388 | -6.0734 | -0.03287 |
| hsa-miR-640 | 9.89E-01 | 9.72E-01 | -0.035388 | -6.0734 | -0.03287 |
| hsa-miR-641 | 9.89E-01 | 9.72E-01 | -0.035388 | -6.0734 | -0.03287 |
| hsa-miR-642a-5p | 9.89E-01 | 9.72E-01 | -0.035388 | -6.0734 | -0.03287 |
| hsa-miR-642b-5p | 9.89E-01 | 9.72E-01 | -0.035388 | -6.0734 | -0.03287 |
| hsa-miR-643 | 9.89E-01 | 9.72E-01 | -0.035388 | -6.0734 | -0.03287 |
| hsa-miR-644a | 9.89E-01 | 9.72E-01 | -0.035388 | -6.0734 | -0.03287 |
| hsa-miR-646 | 9.89E-01 | 9.72E-01 | -0.035388 | -6.0734 | -0.03287 |
| hsa-miR-647 | 9.89E-01 | 9.72E-01 | -0.035388 | -6.0734 | -0.03287 |
| hsa-miR-649 | 9.89E-01 | 9.72E-01 | -0.035388 | -6.0734 | -0.03287 |
| hsa-miR-6499-3p | 9.89E-01 | 9.72E-01 | -0.035388 | -6.0734 | -0.03287 |
| hsa-miR-6499-5p | 9.89E-01 | 9.72E-01 | -0.035388 | -6.0734 | -0.03287 |
| hsa-miR-6500-3p | 9.89E-01 | 9.72E-01 | -0.035388 | -6.0734 | -0.03287 |
| hsa-miR-6501-3p | 9.89E-01 | 9.72E-01 | -0.035388 | -6.0734 | -0.03287 |
| hsa-miR-6501-5p | 9.89E-01 | 9.72E-01 | -0.035388 | -6.0734 | -0.03287 |
| hsa-miR-6502-3p | 9.89E-01 | 9.72E-01 | -0.035388 | -6.0734 | -0.03287 |
| hsa-miR-6502-5p | 9.89E-01 | 9.72E-01 | -0.035388 | -6.0734 | -0.03287 |
| hsa-miR-6503-3p | 9.89E-01 | 9.72E-01 | -0.035388 | -6.0734 | -0.03287 |
| hsa-miR-6503-5p | 9.89E-01 | 9.72E-01 | -0.035388 | -6.0734 | -0.03287 |
| hsa-miR-6504-3p | 9.89E-01 | 9.72E-01 | -0.035388 | -6.0734 | -0.03287 |
| hsa-miR-6504-5p | 9.89E-01 | 9.72E-01 | -0.035388 | -6.0734 | -0.03287 |
| hsa-miR-6505-3p | 9.89E-01 | 9.72E-01 | -0.035388 | -6.0734 | -0.03287 |
| hsa-miR-6505-5p | 9.89E-01 | 9.72E-01 | -0.035388 | -6.0734 | -0.03287 |
| hsa-miR-6506-3p | 9.89E-01 | 9.72E-01 | -0.035388 | -6.0734 | -0.03287 |
| hsa-miR-6506-5p | 9.89E-01 | 9.72E-01 | -0.035388 | -6.0734 | -0.03287 |
| hsa-miR-6507-3p | 9.89E-01 | 9.72E-01 | -0.035388 | -6.0734 | -0.03287 |
| hsa-miR-6507-5p | 9.89E-01 | 9.72E-01 | -0.035388 | -6.0734 | -0.03287 |
| hsa-miR-6508-3p | 9.89E-01 | 9.72E-01 | -0.035388 | -6.0734 | -0.03287 |
| hsa-miR-6509-3p | 9.89E-01 | 9.72E-01 | -0.035388 | -6.0734 | -0.03287 |
| hsa-miR-651-3p | 9.89E-01 | 9.72E-01 | -0.035388 | -6.0734 | -0.03287 |
| hsa-miR-651-5p | 9.89E-01 | 9.72E-01 | -0.035388 | -6.0734 | -0.03287 |
| hsa-miR-6510-3p | 9.89E-01 | 9.72E-01 | -0.035388 | -6.0734 | -0.03287 |
| hsa-miR-6511a-3p | 9.89E-01 | 9.72E-01 | -0.035388 | -6.0734 | -0.03287 |
| hsa-miR-6511b-3p | 9.89E-01 | 9.72E-01 | -0.035388 | -6.0734 | -0.03287 |
| hsa-miR-6512-3p | 9.89E-01 | 9.72E-01 | -0.035388 | -6.0734 | -0.03287 |
| hsa-miR-6513-3p | 9.89E-01 | 9.72E-01 | -0.035388 | -6.0734 | -0.03287 |
| hsa-miR-6513-5p | 9.89E-01 | 9.72E-01 | -0.035388 | -6.0734 | -0.03287 |
| hsa-miR-6514-3p | 9.89E-01 | 9.72E-01 | -0.035388 | -6.0734 | -0.03287 |
| hsa-miR-6514-5p | 9.89E-01 | 9.72E-01 | -0.035388 | -6.0734 | -0.03287 |
| hsa-miR-6515-5p | 9.89E-01 | 9.72E-01 | -0.035388 | -6.0734 | -0.03287 |
| hsa-miR-653-3p | 9.89E-01 | 9.72E-01 | -0.035388 | -6.0734 | -0.03287 |
| hsa-miR-653-5p | 9.89E-01 | 9.72E-01 | -0.035388 | -6.0734 | -0.03287 |
| hsa-miR-655-3p | 9.89E-01 | 9.72E-01 | -0.035388 | -6.0734 | -0.03287 |
| hsa-miR-655-5p | 9.89E-01 | 9.72E-01 | -0.035388 | -6.0734 | -0.03287 |
| hsa-miR-656-3p | 9.89E-01 | 9.72E-01 | -0.035388 | -6.0734 | -0.03287 |
| hsa-miR-656-5p | 9.89E-01 | 9.72E-01 | -0.035388 | -6.0734 | -0.03287 |
| hsa-miR-657 | 9.89E-01 | 9.72E-01 | -0.035388 | -6.0734 | -0.03287 |
| hsa-miR-658 | 9.89E-01 | 9.72E-01 | -0.035388 | -6.0734 | -0.03287 |
| hsa-miR-659-5p | 9.89E-01 | 9.72E-01 | -0.035388 | -6.0734 | -0.03287 |
| hsa-miR-661 | 9.89E-01 | 9.72E-01 | -0.035388 | -6.0734 | -0.03287 |
| hsa-miR-668-3p | 9.89E-01 | 9.72E-01 | -0.035388 | -6.0734 | -0.03287 |
| hsa-miR-668-5p | 9.89E-01 | 9.72E-01 | -0.035388 | -6.0734 | -0.03287 |
| hsa-miR-670-3p | 9.89E-01 | 9.72E-01 | -0.035388 | -6.0734 | -0.03287 |
| hsa-miR-670-5p | 9.89E-01 | 9.72E-01 | -0.035388 | -6.0734 | -0.03287 |
| hsa-miR-671-3p | 9.89E-01 | 9.72E-01 | -0.035388 | -6.0734 | -0.03287 |
| hsa-miR-6715a-3p | 9.89E-01 | 9.72E-01 | -0.035388 | -6.0734 | -0.03287 |
| hsa-miR-6715b-3p | 9.89E-01 | 9.72E-01 | -0.035388 | -6.0734 | -0.03287 |
| hsa-miR-6715b-5p | 9.89E-01 | 9.72E-01 | -0.035388 | -6.0734 | -0.03287 |
| hsa-miR-6716-3p | 9.89E-01 | 9.72E-01 | -0.035388 | -6.0734 | -0.03287 |
| hsa-miR-6716-5p | 9.89E-01 | 9.72E-01 | -0.035388 | -6.0734 | -0.03287 |
| hsa-miR-6718-5p | 9.89E-01 | 9.72E-01 | -0.035388 | -6.0734 | -0.03287 |
| hsa-miR-6719-3p | 9.89E-01 | 9.72E-01 | -0.035388 | -6.0734 | -0.03287 |
| hsa-miR-6720-3p | 9.89E-01 | 9.72E-01 | -0.035388 | -6.0734 | -0.03287 |
| hsa-miR-6720-5p | 9.89E-01 | 9.72E-01 | -0.035388 | -6.0734 | -0.03287 |
| hsa-miR-6721-5p | 9.89E-01 | 9.72E-01 | -0.035388 | -6.0734 | -0.03287 |
| hsa-miR-6722-5p | 9.89E-01 | 9.72E-01 | -0.035388 | -6.0734 | -0.03287 |
| hsa-miR-6726-3p | 9.89E-01 | 9.72E-01 | -0.035388 | -6.0734 | -0.03287 |
| hsa-miR-6726-5p | 9.89E-01 | 9.72E-01 | -0.035388 | -6.0734 | -0.03287 |
| hsa-miR-6727-3p | 9.89E-01 | 9.72E-01 | -0.035388 | -6.0734 | -0.03287 |
| hsa-miR-6728-3p | 9.89E-01 | 9.72E-01 | -0.035388 | -6.0734 | -0.03287 |
| hsa-miR-6729-3p | 9.89E-01 | 9.72E-01 | -0.035388 | -6.0734 | -0.03287 |
| hsa-miR-6730-3p | 9.89E-01 | 9.72E-01 | -0.035388 | -6.0734 | -0.03287 |
| hsa-miR-6731-3p | 9.89E-01 | 9.72E-01 | -0.035388 | -6.0734 | -0.03287 |
| hsa-miR-6731-5p | 9.89E-01 | 9.72E-01 | -0.035388 | -6.0734 | -0.03287 |
| hsa-miR-6732-3p | 9.89E-01 | 9.72E-01 | -0.035388 | -6.0734 | -0.03287 |
| hsa-miR-6733-3p | 9.89E-01 | 9.72E-01 | -0.035388 | -6.0734 | -0.03287 |
| hsa-miR-6733-5p | 9.89E-01 | 9.72E-01 | -0.035388 | -6.0734 | -0.03287 |
| hsa-miR-6734-3p | 9.89E-01 | 9.72E-01 | -0.035388 | -6.0734 | -0.03287 |
| hsa-miR-6735-3p | 9.89E-01 | 9.72E-01 | -0.035388 | -6.0734 | -0.03287 |
| hsa-miR-6735-5p | 9.89E-01 | 9.72E-01 | -0.035388 | -6.0734 | -0.03287 |
| hsa-miR-6736-3p | 9.89E-01 | 9.72E-01 | -0.035388 | -6.0734 | -0.03287 |
| hsa-miR-6737-5p | 9.89E-01 | 9.72E-01 | -0.035388 | -6.0734 | -0.03287 |
| hsa-miR-6738-3p | 9.89E-01 | 9.72E-01 | -0.035388 | -6.0734 | -0.03287 |
| hsa-miR-6739-3p | 9.89E-01 | 9.72E-01 | -0.035388 | -6.0734 | -0.03287 |
| hsa-miR-6739-5p | 9.89E-01 | 9.72E-01 | -0.035388 | -6.0734 | -0.03287 |
| hsa-miR-6740-3p | 9.89E-01 | 9.72E-01 | -0.035388 | -6.0734 | -0.03287 |
| hsa-miR-6741-3p | 9.89E-01 | 9.72E-01 | -0.035388 | -6.0734 | -0.03287 |
| hsa-miR-6742-3p | 9.89E-01 | 9.72E-01 | -0.035388 | -6.0734 | -0.03287 |
| hsa-miR-6742-5p | 9.89E-01 | 9.72E-01 | -0.035388 | -6.0734 | -0.03287 |
| hsa-miR-6743-3p | 9.89E-01 | 9.72E-01 | -0.035388 | -6.0734 | -0.03287 |
| hsa-miR-6743-5p | 9.89E-01 | 9.72E-01 | -0.035388 | -6.0734 | -0.03287 |
| hsa-miR-6744-3p | 9.89E-01 | 9.72E-01 | -0.035388 | -6.0734 | -0.03287 |
| hsa-miR-6744-5p | 9.89E-01 | 9.72E-01 | -0.035388 | -6.0734 | -0.03287 |
| hsa-miR-6746-3p | 9.89E-01 | 9.72E-01 | -0.035388 | -6.0734 | -0.03287 |
| hsa-miR-6747-3p | 9.89E-01 | 9.72E-01 | -0.035388 | -6.0734 | -0.03287 |
| hsa-miR-6748-3p | 9.89E-01 | 9.72E-01 | -0.035388 | -6.0734 | -0.03287 |
| hsa-miR-6749-3p | 9.89E-01 | 9.72E-01 | -0.035388 | -6.0734 | -0.03287 |
| hsa-miR-675-3p | 9.89E-01 | 9.72E-01 | -0.035388 | -6.0734 | -0.03287 |
| hsa-miR-675-5p | 9.89E-01 | 9.72E-01 | -0.035388 | -6.0734 | -0.03287 |
| hsa-miR-6750-3p | 9.89E-01 | 9.72E-01 | -0.035388 | -6.0734 | -0.03287 |
| hsa-miR-6750-5p | 9.89E-01 | 9.72E-01 | -0.035388 | -6.0734 | -0.03287 |
| hsa-miR-6751-3p | 9.89E-01 | 9.72E-01 | -0.035388 | -6.0734 | -0.03287 |
| hsa-miR-6751-5p | 9.89E-01 | 9.72E-01 | -0.035388 | -6.0734 | -0.03287 |
| hsa-miR-6754-3p | 9.89E-01 | 9.72E-01 | -0.035388 | -6.0734 | -0.03287 |
| hsa-miR-6754-5p | 9.89E-01 | 9.72E-01 | -0.035388 | -6.0734 | -0.03287 |
| hsa-miR-6755-3p | 9.89E-01 | 9.72E-01 | -0.035388 | -6.0734 | -0.03287 |
| hsa-miR-6755-5p | 9.89E-01 | 9.72E-01 | -0.035388 | -6.0734 | -0.03287 |
| hsa-miR-6756-3p | 9.89E-01 | 9.72E-01 | -0.035388 | -6.0734 | -0.03287 |
| hsa-miR-6757-3p | 9.89E-01 | 9.72E-01 | -0.035388 | -6.0734 | -0.03287 |
| hsa-miR-6758-3p | 9.89E-01 | 9.72E-01 | -0.035388 | -6.0734 | -0.03287 |
| hsa-miR-6759-3p | 9.89E-01 | 9.72E-01 | -0.035388 | -6.0734 | -0.03287 |
| hsa-miR-6759-5p | 9.89E-01 | 9.72E-01 | -0.035388 | -6.0734 | -0.03287 |
| hsa-miR-676-3p | 9.89E-01 | 9.72E-01 | -0.035388 | -6.0734 | -0.03287 |
| hsa-miR-676-5p | 9.89E-01 | 9.72E-01 | -0.035388 | -6.0734 | -0.03287 |
| hsa-miR-6760-3p | 9.89E-01 | 9.72E-01 | -0.035388 | -6.0734 | -0.03287 |
| hsa-miR-6761-3p | 9.89E-01 | 9.72E-01 | -0.035388 | -6.0734 | -0.03287 |
| hsa-miR-6761-5p | 9.89E-01 | 9.72E-01 | -0.035388 | -6.0734 | -0.03287 |
| hsa-miR-6762-3p | 9.89E-01 | 9.72E-01 | -0.035388 | -6.0734 | -0.03287 |
| hsa-miR-6762-5p | 9.89E-01 | 9.72E-01 | -0.035388 | -6.0734 | -0.03287 |
| hsa-miR-6764-3p | 9.89E-01 | 9.72E-01 | -0.035388 | -6.0734 | -0.03287 |
| hsa-miR-6764-5p | 9.89E-01 | 9.72E-01 | -0.035388 | -6.0734 | -0.03287 |
| hsa-miR-6766-5p | 9.89E-01 | 9.72E-01 | -0.035388 | -6.0734 | -0.03287 |
| hsa-miR-6767-3p | 9.89E-01 | 9.72E-01 | -0.035388 | -6.0734 | -0.03287 |
| hsa-miR-6768-3p | 9.89E-01 | 9.72E-01 | -0.035388 | -6.0734 | -0.03287 |
| hsa-miR-6769a-3p | 9.89E-01 | 9.72E-01 | -0.035388 | -6.0734 | -0.03287 |
| hsa-miR-6769b-3p | 9.89E-01 | 9.72E-01 | -0.035388 | -6.0734 | -0.03287 |
| hsa-miR-6770-3p | 9.89E-01 | 9.72E-01 | -0.035388 | -6.0734 | -0.03287 |
| hsa-miR-6770-5p | 9.89E-01 | 9.72E-01 | -0.035388 | -6.0734 | -0.03287 |
| hsa-miR-6771-3p | 9.89E-01 | 9.72E-01 | -0.035388 | -6.0734 | -0.03287 |
| hsa-miR-6771-5p | 9.89E-01 | 9.72E-01 | -0.035388 | -6.0734 | -0.03287 |
| hsa-miR-6772-3p | 9.89E-01 | 9.72E-01 | -0.035388 | -6.0734 | -0.03287 |
| hsa-miR-6773-3p | 9.89E-01 | 9.72E-01 | -0.035388 | -6.0734 | -0.03287 |
| hsa-miR-6773-5p | 9.89E-01 | 9.72E-01 | -0.035388 | -6.0734 | -0.03287 |
| hsa-miR-6774-3p | 9.89E-01 | 9.72E-01 | -0.035388 | -6.0734 | -0.03287 |
| hsa-miR-6775-3p | 9.89E-01 | 9.72E-01 | -0.035388 | -6.0734 | -0.03287 |
| hsa-miR-6776-3p | 9.89E-01 | 9.72E-01 | -0.035388 | -6.0734 | -0.03287 |
| hsa-miR-6778-3p | 9.89E-01 | 9.72E-01 | -0.035388 | -6.0734 | -0.03287 |
| hsa-miR-6779-3p | 9.89E-01 | 9.72E-01 | -0.035388 | -6.0734 | -0.03287 |
| hsa-miR-6780a-3p | 9.89E-01 | 9.72E-01 | -0.035388 | -6.0734 | -0.03287 |
| hsa-miR-6780b-3p | 9.89E-01 | 9.72E-01 | -0.035388 | -6.0734 | -0.03287 |
| hsa-miR-6781-3p | 9.89E-01 | 9.72E-01 | -0.035388 | -6.0734 | -0.03287 |
| hsa-miR-6782-3p | 9.89E-01 | 9.72E-01 | -0.035388 | -6.0734 | -0.03287 |
| hsa-miR-6782-5p | 9.89E-01 | 9.72E-01 | -0.035388 | -6.0734 | -0.03287 |
| hsa-miR-6783-3p | 9.89E-01 | 9.72E-01 | -0.035388 | -6.0734 | -0.03287 |
| hsa-miR-6783-5p | 9.89E-01 | 9.72E-01 | -0.035388 | -6.0734 | -0.03287 |
| hsa-miR-6784-3p | 9.89E-01 | 9.72E-01 | -0.035388 | -6.0734 | -0.03287 |
| hsa-miR-6785-3p | 9.89E-01 | 9.72E-01 | -0.035388 | -6.0734 | -0.03287 |
| hsa-miR-6786-3p | 9.89E-01 | 9.72E-01 | -0.035388 | -6.0734 | -0.03287 |
| hsa-miR-6787-3p | 9.89E-01 | 9.72E-01 | -0.035388 | -6.0734 | -0.03287 |
| hsa-miR-6788-3p | 9.89E-01 | 9.72E-01 | -0.035388 | -6.0734 | -0.03287 |
| hsa-miR-6789-3p | 9.89E-01 | 9.72E-01 | -0.035388 | -6.0734 | -0.03287 |
| hsa-miR-6790-3p | 9.89E-01 | 9.72E-01 | -0.035388 | -6.0734 | -0.03287 |
| hsa-miR-6791-3p | 9.89E-01 | 9.72E-01 | -0.035388 | -6.0734 | -0.03287 |
| hsa-miR-6792-3p | 9.89E-01 | 9.72E-01 | -0.035388 | -6.0734 | -0.03287 |
| hsa-miR-6793-3p | 9.89E-01 | 9.72E-01 | -0.035388 | -6.0734 | -0.03287 |
| hsa-miR-6794-3p | 9.89E-01 | 9.72E-01 | -0.035388 | -6.0734 | -0.03287 |
| hsa-miR-6795-3p | 9.89E-01 | 9.72E-01 | -0.035388 | -6.0734 | -0.03287 |
| hsa-miR-6796-3p | 9.89E-01 | 9.72E-01 | -0.035388 | -6.0734 | -0.03287 |
| hsa-miR-6799-3p | 9.89E-01 | 9.72E-01 | -0.035388 | -6.0734 | -0.03287 |
| hsa-miR-6801-3p | 9.89E-01 | 9.72E-01 | -0.035388 | -6.0734 | -0.03287 |
| hsa-miR-6802-3p | 9.89E-01 | 9.72E-01 | -0.035388 | -6.0734 | -0.03287 |
| hsa-miR-6803-3p | 9.89E-01 | 9.72E-01 | -0.035388 | -6.0734 | -0.03287 |
| hsa-miR-6804-3p | 9.89E-01 | 9.72E-01 | -0.035388 | -6.0734 | -0.03287 |
| hsa-miR-6805-5p | 9.89E-01 | 9.72E-01 | -0.035388 | -6.0734 | -0.03287 |
| hsa-miR-6806-3p | 9.89E-01 | 9.72E-01 | -0.035388 | -6.0734 | -0.03287 |
| hsa-miR-6807-3p | 9.89E-01 | 9.72E-01 | -0.035388 | -6.0734 | -0.03287 |
| hsa-miR-6808-3p | 9.89E-01 | 9.72E-01 | -0.035388 | -6.0734 | -0.03287 |
| hsa-miR-6809-3p | 9.89E-01 | 9.72E-01 | -0.035388 | -6.0734 | -0.03287 |
| hsa-miR-6810-3p | 9.89E-01 | 9.72E-01 | -0.035388 | -6.0734 | -0.03287 |
| hsa-miR-6810-5p | 9.89E-01 | 9.72E-01 | -0.035388 | -6.0734 | -0.03287 |
| hsa-miR-6811-3p | 9.89E-01 | 9.72E-01 | -0.035388 | -6.0734 | -0.03287 |
| hsa-miR-6811-5p | 9.89E-01 | 9.72E-01 | -0.035388 | -6.0734 | -0.03287 |
| hsa-miR-6812-3p | 9.89E-01 | 9.72E-01 | -0.035388 | -6.0734 | -0.03287 |
| hsa-miR-6813-5p | 9.89E-01 | 9.72E-01 | -0.035388 | -6.0734 | -0.03287 |
| hsa-miR-6814-3p | 9.89E-01 | 9.72E-01 | -0.035388 | -6.0734 | -0.03287 |
| hsa-miR-6814-5p | 9.89E-01 | 9.72E-01 | -0.035388 | -6.0734 | -0.03287 |
| hsa-miR-6815-3p | 9.89E-01 | 9.72E-01 | -0.035388 | -6.0734 | -0.03287 |
| hsa-miR-6816-3p | 9.89E-01 | 9.72E-01 | -0.035388 | -6.0734 | -0.03287 |
| hsa-miR-6816-5p | 9.89E-01 | 9.72E-01 | -0.035388 | -6.0734 | -0.03287 |
| hsa-miR-6817-3p | 9.89E-01 | 9.72E-01 | -0.035388 | -6.0734 | -0.03287 |
| hsa-miR-6817-5p | 9.89E-01 | 9.72E-01 | -0.035388 | -6.0734 | -0.03287 |
| hsa-miR-6818-3p | 9.89E-01 | 9.72E-01 | -0.035388 | -6.0734 | -0.03287 |
| hsa-miR-6818-5p | 9.89E-01 | 9.72E-01 | -0.035388 | -6.0734 | -0.03287 |
| hsa-miR-6820-3p | 9.89E-01 | 9.72E-01 | -0.035388 | -6.0734 | -0.03287 |
| hsa-miR-6821-3p | 9.89E-01 | 9.72E-01 | -0.035388 | -6.0734 | -0.03287 |
| hsa-miR-6822-3p | 9.89E-01 | 9.72E-01 | -0.035388 | -6.0734 | -0.03287 |
| hsa-miR-6822-5p | 9.89E-01 | 9.72E-01 | -0.035388 | -6.0734 | -0.03287 |
| hsa-miR-6823-3p | 9.89E-01 | 9.72E-01 | -0.035388 | -6.0734 | -0.03287 |
| hsa-miR-6823-5p | 9.89E-01 | 9.72E-01 | -0.035388 | -6.0734 | -0.03287 |
| hsa-miR-6824-3p | 9.89E-01 | 9.72E-01 | -0.035388 | -6.0734 | -0.03287 |
| hsa-miR-6825-3p | 9.89E-01 | 9.72E-01 | -0.035388 | -6.0734 | -0.03287 |
| hsa-miR-6826-3p | 9.89E-01 | 9.72E-01 | -0.035388 | -6.0734 | -0.03287 |
| hsa-miR-6827-3p | 9.89E-01 | 9.72E-01 | -0.035388 | -6.0734 | -0.03287 |
| hsa-miR-6827-5p | 9.89E-01 | 9.72E-01 | -0.035388 | -6.0734 | -0.03287 |
| hsa-miR-6828-3p | 9.89E-01 | 9.72E-01 | -0.035388 | -6.0734 | -0.03287 |
| hsa-miR-6828-5p | 9.89E-01 | 9.72E-01 | -0.035388 | -6.0734 | -0.03287 |
| hsa-miR-6829-3p | 9.89E-01 | 9.72E-01 | -0.035388 | -6.0734 | -0.03287 |
| hsa-miR-6830-3p | 9.89E-01 | 9.72E-01 | -0.035388 | -6.0734 | -0.03287 |
| hsa-miR-6831-3p | 9.89E-01 | 9.72E-01 | -0.035388 | -6.0734 | -0.03287 |
| hsa-miR-6832-3p | 9.89E-01 | 9.72E-01 | -0.035388 | -6.0734 | -0.03287 |
| hsa-miR-6833-3p | 9.89E-01 | 9.72E-01 | -0.035388 | -6.0734 | -0.03287 |
| hsa-miR-6834-3p | 9.89E-01 | 9.72E-01 | -0.035388 | -6.0734 | -0.03287 |
| hsa-miR-6834-5p | 9.89E-01 | 9.72E-01 | -0.035388 | -6.0734 | -0.03287 |
| hsa-miR-6835-3p | 9.89E-01 | 9.72E-01 | -0.035388 | -6.0734 | -0.03287 |
| hsa-miR-6835-5p | 9.89E-01 | 9.72E-01 | -0.035388 | -6.0734 | -0.03287 |
| hsa-miR-6836-3p | 9.89E-01 | 9.72E-01 | -0.035388 | -6.0734 | -0.03287 |
| hsa-miR-6836-5p | 9.89E-01 | 9.72E-01 | -0.035388 | -6.0734 | -0.03287 |
| hsa-miR-6837-3p | 9.89E-01 | 9.72E-01 | -0.035388 | -6.0734 | -0.03287 |
| hsa-miR-6838-3p | 9.89E-01 | 9.72E-01 | -0.035388 | -6.0734 | -0.03287 |
| hsa-miR-6838-5p | 9.89E-01 | 9.72E-01 | -0.035388 | -6.0734 | -0.03287 |
| hsa-miR-6839-3p | 9.89E-01 | 9.72E-01 | -0.035388 | -6.0734 | -0.03287 |
| hsa-miR-6839-5p | 9.89E-01 | 9.72E-01 | -0.035388 | -6.0734 | -0.03287 |
| hsa-miR-6840-5p | 9.89E-01 | 9.72E-01 | -0.035388 | -6.0734 | -0.03287 |
| hsa-miR-6841-3p | 9.89E-01 | 9.72E-01 | -0.035388 | -6.0734 | -0.03287 |
| hsa-miR-6841-5p | 9.89E-01 | 9.72E-01 | -0.035388 | -6.0734 | -0.03287 |
| hsa-miR-6842-3p | 9.89E-01 | 9.72E-01 | -0.035388 | -6.0734 | -0.03287 |
| hsa-miR-6842-5p | 9.89E-01 | 9.72E-01 | -0.035388 | -6.0734 | -0.03287 |
| hsa-miR-6843-3p | 9.89E-01 | 9.72E-01 | -0.035388 | -6.0734 | -0.03287 |
| hsa-miR-6844 | 9.89E-01 | 9.72E-01 | -0.035388 | -6.0734 | -0.03287 |
| hsa-miR-6845-3p | 9.89E-01 | 9.72E-01 | -0.035388 | -6.0734 | -0.03287 |
| hsa-miR-6846-3p | 9.89E-01 | 9.72E-01 | -0.035388 | -6.0734 | -0.03287 |
| hsa-miR-6847-3p | 9.89E-01 | 9.72E-01 | -0.035388 | -6.0734 | -0.03287 |
| hsa-miR-6849-3p | 9.89E-01 | 9.72E-01 | -0.035388 | -6.0734 | -0.03287 |
| hsa-miR-6850-3p | 9.89E-01 | 9.72E-01 | -0.035388 | -6.0734 | -0.03287 |
| hsa-miR-6851-3p | 9.89E-01 | 9.72E-01 | -0.035388 | -6.0734 | -0.03287 |
| hsa-miR-6852-3p | 9.89E-01 | 9.72E-01 | -0.035388 | -6.0734 | -0.03287 |
| hsa-miR-6852-5p | 9.89E-01 | 9.72E-01 | -0.035388 | -6.0734 | -0.03287 |
| hsa-miR-6853-3p | 9.89E-01 | 9.72E-01 | -0.035388 | -6.0734 | -0.03287 |
| hsa-miR-6853-5p | 9.89E-01 | 9.72E-01 | -0.035388 | -6.0734 | -0.03287 |
| hsa-miR-6854-3p | 9.89E-01 | 9.72E-01 | -0.035388 | -6.0734 | -0.03287 |
| hsa-miR-6854-5p | 9.89E-01 | 9.72E-01 | -0.035388 | -6.0734 | -0.03287 |
| hsa-miR-6855-3p | 9.89E-01 | 9.72E-01 | -0.035388 | -6.0734 | -0.03287 |
| hsa-miR-6855-5p | 9.89E-01 | 9.72E-01 | -0.035388 | -6.0734 | -0.03287 |
| hsa-miR-6856-3p | 9.89E-01 | 9.72E-01 | -0.035388 | -6.0734 | -0.03287 |
| hsa-miR-6857-3p | 9.89E-01 | 9.72E-01 | -0.035388 | -6.0734 | -0.03287 |
| hsa-miR-6858-3p | 9.89E-01 | 9.72E-01 | -0.035388 | -6.0734 | -0.03287 |
| hsa-miR-6859-3p | 9.89E-01 | 9.72E-01 | -0.035388 | -6.0734 | -0.03287 |
| hsa-miR-6859-5p | 9.89E-01 | 9.72E-01 | -0.035388 | -6.0734 | -0.03287 |
| hsa-miR-6861-3p | 9.89E-01 | 9.72E-01 | -0.035388 | -6.0734 | -0.03287 |
| hsa-miR-6862-3p | 9.89E-01 | 9.72E-01 | -0.035388 | -6.0734 | -0.03287 |
| hsa-miR-6863 | 9.89E-01 | 9.72E-01 | -0.035388 | -6.0734 | -0.03287 |
| hsa-miR-6864-3p | 9.89E-01 | 9.72E-01 | -0.035388 | -6.0734 | -0.03287 |
| hsa-miR-6864-5p | 9.89E-01 | 9.72E-01 | -0.035388 | -6.0734 | -0.03287 |
| hsa-miR-6865-3p | 9.89E-01 | 9.72E-01 | -0.035388 | -6.0734 | -0.03287 |
| hsa-miR-6866-3p | 9.89E-01 | 9.72E-01 | -0.035388 | -6.0734 | -0.03287 |
| hsa-miR-6866-5p | 9.89E-01 | 9.72E-01 | -0.035388 | -6.0734 | -0.03287 |
| hsa-miR-6867-3p | 9.89E-01 | 9.72E-01 | -0.035388 | -6.0734 | -0.03287 |
| hsa-miR-6868-3p | 9.89E-01 | 9.72E-01 | -0.035388 | -6.0734 | -0.03287 |
| hsa-miR-6868-5p | 9.89E-01 | 9.72E-01 | -0.035388 | -6.0734 | -0.03287 |
| hsa-miR-6869-3p | 9.89E-01 | 9.72E-01 | -0.035388 | -6.0734 | -0.03287 |
| hsa-miR-6870-3p | 9.89E-01 | 9.72E-01 | -0.035388 | -6.0734 | -0.03287 |
| hsa-miR-6871-3p | 9.89E-01 | 9.72E-01 | -0.035388 | -6.0734 | -0.03287 |
| hsa-miR-6873-3p | 9.89E-01 | 9.72E-01 | -0.035388 | -6.0734 | -0.03287 |
| hsa-miR-6873-5p | 9.89E-01 | 9.72E-01 | -0.035388 | -6.0734 | -0.03287 |
| hsa-miR-6874-3p | 9.89E-01 | 9.72E-01 | -0.035388 | -6.0734 | -0.03287 |
| hsa-miR-6874-5p | 9.89E-01 | 9.72E-01 | -0.035388 | -6.0734 | -0.03287 |
| hsa-miR-6875-3p | 9.89E-01 | 9.72E-01 | -0.035388 | -6.0734 | -0.03287 |
| hsa-miR-6876-3p | 9.89E-01 | 9.72E-01 | -0.035388 | -6.0734 | -0.03287 |
| hsa-miR-6878-3p | 9.89E-01 | 9.72E-01 | -0.035388 | -6.0734 | -0.03287 |
| hsa-miR-6878-5p | 9.89E-01 | 9.72E-01 | -0.035388 | -6.0734 | -0.03287 |
| hsa-miR-6879-3p | 9.89E-01 | 9.72E-01 | -0.035388 | -6.0734 | -0.03287 |
| hsa-miR-6881-3p | 9.89E-01 | 9.72E-01 | -0.035388 | -6.0734 | -0.03287 |
| hsa-miR-6882-3p | 9.89E-01 | 9.72E-01 | -0.035388 | -6.0734 | -0.03287 |
| hsa-miR-6883-3p | 9.89E-01 | 9.72E-01 | -0.035388 | -6.0734 | -0.03287 |
| hsa-miR-6883-5p | 9.89E-01 | 9.72E-01 | -0.035388 | -6.0734 | -0.03287 |
| hsa-miR-6884-3p | 9.89E-01 | 9.72E-01 | -0.035388 | -6.0734 | -0.03287 |
| hsa-miR-6884-5p | 9.89E-01 | 9.72E-01 | -0.035388 | -6.0734 | -0.03287 |
| hsa-miR-6885-3p | 9.89E-01 | 9.72E-01 | -0.035388 | -6.0734 | -0.03287 |
| hsa-miR-6885-5p | 9.89E-01 | 9.72E-01 | -0.035388 | -6.0734 | -0.03287 |
| hsa-miR-6886-3p | 9.89E-01 | 9.72E-01 | -0.035388 | -6.0734 | -0.03287 |
| hsa-miR-6887-3p | 9.89E-01 | 9.72E-01 | -0.035388 | -6.0734 | -0.03287 |
| hsa-miR-6888-3p | 9.89E-01 | 9.72E-01 | -0.035388 | -6.0734 | -0.03287 |
| hsa-miR-6888-5p | 9.89E-01 | 9.72E-01 | -0.035388 | -6.0734 | -0.03287 |
| hsa-miR-6890-3p | 9.89E-01 | 9.72E-01 | -0.035388 | -6.0734 | -0.03287 |
| hsa-miR-6891-3p | 9.89E-01 | 9.72E-01 | -0.035388 | -6.0734 | -0.03287 |
| hsa-miR-6892-3p | 9.89E-01 | 9.72E-01 | -0.035388 | -6.0734 | -0.03287 |
| hsa-miR-6892-5p | 9.89E-01 | 9.72E-01 | -0.035388 | -6.0734 | -0.03287 |
| hsa-miR-6893-3p | 9.89E-01 | 9.72E-01 | -0.035388 | -6.0734 | -0.03287 |
| hsa-miR-6894-3p | 9.89E-01 | 9.72E-01 | -0.035388 | -6.0734 | -0.03287 |
| hsa-miR-6895-3p | 9.89E-01 | 9.72E-01 | -0.035388 | -6.0734 | -0.03287 |
| hsa-miR-6895-5p | 9.89E-01 | 9.72E-01 | -0.035388 | -6.0734 | -0.03287 |
| hsa-miR-7-2-3p | 9.89E-01 | 9.72E-01 | -0.035388 | -6.0734 | -0.03287 |
| hsa-miR-708-3p | 9.89E-01 | 9.72E-01 | -0.035388 | -6.0734 | -0.03287 |
| hsa-miR-708-5p | 9.89E-01 | 9.72E-01 | -0.035388 | -6.0734 | -0.03287 |
| hsa-miR-7106-3p | 9.89E-01 | 9.72E-01 | -0.035388 | -6.0734 | -0.03287 |
| hsa-miR-7107-3p | 9.89E-01 | 9.72E-01 | -0.035388 | -6.0734 | -0.03287 |
| hsa-miR-7108-3p | 9.89E-01 | 9.72E-01 | -0.035388 | -6.0734 | -0.03287 |
| hsa-miR-7109-3p | 9.89E-01 | 9.72E-01 | -0.035388 | -6.0734 | -0.03287 |
| hsa-miR-7110-3p | 9.89E-01 | 9.72E-01 | -0.035388 | -6.0734 | -0.03287 |
| hsa-miR-7112-3p | 9.89E-01 | 9.72E-01 | -0.035388 | -6.0734 | -0.03287 |
| hsa-miR-7112-5p | 9.89E-01 | 9.72E-01 | -0.035388 | -6.0734 | -0.03287 |
| hsa-miR-7113-3p | 9.89E-01 | 9.72E-01 | -0.035388 | -6.0734 | -0.03287 |
| hsa-miR-7113-5p | 9.89E-01 | 9.72E-01 | -0.035388 | -6.0734 | -0.03287 |
| hsa-miR-7114-3p | 9.89E-01 | 9.72E-01 | -0.035388 | -6.0734 | -0.03287 |
| hsa-miR-7151-3p | 9.89E-01 | 9.72E-01 | -0.035388 | -6.0734 | -0.03287 |
| hsa-miR-7151-5p | 9.89E-01 | 9.72E-01 | -0.035388 | -6.0734 | -0.03287 |
| hsa-miR-7152-5p | 9.89E-01 | 9.72E-01 | -0.035388 | -6.0734 | -0.03287 |
| hsa-miR-7153-3p | 9.89E-01 | 9.72E-01 | -0.035388 | -6.0734 | -0.03287 |
| hsa-miR-7153-5p | 9.89E-01 | 9.72E-01 | -0.035388 | -6.0734 | -0.03287 |
| hsa-miR-7154-3p | 9.89E-01 | 9.72E-01 | -0.035388 | -6.0734 | -0.03287 |
| hsa-miR-7154-5p | 9.89E-01 | 9.72E-01 | -0.035388 | -6.0734 | -0.03287 |
| hsa-miR-7155-3p | 9.89E-01 | 9.72E-01 | -0.035388 | -6.0734 | -0.03287 |
| hsa-miR-7155-5p | 9.89E-01 | 9.72E-01 | -0.035388 | -6.0734 | -0.03287 |
| hsa-miR-7156-3p | 9.89E-01 | 9.72E-01 | -0.035388 | -6.0734 | -0.03287 |
| hsa-miR-7156-5p | 9.89E-01 | 9.72E-01 | -0.035388 | -6.0734 | -0.03287 |
| hsa-miR-7157-3p | 9.89E-01 | 9.72E-01 | -0.035388 | -6.0734 | -0.03287 |
| hsa-miR-7157-5p | 9.89E-01 | 9.72E-01 | -0.035388 | -6.0734 | -0.03287 |
| hsa-miR-7158-3p | 9.89E-01 | 9.72E-01 | -0.035388 | -6.0734 | -0.03287 |
| hsa-miR-7158-5p | 9.89E-01 | 9.72E-01 | -0.035388 | -6.0734 | -0.03287 |
| hsa-miR-7159-3p | 9.89E-01 | 9.72E-01 | -0.035388 | -6.0734 | -0.03287 |
| hsa-miR-7160-3p | 9.89E-01 | 9.72E-01 | -0.035388 | -6.0734 | -0.03287 |
| hsa-miR-7160-5p | 9.89E-01 | 9.72E-01 | -0.035388 | -6.0734 | -0.03287 |
| hsa-miR-7161-3p | 9.89E-01 | 9.72E-01 | -0.035388 | -6.0734 | -0.03287 |
| hsa-miR-7161-5p | 9.89E-01 | 9.72E-01 | -0.035388 | -6.0734 | -0.03287 |
| hsa-miR-7162-5p | 9.89E-01 | 9.72E-01 | -0.035388 | -6.0734 | -0.03287 |
| hsa-miR-744-3p | 9.89E-01 | 9.72E-01 | -0.035388 | -6.0734 | -0.03287 |
| hsa-miR-758-3p | 9.89E-01 | 9.72E-01 | -0.035388 | -6.0734 | -0.03287 |
| hsa-miR-759 | 9.89E-01 | 9.72E-01 | -0.035388 | -6.0734 | -0.03287 |
| hsa-miR-761 | 9.89E-01 | 9.72E-01 | -0.035388 | -6.0734 | -0.03287 |
| hsa-miR-764 | 9.89E-01 | 9.72E-01 | -0.035388 | -6.0734 | -0.03287 |
| hsa-miR-766-5p | 9.89E-01 | 9.72E-01 | -0.035388 | -6.0734 | -0.03287 |
| hsa-miR-767-3p | 9.89E-01 | 9.72E-01 | -0.035388 | -6.0734 | -0.03287 |
| hsa-miR-767-5p | 9.89E-01 | 9.72E-01 | -0.035388 | -6.0734 | -0.03287 |
| hsa-miR-770-5p | 9.89E-01 | 9.72E-01 | -0.035388 | -6.0734 | -0.03287 |
| hsa-miR-7702 | 9.89E-01 | 9.72E-01 | -0.035388 | -6.0734 | -0.03287 |
| hsa-miR-7703 | 9.89E-01 | 9.72E-01 | -0.035388 | -6.0734 | -0.03287 |
| hsa-miR-7705 | 9.89E-01 | 9.72E-01 | -0.035388 | -6.0734 | -0.03287 |
| hsa-miR-7706 | 9.89E-01 | 9.72E-01 | -0.035388 | -6.0734 | -0.03287 |
| hsa-miR-7843-3p | 9.89E-01 | 9.72E-01 | -0.035388 | -6.0734 | -0.03287 |
| hsa-miR-7843-5p | 9.89E-01 | 9.72E-01 | -0.035388 | -6.0734 | -0.03287 |
| hsa-miR-7844-5p | 9.89E-01 | 9.72E-01 | -0.035388 | -6.0734 | -0.03287 |
| hsa-miR-7848-3p | 9.89E-01 | 9.72E-01 | -0.035388 | -6.0734 | -0.03287 |
| hsa-miR-7849-3p | 9.89E-01 | 9.72E-01 | -0.035388 | -6.0734 | -0.03287 |
| hsa-miR-7850-5p | 9.89E-01 | 9.72E-01 | -0.035388 | -6.0734 | -0.03287 |
| hsa-miR-7852-3p | 9.89E-01 | 9.72E-01 | -0.035388 | -6.0734 | -0.03287 |
| hsa-miR-7853-5p | 9.89E-01 | 9.72E-01 | -0.035388 | -6.0734 | -0.03287 |
| hsa-miR-7855-5p | 9.89E-01 | 9.72E-01 | -0.035388 | -6.0734 | -0.03287 |
| hsa-miR-7856-5p | 9.89E-01 | 9.72E-01 | -0.035388 | -6.0734 | -0.03287 |
| hsa-miR-7973 | 9.89E-01 | 9.72E-01 | -0.035388 | -6.0734 | -0.03287 |
| hsa-miR-7974 | 9.89E-01 | 9.72E-01 | -0.035388 | -6.0734 | -0.03287 |
| hsa-miR-7976 | 9.89E-01 | 9.72E-01 | -0.035388 | -6.0734 | -0.03287 |
| hsa-miR-7978 | 9.89E-01 | 9.72E-01 | -0.035388 | -6.0734 | -0.03287 |
| hsa-miR-802 | 9.89E-01 | 9.72E-01 | -0.035388 | -6.0734 | -0.03287 |
| hsa-miR-8052 | 9.89E-01 | 9.72E-01 | -0.035388 | -6.0734 | -0.03287 |
| hsa-miR-8053 | 9.89E-01 | 9.72E-01 | -0.035388 | -6.0734 | -0.03287 |
| hsa-miR-8054 | 9.89E-01 | 9.72E-01 | -0.035388 | -6.0734 | -0.03287 |
| hsa-miR-8055 | 9.89E-01 | 9.72E-01 | -0.035388 | -6.0734 | -0.03287 |
| hsa-miR-8056 | 9.89E-01 | 9.72E-01 | -0.035388 | -6.0734 | -0.03287 |
| hsa-miR-8057 | 9.89E-01 | 9.72E-01 | -0.035388 | -6.0734 | -0.03287 |
| hsa-miR-8058 | 9.89E-01 | 9.72E-01 | -0.035388 | -6.0734 | -0.03287 |
| hsa-miR-8059 | 9.89E-01 | 9.72E-01 | -0.035388 | -6.0734 | -0.03287 |
| hsa-miR-8061 | 9.89E-01 | 9.72E-01 | -0.035388 | -6.0734 | -0.03287 |
| hsa-miR-8062 | 9.89E-01 | 9.72E-01 | -0.035388 | -6.0734 | -0.03287 |
| hsa-miR-8065 | 9.89E-01 | 9.72E-01 | -0.035388 | -6.0734 | -0.03287 |
| hsa-miR-8066 | 9.89E-01 | 9.72E-01 | -0.035388 | -6.0734 | -0.03287 |
| hsa-miR-8067 | 9.89E-01 | 9.72E-01 | -0.035388 | -6.0734 | -0.03287 |
| hsa-miR-8068 | 9.89E-01 | 9.72E-01 | -0.035388 | -6.0734 | -0.03287 |
| hsa-miR-8070 | 9.89E-01 | 9.72E-01 | -0.035388 | -6.0734 | -0.03287 |
| hsa-miR-8074 | 9.89E-01 | 9.72E-01 | -0.035388 | -6.0734 | -0.03287 |
| hsa-miR-8075 | 9.89E-01 | 9.72E-01 | -0.035388 | -6.0734 | -0.03287 |
| hsa-miR-8076 | 9.89E-01 | 9.72E-01 | -0.035388 | -6.0734 | -0.03287 |
| hsa-miR-8077 | 9.89E-01 | 9.72E-01 | -0.035388 | -6.0734 | -0.03287 |
| hsa-miR-8078 | 9.89E-01 | 9.72E-01 | -0.035388 | -6.0734 | -0.03287 |
| hsa-miR-8079 | 9.89E-01 | 9.72E-01 | -0.035388 | -6.0734 | -0.03287 |
| hsa-miR-8080 | 9.89E-01 | 9.72E-01 | -0.035388 | -6.0734 | -0.03287 |
| hsa-miR-8081 | 9.89E-01 | 9.72E-01 | -0.035388 | -6.0734 | -0.03287 |
| hsa-miR-8082 | 9.89E-01 | 9.72E-01 | -0.035388 | -6.0734 | -0.03287 |
| hsa-miR-8083 | 9.89E-01 | 9.72E-01 | -0.035388 | -6.0734 | -0.03287 |
| hsa-miR-8084 | 9.89E-01 | 9.72E-01 | -0.035388 | -6.0734 | -0.03287 |
| hsa-miR-8086 | 9.89E-01 | 9.72E-01 | -0.035388 | -6.0734 | -0.03287 |
| hsa-miR-8088 | 9.89E-01 | 9.72E-01 | -0.035388 | -6.0734 | -0.03287 |
| hsa-miR-873-3p | 9.89E-01 | 9.72E-01 | -0.035388 | -6.0734 | -0.03287 |
| hsa-miR-873-5p | 9.89E-01 | 9.72E-01 | -0.035388 | -6.0734 | -0.03287 |
| hsa-miR-874-5p | 9.89E-01 | 9.72E-01 | -0.035388 | -6.0734 | -0.03287 |
| hsa-miR-875-3p | 9.89E-01 | 9.72E-01 | -0.035388 | -6.0734 | -0.03287 |
| hsa-miR-875-5p | 9.89E-01 | 9.72E-01 | -0.035388 | -6.0734 | -0.03287 |
| hsa-miR-876-3p | 9.89E-01 | 9.72E-01 | -0.035388 | -6.0734 | -0.03287 |
| hsa-miR-876-5p | 9.89E-01 | 9.72E-01 | -0.035388 | -6.0734 | -0.03287 |
| hsa-miR-885-3p | 9.89E-01 | 9.72E-01 | -0.035388 | -6.0734 | -0.03287 |
| hsa-miR-887-5p | 9.89E-01 | 9.72E-01 | -0.035388 | -6.0734 | -0.03287 |
| hsa-miR-888-3p | 9.89E-01 | 9.72E-01 | -0.035388 | -6.0734 | -0.03287 |
| hsa-miR-888-5p | 9.89E-01 | 9.72E-01 | -0.035388 | -6.0734 | -0.03287 |
| hsa-miR-889-3p | 9.89E-01 | 9.72E-01 | -0.035388 | -6.0734 | -0.03287 |
| hsa-miR-889-5p | 9.89E-01 | 9.72E-01 | -0.035388 | -6.0734 | -0.03287 |
| hsa-miR-890 | 9.89E-01 | 9.72E-01 | -0.035388 | -6.0734 | -0.03287 |
| hsa-miR-891a-3p | 9.89E-01 | 9.72E-01 | -0.035388 | -6.0734 | -0.03287 |
| hsa-miR-891a-5p | 9.89E-01 | 9.72E-01 | -0.035388 | -6.0734 | -0.03287 |
| hsa-miR-891b | 9.89E-01 | 9.72E-01 | -0.035388 | -6.0734 | -0.03287 |
| hsa-miR-892a | 9.89E-01 | 9.72E-01 | -0.035388 | -6.0734 | -0.03287 |
| hsa-miR-892c-3p | 9.89E-01 | 9.72E-01 | -0.035388 | -6.0734 | -0.03287 |
| hsa-miR-892c-5p | 9.89E-01 | 9.72E-01 | -0.035388 | -6.0734 | -0.03287 |
| hsa-miR-9-3p | 9.89E-01 | 9.72E-01 | -0.035388 | -6.0734 | -0.03287 |
| hsa-miR-9-5p | 9.89E-01 | 9.72E-01 | -0.035388 | -6.0734 | -0.03287 |
| hsa-miR-920 | 9.89E-01 | 9.72E-01 | -0.035388 | -6.0734 | -0.03287 |
| hsa-miR-922 | 9.89E-01 | 9.72E-01 | -0.035388 | -6.0734 | -0.03287 |
| hsa-miR-924 | 9.89E-01 | 9.72E-01 | -0.035388 | -6.0734 | -0.03287 |
| hsa-miR-92a-1-5p | 9.89E-01 | 9.72E-01 | -0.035388 | -6.0734 | -0.03287 |
| hsa-miR-92a-2-5p | 9.89E-01 | 9.72E-01 | -0.035388 | -6.0734 | -0.03287 |
| hsa-miR-92b-3p | 9.89E-01 | 9.72E-01 | -0.035388 | -6.0734 | -0.03287 |
| hsa-miR-92b-5p | 9.89E-01 | 9.72E-01 | -0.035388 | -6.0734 | -0.03287 |
| hsa-miR-933 | 9.89E-01 | 9.72E-01 | -0.035388 | -6.0734 | -0.03287 |
| hsa-miR-935 | 9.89E-01 | 9.72E-01 | -0.035388 | -6.0734 | -0.03287 |
| hsa-miR-937-3p | 9.89E-01 | 9.72E-01 | -0.035388 | -6.0734 | -0.03287 |
| hsa-miR-938 | 9.89E-01 | 9.72E-01 | -0.035388 | -6.0734 | -0.03287 |
| hsa-miR-939-3p | 9.89E-01 | 9.72E-01 | -0.035388 | -6.0734 | -0.03287 |
| hsa-miR-941 | 9.89E-01 | 9.72E-01 | -0.035388 | -6.0734 | -0.03287 |
| hsa-miR-942-3p | 9.89E-01 | 9.72E-01 | -0.035388 | -6.0734 | -0.03287 |
| hsa-miR-942-5p | 9.89E-01 | 9.72E-01 | -0.035388 | -6.0734 | -0.03287 |
| hsa-miR-943 | 9.89E-01 | 9.72E-01 | -0.035388 | -6.0734 | -0.03287 |
| hsa-miR-944 | 9.89E-01 | 9.72E-01 | -0.035388 | -6.0734 | -0.03287 |
| hsa-miR-95-5p | 9.89E-01 | 9.72E-01 | -0.035388 | -6.0734 | -0.03287 |
| hsa-miR-96-3p | 9.89E-01 | 9.72E-01 | -0.035388 | -6.0734 | -0.03287 |
| hsa-miR-98-3p | 9.89E-01 | 9.72E-01 | -0.035388 | -6.0734 | -0.03287 |
| hsa-miR-99a-3p | 9.89E-01 | 9.72E-01 | -0.035388 | -6.0734 | -0.03287 |
| hsa-miR-99b-3p | 9.89E-01 | 9.72E-01 | -0.035388 | -6.0734 | -0.03287 |
| hsa-miR-424-5p | 9.89E-01 | 9.73E-01 | -0.034439 | -6.0734 | -0.031989 |
| hsa-miR-1273c | 9.90E-01 | 9.73E-01 | -0.033499 | -6.0734 | -0.031116 |
| hsa-miR-6738-5p | 9.90E-01 | 9.74E-01 | 0.032515 | -6.0735 | 0.030202 |
| hsa-miR-3926 | 9.90E-01 | 9.74E-01 | 0.032154 | -6.0735 | 0.029867 |
| hsa-miR-4656 | 9.90E-01 | 9.75E-01 | 0.031107 | -6.0735 | 0.028894 |
| hsa-miR-4738-3p | 9.90E-01 | 9.75E-01 | 0.03087 | -6.0735 | 0.028674 |
| hsa-miR-7111-5p | 9.90E-01 | 9.75E-01 | -0.030788 | -6.0735 | -0.028598 |
| hsa-miR-4499 | 9.90E-01 | 9.76E-01 | 0.030163 | -6.0735 | 0.028017 |
| hsa-miR-4745-5p | 9.90E-01 | 9.76E-01 | 0.029674 | -6.0735 | 0.027563 |
| hsa-miR-4476 | 9.91E-01 | 9.78E-01 | 0.02809 | -6.0736 | 0.026092 |
| hsa-miR-6780b-5p | 9.91E-01 | 9.78E-01 | 0.027053 | -6.0736 | 0.025128 |
| hsa-miR-6871-5p | 9.91E-01 | 9.78E-01 | -0.026977 | -6.0736 | -0.025058 |
| hsa-miR-564 | 9.91E-01 | 9.80E-01 | -0.025183 | -6.0737 | -0.023391 |
| hsa-miR-6806-5p | 9.91E-01 | 9.80E-01 | 0.024492 | -6.0737 | 0.022749 |
| hsa-miR-1973 | 9.91E-01 | 9.80E-01 | 0.024451 | -6.0737 | 0.022711 |
| hsa-let-7d-5p | 9.91E-01 | 9.81E-01 | -0.023933 | -6.0737 | -0.022231 |
| hsa-miR-6870-5p | 9.91E-01 | 9.81E-01 | -0.023924 | -6.0737 | -0.022222 |
| hsa-miR-505-5p | 9.91E-01 | 9.81E-01 | 0.023503 | -6.0737 | 0.021831 |
| hsa-miR-423-5p | 9.91E-01 | 9.82E-01 | 0.022843 | -6.0737 | 0.021218 |
| hsa-miR-3141 | 9.91E-01 | 9.82E-01 | 0.022665 | -6.0737 | 0.021052 |
| hsa-miR-6068 | 9.91E-01 | 9.82E-01 | 0.022636 | -6.0737 | 0.021025 |
| hsa-miR-6876-5p | 9.91E-01 | 9.83E-01 | -0.021573 | -6.0737 | -0.020038 |
| hsa-miR-4665-3p | 9.92E-01 | 9.84E-01 | -0.020029 | -6.0738 | -0.018604 |
| hsa-miR-1273g-5p | 9.92E-01 | 9.84E-01 | -0.019714 | -6.0738 | -0.018311 |
| hsa-miR-493-3p | 9.92E-01 | 9.85E-01 | 0.019183 | -6.0738 | 0.017818 |
| hsa-miR-6756-5p | 9.92E-01 | 9.85E-01 | 0.01883 | -6.0738 | 0.01749 |
| hsa-miR-4767 | 9.92E-01 | 9.85E-01 | 0.018405 | -6.0738 | 0.017095 |
| hsa-miR-4485-3p | 9.92E-01 | 9.86E-01 | -0.0173 | -6.0738 | -0.01607 |
| hsa-miR-6858-5p | 9.93E-01 | 9.87E-01 | 0.016401 | -6.0738 | 0.015234 |
| hsa-miR-5190 | 9.97E-01 | 9.91E-01 | -0.01067 | -6.0739 | -0.009911 |
| hsa-miR-4428 | 9.97E-01 | 9.92E-01 | -0.00974 | -6.0739 | -0.009048 |
| hsa-miR-1228-5p | 9.98E-01 | 9.93E-01 | -0.008271 | -6.0739 | -0.007682 |
| hsa-miR-3911 | 9.98E-01 | 9.94E-01 | -0.007818 | -6.0739 | -0.007261 |
| hsa-miR-4695-5p | 9.98E-01 | 9.94E-01 | -0.007574 | -6.0739 | -0.007035 |
| hsa-miR-4487 | 9.98E-01 | 9.95E-01 | 0.006817 | -6.0739 | 0.006332 |
| hsa-miR-4644 | 9.98E-01 | 9.95E-01 | 0.006171 | -6.0739 | 0.005732 |
| hsa-miR-4698 | 9.98E-01 | 9.96E-01 | 0.005313 | -6.0739 | 0.004935 |
| hsa-miR-4314 | 9.98E-01 | 9.96E-01 | 0.005081 | -6.0739 | 0.00472 |
| hsa-miR-1236-5p | 9.98E-01 | 9.96E-01 | 0.004835 | -6.0739 | 0.004491 |
| hsa-miR-1273g-3p | 9.98E-01 | 9.96E-01 | -0.004548 | -6.0739 | -0.004224 |
| hsa-miR-631 | 9.98E-01 | 9.96E-01 | 0.004456 | -6.0739 | 0.004139 |
| hsa-miR-6837-5p | 9.98E-01 | 9.97E-01 | 0.003334 | -6.074 | 0.003097 |
| hsa-miR-2276-3p | 9.98E-01 | 9.98E-01 | 0.003114 | -6.074 | 0.002893 |
| hsa-miR-622 | 9.99E-01 | 9.99E-01 | 0.000838 | -6.074 | 0.000779 |

**Table S3** Target genes of DEMs predicted by miRNet.

| ID | Gene |
| --- | --- |
| hsa-mir-95-3p | CCND1 |
| hsa-mir-95-3p | CANX |
| hsa-mir-95-3p | CDKN1A |
| hsa-mir-95-3p | CEBPD |
| hsa-mir-95-3p | DUSP8 |
| hsa-mir-95-3p | SNX1 |
| hsa-mir-95-3p | NSD2 |
| hsa-mir-95-3p | DGS2 |
| hsa-mir-95-3p | CELF2 |
| hsa-mir-95-3p | NXPH3 |
| hsa-mir-95-3p | TMEM245 |
| hsa-mir-95-3p | TMEM33 |
| hsa-mir-95-3p | MRM3 |
| hsa-mir-552-3p | ADORA2B |
| hsa-mir-552-3p | AK2 |
| hsa-mir-552-3p | ALDOA |
| hsa-mir-552-3p | BIRC5 |
| hsa-mir-552-3p | AR |
| hsa-mir-552-3p | C3 |
| hsa-mir-552-3p | CAD |
| hsa-mir-552-3p | CAV2 |
| hsa-mir-552-3p | RUNX1T1 |
| hsa-mir-552-3p | CD81 |
| hsa-mir-552-3p | CEACAM5 |
| hsa-mir-552-3p | COX6B1 |
| hsa-mir-552-3p | CYB5A |
| hsa-mir-552-3p | ELK1 |
| hsa-mir-552-3p | ERBB2 |
| hsa-mir-552-3p | ESRRB |
| hsa-mir-552-3p | FARSA |
| hsa-mir-552-3p | FHL2 |
| hsa-mir-552-3p | FOXC1 |
| hsa-mir-552-3p | GABRB3 |
| hsa-mir-552-3p | NPBWR1 |
| hsa-mir-552-3p | HSPA4 |
| hsa-mir-552-3p | IPP |
| hsa-mir-552-3p | KCNMB1 |
| hsa-mir-552-3p | LFNG |
| hsa-mir-552-3p | MTAP |
| hsa-mir-552-3p | MYO1F |
| hsa-mir-552-3p | PFKFB2 |
| hsa-mir-552-3p | PIK3R2 |
| hsa-mir-552-3p | PITPNA |
| hsa-mir-552-3p | PKM |
| hsa-mir-552-3p | PKNOX1 |
| hsa-mir-552-3p | PLAGL2 |
| hsa-mir-552-3p | PRKX |
| hsa-mir-552-3p | SGK1 |
| hsa-mir-552-3p | SKI |
| hsa-mir-552-3p | SLC1A5 |
| hsa-mir-552-3p | SLC6A4 |
| hsa-mir-552-3p | SOD2 |
| hsa-mir-552-3p | SURF6 |
| hsa-mir-552-3p | ELOC |
| hsa-mir-552-3p | SUMO1 |
| hsa-mir-552-3p | EIF4H |
| hsa-mir-552-3p | BTG2 |
| hsa-mir-552-3p | REEP5 |
| hsa-mir-552-3p | AP3B2 |
| hsa-mir-552-3p | HIST2H2AA3 |
| hsa-mir-552-3p | TTF2 |
| hsa-mir-552-3p | IFITM1 |
| hsa-mir-552-3p | DGKE |
| hsa-mir-552-3p | TRIM24 |
| hsa-mir-552-3p | RABEP1 |
| hsa-mir-552-3p | SYNGR2 |
| hsa-mir-552-3p | RPL23 |
| hsa-mir-552-3p | PLAA |
| hsa-mir-552-3p | FXR2 |
| hsa-mir-552-3p | MTFR1 |
| hsa-mir-552-3p | SOCS5 |
| hsa-mir-552-3p | PCLAF |
| hsa-mir-552-3p | TSC22D2 |
| hsa-mir-552-3p | NUP153 |
| hsa-mir-552-3p | PAK4 |
| hsa-mir-552-3p | TUBA1B |
| hsa-mir-552-3p | IFITM3 |
| hsa-mir-552-3p | N4BP2L2 |
| hsa-mir-552-3p | CRTAP |
| hsa-mir-552-3p | ARFGEF2 |
| hsa-mir-552-3p | NFAT5 |
| hsa-mir-552-3p | SUGT1 |
| hsa-mir-552-3p | SUB1 |
| hsa-mir-552-3p | RAB32 |
| hsa-mir-552-3p | SLC27A4 |
| hsa-mir-552-3p | KIF3A |
| hsa-mir-552-3p | HSPA4L |
| hsa-mir-552-3p | MAST3 |
| hsa-mir-552-3p | PLXND1 |
| hsa-mir-552-3p | TTLL12 |
| hsa-mir-552-3p | DIP2A |
| hsa-mir-552-3p | NPTXR |
| hsa-mir-552-3p | RPL13A |
| hsa-mir-552-3p | ACOT9 |
| hsa-mir-552-3p | RTL8A |
| hsa-mir-552-3p | ABHD12 |
| hsa-mir-552-3p | CACYBP |
| hsa-mir-552-3p | TMOD3 |
| hsa-mir-552-3p | IRAK4 |
| hsa-mir-552-3p | MRNIP |
| hsa-mir-552-3p | DDX47 |
| hsa-mir-552-3p | SLC25A37 |
| hsa-mir-552-3p | ZNF44 |
| hsa-mir-552-3p | DNAJC10 |
| hsa-mir-552-3p | GPN2 |
| hsa-mir-552-3p | RBM28 |
| hsa-mir-552-3p | DARS2 |
| hsa-mir-552-3p | CCDC198 |
| hsa-mir-552-3p | KIAA1551 |
| hsa-mir-552-3p | AGPAT5 |
| hsa-mir-552-3p | SYNJ2BP |
| hsa-mir-552-3p | PIGV |
| hsa-mir-552-3p | PNPLA2 |
| hsa-mir-552-3p | MAN1C1 |
| hsa-mir-552-3p | STRIP2 |
| hsa-mir-552-3p | PNMA8B |
| hsa-mir-552-3p | NUFIP2 |
| hsa-mir-552-3p | TXNDC16 |
| hsa-mir-552-3p | TAOK1 |
| hsa-mir-552-3p | PHF12 |
| hsa-mir-552-3p | GBA2 |
| hsa-mir-552-3p | MANBAL |
| hsa-mir-552-3p | NECAB3 |
| hsa-mir-552-3p | ELOVL1 |
| hsa-mir-552-3p | SOWAHC |
| hsa-mir-552-3p | MPPE1 |
| hsa-mir-552-3p | WDR77 |
| hsa-mir-552-3p | PAGR1 |
| hsa-mir-552-3p | C12orf49 |
| hsa-mir-552-3p | DSN1 |
| hsa-mir-552-3p | DNAJB14 |
| hsa-mir-552-3p | FOXRED2 |
| hsa-mir-552-3p | GPR157 |
| hsa-mir-552-3p | ABHD18 |
| hsa-mir-552-3p | SLC44A4 |
| hsa-mir-552-3p | ISG20L2 |
| hsa-mir-552-3p | RAB1B |
| hsa-mir-552-3p | ABRAXAS1 |
| hsa-mir-552-3p | ARID5B |
| hsa-mir-552-3p | MRI1 |
| hsa-mir-552-3p | FAM213A |
| hsa-mir-552-3p | DCTN5 |
| hsa-mir-552-3p | ST6GAL2 |
| hsa-mir-552-3p | PIGO |
| hsa-mir-552-3p | ADO |
| hsa-mir-552-3p | ARHGEF39 |
| hsa-mir-552-3p | SHANK3 |
| hsa-mir-552-3p | STPG1 |
| hsa-mir-552-3p | BTF3L4 |
| hsa-mir-552-3p | NLRP12 |
| hsa-mir-552-3p | MTDH |
| hsa-mir-552-3p | ASB16 |
| hsa-mir-552-3p | RDH13 |
| hsa-mir-552-3p | ZNF354B |
| hsa-mir-552-3p | GJD3 |
| hsa-mir-552-3p | EDARADD |
| hsa-mir-552-3p | NACC2 |
| hsa-mir-552-3p | TMEM37 |
| hsa-mir-552-3p | ZNF785 |
| hsa-mir-552-3p | DIRAS1 |
| hsa-mir-552-3p | RNF187 |
| hsa-mir-552-3p | NIPAL1 |
| hsa-mir-552-3p | VKORC1L1 |
| hsa-mir-552-3p | ZNF791 |
| hsa-mir-552-3p | TTLL9 |
| hsa-mir-552-3p | ZNF48 |
| hsa-mir-552-3p | GPR137C |
| hsa-mir-552-3p | ARL10 |
| hsa-mir-552-3p | NSMCE2 |
| hsa-mir-552-3p | GPIHBP1 |
| hsa-mir-552-3p | WDR97 |
| hsa-mir-552-3p | ZNF677 |
| hsa-mir-552-3p | LCE1A |
| hsa-mir-552-3p | NHLRC3 |
| hsa-mir-552-3p | PRR5-ARHGAP8 |
| hsa-mir-552-3p | LINC00598 |
| hsa-mir-552-3p | ANKRD33B |
| hsa-mir-552-3p | HIST2H2AA4 |
| hsa-mir-552-3p | SHISA9 |
| hsa-mir-552-3p | TGFBR3L |
| hsa-mir-95-3p | CCND1 |
| hsa-mir-95-3p | CANX |
| hsa-mir-95-3p | SNX1 |
| hsa-mir-552-3p | CD81 |
| hsa-mir-552-3p | ERBB2 |
| hsa-mir-552-3p | PITPNA |
| hsa-mir-552-3p | PKM |
| hsa-mir-552-3p | PIGO |
| hsa-mir-552-3p | BTF3L4 |
| hsa-mir-95-3p | KRIT1 |
| hsa-mir-95-3p | KRIT1 |
| hsa-mir-552-3p | LASP1 |
| hsa-mir-552-3p | PHTF2 |
| hsa-mir-95-3p | CSDE1 |
| hsa-mir-95-3p | BTBD7 |
| hsa-mir-95-3p | BTBD7 |
| hsa-mir-552-3p | RNF216 |
| hsa-mir-95-3p | MBTPS2 |
| hsa-mir-552-3p | HEBP1 |
| hsa-mir-552-3p | STRAP |
| hsa-mir-95-3p | TOMM34 |
| hsa-mir-95-3p | VIM |
| hsa-mir-552-3p | BCLAF1 |
| hsa-mir-95-3p | ASTE1 |
| hsa-mir-552-3p | TIMP2 |
| hsa-mir-95-3p | TRIO |
| hsa-mir-552-3p | VCAN |
| hsa-mir-95-3p | RAI14 |
| hsa-mir-95-3p | FNIP2 |
| hsa-mir-552-3p | TAB2 |
| hsa-mir-552-3p | TAB2 |
| hsa-mir-552-3p | WNK1 |
| hsa-mir-552-3p | GNAI3 |
| hsa-mir-552-3p | ASB1 |
| hsa-mir-552-3p | YBX1 |
| hsa-mir-95-3p | TTC7A |
| hsa-mir-95-3p | ATP2B1 |
| hsa-mir-95-3p | KDM5A |
| hsa-mir-552-3p | ENO1 |
| hsa-mir-552-3p | MOCOS |
| hsa-mir-95-3p | SEC31B |
| hsa-mir-552-3p | SMAP2 |
| hsa-mir-95-3p | COL16A1 |
| hsa-mir-95-3p | SLC25A24 |
| hsa-mir-95-3p | SLC25A24 |
| hsa-mir-95-3p | DDHD2 |
| hsa-mir-552-3p | TTC39A |
| hsa-mir-95-3p | ADAMTS2 |
| hsa-mir-95-3p | ADAMTS2 |
| hsa-mir-552-3p | SUPT16H |
| hsa-mir-552-3p | MKNK2 |
| hsa-mir-552-3p | MAPK1 |
| hsa-mir-95-3p | MYH9 |
| hsa-mir-95-3p | NIN |
| hsa-mir-552-3p | DDHD1 |
| hsa-mir-95-3p | DDHD1 |
| hsa-mir-552-3p | SIX4 |
| hsa-mir-95-3p | GSKIP |
| hsa-mir-95-3p | ARHGAP5 |
| hsa-mir-552-3p | ADNP |
| hsa-mir-552-3p | PLS3 |
| hsa-mir-95-3p | RBBP7 |
| hsa-mir-552-3p | FLT1 |
| hsa-mir-95-3p | CORO1A |
| hsa-mir-552-3p | CTCF |
| hsa-mir-95-3p | CTCF |
| hsa-mir-95-3p | CAPN15 |
| hsa-mir-95-3p | CAPN15 |
| hsa-mir-95-3p | BLOC1S6 |
| hsa-mir-95-3p | MCM4 |
| hsa-mir-95-3p | WDR83OS |
| hsa-mir-552-3p | USP42 |
| hsa-mir-552-3p | CXCL12 |
| hsa-mir-95-3p | EIF3A |
| hsa-mir-95-3p | DKK1 |
| hsa-mir-95-3p | RASD1 |
| hsa-mir-95-3p | SLC16A6 |
| hsa-mir-95-3p | MMD |
| hsa-mir-95-3p | NMU |
| hsa-mir-95-3p | NUP98 |
| hsa-mir-95-3p | HPS5 |
| hsa-mir-95-3p | HPS5 |
| hsa-mir-552-3p | ELK3 |
| hsa-mir-95-3p | C2CD5 |
| hsa-mir-95-3p | BAG2 |
| hsa-mir-95-3p | QKI |
| hsa-mir-552-3p | NNT |
| hsa-mir-552-3p | ARRDC3 |
| hsa-mir-552-3p | GNPDA1 |
| hsa-mir-552-3p | NUP155 |
| hsa-mir-552-3p | GNAI2 |
| hsa-mir-95-3p | COMMD2 |
| hsa-mir-552-3p | COX7A2L |
| hsa-mir-95-3p | ASH1L |
| hsa-mir-552-3p | SMG7 |
| hsa-mir-95-3p | OSCP1 |
| hsa-mir-552-3p | MTR |
| hsa-mir-552-3p | ATP6V0B |
| hsa-mir-552-3p | IRF6 |
| hsa-mir-95-3p | OSBPL9 |
| hsa-mir-95-3p | KIF14 |
| hsa-mir-95-3p | CREB1 |
| hsa-mir-95-3p | ELOVL4 |
| hsa-mir-95-3p | RPN2 |
| hsa-mir-552-3p | PTBP3 |
| hsa-mir-552-3p | SMNDC1 |
| hsa-mir-95-3p | ARL1 |
| hsa-mir-552-3p | CPXM2 |
| hsa-mir-95-3p | ZNF205 |
| hsa-mir-552-3p | ZNF644 |
| hsa-mir-95-3p | ZNF644 |
| hsa-mir-552-3p | AGO2 |
| hsa-mir-552-3p | ZNFX1 |
| hsa-mir-95-3p | THOC2 |
| hsa-mir-95-3p | FOSB |
| hsa-mir-95-3p | UXT |
| hsa-mir-552-3p | RAB3IP |
| hsa-mir-95-3p | MPST |
| hsa-mir-95-3p | CGNL1 |
| hsa-mir-552-3p | DUT |
| hsa-mir-95-3p | ARPP19 |
| hsa-mir-95-3p | RPS4Y1 |
| hsa-mir-95-3p | CLIP1 |
| hsa-mir-95-3p | ZNF317 |
| hsa-mir-552-3p | ZNF426 |
| hsa-mir-95-3p | POLN |
| hsa-mir-95-3p | EMC8 |
| hsa-mir-552-3p | CAP1 |
| hsa-mir-552-3p | RLIM |
| hsa-mir-552-3p | TRAF7 |
| hsa-mir-552-3p | LRRC41 |
| hsa-mir-95-3p | GRSF1 |
| hsa-mir-552-3p | XPO4 |
| hsa-mir-552-3p | TPT1 |
| hsa-mir-95-3p | GPALPP1 |
| hsa-mir-552-3p | BEX1 |
| hsa-mir-95-3p | AGO4 |
| hsa-mir-552-3p | STT3A |
| hsa-mir-552-3p | GOLM1 |
| hsa-mir-95-3p | TMEM243 |
| hsa-mir-552-3p | HNRNPA1 |
| hsa-mir-552-3p | DHX9 |
| hsa-mir-95-3p | ITM2B |
| hsa-mir-552-3p | IREB2 |
| hsa-mir-552-3p | SAP130 |
| hsa-mir-95-3p | FGFBP1 |
| hsa-mir-95-3p | CENPE |
| hsa-mir-95-3p | NDUFA9 |
| hsa-mir-95-3p | PHLDA1 |
| hsa-mir-95-3p | VPS37B |
| hsa-mir-95-3p | HMG20A |
| hsa-mir-95-3p | PARN |
| hsa-mir-95-3p | PARN |
| hsa-mir-552-3p | BCAS3 |
| hsa-mir-552-3p | NPC1 |
| hsa-mir-95-3p | CBX4 |
| hsa-mir-552-3p | COL6A1 |
| hsa-mir-95-3p | ZNF614 |
| hsa-mir-95-3p | PRCC |
| hsa-mir-95-3p | HDGF |
| hsa-mir-95-3p | ABL2 |
| hsa-mir-552-3p | MCL1 |
| hsa-mir-95-3p | SUMF1 |
| hsa-mir-95-3p | BOC |
| hsa-mir-552-3p | BDP1 |
| hsa-mir-552-3p | BDP1 |
| hsa-mir-552-3p | BDP1 |
| hsa-mir-95-3p | SNX12 |
| hsa-mir-95-3p | IMMP1L |
| hsa-mir-552-3p | ZC3H12C |
| hsa-mir-95-3p | YTHDF1 |
| hsa-mir-552-3p | DIP2C |
| hsa-mir-552-3p | EPS8 |
| hsa-mir-552-3p | SACS |
| hsa-mir-95-3p | RABGAP1L |
| hsa-mir-95-3p | DSPP |
| hsa-mir-95-3p | CLEC4F |
| hsa-mir-95-3p | RANBP2 |
| hsa-mir-95-3p | GTF2E1 |
| hsa-mir-552-3p | TRIP12 |
| hsa-mir-95-3p | ADAMTS1 |
| hsa-mir-95-3p | SCAF4 |
| hsa-mir-552-3p | HK1 |
| hsa-mir-552-3p | NPTN |
| hsa-mir-95-3p | NPTN |
| hsa-mir-95-3p | UTP14A |
| hsa-mir-95-3p | SKI |
| hsa-mir-552-3p | DUSP2 |
| hsa-mir-95-3p | ELK4 |
| hsa-mir-552-3p | SSU72 |
| hsa-mir-95-3p | ADAR |
| hsa-mir-95-3p | LARP4 |
| hsa-mir-552-3p | DDR2 |
| hsa-mir-552-3p | CNST |
| hsa-mir-95-3p | SLC16A14 |
| hsa-mir-552-3p | FZD5 |
| hsa-mir-95-3p | PPP4R2 |
| hsa-mir-95-3p | DNAJC5G |
| hsa-mir-95-3p | UCN |
| hsa-mir-95-3p | PSIP1 |
| hsa-mir-552-3p | ARHGAP12 |
| hsa-mir-95-3p | ISCA2 |
| hsa-mir-95-3p | SMCO4 |
| hsa-mir-95-3p | B4GALNT2 |
| hsa-mir-95-3p | TRUB2 |
| hsa-mir-95-3p | DUS2 |
| hsa-mir-95-3p | KMT2D |
| hsa-mir-95-3p | TMEM68 |
| hsa-mir-95-3p | ECI1 |
| hsa-mir-552-3p | SRRM2 |
| hsa-mir-95-3p | SRRM2 |
| hsa-mir-95-3p | SETD5 |
| hsa-mir-552-3p | SEMA4C |
| hsa-mir-95-3p | SNTB2 |
| hsa-mir-95-3p | SNTB2 |
| hsa-mir-95-3p | MMGT1 |
| hsa-mir-95-3p | CLIC3 |
| hsa-mir-95-3p | HNRNPF |
| hsa-mir-552-3p | TRAPPC1 |
| hsa-mir-95-3p | SERPINA9 |
| hsa-mir-552-3p | FOS |
| hsa-mir-552-3p | SERPINB9 |
| hsa-mir-95-3p | CDCA4 |
| hsa-mir-95-3p | NPTX1 |
| hsa-mir-95-3p | ZNF581 |
| hsa-mir-552-3p | JMJD1C |
| hsa-mir-95-3p | ZNF131 |
| hsa-mir-552-3p | ZNF24 |
| hsa-mir-552-3p | FAM222B |
| hsa-mir-552-3p | KDM2A |
| hsa-mir-95-3p | PLAC8L1 |
| hsa-mir-95-3p | RNF213 |
| hsa-mir-95-3p | RPL15 |
| hsa-mir-95-3p | PDZK1 |
| hsa-mir-95-3p | PDIK1L |
| hsa-mir-95-3p | LYSMD3 |
| hsa-mir-95-3p | KBTBD11 |
| hsa-mir-95-3p | KBTBD11 |
| hsa-mir-95-3p | LMNB2 |
| hsa-mir-95-3p | NFATC2IP |
| hsa-mir-95-3p | ZBTB41 |
| hsa-mir-95-3p | PSAPL1 |
| hsa-mir-95-3p | FJX1 |
| hsa-mir-95-3p | OAZ2 |
| hsa-mir-95-3p | POLR2A |
| hsa-mir-95-3p | POLR2A |
| hsa-mir-552-3p | SHMT2 |
| hsa-mir-95-3p | TRAPPC6B |
| hsa-mir-552-3p | TTC3 |
| hsa-mir-95-3p | C16orf72 |
| hsa-mir-552-3p | TMEM50A |
| hsa-mir-552-3p | AMER1 |
| hsa-mir-95-3p | C6orf120 |
| hsa-mir-95-3p | CCDC137 |
| hsa-mir-552-3p | PRKG1 |
| hsa-mir-552-3p | PCGF3 |
| hsa-mir-552-3p | CYP2R1 |
| hsa-mir-95-3p | LCE1E |
| hsa-mir-95-3p | HEXIM1 |
| hsa-mir-95-3p | MAGEH1 |
| hsa-mir-552-3p | EIF4EBP1 |
| hsa-mir-95-3p | FAM166A |
| hsa-mir-95-3p | SMIM15 |
| hsa-mir-552-3p | ARID2 |
| hsa-mir-552-3p | AFAP1 |
| hsa-mir-95-3p | ANXA4 |
| hsa-mir-95-3p | GMFB |
| hsa-mir-552-3p | SYNGAP1 |
| hsa-mir-552-3p | SYNGAP1 |
| hsa-mir-552-3p | CD2AP |
| hsa-mir-95-3p | MIER1 |
| hsa-mir-552-3p | MAN1A2 |
| hsa-mir-95-3p | SVIP |
| hsa-mir-95-3p | ZNF26 |
| hsa-mir-95-3p | MDM4 |
| hsa-mir-95-3p | CALM1 |
| hsa-mir-95-3p | MTOR |
| hsa-mir-95-3p | BRINP2 |
| hsa-mir-552-3p | DENND4B |
| hsa-mir-552-3p | TOP1 |
| hsa-mir-95-3p | SOWAHA |
| hsa-mir-95-3p | EFCAB7 |
| hsa-mir-552-3p | HLA-E |
| hsa-mir-552-3p | HLA-E |
| hsa-mir-552-3p | HLA-E |
| hsa-mir-552-3p | HLA-E |
| hsa-mir-552-3p | HLA-E |
| hsa-mir-552-3p | HLA-E |
| hsa-mir-552-3p | HLA-E |
| hsa-mir-95-3p | C12orf73 |
| hsa-mir-552-3p | SNX2 |
| hsa-mir-552-3p | HNRNPUL2 |
| hsa-mir-95-3p | ARFGAP3 |
| hsa-mir-552-3p | KCTD7 |
| hsa-mir-95-3p | EID1 |
| hsa-mir-95-3p | TAS2R38 |
| hsa-mir-552-3p | SPON1 |
| hsa-mir-95-3p | OTUD7B |
| hsa-mir-204-5p | ADCY5 |
| hsa-mir-204-5p | ADCYAP1R1 |
| hsa-mir-204-5p | ADORA3 |
| hsa-mir-204-5p | AK2 |
| hsa-mir-204-5p | ALPL |
| hsa-mir-204-5p | BIRC2 |
| hsa-mir-204-5p | ARHGAP6 |
| hsa-mir-204-5p | ARSE |
| hsa-mir-204-5p | ASGR1 |
| hsa-mir-204-5p | SERPINC1 |
| hsa-mir-204-5p | ZFHX3 |
| hsa-mir-204-5p | ATP2B1 |
| hsa-mir-204-5p | BCL2 |
| hsa-mir-204-5p | BCL2L2 |
| hsa-mir-204-5p | BCL7A |
| hsa-mir-204-5p | BDNF |
| hsa-mir-204-5p | BID |
| hsa-mir-204-5p | BMPR1A |
| hsa-mir-204-5p | MYRF |
| hsa-mir-204-5p | CACNA1C |
| hsa-mir-204-5p | CAMK2G |
| hsa-mir-204-5p | RUNX2 |
| hsa-mir-204-5p | CCNT2 |
| hsa-mir-204-5p | CD28 |
| hsa-mir-204-5p | CD44 |
| hsa-mir-204-5p | CDC42 |
| hsa-mir-204-5p | CDH1 |
| hsa-mir-204-5p | CDH2 |
| hsa-mir-204-5p | CDH4 |
| hsa-mir-204-5p | CDH8 |
| hsa-mir-204-5p | CDX2 |
| hsa-mir-204-5p | AP1S1 |
| hsa-mir-204-5p | CCR5 |
| hsa-mir-204-5p | CPT1B |
| hsa-mir-204-5p | CREB1 |
| hsa-mir-204-5p | ATF6B |
| hsa-mir-204-5p | CYBB |
| hsa-mir-204-5p | DHODH |
| hsa-mir-204-5p | DVL3 |
| hsa-mir-204-5p | EFNB2 |
| hsa-mir-204-5p | ENO1 |
| hsa-mir-204-5p | EPHA4 |
| hsa-mir-204-5p | ETF1 |
| hsa-mir-204-5p | ETV3 |
| hsa-mir-204-5p | BPTF |
| hsa-mir-204-5p | FOXC1 |
| hsa-mir-204-5p | FOXM1 |
| hsa-mir-204-5p | GALNT1 |
| hsa-mir-204-5p | GLP1R |
| hsa-mir-204-5p | GP2 |
| hsa-mir-204-5p | NR3C1 |
| hsa-mir-204-5p | CXCL3 |
| hsa-mir-204-5p | HARS |
| hsa-mir-204-5p | HAS2 |
| hsa-mir-204-5p | HLA-DRB1 |
| hsa-mir-204-5p | HLA-DRB5 |
| hsa-mir-204-5p | HLCS |
| hsa-mir-204-5p | HMX1 |
| hsa-mir-204-5p | HNRNPA2B1 |
| hsa-mir-204-5p | HOXA10 |
| hsa-mir-204-5p | HOXC8 |
| hsa-mir-204-5p | HSP90AA1 |
| hsa-mir-204-5p | HTR1F |
| hsa-mir-204-5p | IGFBP2 |
| hsa-mir-204-5p | IGFBP5 |
| hsa-mir-204-5p | IL1B |
| hsa-mir-204-5p | IL1RAP |
| hsa-mir-204-5p | CXCL8 |
| hsa-mir-204-5p | IL11 |
| hsa-mir-204-5p | ITGB3 |
| hsa-mir-204-5p | ITPR1 |
| hsa-mir-204-5p | JAK2 |
| hsa-mir-204-5p | JARID2 |
| hsa-mir-204-5p | LIPC |
| hsa-mir-204-5p | LSAMP |
| hsa-mir-204-5p | M6PR |
| hsa-mir-204-5p | CAPRIN1 |
| hsa-mir-204-5p | SMAD4 |
| hsa-mir-204-5p | SMAD6 |
| hsa-mir-204-5p | MAFG |
| hsa-mir-204-5p | MAGEB4 |
| hsa-mir-204-5p | MBNL1 |
| hsa-mir-204-5p | MDFI |
| hsa-mir-204-5p | MEIS1 |
| hsa-mir-204-5p | MEIS2 |
| hsa-mir-204-5p | MMP9 |
| hsa-mir-204-5p | ALDH6A1 |
| hsa-mir-204-5p | MUC4 |
| hsa-mir-204-5p | MX1 |
| hsa-mir-204-5p | MYOC |
| hsa-mir-204-5p | NFATC1 |
| hsa-mir-204-5p | NOTCH1 |
| hsa-mir-204-5p | NPTX1 |
| hsa-mir-204-5p | NTRK2 |
| hsa-mir-204-5p | SERPINE1 |
| hsa-mir-204-5p | PHKG1 |
| hsa-mir-204-5p | PLAG1 |
| hsa-mir-204-5p | PLAT |
| hsa-mir-204-5p | PLAUR |
| hsa-mir-204-5p | PLCG1 |
| hsa-mir-204-5p | SERPINF2 |
| hsa-mir-204-5p | POU2F1 |
| hsa-mir-204-5p | POU2F2 |
| hsa-mir-204-5p | PPP1CC |
| hsa-mir-204-5p | PPP3R1 |
| hsa-mir-204-5p | PRKAR1A |
| hsa-mir-204-5p | MAP2K1 |
| hsa-mir-204-5p | PRLR |
| hsa-mir-204-5p | PTGS2 |
| hsa-mir-204-5p | RAB5B |
| hsa-mir-204-5p | DPF2 |
| hsa-mir-204-5p | BRD2 |
| hsa-mir-204-5p | RORB |
| hsa-mir-204-5p | RPL3 |
| hsa-mir-204-5p | RPLP1 |
| hsa-mir-204-5p | ATXN1 |
| hsa-mir-204-5p | ATXN7 |
| hsa-mir-204-5p | SHOX2 |
| hsa-mir-204-5p | ST3GAL1 |
| hsa-mir-204-5p | SIX1 |
| hsa-mir-204-5p | SLC1A1 |
| hsa-mir-204-5p | SNAI2 |
| hsa-mir-204-5p | SNAI1 |
| hsa-mir-204-5p | SOD2 |
| hsa-mir-204-5p | SOS2 |
| hsa-mir-204-5p | SOX4 |
| hsa-mir-204-5p | SP1 |
| hsa-mir-204-5p | SPIB |
| hsa-mir-204-5p | TCF4 |
| hsa-mir-204-5p | TCF7L2 |
| hsa-mir-204-5p | TCF12 |
| hsa-mir-204-5p | TGFBR1 |
| hsa-mir-204-5p | TGFBR2 |
| hsa-mir-204-5p | THRB |
| hsa-mir-204-5p | UFD1 |
| hsa-mir-204-5p | VASP |
| hsa-mir-204-5p | EZR |
| hsa-mir-204-5p | VIM |
| hsa-mir-204-5p | ZNF22 |
| hsa-mir-204-5p | PRDM2 |
| hsa-mir-204-5p | CSDE1 |
| hsa-mir-204-5p | CXCR4 |
| hsa-mir-204-5p | HMGA2 |
| hsa-mir-204-5p | DPF3 |
| hsa-mir-204-5p | GAN |
| hsa-mir-204-5p | AKAP1 |
| hsa-mir-204-5p | MKKS |
| hsa-mir-204-5p | FZD1 |
| hsa-mir-204-5p | FZD8 |
| hsa-mir-204-5p | SPOP |
| hsa-mir-204-5p | SLC43A1 |
| hsa-mir-204-5p | DENR |
| hsa-mir-204-5p | STC2 |
| hsa-mir-204-5p | CDC23 |
| hsa-mir-204-5p | HRK |
| hsa-mir-204-5p | WISP1 |
| hsa-mir-204-5p | ALDH1A2 |
| hsa-mir-204-5p | SGPL1 |
| hsa-mir-204-5p | AP1S2 |
| hsa-mir-204-5p | CCRL2 |
| hsa-mir-204-5p | USP14 |
| hsa-mir-204-5p | SLC16A5 |
| hsa-mir-204-5p | SYNGR1 |
| hsa-mir-204-5p | TIAF1 |
| hsa-mir-204-5p | ZBTB22 |
| hsa-mir-204-5p | GTF3C3 |
| hsa-mir-204-5p | SLC22A6 |
| hsa-mir-204-5p | ARHGAP29 |
| hsa-mir-204-5p | KCNK6 |
| hsa-mir-204-5p | CREB5 |
| hsa-mir-204-5p | SH3PXD2A |
| hsa-mir-204-5p | BZW1 |
| hsa-mir-204-5p | EDEM1 |
| hsa-mir-204-5p | KNTC1 |
| hsa-mir-204-5p | TSC22D2 |
| hsa-mir-204-5p | ZNF623 |
| hsa-mir-204-5p | TECPR2 |
| hsa-mir-204-5p | G3BP2 |
| hsa-mir-204-5p | SH2B3 |
| hsa-mir-204-5p | FEM1B |
| hsa-mir-204-5p | FARP1 |
| hsa-mir-204-5p | PATJ |
| hsa-mir-204-5p | DCAF7 |
| hsa-mir-204-5p | VAV3 |
| hsa-mir-204-5p | IPO7 |
| hsa-mir-204-5p | TXNIP |
| hsa-mir-204-5p | POP4 |
| hsa-mir-204-5p | RPP40 |
| hsa-mir-204-5p | UTP14A |
| hsa-mir-204-5p | SRCAP |
| hsa-mir-204-5p | WDR3 |
| hsa-mir-204-5p | RAB10 |
| hsa-mir-204-5p | SERINC3 |
| hsa-mir-204-5p | PNRC1 |
| hsa-mir-204-5p | RAB40B |
| hsa-mir-204-5p | MAPRE2 |
| hsa-mir-204-5p | SLC27A2 |
| hsa-mir-204-5p | TLK2 |
| hsa-mir-204-5p | TPPP |
| hsa-mir-204-5p | NUPL2 |
| hsa-mir-204-5p | PTPRT |
| hsa-mir-204-5p | NUDT3 |
| hsa-mir-204-5p | AKAP13 |
| hsa-mir-204-5p | AKAP2 |
| hsa-mir-204-5p | DUSP10 |
| hsa-mir-204-5p | PADI2 |
| hsa-mir-204-5p | GPR45 |
| hsa-mir-204-5p | IKZF2 |
| hsa-mir-204-5p | VASH1 |
| hsa-mir-204-5p | CLUAP1 |
| hsa-mir-204-5p | ZBTB43 |
| hsa-mir-204-5p | FSTL4 |
| hsa-mir-204-5p | MRPS27 |
| hsa-mir-204-5p | IQCE |
| hsa-mir-204-5p | DNAJC16 |
| hsa-mir-204-5p | SIRT1 |
| hsa-mir-204-5p | ANGPTL2 |
| hsa-mir-204-5p | BRD4 |
| hsa-mir-204-5p | SEZ6L |
| hsa-mir-204-5p | CDC42EP4 |
| hsa-mir-204-5p | TMEFF2 |
| hsa-mir-204-5p | SPDEF |
| hsa-mir-204-5p | DNAJB5 |
| hsa-mir-204-5p | SEC31B |
| hsa-mir-204-5p | NGDN |
| hsa-mir-204-5p | CHD5 |
| hsa-mir-204-5p | HACL1 |
| hsa-mir-204-5p | TOR1AIP1 |
| hsa-mir-204-5p | ZBTB20 |
| hsa-mir-204-5p | CHORDC1 |
| hsa-mir-204-5p | AFF4 |
| hsa-mir-204-5p | AGO2 |
| hsa-mir-204-5p | SERP1 |
| hsa-mir-204-5p | BBS9 |
| hsa-mir-204-5p | RACGAP1 |
| hsa-mir-204-5p | HCFC2 |
| hsa-mir-204-5p | PRICKLE4 |
| hsa-mir-204-5p | A1CF |
| hsa-mir-204-5p | COL5A3 |
| hsa-mir-204-5p | SOST |
| hsa-mir-204-5p | AMOTL2 |
| hsa-mir-204-5p | SNX9 |
| hsa-mir-204-5p | UBE2J1 |
| hsa-mir-204-5p | ANKFY1 |
| hsa-mir-204-5p | RAB14 |
| hsa-mir-204-5p | RBM27 |
| hsa-mir-204-5p | UGT2B28 |
| hsa-mir-204-5p | CCDC93 |
| hsa-mir-204-5p | FNBP1L |
| hsa-mir-204-5p | CHCHD3 |
| hsa-mir-204-5p | 1-Mar |
| hsa-mir-204-5p | TTC38 |
| hsa-mir-204-5p | USP47 |
| hsa-mir-204-5p | ZWILCH |
| hsa-mir-204-5p | MRM3 |
| hsa-mir-204-5p | SLC38A7 |
| hsa-mir-204-5p | ELP2 |
| hsa-mir-204-5p | SYNJ2BP |
| hsa-mir-204-5p | SLC39A9 |
| hsa-mir-204-5p | STRBP |
| hsa-mir-204-5p | PI4K2A |
| hsa-mir-204-5p | ASIC4 |
| hsa-mir-204-5p | GALNT10 |
| hsa-mir-204-5p | ENAH |
| hsa-mir-204-5p | WWC3 |
| hsa-mir-204-5p | C21orf62 |
| hsa-mir-204-5p | ARNTL2 |
| hsa-mir-204-5p | TIGAR |
| hsa-mir-204-5p | VN1R1 |
| hsa-mir-204-5p | ARFGEF3 |
| hsa-mir-204-5p | RAB22A |
| hsa-mir-204-5p | AS3MT |
| hsa-mir-204-5p | MTA3 |
| hsa-mir-204-5p | SRGAP1 |
| hsa-mir-204-5p | SORCS2 |
| hsa-mir-204-5p | ZNF398 |
| hsa-mir-204-5p | JCAD |
| hsa-mir-204-5p | CACHD1 |
| hsa-mir-204-5p | SEMA4G |
| hsa-mir-204-5p | LY6G6D |
| hsa-mir-204-5p | TLNRD1 |
| hsa-mir-204-5p | CACNG8 |
| hsa-mir-204-5p | BACH2 |
| hsa-mir-204-5p | BCAN |
| hsa-mir-204-5p | SH2D4A |
| hsa-mir-204-5p | BLOC1S5 |
| hsa-mir-204-5p | SMOC1 |
| hsa-mir-204-5p | LRRC4 |
| hsa-mir-204-5p | PDF |
| hsa-mir-204-5p | GIGYF1 |
| hsa-mir-204-5p | TMPRSS3 |
| hsa-mir-204-5p | MRPS15 |
| hsa-mir-204-5p | SOWAHC |
| hsa-mir-204-5p | WNK3 |
| hsa-mir-204-5p | ZNF747 |
| hsa-mir-204-5p | ELOVL6 |
| hsa-mir-204-5p | ZNF576 |
| hsa-mir-204-5p | NOX5 |
| hsa-mir-204-5p | CEP97 |
| hsa-mir-204-5p | STEAP4 |
| hsa-mir-204-5p | ZYG11B |
| hsa-mir-204-5p | GTDC1 |
| hsa-mir-204-5p | ZNF385D |
| hsa-mir-204-5p | MCTP1 |
| hsa-mir-204-5p | FBXO31 |
| hsa-mir-204-5p | RNF122 |
| hsa-mir-204-5p | TM4SF20 |
| hsa-mir-204-5p | TMEM156 |
| hsa-mir-204-5p | WWC2 |
| hsa-mir-204-5p | TRPM3 |
| hsa-mir-204-5p | SH3BP5L |
| hsa-mir-204-5p | MAP1LC3B |
| hsa-mir-204-5p | FCRL4 |
| hsa-mir-204-5p | SPNS1 |
| hsa-mir-204-5p | RBM48 |
| hsa-mir-204-5p | CHCHD5 |
| hsa-mir-204-5p | DNAJC30 |
| hsa-mir-204-5p | RAB11FIP4 |
| hsa-mir-204-5p | FAXC |
| hsa-mir-204-5p | IL1F10 |
| hsa-mir-204-5p | BEX2 |
| hsa-mir-204-5p | FAM167B |
| hsa-mir-204-5p | ANKRD13A |
| hsa-mir-204-5p | SAMD1 |
| hsa-mir-204-5p | AP5B1 |
| hsa-mir-204-5p | ELMSAN1 |
| hsa-mir-204-5p | CHRDL1 |
| hsa-mir-204-5p | ATP6AP1L |
| hsa-mir-204-5p | MEX3A |
| hsa-mir-204-5p | MYOCD |
| hsa-mir-204-5p | SYAP1 |
| hsa-mir-204-5p | ABCC12 |
| hsa-mir-204-5p | MED8 |
| hsa-mir-204-5p | FAM83F |
| hsa-mir-204-5p | XKR4 |
| hsa-mir-204-5p | ZNF689 |
| hsa-mir-204-5p | ABHD15 |
| hsa-mir-204-5p | CLNK |
| hsa-mir-204-5p | ARAP2 |
| hsa-mir-204-5p | AGAP1 |
| hsa-mir-204-5p | C11orf74 |
| hsa-mir-204-5p | C17orf64 |
| hsa-mir-204-5p | CCDC43 |
| hsa-mir-204-5p | SLC43A2 |
| hsa-mir-204-5p | EID2B |
| hsa-mir-204-5p | KLHL40 |
| hsa-mir-204-5p | ADAT2 |
| hsa-mir-204-5p | XRRA1 |
| hsa-mir-204-5p | ODF4 |
| hsa-mir-204-5p | KCTD11 |
| hsa-mir-204-5p | SYT6 |
| hsa-mir-204-5p | PHF13 |
| hsa-mir-204-5p | ZNF362 |
| hsa-mir-204-5p | SRARP |
| hsa-mir-204-5p | TTC39B |
| hsa-mir-204-5p | TMTC2 |
| hsa-mir-204-5p | PAQR7 |
| hsa-mir-204-5p | MIER3 |
| hsa-mir-204-5p | ZNF48 |
| hsa-mir-204-5p | SLC39A11 |
| hsa-mir-204-5p | UNC13D |
| hsa-mir-204-5p | LRRC55 |
| hsa-mir-204-5p | ZCCHC24 |
| hsa-mir-204-5p | OPN5 |
| hsa-mir-204-5p | C6orf223 |
| hsa-mir-204-5p | KIAA1324L |
| hsa-mir-204-5p | ZDHHC20 |
| hsa-mir-204-5p | LY6G6F |
| hsa-mir-204-5p | BCL9L |
| hsa-mir-204-5p | B4GALNT3 |
| hsa-mir-204-5p | NUDT7 |
| hsa-mir-204-5p | KANSL1 |
| hsa-mir-204-5p | HCAR2 |
| hsa-mir-204-5p | ANKRD45 |
| hsa-mir-204-5p | PRSS38 |
| hsa-mir-204-5p | MYLK4 |
| hsa-mir-204-5p | ZSCAN22 |
| hsa-mir-204-5p | PLCXD3 |
| hsa-mir-204-5p | IRF2BP2 |
| hsa-mir-204-5p | SFT2D2 |
| hsa-mir-204-5p | MALAT1 |
| hsa-mir-204-5p | ZKSCAN4 |
| hsa-mir-204-5p | TBPL2 |
| hsa-mir-204-5p | SAMD5 |
| hsa-mir-204-5p | ONECUT3 |
| hsa-mir-204-5p | MYO18A |
| hsa-mir-204-5p | C4orf3 |
| hsa-mir-204-5p | ZNF705A |
| hsa-mir-204-5p | PALM2-AKAP2 |
| hsa-mir-204-5p | LURAP1 |
| hsa-mir-204-5p | HIST2H4B |
| hsa-mir-204-5p | KIAA0754 |
| hsa-mir-204-5p | LINC00598 |
| hsa-mir-204-5p | C6orf132 |
| hsa-mir-204-5p | UCA1 |
| hsa-mir-204-5p | TMEM236 |
| hsa-mir-204-5p | ZBTB8B |
| hsa-mir-204-5p | TEC |
| hsa-mir-204-5p | MICA |
| hsa-mir-122-5p | A2M |
| hsa-mir-122-5p | ABCF1 |
| hsa-mir-122-5p | ABL2 |
| hsa-mir-122-5p | ADAM10 |
| hsa-mir-122-5p | ADCY2 |
| hsa-mir-122-5p | GRK3 |
| hsa-mir-122-5p | ALDOA |
| hsa-mir-122-5p | ALOX5AP |
| hsa-mir-122-5p | ANG |
| hsa-mir-122-5p | ANK2 |
| hsa-mir-122-5p | ANXA7 |
| hsa-mir-122-5p | ANXA11 |
| hsa-mir-122-5p | XIAP |
| hsa-mir-122-5p | BIRC5 |
| hsa-mir-122-5p | RHOA |
| hsa-mir-122-5p | PHOX2A |
| hsa-mir-122-5p | ARL2 |
| hsa-mir-122-5p | ARSA |
| hsa-mir-122-5p | ARSB |
| hsa-mir-122-5p | ART3 |
| hsa-mir-122-5p | ATP1A2 |
| hsa-mir-122-5p | ATP7A |
| hsa-mir-122-5p | AXL |
| hsa-mir-122-5p | BAX |
| hsa-mir-122-5p | BCL2L1 |
| hsa-mir-122-5p | BCL2L2 |
| hsa-mir-122-5p | BPGM |
| hsa-mir-122-5p | BRCA2 |
| hsa-mir-122-5p | C3 |
| hsa-mir-122-5p | CALD1 |
| hsa-mir-122-5p | CALM3 |
| hsa-mir-122-5p | CALR |
| hsa-mir-122-5p | CALU |
| hsa-mir-122-5p | CASP7 |
| hsa-mir-122-5p | CCNG1 |
| hsa-mir-122-5p | SIGLEC6 |
| hsa-mir-122-5p | ENTPD1 |
| hsa-mir-122-5p | CDK4 |
| hsa-mir-122-5p | CENPF |
| hsa-mir-122-5p | CEACAM8 |
| hsa-mir-122-5p | AP1S1 |
| hsa-mir-122-5p | CCR6 |
| hsa-mir-122-5p | CNN3 |
| hsa-mir-122-5p | COL13A1 |
| hsa-mir-122-5p | COPA |
| hsa-mir-122-5p | SLC31A1 |
| hsa-mir-122-5p | CPA3 |
| hsa-mir-122-5p | CREB1 |
| hsa-mir-122-5p | CS |
| hsa-mir-122-5p | CSRP1 |
| hsa-mir-122-5p | CTPS1 |
| hsa-mir-122-5p | CYP3A5 |
| hsa-mir-122-5p | CYP7A1 |
| hsa-mir-122-5p | DBT |
| hsa-mir-122-5p | DMXL1 |
| hsa-mir-122-5p | DYNC1H1 |
| hsa-mir-122-5p | DUSP2 |
| hsa-mir-122-5p | ECE1 |
| hsa-mir-122-5p | EGFR |
| hsa-mir-122-5p | EYA4 |
| hsa-mir-122-5p | F2 |
| hsa-mir-122-5p | F2RL1 |
| hsa-mir-122-5p | FANCC |
| hsa-mir-122-5p | FHL2 |
| hsa-mir-122-5p | FUT8 |
| hsa-mir-122-5p | G6PC |
| hsa-mir-122-5p | GALNT3 |
| hsa-mir-122-5p | GFPT1 |
| hsa-mir-122-5p | B4GALT1 |
| hsa-mir-122-5p | GLUL |
| hsa-mir-122-5p | GP2 |
| hsa-mir-122-5p | FFAR1 |
| hsa-mir-122-5p | GRSF1 |
| hsa-mir-122-5p | GSTM3 |
| hsa-mir-122-5p | GTF2B |
| hsa-mir-122-5p | GTF2F1 |
| hsa-mir-122-5p | GTF2H2 |
| hsa-mir-122-5p | GYS1 |
| hsa-mir-122-5p | H1F0 |
| hsa-mir-122-5p | HCCS |
| hsa-mir-122-5p | UBE2K |
| hsa-mir-122-5p | HLA-DQA1 |
| hsa-mir-122-5p | HLA-E |
| hsa-mir-122-5p | HMOX1 |
| hsa-mir-122-5p | DNAJB1 |
| hsa-mir-122-5p | IDS |
| hsa-mir-122-5p | IFNA1 |
| hsa-mir-122-5p | IFNAR2 |
| hsa-mir-122-5p | IGF1R |
| hsa-mir-122-5p | IL1A |
| hsa-mir-122-5p | IL2RA |
| hsa-mir-122-5p | FOXK2 |
| hsa-mir-122-5p | KCNA7 |
| hsa-mir-122-5p | KRT10 |
| hsa-mir-122-5p | KRT14 |
| hsa-mir-122-5p | KRT18 |
| hsa-mir-122-5p | LAMP1 |
| hsa-mir-122-5p | ABLIM1 |
| hsa-mir-122-5p | LIMS1 |
| hsa-mir-122-5p | LRP3 |
| hsa-mir-122-5p | LYN |
| hsa-mir-122-5p | MARCKS |
| hsa-mir-122-5p | MAFG |
| hsa-mir-122-5p | MAZ |
| hsa-mir-122-5p | MCAM |
| hsa-mir-122-5p | MDM4 |
| hsa-mir-122-5p | MECP2 |
| hsa-mir-122-5p | MEF2D |
| hsa-mir-122-5p | MGAT1 |
| hsa-mir-122-5p | MKLN1 |
| hsa-mir-122-5p | MPV17 |
| hsa-mir-122-5p | MSN |
| hsa-mir-122-5p | MTAP |
| hsa-mir-122-5p | MYH11 |
| hsa-mir-122-5p | NARS |
| hsa-mir-122-5p | NASP |
| hsa-mir-122-5p | NCAM1 |
| hsa-mir-122-5p | NFE2L1 |
| hsa-mir-122-5p | NFX1 |
| hsa-mir-122-5p | NODAL |
| hsa-mir-122-5p | SLC11A2 |
| hsa-mir-122-5p | OMD |
| hsa-mir-122-5p | OLR1 |
| hsa-mir-122-5p | ORC2 |
| hsa-mir-122-5p | P4HA1 |
| hsa-mir-122-5p | PAK1 |
| hsa-mir-122-5p | PDK4 |
| hsa-mir-122-5p | PFDN1 |
| hsa-mir-122-5p | PFKFB2 |
| hsa-mir-122-5p | PHKA1 |
| hsa-mir-122-5p | PIK3R2 |
| hsa-mir-122-5p | PIP4K2A |
| hsa-mir-122-5p | PKM |
| hsa-mir-122-5p | PKNOX1 |
| hsa-mir-122-5p | PLAGL2 |
| hsa-mir-122-5p | PMP22 |
| hsa-mir-122-5p | POLR2D |
| hsa-mir-122-5p | PPIC |
| hsa-mir-122-5p | PRKAB1 |
| hsa-mir-122-5p | MAPK1 |
| hsa-mir-122-5p | MAPK11 |
| hsa-mir-122-5p | PSMB5 |
| hsa-mir-122-5p | PSMD10 |
| hsa-mir-122-5p | PSPH |
| hsa-mir-122-5p | QSOX1 |
| hsa-mir-122-5p | PTPN1 |
| hsa-mir-122-5p | PTPN2 |
| hsa-mir-122-5p | MAP4K2 |
| hsa-mir-122-5p | RAC1 |
| hsa-mir-122-5p | RAD21 |
| hsa-mir-122-5p | RBBP5 |
| hsa-mir-122-5p | RBL1 |
| hsa-mir-122-5p | REL |
| hsa-mir-122-5p | RFC2 |
| hsa-mir-122-5p | RPS15A |
| hsa-mir-122-5p | CLEC11A |
| hsa-mir-122-5p | SCN4B |
| hsa-mir-122-5p | SET |
| hsa-mir-122-5p | SH3GL1 |
| hsa-mir-122-5p | ST3GAL1 |
| hsa-mir-122-5p | SLC1A5 |
| hsa-mir-122-5p | SLC2A3 |
| hsa-mir-122-5p | SLC4A1 |
| hsa-mir-122-5p | SLC7A1 |
| hsa-mir-122-5p | SLC9A1 |
| hsa-mir-122-5p | SLC15A2 |
| hsa-mir-122-5p | SNTB2 |
| hsa-mir-122-5p | SOX2 |
| hsa-mir-122-5p | SPIB |
| hsa-mir-122-5p | SRF |
| hsa-mir-122-5p | SSR3 |
| hsa-mir-122-5p | SSTR2 |
| hsa-mir-122-5p | STXBP2 |
| hsa-mir-122-5p | ADAM17 |
| hsa-mir-122-5p | TCP11 |
| hsa-mir-122-5p | TEP1 |
| hsa-mir-122-5p | TERF2 |
| hsa-mir-122-5p | TFDP2 |
| hsa-mir-122-5p | TGFB1 |
| hsa-mir-122-5p | TIAL1 |
| hsa-mir-122-5p | TPD52L2 |
| hsa-mir-122-5p | UBE2G2 |
| hsa-mir-122-5p | UBE2L3 |
| hsa-mir-122-5p | SUMO1 |
| hsa-mir-122-5p | UBTF |
| hsa-mir-122-5p | UROS |
| hsa-mir-122-5p | VDR |
| hsa-mir-122-5p | VEGFC |
| hsa-mir-122-5p | VHL |
| hsa-mir-122-5p | WNT1 |
| hsa-mir-122-5p | YWHAB |
| hsa-mir-122-5p | ZNF74 |
| hsa-mir-122-5p | ZNF154 |
| hsa-mir-122-5p | ZNF226 |
| hsa-mir-122-5p | LUZP1 |
| hsa-mir-122-5p | MAFK |
| hsa-mir-122-5p | ARHGEF5 |
| hsa-mir-122-5p | MLF2 |
| hsa-mir-122-5p | SLC7A5 |
| hsa-mir-122-5p | SLC10A3 |
| hsa-mir-122-5p | IFITM1 |
| hsa-mir-122-5p | DGKE |
| hsa-mir-122-5p | MAPKAPK5 |
| hsa-mir-122-5p | PRKRA |
| hsa-mir-122-5p | SOCS1 |
| hsa-mir-122-5p | PEA15 |
| hsa-mir-122-5p | TNFSF14 |
| hsa-mir-122-5p | SUCLA2 |
| hsa-mir-122-5p | CDKL1 |
| hsa-mir-122-5p | WASF1 |
| hsa-mir-122-5p | SPAG9 |
| hsa-mir-122-5p | TBX19 |
| hsa-mir-122-5p | USP10 |
| hsa-mir-122-5p | SLC16A5 |
| hsa-mir-122-5p | LRAT |
| hsa-mir-122-5p | RPS6KA5 |
| hsa-mir-122-5p | NUMBL |
| hsa-mir-122-5p | CD83 |
| hsa-mir-122-5p | EFTUD2 |
| hsa-mir-122-5p | TGFBRAP1 |
| hsa-mir-122-5p | ZNF264 |
| hsa-mir-122-5p | MED7 |
| hsa-mir-122-5p | CHST3 |
| hsa-mir-122-5p | AKAP5 |
| hsa-mir-122-5p | NPEPPS |
| hsa-mir-122-5p | ENTPD4 |
| hsa-mir-122-5p | PHF14 |
| hsa-mir-122-5p | TRAM2 |
| hsa-mir-122-5p | KIAA0100 |
| hsa-mir-122-5p | PCLAF |
| hsa-mir-122-5p | BMS1 |
| hsa-mir-122-5p | IST1 |
| hsa-mir-122-5p | TOMM70 |
| hsa-mir-122-5p | FIG4 |
| hsa-mir-122-5p | NCAPD2 |
| hsa-mir-122-5p | HS3ST1 |
| hsa-mir-122-5p | CCS |
| hsa-mir-122-5p | AKT3 |
| hsa-mir-122-5p | ABCF2 |
| hsa-mir-122-5p | OPTN |
| hsa-mir-122-5p | PRMT3 |
| hsa-mir-122-5p | TRIB1 |
| hsa-mir-122-5p | DCAF7 |
| hsa-mir-122-5p | CALCOCO2 |
| hsa-mir-122-5p | SPRY2 |
| hsa-mir-122-5p | FSTL3 |
| hsa-mir-122-5p | PRSS16 |
| hsa-mir-122-5p | SF3A1 |
| hsa-mir-122-5p | SEC23B |
| hsa-mir-122-5p | SPTLC1 |
| hsa-mir-122-5p | SLC19A2 |
| hsa-mir-122-5p | CDC42EP3 |
| hsa-mir-122-5p | YKT6 |
| hsa-mir-122-5p | GMEB1 |
| hsa-mir-122-5p | YME1L1 |
| hsa-mir-122-5p | KIF1C |
| hsa-mir-122-5p | TOB2 |
| hsa-mir-122-5p | RAB10 |
| hsa-mir-122-5p | GPR75 |
| hsa-mir-122-5p | KDELR1 |
| hsa-mir-122-5p | AP3M2 |
| hsa-mir-122-5p | PNRC1 |
| hsa-mir-122-5p | ERP29 |
| hsa-mir-122-5p | LILRA2 |
| hsa-mir-122-5p | BTN3A2 |
| hsa-mir-122-5p | KAT7 |
| hsa-mir-122-5p | NUDT3 |
| hsa-mir-122-5p | AKAP11 |
| hsa-mir-122-5p | GALNT6 |
| hsa-mir-122-5p | PXMP4 |
| hsa-mir-122-5p | Cux1 |
| hsa-mir-122-5p | HSPA4L |
| hsa-mir-122-5p | SLITRK3 |
| hsa-mir-122-5p | MLXIP |
| hsa-mir-122-5p | FOXJ3 |
| hsa-mir-122-5p | MAPRE1 |
| hsa-mir-122-5p | POMZP3 |
| hsa-mir-122-5p | FBXO21 |
| hsa-mir-122-5p | UNC13A |
| hsa-mir-122-5p | SMG1 |
| hsa-mir-122-5p | NMNAT2 |
| hsa-mir-122-5p | ERC1 |
| hsa-mir-122-5p | PEG10 |
| hsa-mir-122-5p | CDK19 |
| hsa-mir-122-5p | NCDN |
| hsa-mir-122-5p | MPRIP |
| hsa-mir-122-5p | TTLL12 |
| hsa-mir-122-5p | LPIN1 |
| hsa-mir-122-5p | XPO6 |
| hsa-mir-122-5p | SIK2 |
| hsa-mir-122-5p | WWC1 |
| hsa-mir-122-5p | CTDNEP1 |
| hsa-mir-122-5p | GPR161 |
| hsa-mir-122-5p | SLC44A1 |
| hsa-mir-122-5p | ABCA6 |
| hsa-mir-122-5p | QPRT |
| hsa-mir-122-5p | POFUT1 |
| hsa-mir-122-5p | ZNF281 |
| hsa-mir-122-5p | DNPEP |
| hsa-mir-122-5p | HEBP2 |
| hsa-mir-122-5p | ORC6 |
| hsa-mir-122-5p | ACOT9 |
| hsa-mir-122-5p | PATZ1 |
| hsa-mir-122-5p | PHLDA3 |
| hsa-mir-122-5p | TRIM29 |
| hsa-mir-122-5p | SLC7A11 |
| hsa-mir-122-5p | OSBP2 |
| hsa-mir-122-5p | MAFF |
| hsa-mir-122-5p | DSTYK |
| hsa-mir-122-5p | FBXO7 |
| hsa-mir-122-5p | BAMBI |
| hsa-mir-122-5p | MTO1 |
| hsa-mir-122-5p | TECPR1 |
| hsa-mir-122-5p | CLIC4 |
| hsa-mir-122-5p | HEATR5A |
| hsa-mir-122-5p | RAI14 |
| hsa-mir-122-5p | PYGO1 |
| hsa-mir-122-5p | WSB1 |
| hsa-mir-122-5p | ZNF658 |
| hsa-mir-122-5p | MYCBP |
| hsa-mir-122-5p | STAU2 |
| hsa-mir-122-5p | FOXP1 |
| hsa-mir-122-5p | ST6GALNAC4 |
| hsa-mir-122-5p | TNRC6A |
| hsa-mir-122-5p | RABGEF1 |
| hsa-mir-122-5p | PHPT1 |
| hsa-mir-122-5p | RACGAP1 |
| hsa-mir-122-5p | PARVB |
| hsa-mir-122-5p | OLA1 |
| hsa-mir-122-5p | GPSM2 |
| hsa-mir-122-5p | EEF2K |
| hsa-mir-122-5p | AK3 |
| hsa-mir-122-5p | HDDC2 |
| hsa-mir-122-5p | GLOD4 |
| hsa-mir-122-5p | TUBD1 |
| hsa-mir-122-5p | ACP6 |
| hsa-mir-122-5p | NT5C3A |
| hsa-mir-122-5p | NIP7 |
| hsa-mir-122-5p | CINP |
| hsa-mir-122-5p | RAB6B |
| hsa-mir-122-5p | GDE1 |
| hsa-mir-122-5p | ESF1 |
| hsa-mir-122-5p | MRPS23 |
| hsa-mir-122-5p | MSRB1 |
| hsa-mir-122-5p | NLGN3 |
| hsa-mir-122-5p | TAS2R5 |
| hsa-mir-122-5p | RNF216 |
| hsa-mir-122-5p | TMCO1 |
| hsa-mir-122-5p | RBM47 |
| hsa-mir-122-5p | GNL3L |
| hsa-mir-122-5p | INO80 |
| hsa-mir-122-5p | MED18 |
| hsa-mir-122-5p | PIGG |
| hsa-mir-122-5p | ZCCHC2 |
| hsa-mir-122-5p | RHBDL2 |
| hsa-mir-122-5p | PIGX |
| hsa-mir-122-5p | FAM118A |
| hsa-mir-122-5p | PTCD3 |
| hsa-mir-122-5p | RBM23 |
| hsa-mir-122-5p | SBNO1 |
| hsa-mir-122-5p | VPS53 |
| hsa-mir-122-5p | C4orf19 |
| hsa-mir-122-5p | TMEM40 |
| hsa-mir-122-5p | SPTLC3 |
| hsa-mir-122-5p | CHDH |
| hsa-mir-122-5p | NCBP3 |
| hsa-mir-122-5p | CHST12 |
| hsa-mir-122-5p | SLC35E3 |
| hsa-mir-122-5p | RBM38 |
| hsa-mir-122-5p | GALNT10 |
| hsa-mir-122-5p | NAGK |
| hsa-mir-122-5p | MED29 |
| hsa-mir-122-5p | ANKRD10 |
| hsa-mir-122-5p | TBC1D22B |
| hsa-mir-122-5p | MREG |
| hsa-mir-122-5p | LUC7L |
| hsa-mir-122-5p | HHAT |
| hsa-mir-122-5p | PRR11 |
| hsa-mir-122-5p | METTL2B |
| hsa-mir-122-5p | LRP2BP |
| hsa-mir-122-5p | EMC3 |
| hsa-mir-122-5p | UBAP2 |
| hsa-mir-122-5p | ZC3H15 |
| hsa-mir-122-5p | ZNF395 |
| hsa-mir-122-5p | KLHL7 |
| hsa-mir-122-5p | ALG1 |
| hsa-mir-122-5p | TDRD1 |
| hsa-mir-122-5p | SAR1A |
| hsa-mir-122-5p | KCMF1 |
| hsa-mir-122-5p | PARP11 |
| hsa-mir-122-5p | LYRM4 |
| hsa-mir-122-5p | APMAP |
| hsa-mir-122-5p | JPH2 |
| hsa-mir-122-5p | CLK4 |
| hsa-mir-122-5p | CYP20A1 |
| hsa-mir-122-5p | KIAA1143 |
| hsa-mir-122-5p | ESYT2 |
| hsa-mir-122-5p | XPO5 |
| hsa-mir-122-5p | HECW2 |
| hsa-mir-122-5p | PDP2 |
| hsa-mir-122-5p | EP400 |
| hsa-mir-122-5p | USP28 |
| hsa-mir-122-5p | ZBTB4 |
| hsa-mir-122-5p | ZFP14 |
| hsa-mir-122-5p | CPNE5 |
| hsa-mir-122-5p | POLD4 |
| hsa-mir-122-5p | MRPL17 |
| hsa-mir-122-5p | FAM217B |
| hsa-mir-122-5p | CLSPN |
| hsa-mir-122-5p | NOD2 |
| hsa-mir-122-5p | ZNF106 |
| hsa-mir-122-5p | TMEM168 |
| hsa-mir-122-5p | MRPS25 |
| hsa-mir-122-5p | NOM1 |
| hsa-mir-122-5p | SMURF2 |
| hsa-mir-122-5p | MEAF6 |
| hsa-mir-122-5p | EFCAB6 |
| hsa-mir-122-5p | KRI1 |
| hsa-mir-122-5p | AACS |
| hsa-mir-122-5p | FUNDC2 |
| hsa-mir-122-5p | CENPM |
| hsa-mir-122-5p | ZNF655 |
| hsa-mir-122-5p | GLB1L |
| hsa-mir-122-5p | ATP13A3 |
| hsa-mir-122-5p | NKAP |
| hsa-mir-122-5p | SLC52A2 |
| hsa-mir-122-5p | PGBD5 |
| hsa-mir-122-5p | HECTD3 |
| hsa-mir-122-5p | ZYG11B |
| hsa-mir-122-5p | TBL1XR1 |
| hsa-mir-122-5p | MOB3B |
| hsa-mir-122-5p | MFSD13A |
| hsa-mir-122-5p | SNX22 |
| hsa-mir-122-5p | ACTR5 |
| hsa-mir-122-5p | ADM2 |
| hsa-mir-122-5p | TNIP3 |
| hsa-mir-122-5p | SYNPO2L |
| hsa-mir-122-5p | SLC35E1 |
| hsa-mir-122-5p | PLEKHS1 |
| hsa-mir-122-5p | NOL10 |
| hsa-mir-122-5p | CCDC170 |
| hsa-mir-122-5p | NAA50 |
| hsa-mir-122-5p | RAB11FIP1 |
| hsa-mir-122-5p | ORAI2 |
| hsa-mir-122-5p | CXorf21 |
| hsa-mir-122-5p | HM13 |
| hsa-mir-122-5p | ANKRD13C |
| hsa-mir-122-5p | NIPA2 |
| hsa-mir-122-5p | TIGD6 |
| hsa-mir-122-5p | RNF170 |
| hsa-mir-122-5p | THAP2 |
| hsa-mir-122-5p | KATNAL1 |
| hsa-mir-122-5p | MAGT1 |
| hsa-mir-122-5p | MRI1 |
| hsa-mir-122-5p | YIPF4 |
| hsa-mir-122-5p | SLC25A33 |
| hsa-mir-122-5p | MCM8 |
| hsa-mir-122-5p | DCTN5 |
| hsa-mir-122-5p | MT4 |
| hsa-mir-122-5p | MFSD14B |
| hsa-mir-122-5p | ZNF347 |
| hsa-mir-122-5p | PPP1R9B |
| hsa-mir-122-5p | PIGO |
| hsa-mir-122-5p | CNDP1 |
| hsa-mir-122-5p | LMNB2 |
| hsa-mir-122-5p | LRCH3 |
| hsa-mir-122-5p | CCDC142 |
| hsa-mir-122-5p | ADO |
| hsa-mir-122-5p | POMGNT2 |
| hsa-mir-122-5p | PLXDC2 |
| hsa-mir-122-5p | NFATC2IP |
| hsa-mir-122-5p | CEP89 |
| hsa-mir-122-5p | UTP4 |
| hsa-mir-122-5p | LRP11 |
| hsa-mir-122-5p | MASTL |
| hsa-mir-122-5p | SERAC1 |
| hsa-mir-122-5p | PRPF38A |
| hsa-mir-122-5p | RRP36 |
| hsa-mir-122-5p | SIGLEC12 |
| hsa-mir-122-5p | C16orf45 |
| hsa-mir-122-5p | TMEM250 |
| hsa-mir-122-5p | ZSWIM1 |
| hsa-mir-122-5p | ZNF160 |
| hsa-mir-122-5p | TIMM29 |
| hsa-mir-122-5p | STARD13 |
| hsa-mir-122-5p | MFSD4B |
| hsa-mir-122-5p | LIN52 |
| hsa-mir-122-5p | SPECC1 |
| hsa-mir-122-5p | G6PC3 |
| hsa-mir-122-5p | TIMM50 |
| hsa-mir-122-5p | SYAP1 |
| hsa-mir-122-5p | EGLN3 |
| hsa-mir-122-5p | GTF3C6 |
| hsa-mir-122-5p | XKR4 |
| hsa-mir-122-5p | RNF157 |
| hsa-mir-122-5p | SMYD4 |
| hsa-mir-122-5p | OSBPL10 |
| hsa-mir-122-5p | FLYWCH2 |
| hsa-mir-122-5p | WDR31 |
| hsa-mir-122-5p | ZNF618 |
| hsa-mir-122-5p | BATF2 |
| hsa-mir-122-5p | FAM210B |
| hsa-mir-122-5p | MOGAT1 |
| hsa-mir-122-5p | WDR17 |
| hsa-mir-122-5p | SLC16A10 |
| hsa-mir-122-5p | ZNF354B |
| hsa-mir-122-5p | BORCS7 |
| hsa-mir-122-5p | ANKRD9 |
| hsa-mir-122-5p | MRPL52 |
| hsa-mir-122-5p | GPHB5 |
| hsa-mir-122-5p | CYB5D1 |
| hsa-mir-122-5p | KRBA2 |
| hsa-mir-122-5p | CCDC43 |
| hsa-mir-122-5p | ZNF573 |
| hsa-mir-122-5p | FBXO27 |
| hsa-mir-122-5p | RNF19B |
| hsa-mir-122-5p | C1orf122 |
| hsa-mir-122-5p | TSHZ2 |
| hsa-mir-122-5p | ACVR1C |
| hsa-mir-122-5p | CPNE4 |
| hsa-mir-122-5p | GNPDA2 |
| hsa-mir-122-5p | ZNF786 |
| hsa-mir-122-5p | MTPN |
| hsa-mir-122-5p | MPLKIP |
| hsa-mir-122-5p | PTPDC1 |
| hsa-mir-122-5p | DOCK11 |
| hsa-mir-122-5p | BRI3BP |
| hsa-mir-122-5p | SMCR8 |
| hsa-mir-122-5p | ROMO1 |
| hsa-mir-122-5p | KDELC2 |
| hsa-mir-122-5p | PTGR2 |
| hsa-mir-122-5p | RUNDC1 |
| hsa-mir-122-5p | BROX |
| hsa-mir-122-5p | TMEM56 |
| hsa-mir-122-5p | DUSP18 |
| hsa-mir-122-5p | FAM117B |
| hsa-mir-122-5p | TCF23 |
| hsa-mir-122-5p | GPR155 |
| hsa-mir-122-5p | SGO1 |
| hsa-mir-122-5p | THAP6 |
| hsa-mir-122-5p | SLC38A9 |
| hsa-mir-122-5p | METTL27 |
| hsa-mir-122-5p | TMEM74 |
| hsa-mir-122-5p | FAM120AOS |
| hsa-mir-122-5p | PGBD4 |
| hsa-mir-122-5p | ZNF320 |
| hsa-mir-122-5p | DENND2C |
| hsa-mir-122-5p | LCA5 |
| hsa-mir-122-5p | FUT11 |
| hsa-mir-122-5p | ZNF431 |
| hsa-mir-122-5p | FAM9B |
| hsa-mir-122-5p | AGO3 |
| hsa-mir-122-5p | DZIP1L |
| hsa-mir-122-5p | ALG14 |
| hsa-mir-122-5p | IBA57 |
| hsa-mir-122-5p | APOBEC3A |
| hsa-mir-122-5p | TRIM65 |
| hsa-mir-122-5p | ZSCAN4 |
| hsa-mir-122-5p | DNAJC18 |
| hsa-mir-122-5p | VMA21 |
| hsa-mir-122-5p | CDY2B |
| hsa-mir-122-5p | TMEM136 |
| hsa-mir-122-5p | ZNF485 |
| hsa-mir-122-5p | KIF6 |
| hsa-mir-122-5p | AKR7L |
| hsa-mir-122-5p | SLC25A30 |
| hsa-mir-122-5p | GK5 |
| hsa-mir-122-5p | NALCN |
| hsa-mir-122-5p | FADS6 |
| hsa-mir-122-5p | NKPD1 |
| hsa-mir-122-5p | ZNF841 |
| hsa-mir-122-5p | FAM19A3 |
| hsa-mir-122-5p | SEC14L4 |
| hsa-mir-122-5p | RABL3 |
| hsa-mir-122-5p | XKR6 |
| hsa-mir-122-5p | DPY19L4 |
| hsa-mir-122-5p | C14orf39 |
| hsa-mir-122-5p | RAB43 |
| hsa-mir-122-5p | ACER2 |
| hsa-mir-122-5p | VSIG1 |
| hsa-mir-122-5p | FMN1 |
| hsa-mir-122-5p | SMTNL2 |
| hsa-mir-122-5p | MOGAT3 |
| hsa-mir-122-5p | FAM71F2 |
| hsa-mir-122-5p | ZNF233 |
| hsa-mir-122-5p | DNAJB13 |
| hsa-mir-122-5p | ANKRD36 |
| hsa-mir-122-5p | RBM43 |
| hsa-mir-122-5p | C3orf62 |
| hsa-mir-122-5p | VWC2 |
| hsa-mir-122-5p | ZNF322P1 |
| hsa-mir-122-5p | RGS9BP |
| hsa-mir-122-5p | FLG2 |
| hsa-mir-122-5p | PLEKHM3 |
| hsa-mir-122-5p | IYD |
| hsa-mir-122-5p | FAM102A |
| hsa-mir-122-5p | ZNF321P |
| hsa-mir-122-5p | HACD4 |
| hsa-mir-122-5p | BCL2L15 |
| hsa-mir-122-5p | CISD2 |
| hsa-mir-122-5p | AGAP9 |
| hsa-mir-122-5p | ARIH2OS |
| hsa-mir-122-5p | ZBTB8A |
| hsa-mir-122-5p | POM121L7P |
| hsa-mir-122-5p | SLC35E2B |
| hsa-mir-122-5p | PRR23A |
| hsa-mir-122-5p | ISPD |
| hsa-mir-122-5p | LRRC3C |
| hsa-mir-122-5p | ARL17B |
| hsa-mir-122-5p | ISY1-RAB43 |
| hsa-mir-204-5p | BIRC2 |
| hsa-mir-204-5p | ATP2B1 |
| hsa-mir-204-5p | BCL2 |
| hsa-mir-204-5p | BCL2L2 |
| hsa-mir-204-5p | CDH2 |
| hsa-mir-204-5p | AP1S1 |
| hsa-mir-204-5p | EPHA4 |
| hsa-mir-204-5p | BPTF |
| hsa-mir-204-5p | FOXC1 |
| hsa-mir-204-5p | CXCL3 |
| hsa-mir-204-5p | HAS2 |
| hsa-mir-204-5p | HOXA10 |
| hsa-mir-204-5p | HOXC8 |
| hsa-mir-204-5p | HSP90AA1 |
| hsa-mir-204-5p | IL1B |
| hsa-mir-204-5p | IL1RAP |
| hsa-mir-204-5p | IL11 |
| hsa-mir-204-5p | ITGB3 |
| hsa-mir-204-5p | ITPR1 |
| hsa-mir-204-5p | JARID2 |
| hsa-mir-204-5p | M6PR |
| hsa-mir-204-5p | CAPRIN1 |
| hsa-mir-204-5p | MEIS1 |
| hsa-mir-204-5p | MYOC |
| hsa-mir-204-5p | NPTX1 |
| hsa-mir-204-5p | NTRK2 |
| hsa-mir-204-5p | SERPINE1 |
| hsa-mir-204-5p | PLAT |
| hsa-mir-204-5p | PLAUR |
| hsa-mir-204-5p | PLCG1 |
| hsa-mir-204-5p | DPF2 |
| hsa-mir-204-5p | BRD2 |
| hsa-mir-204-5p | ST3GAL1 |
| hsa-mir-204-5p | SNAI2 |
| hsa-mir-204-5p | SNAI1 |
| hsa-mir-204-5p | SOD2 |
| hsa-mir-204-5p | SOX4 |
| hsa-mir-204-5p | TCF4 |
| hsa-mir-204-5p | TCF12 |
| hsa-mir-204-5p | TGFBR1 |
| hsa-mir-204-5p | TGFBR2 |
| hsa-mir-204-5p | EZR |
| hsa-mir-204-5p | CSDE1 |
| hsa-mir-204-5p | AKAP1 |
| hsa-mir-204-5p | FZD1 |
| hsa-mir-204-5p | CDC23 |
| hsa-mir-204-5p | SGPL1 |
| hsa-mir-204-5p | AP1S2 |
| hsa-mir-204-5p | ARHGAP29 |
| hsa-mir-204-5p | SH3PXD2A |
| hsa-mir-204-5p | EDEM1 |
| hsa-mir-204-5p | G3BP2 |
| hsa-mir-204-5p | FARP1 |
| hsa-mir-204-5p | DCAF7 |
| hsa-mir-204-5p | TXNIP |
| hsa-mir-204-5p | SERINC3 |
| hsa-mir-204-5p | RAB40B |
| hsa-mir-204-5p | MAPRE2 |
| hsa-mir-204-5p | TPPP |
| hsa-mir-204-5p | SERP1 |
| hsa-mir-204-5p | COL5A3 |
| hsa-mir-204-5p | RAB14 |
| hsa-mir-204-5p | USP47 |
| hsa-mir-204-5p | ENAH |
| hsa-mir-204-5p | RAB22A |
| hsa-mir-204-5p | SMOC1 |
| hsa-mir-204-5p | SOWAHC |
| hsa-mir-204-5p | BEX2 |
| hsa-mir-204-5p | ADAT2 |
| hsa-mir-204-5p | IRF2BP2 |
| hsa-mir-122-5p | A2M |
| hsa-mir-122-5p | ABL2 |
| hsa-mir-122-5p | ADAM10 |
| hsa-mir-122-5p | ALDOA |
| hsa-mir-122-5p | ANK2 |
| hsa-mir-122-5p | ANXA7 |
| hsa-mir-122-5p | ANXA11 |
| hsa-mir-122-5p | PHOX2A |
| hsa-mir-122-5p | ARL2 |
| hsa-mir-122-5p | ARSB |
| hsa-mir-122-5p | ART3 |
| hsa-mir-122-5p | ATP1A2 |
| hsa-mir-122-5p | ATP7A |
| hsa-mir-122-5p | BAX |
| hsa-mir-122-5p | BCL2L2 |
| hsa-mir-122-5p | BPGM |
| hsa-mir-122-5p | BRCA2 |
| hsa-mir-122-5p | CALD1 |
| hsa-mir-122-5p | CALR |
| hsa-mir-122-5p | CALU |
| hsa-mir-122-5p | CASP7 |
| hsa-mir-122-5p | CCNG1 |
| hsa-mir-122-5p | CDK4 |
| hsa-mir-122-5p | CENPF |
| hsa-mir-122-5p | CEACAM8 |
| hsa-mir-122-5p | CNN3 |
| hsa-mir-122-5p | CPA3 |
| hsa-mir-122-5p | CREB1 |
| hsa-mir-122-5p | CSRP1 |
| hsa-mir-122-5p | CTPS1 |
| hsa-mir-122-5p | CYP3A5 |
| hsa-mir-122-5p | CYP7A1 |
| hsa-mir-122-5p | DBT |
| hsa-mir-122-5p | DMXL1 |
| hsa-mir-122-5p | DYNC1H1 |
| hsa-mir-122-5p | DUSP2 |
| hsa-mir-122-5p | EGFR |
| hsa-mir-122-5p | EYA4 |
| hsa-mir-122-5p | GALNT3 |
| hsa-mir-122-5p | B4GALT1 |
| hsa-mir-122-5p | GP2 |
| hsa-mir-122-5p | GSTM3 |
| hsa-mir-122-5p | GTF2H2 |
| hsa-mir-122-5p | GYS1 |
| hsa-mir-122-5p | HCCS |
| hsa-mir-122-5p | UBE2K |
| hsa-mir-122-5p | HLA-DQA1 |
| hsa-mir-122-5p | HMOX1 |
| hsa-mir-122-5p | DNAJB1 |
| hsa-mir-122-5p | IDS |
| hsa-mir-122-5p | IFNA1 |
| hsa-mir-122-5p | IGF1R |
| hsa-mir-122-5p | FOXK2 |
| hsa-mir-122-5p | KRT10 |
| hsa-mir-122-5p | KRT14 |
| hsa-mir-122-5p | KRT18 |
| hsa-mir-122-5p | LAMP1 |
| hsa-mir-122-5p | ABLIM1 |
| hsa-mir-122-5p | MARCKS |
| hsa-mir-122-5p | MAZ |
| hsa-mir-122-5p | MDM4 |
| hsa-mir-122-5p | MECP2 |
| hsa-mir-122-5p | MEF2D |
| hsa-mir-122-5p | MKLN1 |
| hsa-mir-122-5p | MPV17 |
| hsa-mir-122-5p | NASP |
| hsa-mir-122-5p | NCAM1 |
| hsa-mir-122-5p | NFX1 |
| hsa-mir-122-5p | NODAL |
| hsa-mir-122-5p | SLC11A2 |
| hsa-mir-122-5p | OLR1 |
| hsa-mir-122-5p | ORC2 |
| hsa-mir-122-5p | P4HA1 |
| hsa-mir-122-5p | PAK1 |
| hsa-mir-122-5p | PFDN1 |
| hsa-mir-122-5p | PFKFB2 |
| hsa-mir-122-5p | PHKA1 |
| hsa-mir-122-5p | PIP4K2A |
| hsa-mir-122-5p | PKM |
| hsa-mir-122-5p | PLAGL2 |
| hsa-mir-122-5p | PMP22 |
| hsa-mir-122-5p | MAPK11 |
| hsa-mir-122-5p | PSMD10 |
| hsa-mir-122-5p | PSPH |
| hsa-mir-122-5p | RAC1 |
| hsa-mir-122-5p | RAD21 |
| hsa-mir-122-5p | RBBP5 |
| hsa-mir-122-5p | RBL1 |
| hsa-mir-122-5p | RPS15A |
| hsa-mir-122-5p | CLEC11A |
| hsa-mir-122-5p | SCN4B |
| hsa-mir-122-5p | SET |
| hsa-mir-122-5p | SLC1A5 |
| hsa-mir-122-5p | SLC2A3 |
| hsa-mir-122-5p | SLC7A1 |
| hsa-mir-122-5p | SLC9A1 |
| hsa-mir-122-5p | SLC15A2 |
| hsa-mir-122-5p | SOX2 |
| hsa-mir-122-5p | SSR3 |
| hsa-mir-122-5p | ADAM17 |
| hsa-mir-122-5p | TCP11 |
| hsa-mir-122-5p | TFDP2 |
| hsa-mir-122-5p | TPD52L2 |
| hsa-mir-122-5p | UBE2L3 |
| hsa-mir-122-5p | VHL |
| hsa-mir-122-5p | LUZP1 |
| hsa-mir-122-5p | SLC7A5 |
| hsa-mir-122-5p | SLC10A3 |
| hsa-mir-122-5p | PRKRA |
| hsa-mir-122-5p | PEA15 |
| hsa-mir-122-5p | SUCLA2 |
| hsa-mir-122-5p | WASF1 |
| hsa-mir-122-5p | SPAG9 |
| hsa-mir-122-5p | TBX19 |
| hsa-mir-122-5p | USP10 |
| hsa-mir-122-5p | NUMBL |
| hsa-mir-122-5p | CD83 |
| hsa-mir-122-5p | TGFBRAP1 |
| hsa-mir-122-5p | ZNF264 |
| hsa-mir-122-5p | CHST3 |
| hsa-mir-122-5p | NPEPPS |
| hsa-mir-122-5p | ENTPD4 |
| hsa-mir-122-5p | PHF14 |
| hsa-mir-122-5p | TRAM2 |
| hsa-mir-122-5p | IST1 |
| hsa-mir-122-5p | NCAPD2 |
| hsa-mir-122-5p | AKT3 |
| hsa-mir-122-5p | TRIB1 |
| hsa-mir-122-5p | DCAF7 |
| hsa-mir-122-5p | SPRY2 |
| hsa-mir-122-5p | FSTL3 |
| hsa-mir-122-5p | SPTLC1 |
| hsa-mir-122-5p | SLC19A2 |
| hsa-mir-122-5p | CDC42EP3 |
| hsa-mir-122-5p | YKT6 |
| hsa-mir-122-5p | TOB2 |
| hsa-mir-122-5p | AP3M2 |
| hsa-mir-122-5p | ERP29 |
| hsa-mir-122-5p | NUDT3 |
| hsa-mir-122-5p | AKAP11 |
| hsa-mir-122-5p | MLXIP |
| hsa-mir-122-5p | FOXJ3 |
| hsa-mir-122-5p | MAPRE1 |
| hsa-mir-122-5p | POMZP3 |
| hsa-mir-122-5p | FBXO21 |
| hsa-mir-122-5p | UNC13A |
| hsa-mir-122-5p | NMNAT2 |
| hsa-mir-122-5p | NCDN |
| hsa-mir-122-5p | XPO6 |
| hsa-mir-122-5p | CTDNEP1 |
| hsa-mir-122-5p | GPR161 |
| hsa-mir-122-5p | SLC44A1 |
| hsa-mir-122-5p | POFUT1 |
| hsa-mir-122-5p | DNPEP |
| hsa-mir-122-5p | ORC6 |
| hsa-mir-122-5p | TRIM29 |
| hsa-mir-122-5p | SLC7A11 |
| hsa-mir-122-5p | OSBP2 |
| hsa-mir-122-5p | DSTYK |
| hsa-mir-122-5p | FBXO7 |
| hsa-mir-122-5p | CLIC4 |
| hsa-mir-122-5p | RAI14 |
| hsa-mir-122-5p | PYGO1 |
| hsa-mir-122-5p | WSB1 |
| hsa-mir-122-5p | MYCBP |
| hsa-mir-122-5p | STAU2 |
| hsa-mir-122-5p | FOXP1 |
| hsa-mir-122-5p | ST6GALNAC4 |
| hsa-mir-122-5p | TNRC6A |
| hsa-mir-122-5p | RABGEF1 |
| hsa-mir-122-5p | PHPT1 |
| hsa-mir-122-5p | GLOD4 |
| hsa-mir-122-5p | NT5C3A |
| hsa-mir-122-5p | RAB6B |
| hsa-mir-122-5p | NLGN3 |
| hsa-mir-122-5p | RBM47 |
| hsa-mir-122-5p | GNL3L |
| hsa-mir-122-5p | ZCCHC2 |
| hsa-mir-122-5p | PIGX |
| hsa-mir-122-5p | FAM118A |
| hsa-mir-122-5p | PTCD3 |
| hsa-mir-122-5p | SPTLC3 |
| hsa-mir-122-5p | CHST12 |
| hsa-mir-122-5p | GALNT10 |
| hsa-mir-122-5p | MED29 |
| hsa-mir-122-5p | ANKRD10 |
| hsa-mir-122-5p | TBC1D22B |
| hsa-mir-122-5p | HHAT |
| hsa-mir-122-5p | PRR11 |
| hsa-mir-122-5p | UBAP2 |
| hsa-mir-122-5p | ALG1 |
| hsa-mir-122-5p | PARP11 |
| hsa-mir-122-5p | APMAP |
| hsa-mir-122-5p | CLK4 |
| hsa-mir-122-5p | HECW2 |
| hsa-mir-122-5p | EP400 |
| hsa-mir-122-5p | USP28 |
| hsa-mir-122-5p | ZBTB4 |
| hsa-mir-122-5p | CPNE5 |
| hsa-mir-122-5p | CLSPN |
| hsa-mir-122-5p | SMURF2 |
| hsa-mir-122-5p | EFCAB6 |
| hsa-mir-122-5p | AACS |
| hsa-mir-122-5p | FUNDC2 |
| hsa-mir-122-5p | ATP13A3 |
| hsa-mir-122-5p | SLC52A2 |
| hsa-mir-122-5p | HECTD3 |
| hsa-mir-122-5p | TBL1XR1 |
| hsa-mir-122-5p | MOB3B |
| hsa-mir-122-5p | SLC35E1 |
| hsa-mir-122-5p | RAB11FIP1 |
| hsa-mir-122-5p | ORAI2 |
| hsa-mir-122-5p | ANKRD13C |
| hsa-mir-122-5p | RNF170 |
| hsa-mir-122-5p | KATNAL1 |
| hsa-mir-122-5p | MAGT1 |
| hsa-mir-122-5p | YIPF4 |
| hsa-mir-122-5p | SLC25A33 |
| hsa-mir-122-5p | MCM8 |
| hsa-mir-122-5p | DCTN5 |
| hsa-mir-122-5p | MT4 |
| hsa-mir-122-5p | PPP1R9B |
| hsa-mir-122-5p | LMNB2 |
| hsa-mir-122-5p | LRCH3 |
| hsa-mir-122-5p | CCDC142 |
| hsa-mir-122-5p | ADO |
| hsa-mir-122-5p | POMGNT2 |
| hsa-mir-122-5p | NFATC2IP |
| hsa-mir-122-5p | LRP11 |
| hsa-mir-122-5p | SERAC1 |
| hsa-mir-122-5p | ZNF160 |
| hsa-mir-122-5p | STARD13 |
| hsa-mir-122-5p | G6PC3 |
| hsa-mir-122-5p | EGLN3 |
| hsa-mir-122-5p | GTF3C6 |
| hsa-mir-122-5p | OSBPL10 |
| hsa-mir-122-5p | ZNF618 |
| hsa-mir-122-5p | BATF2 |
| hsa-mir-122-5p | ANKRD9 |
| hsa-mir-122-5p | MRPL52 |
| hsa-mir-122-5p | GPHB5 |
| hsa-mir-122-5p | ZNF573 |
| hsa-mir-122-5p | C1orf122 |
| hsa-mir-122-5p | GNPDA2 |
| hsa-mir-122-5p | ZNF786 |
| hsa-mir-122-5p | MTPN |
| hsa-mir-122-5p | BRI3BP |
| hsa-mir-122-5p | FAM117B |
| hsa-mir-122-5p | TMEM74 |
| hsa-mir-122-5p | DENND2C |
| hsa-mir-122-5p | LCA5 |
| hsa-mir-122-5p | FAM9B |
| hsa-mir-122-5p | DZIP1L |
| hsa-mir-122-5p | ALG14 |
| hsa-mir-122-5p | TRIM65 |
| hsa-mir-122-5p | ZSCAN4 |
| hsa-mir-122-5p | DNAJC18 |
| hsa-mir-122-5p | VMA21 |
| hsa-mir-122-5p | CDY2B |
| hsa-mir-122-5p | SLC25A30 |
| hsa-mir-122-5p | NALCN |
| hsa-mir-122-5p | C14orf39 |
| hsa-mir-122-5p | ZNF233 |
| hsa-mir-122-5p | FAM102A |
| hsa-mir-122-5p | ZNF321P |
| hsa-mir-122-5p | SLC35E2B |
| hsa-mir-122-5p | ARL17B |
| hsa-mir-122-5p | NFYA |
| hsa-mir-204-5p | CYP51A1 |
| hsa-mir-122-5p | CFLAR |
| hsa-mir-122-5p | TFPI |
| hsa-mir-122-5p | NDUFAF7 |
| hsa-mir-122-5p | MTMR7 |
| hsa-mir-122-5p | RBM6 |
| hsa-mir-204-5p | ST7 |
| hsa-mir-204-5p | UPF1 |
| hsa-mir-122-5p | POLR2J |
| hsa-mir-122-5p | FAM214B |
| hsa-mir-122-5p | CROT |
| hsa-mir-122-5p | KMT2E |
| hsa-mir-122-5p | ZNF195 |
| hsa-mir-122-5p | MYCBP2 |
| hsa-mir-122-5p | FBXL3 |
| hsa-mir-204-5p | TNFRSF12A |
| hsa-mir-122-5p | ETV1 |
| hsa-mir-122-5p | PHTF2 |
| hsa-mir-204-5p | PAFAH1B1 |
| hsa-mir-122-5p | MYLIP |
| hsa-mir-122-5p | E2F2 |
| hsa-mir-122-5p | E2F2 |
| hsa-mir-122-5p | JARID2 |
| hsa-mir-122-5p | DLEC1 |
| hsa-mir-122-5p | CYB561 |
| hsa-mir-122-5p | CELSR3 |
| hsa-mir-122-5p | REV3L |
| hsa-mir-122-5p | POMT2 |
| hsa-mir-204-5p | BAZ1B |
| hsa-mir-122-5p | ZNF207 |
| hsa-mir-204-5p | STARD3NL |
| hsa-mir-122-5p | CD9 |
| hsa-mir-122-5p | IFFO1 |
| hsa-mir-122-5p | NISCH |
| hsa-mir-204-5p | CD4 |
| hsa-mir-122-5p | SCMH1 |
| hsa-mir-122-5p | MRC2 |
| hsa-mir-122-5p | AKAP8L |
| hsa-mir-122-5p | PIK3C2A |
| hsa-mir-204-5p | ANLN |
| hsa-mir-122-5p | DCN |
| hsa-mir-122-5p | ERCC1 |
| hsa-mir-122-5p | CLK1 |
| hsa-mir-122-5p | DNASE1L1 |
| hsa-mir-122-5p | CAPN1 |
| hsa-mir-122-5p | ACPP |
| hsa-mir-122-5p | MDH1 |
| hsa-mir-122-5p | ISL1 |
| hsa-mir-122-5p | RUFY3 |
| hsa-mir-122-5p | CXorf56 |
| hsa-mir-122-5p | TTC27 |
| hsa-mir-204-5p | SPAST |
| hsa-mir-204-5p | NRXN3 |
| hsa-mir-204-5p | OSBPL5 |
| hsa-mir-122-5p | CPS1 |
| hsa-mir-204-5p | RB1CC1 |
| hsa-mir-204-5p | AKAP11 |
| hsa-mir-122-5p | ABCC2 |
| hsa-mir-122-5p | PHF20 |
| hsa-mir-204-5p | PHF20 |
| hsa-mir-204-5p | NR1H3 |
| hsa-mir-122-5p | CD44 |
| hsa-mir-204-5p | SLAMF7 |
| hsa-mir-122-5p | BTN3A1 |
| hsa-mir-122-5p | VEZT |
| hsa-mir-204-5p | VEZT |
| hsa-mir-204-5p | BRD9 |
| hsa-mir-122-5p | BCLAF1 |
| hsa-mir-122-5p | ANK1 |
| hsa-mir-122-5p | ZCCHC8 |
| hsa-mir-122-5p | UBA6 |
| hsa-mir-122-5p | PEX3 |
| hsa-mir-204-5p | TIMP2 |
| hsa-mir-122-5p | CUL3 |
| hsa-mir-122-5p | BOD1L1 |
| hsa-mir-122-5p | TLL1 |
| hsa-mir-122-5p | TRIO |
| hsa-mir-122-5p | CDH1 |
| hsa-mir-122-5p | ZFYVE16 |
| hsa-mir-122-5p | PARP3 |
| hsa-mir-122-5p | MED17 |
| hsa-mir-122-5p | DCUN1D1 |
| hsa-mir-204-5p | CTNNA1 |
| hsa-mir-122-5p | PHKA2 |
| hsa-mir-122-5p | EPHA3 |
| hsa-mir-122-5p | DSG2 |
| hsa-mir-122-5p | OFD1 |
| hsa-mir-122-5p | GPM6B |
| hsa-mir-122-5p | TPR |
| hsa-mir-204-5p | MAP4 |
| hsa-mir-122-5p | GOPC |
| hsa-mir-122-5p | MRPS10 |
| hsa-mir-122-5p | VPS13D |
| hsa-mir-122-5p | ADAMTS6 |
| hsa-mir-122-5p | LTBP1 |
| hsa-mir-122-5p | NFE2L3 |
| hsa-mir-122-5p | LIMA1 |
| hsa-mir-204-5p | LIMA1 |
| hsa-mir-122-5p | LAMC3 |
| hsa-mir-204-5p | HERPUD1 |
| hsa-mir-204-5p | RAD51 |
| hsa-mir-204-5p | PIK3CB |
| hsa-mir-122-5p | MPHOSPH9 |
| hsa-mir-122-5p | SIKE1 |
| hsa-mir-122-5p | TTC17 |
| hsa-mir-122-5p | FOXN3 |
| hsa-mir-122-5p | THRAP3 |
| hsa-mir-122-5p | SDCCAG8 |
| hsa-mir-122-5p | SDCCAG8 |
| hsa-mir-122-5p | KIF1B |
| hsa-mir-122-5p | TBC1D22A |
| hsa-mir-122-5p | SYNE2 |
| hsa-mir-122-5p | PLEKHH1 |
| hsa-mir-122-5p | CHRDL2 |
| hsa-mir-122-5p | SZRD1 |
| hsa-mir-204-5p | TAB2 |
| hsa-mir-204-5p | TAB2 |
| hsa-mir-204-5p | EIF2AK2 |
| hsa-mir-122-5p | KMT2C |
| hsa-mir-122-5p | RC3H2 |
| hsa-mir-204-5p | DCBLD2 |
| hsa-mir-122-5p | SOAT1 |
| hsa-mir-122-5p | TMCC3 |
| hsa-mir-204-5p | SEC61A1 |
| hsa-mir-204-5p | ATP2B4 |
| hsa-mir-122-5p | ZC3H11A |
| hsa-mir-204-5p | NDC1 |
| hsa-mir-122-5p | WNK1 |
| hsa-mir-122-5p | CCAR1 |
| hsa-mir-122-5p | QSER1 |
| hsa-mir-204-5p | QSER1 |
| hsa-mir-204-5p | LZTS1 |
| hsa-mir-204-5p | NCKAP1 |
| hsa-mir-122-5p | ELMO2 |
| hsa-mir-122-5p | VMP1 |
| hsa-mir-122-5p | AHRR |
| hsa-mir-122-5p | LIMCH1 |
| hsa-mir-122-5p | HIPK2 |
| hsa-mir-122-5p | LPAR2 |
| hsa-mir-122-5p | SUGP2 |
| hsa-mir-122-5p | EYA2 |
| hsa-mir-122-5p | ANKS1A |
| hsa-mir-122-5p | PKN2 |
| hsa-mir-122-5p | SPEN |
| hsa-mir-122-5p | MYLK |
| hsa-mir-204-5p | FAM107B |
| hsa-mir-122-5p | CDK13 |
| hsa-mir-122-5p | MTHFD2 |
| hsa-mir-122-5p | ASPM |
| hsa-mir-204-5p | KLF6 |
| hsa-mir-122-5p | EVI5 |
| hsa-mir-122-5p | CBFB |
| hsa-mir-122-5p | HEATR6 |
| hsa-mir-204-5p | ACSL4 |
| hsa-mir-122-5p | PSME4 |
| hsa-mir-204-5p | NUCKS1 |
| hsa-mir-122-5p | RORA |
| hsa-mir-122-5p | NUCB2 |
| hsa-mir-122-5p | TMEM260 |
| hsa-mir-204-5p | EXOC5 |
| hsa-mir-122-5p | MNT |
| hsa-mir-122-5p | ST6GALNAC1 |
| hsa-mir-122-5p | OSBPL3 |
| hsa-mir-122-5p | RAD18 |
| hsa-mir-122-5p | ATP2B1 |
| hsa-mir-122-5p | MAP4K4 |
| hsa-mir-204-5p | MGAT4A |
| hsa-mir-122-5p | MBD3 |
| hsa-mir-122-5p | RDH11 |
| hsa-mir-204-5p | ZFYVE26 |
| hsa-mir-122-5p | LNX1 |
| hsa-mir-122-5p | AFF4 |
| hsa-mir-122-5p | NDE1 |
| hsa-mir-122-5p | NDE1 |
| hsa-mir-122-5p | ALPK1 |
| hsa-mir-122-5p | ST6GAL1 |
| hsa-mir-204-5p | PICALM |
| hsa-mir-122-5p | CLNS1A |
| hsa-mir-122-5p | CA12 |
| hsa-mir-204-5p | NTN4 |
| hsa-mir-122-5p | NUAK1 |
| hsa-mir-204-5p | DPP8 |
| hsa-mir-122-5p | ZNF532 |
| hsa-mir-122-5p | ANO8 |
| hsa-mir-122-5p | GTSE1 |
| hsa-mir-122-5p | SEMA3C |
| hsa-mir-122-5p | ZNF638 |
| hsa-mir-122-5p | SLC25A40 |
| hsa-mir-122-5p | RASAL2 |
| hsa-mir-122-5p | MARK3 |
| hsa-mir-204-5p | SLC25A3 |
| hsa-mir-122-5p | FNDC3B |
| hsa-mir-204-5p | FNDC3B |
| hsa-mir-122-5p | FRYL |
| hsa-mir-122-5p | ACTB |
| hsa-mir-122-5p | PLD1 |
| hsa-mir-122-5p | DLG1 |
| hsa-mir-122-5p | MKRN2 |
| hsa-mir-204-5p | BAZ2A |
| hsa-mir-122-5p | PLXNA2 |
| hsa-mir-122-5p | ANKRD13A |
| hsa-mir-122-5p | ACACB |
| hsa-mir-122-5p | TRAF4 |
| hsa-mir-204-5p | TOP2B |
| hsa-mir-204-5p | IL4R |
| hsa-mir-122-5p | IL4R |
| hsa-mir-122-5p | USP33 |
| hsa-mir-122-5p | SNRPA |
| hsa-mir-122-5p | FGFR1 |
| hsa-mir-122-5p | MAP2 |
| hsa-mir-204-5p | MAP2 |
| hsa-mir-122-5p | PIAS2 |
| hsa-mir-122-5p | PIK3C3 |
| hsa-mir-122-5p | CBFA2T2 |
| hsa-mir-122-5p | TP73 |
| hsa-mir-122-5p | XRCC5 |
| hsa-mir-122-5p | MKNK1 |
| hsa-mir-122-5p | TNS1 |
| hsa-mir-122-5p | RAPGEF3 |
| hsa-mir-204-5p | SENP1 |
| hsa-mir-122-5p | FDFT1 |
| hsa-mir-122-5p | FDFT1 |
| hsa-mir-122-5p | OPHN1 |
| hsa-mir-204-5p | DNM2 |
| hsa-mir-122-5p | RIMS1 |
| hsa-mir-122-5p | RIF1 |
| hsa-mir-204-5p | RIF1 |
| hsa-mir-122-5p | CNOT4 |
| hsa-mir-122-5p | DLGAP4 |
| hsa-mir-122-5p | CFHR2 |
| hsa-mir-122-5p | RSBN1 |
| hsa-mir-122-5p | OSTM1 |
| hsa-mir-122-5p | ARG2 |
| hsa-mir-122-5p | ATP8B1 |
| hsa-mir-122-5p | ME2 |
| hsa-mir-122-5p | CCNT2 |
| hsa-mir-204-5p | EPB41L3 |
| hsa-mir-122-5p | COBLL1 |
| hsa-mir-204-5p | GSK3B |
| hsa-mir-204-5p | XPO1 |
| hsa-mir-122-5p | TNPO1 |
| hsa-mir-122-5p | YTHDC1 |
| hsa-mir-122-5p | YTHDC1 |
| hsa-mir-204-5p | YTHDC1 |
| hsa-mir-204-5p | YTHDC1 |
| hsa-mir-122-5p | HAL |
| hsa-mir-122-5p | APLP2 |
| hsa-mir-122-5p | EIF3I |
| hsa-mir-122-5p | NCOA1 |
| hsa-mir-122-5p | ATRX |
| hsa-mir-122-5p | MECOM |
| hsa-mir-204-5p | SEH1L |
| hsa-mir-122-5p | SEH1L |
| hsa-mir-122-5p | WDR47 |
| hsa-mir-204-5p | SLC25A24 |
| hsa-mir-204-5p | SLC25A24 |
| hsa-mir-122-5p | ABCB1 |
| hsa-mir-122-5p | ZNF213 |
| hsa-mir-122-5p | CTTN |
| hsa-mir-122-5p | CTTN |
| hsa-mir-204-5p | DDHD2 |
| hsa-mir-122-5p | SNX10 |
| hsa-mir-122-5p | RBM22 |
| hsa-mir-122-5p | ZFAND6 |
| hsa-mir-122-5p | PPEF1 |
| hsa-mir-122-5p | HUWE1 |
| hsa-mir-204-5p | FTL |
| hsa-mir-122-5p | PIGS |
| hsa-mir-122-5p | SF3B2 |
| hsa-mir-122-5p | KLHL42 |
| hsa-mir-122-5p | GNAS |
| hsa-mir-204-5p | GNAS |
| hsa-mir-122-5p | CNOT3 |
| hsa-mir-122-5p | CNOT3 |
| hsa-mir-122-5p | CNOT3 |
| hsa-mir-122-5p | CNOT3 |
| hsa-mir-122-5p | CNOT3 |
| hsa-mir-122-5p | CNOT3 |
| hsa-mir-122-5p | CNOT3 |
| hsa-mir-122-5p | CNOT3 |
| hsa-mir-122-5p | CNOT3 |
| hsa-mir-122-5p | CNOT3 |
| hsa-mir-122-5p | DOCK9 |
| hsa-mir-122-5p | TGDS |
| hsa-mir-122-5p | COQ9 |
| hsa-mir-204-5p | MAVS |
| hsa-mir-122-5p | KIZ |
| hsa-mir-122-5p | ANAPC5 |
| hsa-mir-122-5p | SIRT4 |
| hsa-mir-122-5p | FUS |
| hsa-mir-122-5p | RBM41 |
| hsa-mir-122-5p | OTUB2 |
| hsa-mir-122-5p | OTUB2 |
| hsa-mir-204-5p | NECAP1 |
| hsa-mir-122-5p | NECAP1 |
| hsa-mir-204-5p | DHX32 |
| hsa-mir-122-5p | GPATCH2L |
| hsa-mir-122-5p | IRAK3 |
| hsa-mir-122-5p | SPG21 |
| hsa-mir-122-5p | RAB11FIP3 |
| hsa-mir-122-5p | RAB11FIP3 |
| hsa-mir-122-5p | GLG1 |
| hsa-mir-122-5p | RBM27 |
| hsa-mir-122-5p | RBM27 |
| hsa-mir-122-5p | OSBPL8 |
| hsa-mir-122-5p | LAMB1 |
| hsa-mir-122-5p | CDV3 |
| hsa-mir-204-5p | ZFHX4 |
| hsa-mir-122-5p | SCFD1 |
| hsa-mir-122-5p | HNRNPC |
| hsa-mir-122-5p | GPATCH2 |
| hsa-mir-122-5p | SEC22C |
| hsa-mir-204-5p | CDC6 |
| hsa-mir-122-5p | CBX5 |
| hsa-mir-204-5p | CBX5 |
| hsa-mir-204-5p | SUCO |
| hsa-mir-122-5p | TMEM38B |
| hsa-mir-122-5p | PTGS1 |
| hsa-mir-122-5p | CWF19L1 |
| hsa-mir-122-5p | FKBP5 |
| hsa-mir-122-5p | EFHC1 |
| hsa-mir-122-5p | JAK2 |
| hsa-mir-204-5p | SCD |
| hsa-mir-122-5p | RAB18 |
| hsa-mir-122-5p | PRTFDC1 |
| hsa-mir-122-5p | HNRNPM |
| hsa-mir-122-5p | MKNK2 |
| hsa-mir-122-5p | SLC35E4 |
| hsa-mir-122-5p | MFNG |
| hsa-mir-122-5p | GGA1 |
| hsa-mir-122-5p | HIRA |
| hsa-mir-122-5p | HPS4 |
| hsa-mir-122-5p | DDX17 |
| hsa-mir-122-5p | CBY1 |
| hsa-mir-122-5p | RHBDD3 |
| hsa-mir-204-5p | HMGXB4 |
| hsa-mir-122-5p | CHKB |
| hsa-mir-122-5p | MTMR3 |
| hsa-mir-204-5p | MTMR3 |
| hsa-mir-122-5p | PNPLA3 |
| hsa-mir-122-5p | TNRC6B |
| hsa-mir-204-5p | TNRC6B |
| hsa-mir-122-5p | IFT27 |
| hsa-mir-122-5p | ZC3H7B |
| hsa-mir-122-5p | BRD1 |
| hsa-mir-122-5p | SOS2 |
| hsa-mir-204-5p | ALKBH1 |
| hsa-mir-122-5p | SNW1 |
| hsa-mir-122-5p | DICER1 |
| hsa-mir-204-5p | DICER1 |
| hsa-mir-122-5p | PAPLN |
| hsa-mir-204-5p | RPS6KA5 |
| hsa-mir-122-5p | NFKBIA |
| hsa-mir-122-5p | MYBL2 |
| hsa-mir-122-5p | NDRG3 |
| hsa-mir-122-5p | PABPC1L |
| hsa-mir-122-5p | STK4 |
| hsa-mir-122-5p | PLCB4 |
| hsa-mir-122-5p | TM9SF4 |
| hsa-mir-204-5p | KIF3B |
| hsa-mir-122-5p | NOP56 |
| hsa-mir-204-5p | MAPRE1 |
| hsa-mir-122-5p | TTI1 |
| hsa-mir-122-5p | E2F1 |
| hsa-mir-122-5p | TNNC2 |
| hsa-mir-122-5p | SMCHD1 |
| hsa-mir-122-5p | RIOK3 |
| hsa-mir-122-5p | MID1 |
| hsa-mir-122-5p | MOSPD1 |
| hsa-mir-122-5p | CCDC22 |
| hsa-mir-204-5p | PLP2 |
| hsa-mir-204-5p | ELF4 |
| hsa-mir-204-5p | RBBP7 |
| hsa-mir-122-5p | GABRE |
| hsa-mir-122-5p | SYTL4 |
| hsa-mir-122-5p | CENPI |
| hsa-mir-122-5p | FNDC3A |
| hsa-mir-122-5p | STK24 |
| hsa-mir-122-5p | ARHGEF7 |
| hsa-mir-122-5p | SUPT20H |
| hsa-mir-122-5p | MRPS31 |
| hsa-mir-122-5p | FLT1 |
| hsa-mir-122-5p | VWA8 |
| hsa-mir-122-5p | MSLN |
| hsa-mir-204-5p | MGRN1 |
| hsa-mir-122-5p | NFAT5 |
| hsa-mir-204-5p | NFAT5 |
| hsa-mir-122-5p | LONP2 |
| hsa-mir-122-5p | ARL2BP |
| hsa-mir-122-5p | DHODH |
| hsa-mir-122-5p | PSMD7 |
| hsa-mir-122-5p | SETD6 |
| hsa-mir-122-5p | TAF1C |
| hsa-mir-204-5p | USP10 |
| hsa-mir-122-5p | ZNF500 |
| hsa-mir-122-5p | MTHFSD |
| hsa-mir-122-5p | UBE2I |
| hsa-mir-204-5p | UBE2I |
| hsa-mir-122-5p | PIEZO1 |
| hsa-mir-122-5p | GSPT1 |
| hsa-mir-122-5p | USP31 |
| hsa-mir-122-5p | TOX3 |
| hsa-mir-122-5p | TMC5 |
| hsa-mir-122-5p | KNOP1 |
| hsa-mir-122-5p | TMEM87A |
| hsa-mir-122-5p | CEP152 |
| hsa-mir-122-5p | DTWD1 |
| hsa-mir-122-5p | CSPP1 |
| hsa-mir-122-5p | FZD3 |
| hsa-mir-204-5p | NBN |
| hsa-mir-122-5p | PLAT |
| hsa-mir-122-5p | TRPS1 |
| hsa-mir-204-5p | EEF1D |
| hsa-mir-204-5p | EEF1D |
| hsa-mir-122-5p | INTS10 |
| hsa-mir-122-5p | MTMR9 |
| hsa-mir-122-5p | MTMR9 |
| hsa-mir-122-5p | TNFRSF10A |
| hsa-mir-122-5p | ERICH1 |
| hsa-mir-122-5p | ERICH1 |
| hsa-mir-204-5p | TUSC3 |
| hsa-mir-122-5p | ARHGEF10 |
| hsa-mir-122-5p | ARHGEF10 |
| hsa-mir-122-5p | KLHDC4 |
| hsa-mir-122-5p | MCM4 |
| hsa-mir-122-5p | ASAH1 |
| hsa-mir-122-5p | MAN2B1 |
| hsa-mir-122-5p | HNRNPL |
| hsa-mir-122-5p | HNRNPL |
| hsa-mir-204-5p | HNRNPL |
| hsa-mir-204-5p | HNRNPL |
| hsa-mir-122-5p | DOT1L |
| hsa-mir-204-5p | OAZ1 |
| hsa-mir-122-5p | MED26 |
| hsa-mir-122-5p | CCNE1 |
| hsa-mir-122-5p | URI1 |
| hsa-mir-122-5p | BCAT2 |
| hsa-mir-122-5p | MIER2 |
| hsa-mir-122-5p | TNPO2 |
| hsa-mir-204-5p | TNPO2 |
| hsa-mir-122-5p | CACNG7 |
| hsa-mir-122-5p | PDE4C |
| hsa-mir-122-5p | SCN1B |
| hsa-mir-204-5p | GSK3A |
| hsa-mir-122-5p | ZNF85 |
| hsa-mir-122-5p | ZNF85 |
| hsa-mir-122-5p | AVL9 |
| hsa-mir-122-5p | CFAP69 |
| hsa-mir-122-5p | PIK3CG |
| hsa-mir-122-5p | PON2 |
| hsa-mir-122-5p | ITGB8 |
| hsa-mir-204-5p | MTPN |
| hsa-mir-122-5p | SSBP1 |
| hsa-mir-122-5p | SSBP1 |
| hsa-mir-122-5p | GRB10 |
| hsa-mir-122-5p | ABHD11 |
| hsa-mir-122-5p | PTCD1 |
| hsa-mir-122-5p | ZKSCAN1 |
| hsa-mir-122-5p | EIF3B |
| hsa-mir-204-5p | EIF3B |
| hsa-mir-122-5p | USP42 |
| hsa-mir-122-5p | COA1 |
| hsa-mir-122-5p | URGCP |
| hsa-mir-122-5p | PRKAG2 |
| hsa-mir-122-5p | LIMK1 |
| hsa-mir-122-5p | TGFBR1 |
| hsa-mir-122-5p | SEC61B |
| hsa-mir-204-5p | PTGR1 |
| hsa-mir-122-5p | RLN1 |
| hsa-mir-122-5p | RLN1 |
| hsa-mir-204-5p | TBC1D13 |
| hsa-mir-204-5p | CCNJ |
| hsa-mir-122-5p | EIF3A |
| hsa-mir-122-5p | DDX50 |
| hsa-mir-122-5p | LZTS2 |
| hsa-mir-122-5p | SFXN3 |
| hsa-mir-122-5p | TNKS2 |
| hsa-mir-204-5p | TNKS2 |
| hsa-mir-122-5p | ARHGAP21 |
| hsa-mir-122-5p | CPEB3 |
| hsa-mir-122-5p | SMC3 |
| hsa-mir-204-5p | SMC3 |
| hsa-mir-204-5p | SHOC2 |
| hsa-mir-204-5p | PPIF |
| hsa-mir-204-5p | NUFIP2 |
| hsa-mir-122-5p | GIT1 |
| hsa-mir-204-5p | GOSR2 |
| hsa-mir-122-5p | RPS6KB1 |
| hsa-mir-122-5p | HOXB6 |
| hsa-mir-122-5p | RAI1 |
| hsa-mir-122-5p | MED31 |
| hsa-mir-122-5p | AKAP10 |
| hsa-mir-122-5p | DHX58 |
| hsa-mir-122-5p | COL1A1 |
| hsa-mir-122-5p | ABCC3 |
| hsa-mir-122-5p | LUC7L3 |
| hsa-mir-122-5p | PRKAR1A |
| hsa-mir-122-5p | MMD |
| hsa-mir-204-5p | PMP22 |
| hsa-mir-122-5p | OCIAD1 |
| hsa-mir-122-5p | AREG |
| hsa-mir-122-5p | MFSD10 |
| hsa-mir-122-5p | GLRB |
| hsa-mir-122-5p | RAPGEF2 |
| hsa-mir-204-5p | RAPGEF2 |
| hsa-mir-122-5p | KLF3 |
| hsa-mir-204-5p | KLF3 |
| hsa-mir-204-5p | CCND1 |
| hsa-mir-122-5p | FOLR1 |
| hsa-mir-122-5p | CEP164 |
| hsa-mir-204-5p | DDX6 |
| hsa-mir-122-5p | CBL |
| hsa-mir-122-5p | PTPMT1 |
| hsa-mir-122-5p | PTPMT1 |
| hsa-mir-122-5p | SOX6 |
| hsa-mir-122-5p | NDUFS8 |
| hsa-mir-122-5p | EXPH5 |
| hsa-mir-204-5p | CORO1C |
| hsa-mir-204-5p | MLEC |
| hsa-mir-122-5p | CAMKK2 |
| hsa-mir-122-5p | BCL7A |
| hsa-mir-122-5p | BCL7A |
| hsa-mir-122-5p | CDKN1B |
| hsa-mir-122-5p | NAA25 |
| hsa-mir-122-5p | SCNN1A |
| hsa-mir-204-5p | SLC38A1 |
| hsa-mir-204-5p | VDR |
| hsa-mir-122-5p | IL26 |
| hsa-mir-204-5p | CPSF6 |
| hsa-mir-204-5p | NOP2 |
| hsa-mir-122-5p | CHD4 |
| hsa-mir-122-5p | UHRF1BP1L |
| hsa-mir-204-5p | TPI1 |
| hsa-mir-122-5p | LPCAT3 |
| hsa-mir-122-5p | NT5DC3 |
| hsa-mir-122-5p | SUDS3 |
| hsa-mir-122-5p | FGFR1OP2 |
| hsa-mir-122-5p | RNGTT |
| hsa-mir-204-5p | RNGTT |
| hsa-mir-122-5p | NCOA7 |
| hsa-mir-122-5p | FBXO9 |
| hsa-mir-122-5p | MDN1 |
| hsa-mir-122-5p | MDN1 |
| hsa-mir-122-5p | ZNF451 |
| hsa-mir-204-5p | ZNF451 |
| hsa-mir-122-5p | BAG2 |
| hsa-mir-122-5p | E2F3 |
| hsa-mir-122-5p | B3GAT2 |
| hsa-mir-122-5p | SOBP |
| hsa-mir-122-5p | TRIM38 |
| hsa-mir-122-5p | PHF1 |
| hsa-mir-122-5p | PHF1 |
| hsa-mir-122-5p | PHF1 |
| hsa-mir-122-5p | QKI |
| hsa-mir-204-5p | QKI |
| hsa-mir-204-5p | TMEM30A |
| hsa-mir-122-5p | ENPP5 |
| hsa-mir-204-5p | HMGCS1 |
| hsa-mir-122-5p | BRD8 |
| hsa-mir-122-5p | LMNB1 |
| hsa-mir-204-5p | ARRDC3 |
| hsa-mir-204-5p | SUB1 |
| hsa-mir-122-5p | LNPEP |
| hsa-mir-122-5p | TRAPPC13 |
| hsa-mir-204-5p | CSNK1A1 |
| hsa-mir-122-5p | ATP6V0E1 |
| hsa-mir-122-5p | CPEB4 |
| hsa-mir-122-5p | ZNF346 |
| hsa-mir-122-5p | SMC4 |
| hsa-mir-122-5p | BCL6 |
| hsa-mir-122-5p | ARMC8 |
| hsa-mir-122-5p | SLC25A36 |
| hsa-mir-122-5p | COL7A1 |
| hsa-mir-122-5p | PRKAR2A |
| hsa-mir-122-5p | USP4 |
| hsa-mir-204-5p | USP4 |
| hsa-mir-122-5p | TUSC2 |
| hsa-mir-204-5p | RPL24 |
| hsa-mir-122-5p | FXR1 |
| hsa-mir-122-5p | BBX |
| hsa-mir-204-5p | GBE1 |
| hsa-mir-122-5p | PLXNA1 |
| hsa-mir-122-5p | PLSCR4 |
| hsa-mir-122-5p | MAPKAPK3 |
| hsa-mir-204-5p | WDR48 |
| hsa-mir-122-5p | ZBTB47 |
| hsa-mir-122-5p | NKTR |
| hsa-mir-122-5p | NEK4 |
| hsa-mir-122-5p | INO80D |
| hsa-mir-122-5p | INO80D |
| hsa-mir-204-5p | INO80D |
| hsa-mir-204-5p | INO80D |
| hsa-mir-204-5p | PIKFYVE |
| hsa-mir-204-5p | NCL |
| hsa-mir-204-5p | ACTR3 |
| hsa-mir-204-5p | GPD2 |
| hsa-mir-122-5p | GTF3C2 |
| hsa-mir-204-5p | SNX17 |
| hsa-mir-122-5p | IFIH1 |
| hsa-mir-122-5p | SPTBN1 |
| hsa-mir-204-5p | RTN4 |
| hsa-mir-122-5p | USP34 |
| hsa-mir-122-5p | IL1R1 |
| hsa-mir-122-5p | HDLBP |
| hsa-mir-122-5p | ID2 |
| hsa-mir-122-5p | PLEKHB2 |
| hsa-mir-122-5p | PRKD3 |
| hsa-mir-204-5p | PRKD3 |
| hsa-mir-122-5p | SRSF7 |
| hsa-mir-122-5p | MSH6 |
| hsa-mir-122-5p | FARSB |
| hsa-mir-122-5p | ALMS1 |
| hsa-mir-204-5p | DHCR24 |
| hsa-mir-122-5p | CACYBP |
| hsa-mir-122-5p | RALGPS2 |
| hsa-mir-204-5p | RALGPS2 |
| hsa-mir-122-5p | RPL22 |
| hsa-mir-122-5p | SRSF4 |
| hsa-mir-122-5p | ASH1L |
| hsa-mir-204-5p | ASH1L |
| hsa-mir-122-5p | SFPQ |
| hsa-mir-122-5p | RHOU |
| hsa-mir-122-5p | RHOU |
| hsa-mir-122-5p | GON4L |
| hsa-mir-204-5p | GON4L |
| hsa-mir-204-5p | MEF2D |
| hsa-mir-122-5p | PRG4 |
| hsa-mir-204-5p | WLS |
| hsa-mir-122-5p | SRSF11 |
| hsa-mir-204-5p | SRSF11 |
| hsa-mir-204-5p | C1orf109 |
| hsa-mir-204-5p | KDM5B |
| hsa-mir-204-5p | ZNHIT6 |
| hsa-mir-204-5p | RBBP5 |
| hsa-mir-204-5p | RAB29 |
| hsa-mir-122-5p | APH1A |
| hsa-mir-122-5p | NSUN4 |
| hsa-mir-122-5p | TMED5 |
| hsa-mir-204-5p | CNN3 |
| hsa-mir-122-5p | PRRC2C |
| hsa-mir-204-5p | PRRC2C |
| hsa-mir-122-5p | VAMP4 |
| hsa-mir-122-5p | RSRP1 |
| hsa-mir-204-5p | CENPF |
| hsa-mir-122-5p | KMT2A |
| hsa-mir-122-5p | DDX59 |
| hsa-mir-122-5p | ATF6 |
| hsa-mir-122-5p | B4GALT6 |
| hsa-mir-122-5p | MYB |
| hsa-mir-122-5p | ALDH8A1 |
| hsa-mir-122-5p | SLC16A7 |
| hsa-mir-122-5p | ZNF430 |
| hsa-mir-122-5p | ZNF430 |
| hsa-mir-122-5p | TGIF2 |
| hsa-mir-122-5p | MFSD1 |
| hsa-mir-122-5p | RAB3GAP2 |
| hsa-mir-122-5p | UBN1 |
| hsa-mir-204-5p | CCND2 |
| hsa-mir-204-5p | ELL2 |
| hsa-mir-122-5p | SATB2 |
| hsa-mir-122-5p | TJP2 |
| hsa-mir-204-5p | RAD23B |
| hsa-mir-122-5p | CNTRL |
| hsa-mir-204-5p | FBXW2 |
| hsa-mir-122-5p | PPP6C |
| hsa-mir-122-5p | HSDL2 |
| hsa-mir-122-5p | DENND1A |
| hsa-mir-122-5p | KDSR |
| hsa-mir-122-5p | ONECUT2 |
| hsa-mir-122-5p | YLPM1 |
| hsa-mir-122-5p | PGF |
| hsa-mir-204-5p | NEK9 |
| hsa-mir-122-5p | RBM25 |
| hsa-mir-122-5p | KLHL29 |
| hsa-mir-122-5p | YPEL5 |
| hsa-mir-122-5p | FAM98A |
| hsa-mir-204-5p | YIPF4 |
| hsa-mir-122-5p | OGFRL1 |
| hsa-mir-122-5p | IFIT3 |
| hsa-mir-122-5p | IFIT2 |
| hsa-mir-204-5p | HELLS |
| hsa-mir-122-5p | WDR11 |
| hsa-mir-122-5p | KANSL1 |
| hsa-mir-122-5p | KANSL1 |
| hsa-mir-122-5p | KANSL1 |
| hsa-mir-122-5p | HOXB3 |
| hsa-mir-204-5p | HOXB3 |
| hsa-mir-122-5p | GRIA2 |
| hsa-mir-204-5p | TCP1 |
| hsa-mir-122-5p | KIAA1217 |
| hsa-mir-122-5p | TNFSF11 |
| hsa-mir-122-5p | MTRF1 |
| hsa-mir-122-5p | PROSER1 |
| hsa-mir-122-5p | KBTBD7 |
| hsa-mir-204-5p | EGR1 |
| hsa-mir-122-5p | NR2C1 |
| hsa-mir-122-5p | GLT8D2 |
| hsa-mir-122-5p | DUSP4 |
| hsa-mir-122-5p | TNFRSF10B |
| hsa-mir-204-5p | RNF170 |
| hsa-mir-122-5p | AKAP1 |
| hsa-mir-204-5p | SEC22A |
| hsa-mir-204-5p | KIF18A |
| hsa-mir-122-5p | PILRB |
| hsa-mir-122-5p | ZMYM2 |
| hsa-mir-204-5p | KHDRBS1 |
| hsa-mir-122-5p | CXCR4 |
| hsa-mir-122-5p | OCRL |
| hsa-mir-122-5p | RBBP6 |
| hsa-mir-122-5p | RPL5 |
| hsa-mir-122-5p | ODF2L |
| hsa-mir-122-5p | CBX3 |
| hsa-mir-122-5p | KIAA1549 |
| hsa-mir-122-5p | NUDT10 |
| hsa-mir-122-5p | SLC25A16 |
| hsa-mir-122-5p | MED13L |
| hsa-mir-204-5p | ITPR2 |
| hsa-mir-122-5p | CCDC91 |
| hsa-mir-122-5p | NLN |
| hsa-mir-122-5p | ARHGAP9 |
| hsa-mir-204-5p | PFDN5 |
| hsa-mir-122-5p | HOXC13 |
| hsa-mir-122-5p | IKZF4 |
| hsa-mir-204-5p | AMD1 |
| hsa-mir-122-5p | MORF4L2 |
| hsa-mir-122-5p | KCNJ2 |
| hsa-mir-122-5p | RAP2C |
| hsa-mir-204-5p | RAP2C |
| hsa-mir-204-5p | ACSL3 |
| hsa-mir-122-5p | GCNT7 |
| hsa-mir-122-5p | VAPB |
| hsa-mir-122-5p | PLCG1 |
| hsa-mir-122-5p | SRSF6 |
| hsa-mir-122-5p | ZNFX1 |
| hsa-mir-122-5p | TMEM189-UBE2V1 |
| hsa-mir-204-5p | VAMP7 |
| hsa-mir-204-5p | USP9X |
| hsa-mir-122-5p | SERPINB6 |
| hsa-mir-122-5p | ABCC10 |
| hsa-mir-122-5p | RPS10 |
| hsa-mir-122-5p | SOX4 |
| hsa-mir-122-5p | RREB1 |
| hsa-mir-204-5p | RIOK1 |
| hsa-mir-122-5p | ATXN1 |
| hsa-mir-122-5p | NUP153 |
| hsa-mir-204-5p | DEK |
| hsa-mir-204-5p | AHNAK |
| hsa-mir-204-5p | CNOT1 |
| hsa-mir-122-5p | TMTC4 |
| hsa-mir-122-5p | GGA3 |
| hsa-mir-122-5p | NUP85 |
| hsa-mir-122-5p | SLC25A19 |
| hsa-mir-122-5p | PPDPF |
| hsa-mir-122-5p | POLR1B |
| hsa-mir-122-5p | THOC2 |
| hsa-mir-122-5p | RPL23 |
| hsa-mir-122-5p | FOSB |
| hsa-mir-204-5p | OPA3 |
| hsa-mir-122-5p | EML2 |
| hsa-mir-122-5p | SYMPK |
| hsa-mir-204-5p | RBCK1 |
| hsa-mir-204-5p | STK35 |
| hsa-mir-122-5p | AP5S1 |
| hsa-mir-122-5p | RRBP1 |
| hsa-mir-122-5p | ZNF133 |
| hsa-mir-122-5p | FLRT3 |
| hsa-mir-122-5p | TBC1D20 |
| hsa-mir-122-5p | ST3GAL3 |
| hsa-mir-122-5p | CAPNS1 |
| hsa-mir-122-5p | THRA |
| hsa-mir-122-5p | KTN1 |
| hsa-mir-122-5p | TIMM8A |
| hsa-mir-122-5p | CANX |
| hsa-mir-122-5p | CANX |
| hsa-mir-204-5p | CANX |
| hsa-mir-204-5p | CANX |
| hsa-mir-122-5p | IPPK |
| hsa-mir-122-5p | EMC1 |
| hsa-mir-122-5p | PLA2G5 |
| hsa-mir-122-5p | UBR4 |
| hsa-mir-204-5p | UBR4 |
| hsa-mir-122-5p | CHTF18 |
| hsa-mir-122-5p | GNG13 |
| hsa-mir-122-5p | MACF1 |
| hsa-mir-122-5p | SMARCA4 |
| hsa-mir-122-5p | KDM4B |
| hsa-mir-204-5p | GNG11 |
| hsa-mir-122-5p | PTPN12 |
| hsa-mir-122-5p | PEX1 |
| hsa-mir-122-5p | LRFN1 |
| hsa-mir-122-5p | SRD5A3 |
| hsa-mir-122-5p | GNAZ |
| hsa-mir-122-5p | KRT17 |
| hsa-mir-122-5p | FOXP2 |
| hsa-mir-122-5p | STRIP2 |
| hsa-mir-122-5p | DNAJB9 |
| hsa-mir-122-5p | NDUFA5 |
| hsa-mir-122-5p | GAD1 |
| hsa-mir-122-5p | SNRPN |
| hsa-mir-122-5p | MYO5C |
| hsa-mir-204-5p | TMOD2 |
| hsa-mir-122-5p | TTBK2 |
| hsa-mir-122-5p | VPS13C |
| hsa-mir-204-5p | CALML4 |
| hsa-mir-204-5p | COPB1 |
| hsa-mir-122-5p | PALLD |
| hsa-mir-122-5p | RPAIN |
| hsa-mir-122-5p | AIPL1 |
| hsa-mir-122-5p | PHF20L1 |
| hsa-mir-122-5p | ILF3 |
| hsa-mir-122-5p | MTUS1 |
| hsa-mir-204-5p | HEATR5A |
| hsa-mir-122-5p | MIS18BP1 |
| hsa-mir-122-5p | SEC14L1 |
| hsa-mir-122-5p | RHBDF2 |
| hsa-mir-122-5p | PHF10 |
| hsa-mir-204-5p | PHF10 |
| hsa-mir-204-5p | SAT1 |
| hsa-mir-122-5p | SAT1 |
| hsa-mir-204-5p | LDLR |
| hsa-mir-204-5p | LRCH2 |
| hsa-mir-122-5p | PXDN |
| hsa-mir-122-5p | GDF15 |
| hsa-mir-122-5p | JUND |
| hsa-mir-122-5p | CAMSAP1 |
| hsa-mir-204-5p | LSP1 |
| hsa-mir-204-5p | LSP1 |
| hsa-mir-122-5p | H19 |
| hsa-mir-122-5p | H19 |
| hsa-mir-122-5p | COL5A1 |
| hsa-mir-122-5p | LAMA5 |
| hsa-mir-122-5p | UCK1 |
| hsa-mir-204-5p | PRRC2B |
| hsa-mir-122-5p | YIPF2 |
| hsa-mir-122-5p | EIF2S3 |
| hsa-mir-122-5p | SESN2 |
| hsa-mir-122-5p | SESN2 |
| hsa-mir-204-5p | CASZ1 |
| hsa-mir-122-5p | SLC35D2 |
| hsa-mir-122-5p | SLC35D2 |
| hsa-mir-204-5p | LATS1 |
| hsa-mir-122-5p | RBM39 |
| hsa-mir-122-5p | GSE1 |
| hsa-mir-204-5p | PPT1 |
| hsa-mir-122-5p | RLIM |
| hsa-mir-204-5p | NR1H2 |
| hsa-mir-204-5p | ACLY |
| hsa-mir-122-5p | ANKHD1 |
| hsa-mir-122-5p | DIAPH1 |
| hsa-mir-122-5p | MAP1B |
| hsa-mir-204-5p | ZCCHC9 |
| hsa-mir-122-5p | TOP2A |
| hsa-mir-122-5p | STARD3 |
| hsa-mir-122-5p | PRKAB2 |
| hsa-mir-122-5p | ZNF304 |
| hsa-mir-122-5p | SNRPA1 |
| hsa-mir-122-5p | C19orf12 |
| hsa-mir-122-5p | RAF1 |
| hsa-mir-122-5p | NUP210 |
| hsa-mir-122-5p | ENOSF1 |
| hsa-mir-122-5p | IMMT |
| hsa-mir-122-5p | IQCA1 |
| hsa-mir-122-5p | PRKAA1 |
| hsa-mir-122-5p | PNISR |
| hsa-mir-204-5p | PNISR |
| hsa-mir-122-5p | POPDC3 |
| hsa-mir-204-5p | ANKRD17 |
| hsa-mir-204-5p | UTP3 |
| hsa-mir-122-5p | KDM6B |
| hsa-mir-122-5p | VPS13B |
| hsa-mir-122-5p | SNAP25 |
| hsa-mir-122-5p | DCAF8 |
| hsa-mir-204-5p | DCAF8 |
| hsa-mir-204-5p | NASP |
| hsa-mir-122-5p | ZMYM5 |
| hsa-mir-204-5p | DSTYK |
| hsa-mir-122-5p | DCLK1 |
| hsa-mir-204-5p | POSTN |
| hsa-mir-122-5p | RFXAP |
| hsa-mir-204-5p | TPT1 |
| hsa-mir-122-5p | GPALPP1 |
| hsa-mir-204-5p | BEX1 |
| hsa-mir-122-5p | WDR74 |
| hsa-mir-204-5p | RTN3 |
| hsa-mir-122-5p | SPINK5 |
| hsa-mir-122-5p | COX16 |
| hsa-mir-122-5p | MED6 |
| hsa-mir-204-5p | EIF2S1 |
| hsa-mir-204-5p | IER3IP1 |
| hsa-mir-122-5p | MRPS36 |
| hsa-mir-122-5p | MRPS36 |
| hsa-mir-122-5p | IRAK2 |
| hsa-mir-122-5p | PRPF38B |
| hsa-mir-204-5p | PRPF38B |
| hsa-mir-122-5p | VAV3 |
| hsa-mir-122-5p | SORT1 |
| hsa-mir-122-5p | PPHLN1 |
| hsa-mir-122-5p | FKBP11 |
| hsa-mir-122-5p | TMEM106C |
| hsa-mir-204-5p | YWHAQ |
| hsa-mir-122-5p | IAH1 |
| hsa-mir-204-5p | IL6ST |
| hsa-mir-122-5p | NAV1 |
| hsa-mir-204-5p | RBM17 |
| hsa-mir-122-5p | TMEM241 |
| hsa-mir-204-5p | CDCA8 |
| hsa-mir-204-5p | DSC2 |
| hsa-mir-204-5p | RNF138 |
| hsa-mir-122-5p | TPGS2 |
| hsa-mir-122-5p | COL4A2 |
| hsa-mir-122-5p | ARGLU1 |
| hsa-mir-122-5p | BIVM |
| hsa-mir-204-5p | FBXO21 |
| hsa-mir-122-5p | TBX3 |
| hsa-mir-122-5p | OASL |
| hsa-mir-122-5p | HRK |
| hsa-mir-204-5p | DMTF1 |
| hsa-mir-122-5p | CEP162 |
| hsa-mir-122-5p | EHF |
| hsa-mir-122-5p | CAPRIN1 |
| hsa-mir-122-5p | AVIL |
| hsa-mir-204-5p | ESPL1 |
| hsa-mir-122-5p | KRT7 |
| hsa-mir-122-5p | HNRNPA1 |
| hsa-mir-204-5p | ACVR1B |
| hsa-mir-122-5p | AHI1 |
| hsa-mir-122-5p | USP15 |
| hsa-mir-204-5p | MDM2 |
| hsa-mir-122-5p | DYNC1LI2 |
| hsa-mir-122-5p | TAF5L |
| hsa-mir-122-5p | STX6 |
| hsa-mir-122-5p | DHX9 |
| hsa-mir-122-5p | CEP350 |
| hsa-mir-122-5p | LAMC1 |
| hsa-mir-122-5p | COX5B |
| hsa-mir-122-5p | REV1 |
| hsa-mir-204-5p | TMEM127 |
| hsa-mir-122-5p | ARHGEF4 |
| hsa-mir-204-5p | DRAM1 |
| hsa-mir-122-5p | FLNB |
| hsa-mir-204-5p | FLNB |
| hsa-mir-204-5p | NEK3 |
| hsa-mir-122-5p | PHF11 |
| hsa-mir-122-5p | LMO7 |
| hsa-mir-122-5p | SPDYE1 |
| hsa-mir-122-5p | IGF2BP3 |
| hsa-mir-204-5p | IGF2BP3 |
| hsa-mir-122-5p | IL6 |
| hsa-mir-204-5p | NMT1 |
| hsa-mir-122-5p | SRSF1 |
| hsa-mir-122-5p | DNAJC1 |
| hsa-mir-122-5p | CDK5RAP2 |
| hsa-mir-204-5p | ATP6V1G1 |
| hsa-mir-204-5p | TEX10 |
| hsa-mir-122-5p | GARNL3 |
| hsa-mir-122-5p | PSMB7 |
| hsa-mir-122-5p | MYC |
| hsa-mir-122-5p | DENND4C |
| hsa-mir-122-5p | KIF13A |
| hsa-mir-122-5p | YIPF3 |
| hsa-mir-122-5p | FOXF2 |
| hsa-mir-122-5p | IER3 |
| hsa-mir-122-5p | IER3 |
| hsa-mir-122-5p | IER3 |
| hsa-mir-122-5p | IER3 |
| hsa-mir-122-5p | IER3 |
| hsa-mir-122-5p | IER3 |
| hsa-mir-204-5p | PGBD1 |
| hsa-mir-122-5p | NUMA1 |
| hsa-mir-122-5p | CCDC90B |
| hsa-mir-122-5p | TGS1 |
| hsa-mir-122-5p | NEK1 |
| hsa-mir-204-5p | YAP1 |
| hsa-mir-122-5p | KIF23 |
| hsa-mir-204-5p | ITGA11 |
| hsa-mir-122-5p | HAUS2 |
| hsa-mir-122-5p | UACA |
| hsa-mir-122-5p | IFI44 |
| hsa-mir-122-5p | CYP1B1 |
| hsa-mir-122-5p | RAB1A |
| hsa-mir-122-5p | PREPL |
| hsa-mir-122-5p | FBXO11 |
| hsa-mir-122-5p | ATRAID |
| hsa-mir-122-5p | LRPPRC |
| hsa-mir-122-5p | MYOF |
| hsa-mir-122-5p | KIF11 |
| hsa-mir-204-5p | CUZD1 |
| hsa-mir-122-5p | CEP55 |
| hsa-mir-122-5p | GPR87 |
| hsa-mir-122-5p | IDH1 |
| hsa-mir-122-5p | SENP7 |
| hsa-mir-122-5p | FAM13A |
| hsa-mir-122-5p | AP1AR |
| hsa-mir-122-5p | HNRNPD |
| hsa-mir-122-5p | KIAA1109 |
| hsa-mir-122-5p | BMPR1B |
| hsa-mir-122-5p | LARP1B |
| hsa-mir-122-5p | PRDM5 |
| hsa-mir-122-5p | TRPC3 |
| hsa-mir-122-5p | NUP54 |
| hsa-mir-122-5p | CXCL9 |
| hsa-mir-204-5p | CNOT6L |
| hsa-mir-122-5p | PPA2 |
| hsa-mir-122-5p | FBN2 |
| hsa-mir-204-5p | FBN2 |
| hsa-mir-122-5p | ETV6 |
| hsa-mir-122-5p | C1RL |
| hsa-mir-122-5p | C1RL |
| hsa-mir-122-5p | SCAF11 |
| hsa-mir-122-5p | TMTC3 |
| hsa-mir-122-5p | KERA |
| hsa-mir-122-5p | SYCP3 |
| hsa-mir-122-5p | GAS2L3 |
| hsa-mir-204-5p | TMEM132B |
| hsa-mir-122-5p | TDG |
| hsa-mir-122-5p | SMARCC2 |
| hsa-mir-122-5p | CSAD |
| hsa-mir-122-5p | LMBR1L |
| hsa-mir-204-5p | TMBIM6 |
| hsa-mir-122-5p | TMBIM6 |
| hsa-mir-204-5p | ANKRD52 |
| hsa-mir-122-5p | RBM26 |
| hsa-mir-122-5p | MBNL2 |
| hsa-mir-122-5p | CUL4A |
| hsa-mir-204-5p | STON2 |
| hsa-mir-122-5p | SLC12A6 |
| hsa-mir-122-5p | TCF12 |
| hsa-mir-122-5p | ZSCAN29 |
| hsa-mir-122-5p | GTF2A2 |
| hsa-mir-122-5p | BCL2A1 |
| hsa-mir-122-5p | WDR61 |
| hsa-mir-204-5p | MAN2C1 |
| hsa-mir-122-5p | PCSK6 |
| hsa-mir-122-5p | CYP1A2 |
| hsa-mir-122-5p | ABHD2 |
| hsa-mir-122-5p | MFGE8 |
| hsa-mir-122-5p | UNC45A |
| hsa-mir-122-5p | MCTP2 |
| hsa-mir-122-5p | FURIN |
| hsa-mir-122-5p | IQGAP1 |
| hsa-mir-204-5p | IQGAP1 |
| hsa-mir-204-5p | CRTC3 |
| hsa-mir-122-5p | ZFHX3 |
| hsa-mir-122-5p | NLRC5 |
| hsa-mir-122-5p | PDPK1 |
| hsa-mir-122-5p | TCF25 |
| hsa-mir-204-5p | CLTC |
| hsa-mir-204-5p | RPRD1A |
| hsa-mir-122-5p | ESCO1 |
| hsa-mir-122-5p | GREB1L |
| hsa-mir-122-5p | PELP1 |
| hsa-mir-122-5p | MINK1 |
| hsa-mir-204-5p | MINK1 |
| hsa-mir-122-5p | TP53 |
| hsa-mir-122-5p | TMC6 |
| hsa-mir-122-5p | SLC16A3 |
| hsa-mir-204-5p | FOXK2 |
| hsa-mir-122-5p | SECTM1 |
| hsa-mir-204-5p | RNF157 |
| hsa-mir-122-5p | RNF165 |
| hsa-mir-122-5p | SMAD4 |
| hsa-mir-122-5p | NFIC |
| hsa-mir-122-5p | PRDM15 |
| hsa-mir-122-5p | AKT1 |
| hsa-mir-122-5p | RERE |
| hsa-mir-204-5p | HSPG2 |
| hsa-mir-204-5p | RPS8 |
| hsa-mir-122-5p | LMO4 |
| hsa-mir-122-5p | ATP1B1 |
| hsa-mir-204-5p | ATP1B1 |
| hsa-mir-122-5p | DCAF6 |
| hsa-mir-122-5p | POU2F1 |
| hsa-mir-204-5p | PFDN2 |
| hsa-mir-204-5p | ISG20L2 |
| hsa-mir-204-5p | HDGF |
| hsa-mir-122-5p | XPR1 |
| hsa-mir-122-5p | TOR1AIP1 |
| hsa-mir-122-5p | ADAMTSL4 |
| hsa-mir-204-5p | MCL1 |
| hsa-mir-122-5p | PI4KB |
| hsa-mir-204-5p | ANP32E |
| hsa-mir-122-5p | ENSA |
| hsa-mir-122-5p | GOLPH3L |
| hsa-mir-122-5p | INTS7 |
| hsa-mir-122-5p | KCNN3 |
| hsa-mir-122-5p | GATAD2B |
| hsa-mir-122-5p | GATAD2B |
| hsa-mir-204-5p | RIT1 |
| hsa-mir-204-5p | FBXO28 |
| hsa-mir-122-5p | ARF1 |
| hsa-mir-122-5p | CDC42BPA |
| hsa-mir-122-5p | PLEKHA6 |
| hsa-mir-204-5p | RHOB |
| hsa-mir-122-5p | ASXL2 |
| hsa-mir-204-5p | ASXL2 |
| hsa-mir-122-5p | SNRNP200 |
| hsa-mir-122-5p | MRPS5 |
| hsa-mir-204-5p | MRPS5 |
| hsa-mir-204-5p | EXOC6B |
| hsa-mir-122-5p | DQX1 |
| hsa-mir-122-5p | SLC20A1 |
| hsa-mir-204-5p | UBXN4 |
| hsa-mir-122-5p | SCRN3 |
| hsa-mir-122-5p | TMEFF2 |
| hsa-mir-122-5p | CDCA7 |
| hsa-mir-122-5p | PHOSPHO2 |
| hsa-mir-122-5p | CCDC150 |
| hsa-mir-122-5p | RHBDD1 |
| hsa-mir-122-5p | ACKR3 |
| hsa-mir-122-5p | COPS7B |
| hsa-mir-122-5p | DIS3L2 |
| hsa-mir-122-5p | TAMM41 |
| hsa-mir-122-5p | TAMM41 |
| hsa-mir-122-5p | VGLL4 |
| hsa-mir-122-5p | RBMS3 |
| hsa-mir-122-5p | GOLGA4 |
| hsa-mir-122-5p | CTDSPL |
| hsa-mir-122-5p | TMF1 |
| hsa-mir-204-5p | LIMD1 |
| hsa-mir-122-5p | NFKBIZ |
| hsa-mir-122-5p | COL8A1 |
| hsa-mir-204-5p | ATG3 |
| hsa-mir-122-5p | NCEH1 |
| hsa-mir-122-5p | LPP |
| hsa-mir-122-5p | TMEM44 |
| hsa-mir-204-5p | MANF |
| hsa-mir-122-5p | SCD5 |
| hsa-mir-122-5p | USP53 |
| hsa-mir-122-5p | CBR4 |
| hsa-mir-122-5p | MYO10 |
| hsa-mir-122-5p | RPL37 |
| hsa-mir-204-5p | PAM |
| hsa-mir-204-5p | BDP1 |
| hsa-mir-204-5p | BDP1 |
| hsa-mir-204-5p | BDP1 |
| hsa-mir-122-5p | BTF3 |
| hsa-mir-122-5p | FBXL17 |
| hsa-mir-122-5p | DDX46 |
| hsa-mir-122-5p | RNF145 |
| hsa-mir-122-5p | G3BP1 |
| hsa-mir-204-5p | G3BP1 |
| hsa-mir-122-5p | GFOD1 |
| hsa-mir-122-5p | CDKAL1 |
| hsa-mir-122-5p | SCUBE3 |
| hsa-mir-204-5p | MMS22L |
| hsa-mir-204-5p | TIAM2 |
| hsa-mir-204-5p | TMEM181 |
| hsa-mir-204-5p | WTAP |
| hsa-mir-204-5p | ZMYM4 |
| hsa-mir-122-5p | SLC22A3 |
| hsa-mir-122-5p | VWDE |
| hsa-mir-204-5p | RBAK |
| hsa-mir-122-5p | PURB |
| hsa-mir-204-5p | PURB |
| hsa-mir-204-5p | ZNF92 |
| hsa-mir-122-5p | ATXN7L1 |
| hsa-mir-122-5p | TRIM4 |
| hsa-mir-122-5p | SPIN2A |
| hsa-mir-204-5p | CCDC120 |
| hsa-mir-122-5p | OGT |
| hsa-mir-122-5p | MFHAS1 |
| hsa-mir-122-5p | MFHAS1 |
| hsa-mir-122-5p | SLC25A37 |
| hsa-mir-122-5p | DOCK5 |
| hsa-mir-122-5p | ERLIN2 |
| hsa-mir-122-5p | SLC26A7 |
| hsa-mir-122-5p | MTDH |
| hsa-mir-122-5p | ZCCHC7 |
| hsa-mir-122-5p | ASTN2 |
| hsa-mir-204-5p | SURF4 |
| hsa-mir-204-5p | SURF4 |
| hsa-mir-122-5p | SURF1 |
| hsa-mir-122-5p | SURF1 |
| hsa-mir-122-5p | SH3GLB2 |
| hsa-mir-122-5p | NOTCH1 |
| hsa-mir-122-5p | POLR3A |
| hsa-mir-122-5p | HERC4 |
| hsa-mir-122-5p | PPRC1 |
| hsa-mir-122-5p | ITPRIP |
| hsa-mir-122-5p | CNNM2 |
| hsa-mir-122-5p | EIF3M |
| hsa-mir-204-5p | EIF3M |
| hsa-mir-204-5p | SSRP1 |
| hsa-mir-204-5p | PTPRJ |
| hsa-mir-204-5p | ARFGAP2 |
| hsa-mir-122-5p | CELF1 |
| hsa-mir-122-5p | INTS4 |
| hsa-mir-122-5p | ATM |
| hsa-mir-204-5p | AASDHPPT |
| hsa-mir-122-5p | ST14 |
| hsa-mir-122-5p | ADAM33 |
| hsa-mir-204-5p | INCENP |
| hsa-mir-122-5p | TMEM25 |
| hsa-mir-122-5p | SOGA1 |
| hsa-mir-204-5p | SOGA1 |
| hsa-mir-122-5p | LSM14B |
| hsa-mir-122-5p | ITGB1 |
| hsa-mir-122-5p | LATS2 |
| hsa-mir-204-5p | PDCD4 |
| hsa-mir-204-5p | PRSS23 |
| hsa-mir-204-5p | TMEM263 |
| hsa-mir-122-5p | GXYLT1 |
| hsa-mir-122-5p | DIP2C |
| hsa-mir-122-5p | NPAS3 |
| hsa-mir-122-5p | KCTD14 |
| hsa-mir-122-5p | ME3 |
| hsa-mir-122-5p | C4orf33 |
| hsa-mir-122-5p | PTPRO |
| hsa-mir-122-5p | VTI1A |
| hsa-mir-122-5p | MMAA |
| hsa-mir-122-5p | ZNF827 |
| hsa-mir-204-5p | ZNF827 |
| hsa-mir-204-5p | ADAM17 |
| hsa-mir-204-5p | FLI1 |
| hsa-mir-122-5p | TMEM45B |
| hsa-mir-122-5p | BICD1 |
| hsa-mir-122-5p | PABPC3 |
| hsa-mir-122-5p | DST |
| hsa-mir-204-5p | BAG3 |
| hsa-mir-122-5p | MZT2B |
| hsa-mir-204-5p | PTPN14 |
| hsa-mir-204-5p | POU4F1 |
| hsa-mir-122-5p | EPG5 |
| hsa-mir-204-5p | TGOLN2 |
| hsa-mir-204-5p | UHMK1 |
| hsa-mir-122-5p | DCLRE1C |
| hsa-mir-122-5p | NMT2 |
| hsa-mir-122-5p | USP12 |
| hsa-mir-204-5p | TMEM123 |
| hsa-mir-122-5p | MBNL1 |
| hsa-mir-122-5p | GJA1 |
| hsa-mir-122-5p | FARP1 |
| hsa-mir-122-5p | HNRNPDL |
| hsa-mir-204-5p | HNRNPDL |
| hsa-mir-122-5p | BCL2L11 |
| hsa-mir-204-5p | CAST |
| hsa-mir-204-5p | RASSF3 |
| hsa-mir-122-5p | RANBP2 |
| hsa-mir-204-5p | RANBP2 |
| hsa-mir-204-5p | RBMS1 |
| hsa-mir-122-5p | SLC25A27 |
| hsa-mir-122-5p | CMTM7 |
| hsa-mir-122-5p | CFDP1 |
| hsa-mir-204-5p | ZDHHC7 |
| hsa-mir-122-5p | CMIP |
| hsa-mir-122-5p | KCTD15 |
| hsa-mir-122-5p | DDAH1 |
| hsa-mir-204-5p | SREK1 |
| hsa-mir-204-5p | CHD1 |
| hsa-mir-122-5p | MSI2 |
| hsa-mir-122-5p | JPH3 |
| hsa-mir-122-5p | ANKH |
| hsa-mir-122-5p | TBRG1 |
| hsa-mir-122-5p | ABCA5 |
| hsa-mir-122-5p | OBSCN |
| hsa-mir-122-5p | LONRF1 |
| hsa-mir-122-5p | LONRF1 |
| hsa-mir-122-5p | PPP1R3A |
| hsa-mir-122-5p | SH3RF1 |
| hsa-mir-122-5p | BUB3 |
| hsa-mir-204-5p | ADAMTS1 |
| hsa-mir-204-5p | ADAMTS5 |
| hsa-mir-122-5p | FGD5 |
| hsa-mir-204-5p | FLCN |
| hsa-mir-122-5p | EME1 |
| hsa-mir-204-5p | AGPAT5 |
| hsa-mir-204-5p | AGPAT5 |
| hsa-mir-122-5p | USP25 |
| hsa-mir-204-5p | LARP1 |
| hsa-mir-122-5p | MIER3 |
| hsa-mir-204-5p | FAM126B |
| hsa-mir-122-5p | RMND1 |
| hsa-mir-122-5p | PSD3 |
| hsa-mir-122-5p | GNAQ |
| hsa-mir-122-5p | DRAM2 |
| hsa-mir-122-5p | BACH1 |
| hsa-mir-122-5p | SCAF4 |
| hsa-mir-122-5p | FGF18 |
| hsa-mir-122-5p | EEF1A1 |
| hsa-mir-204-5p | ZDHHC5 |
| hsa-mir-122-5p | BAG4 |
| hsa-mir-122-5p | VPS8 |
| hsa-mir-122-5p | EIF4A2 |
| hsa-mir-122-5p | BRPF1 |
| hsa-mir-122-5p | ATP2B2 |
| hsa-mir-204-5p | SMG1 |
| hsa-mir-122-5p | FZD1 |
| hsa-mir-122-5p | TMEM164 |
| hsa-mir-122-5p | TAB3 |
| hsa-mir-204-5p | TAB3 |
| hsa-mir-122-5p | SKI |
| hsa-mir-204-5p | SKI |
| hsa-mir-122-5p | RNF207 |
| hsa-mir-204-5p | EIF5B |
| hsa-mir-122-5p | RIBC1 |
| hsa-mir-204-5p | B4GALT5 |
| hsa-mir-204-5p | PPP1R9A |
| hsa-mir-204-5p | TMED4 |
| hsa-mir-122-5p | CDA |
| hsa-mir-122-5p | PAXBP1 |
| hsa-mir-122-5p | PAXBP1 |
| hsa-mir-122-5p | GART |
| hsa-mir-122-5p | GART |
| hsa-mir-122-5p | SON |
| hsa-mir-122-5p | UBE2Z |
| hsa-mir-204-5p | UBE2Z |
| hsa-mir-122-5p | RUNX1 |
| hsa-mir-204-5p | IGF2BP1 |
| hsa-mir-122-5p | CTBP1 |
| hsa-mir-204-5p | CTBP1 |
| hsa-mir-122-5p | ABR |
| hsa-mir-122-5p | ABR |
| hsa-mir-122-5p | ABR |
| hsa-mir-204-5p | BSDC1 |
| hsa-mir-122-5p | WDR4 |
| hsa-mir-122-5p | U2AF1 |
| hsa-mir-122-5p | G6PD |
| hsa-mir-122-5p | RRP1 |
| hsa-mir-122-5p | TRAPPC10 |
| hsa-mir-122-5p | DIP2A |
| hsa-mir-122-5p | PRMT2 |
| hsa-mir-204-5p | GPSM1 |
| hsa-mir-122-5p | RDH13 |
| hsa-mir-122-5p | RDH13 |
| hsa-mir-122-5p | RDH13 |
| hsa-mir-122-5p | RDH13 |
| hsa-mir-122-5p | RDH13 |
| hsa-mir-122-5p | RDH13 |
| hsa-mir-122-5p | RDH13 |
| hsa-mir-122-5p | RDH13 |
| hsa-mir-122-5p | RDH13 |
| hsa-mir-122-5p | RDH13 |
| hsa-mir-122-5p | ZER1 |
| hsa-mir-122-5p | IL6R |
| hsa-mir-122-5p | UBQLN4 |
| hsa-mir-122-5p | LRRC14 |
| hsa-mir-122-5p | ZNF333 |
| hsa-mir-122-5p | SQSTM1 |
| hsa-mir-122-5p | SQSTM1 |
| hsa-mir-204-5p | MAML1 |
| hsa-mir-204-5p | MAML1 |
| hsa-mir-122-5p | NAPEPLD |
| hsa-mir-122-5p | NAPEPLD |
| hsa-mir-122-5p | RAVER1 |
| hsa-mir-204-5p | RAVER1 |
| hsa-mir-122-5p | SENP3 |
| hsa-mir-122-5p | EIF4A1 |
| hsa-mir-122-5p | WDR90 |
| hsa-mir-122-5p | SYVN1 |
| hsa-mir-204-5p | SYVN1 |
| hsa-mir-122-5p | LRP5 |
| hsa-mir-122-5p | SLC25A34 |
| hsa-mir-122-5p | RBBP4 |
| hsa-mir-122-5p | CAMK2N1 |
| hsa-mir-122-5p | MEGF6 |
| hsa-mir-122-5p | USP1 |
| hsa-mir-122-5p | FUBP1 |
| hsa-mir-122-5p | FLVCR1 |
| hsa-mir-122-5p | RBM15 |
| hsa-mir-204-5p | RNF149 |
| hsa-mir-122-5p | DHX57 |
| hsa-mir-204-5p | DHX57 |
| hsa-mir-122-5p | CCNYL1 |
| hsa-mir-122-5p | SLC22A15 |
| hsa-mir-122-5p | AZI2 |
| hsa-mir-122-5p | STT3B |
| hsa-mir-122-5p | IFI16 |
| hsa-mir-122-5p | GTPBP8 |
| hsa-mir-122-5p | PTPN13 |
| hsa-mir-122-5p | ATXN7 |
| hsa-mir-122-5p | ATXN7 |
| hsa-mir-122-5p | TIPARP |
| hsa-mir-122-5p | SMIM14 |
| hsa-mir-122-5p | RPP14 |
| hsa-mir-122-5p | APBB2 |
| hsa-mir-122-5p | CRELD1 |
| hsa-mir-122-5p | U2SURP |
| hsa-mir-122-5p | TTC14 |
| hsa-mir-204-5p | PLB1 |
| hsa-mir-122-5p | LIPH |
| hsa-mir-122-5p | PBRM1 |
| hsa-mir-204-5p | ZNF691 |
| hsa-mir-204-5p | DNAJB14 |
| hsa-mir-122-5p | CDC25A |
| hsa-mir-122-5p | FBXW12 |
| hsa-mir-122-5p | MST1R |
| hsa-mir-122-5p | WDR82 |
| hsa-mir-122-5p | TMEM144 |
| hsa-mir-122-5p | NAA15 |
| hsa-mir-122-5p | FAM160A1 |
| hsa-mir-122-5p | NIPBL |
| hsa-mir-122-5p | SLC25A46 |
| hsa-mir-122-5p | CMBL |
| hsa-mir-122-5p | WDR41 |
| hsa-mir-122-5p | SCGB3A2 |
| hsa-mir-122-5p | ERAP1 |
| hsa-mir-204-5p | CFAP97 |
| hsa-mir-122-5p | RICTOR |
| hsa-mir-122-5p | ANKRA2 |
| hsa-mir-122-5p | FOXQ1 |
| hsa-mir-204-5p | CITED2 |
| hsa-mir-122-5p | CREBRF |
| hsa-mir-122-5p | SFXN1 |
| hsa-mir-122-5p | STXBP5 |
| hsa-mir-122-5p | TRA2A |
| hsa-mir-122-5p | SAP30L |
| hsa-mir-122-5p | ZNF12 |
| hsa-mir-204-5p | ZNF704 |
| hsa-mir-122-5p | CTSB |
| hsa-mir-122-5p | CTSB |
| hsa-mir-204-5p | OSGIN2 |
| hsa-mir-122-5p | SPAG11B |
| hsa-mir-122-5p | SPAG11B |
| hsa-mir-122-5p | SPAG11B |
| hsa-mir-122-5p | RASEF |
| hsa-mir-122-5p | STRBP |
| hsa-mir-122-5p | GAPVD1 |
| hsa-mir-122-5p | TRMT10B |
| hsa-mir-204-5p | BRWD3 |
| hsa-mir-122-5p | BRWD3 |
| hsa-mir-122-5p | PGM2L1 |
| hsa-mir-122-5p | ZNF22 |
| hsa-mir-122-5p | EML5 |
| hsa-mir-122-5p | NEMF |
| hsa-mir-122-5p | BEND7 |
| hsa-mir-122-5p | NSD1 |
| hsa-mir-204-5p | GHITM |
| hsa-mir-122-5p | TSC1 |
| hsa-mir-122-5p | METTL3 |
| hsa-mir-204-5p | HSPA12A |
| hsa-mir-122-5p | TC2N |
| hsa-mir-122-5p | TC2N |
| hsa-mir-122-5p | IFI27 |
| hsa-mir-122-5p | IFI27 |
| hsa-mir-122-5p | NELL1 |
| hsa-mir-204-5p | PTER |
| hsa-mir-204-5p | ARL5B |
| hsa-mir-204-5p | SMCO4 |
| hsa-mir-122-5p | KCNC2 |
| hsa-mir-122-5p | TAF1D |
| hsa-mir-204-5p | R3HCC1L |
| hsa-mir-122-5p | SPRED1 |
| hsa-mir-204-5p | SENP8 |
| hsa-mir-122-5p | CLMP |
| hsa-mir-122-5p | STXBP4 |
| hsa-mir-122-5p | WBP1L |
| hsa-mir-122-5p | ANAPC16 |
| hsa-mir-122-5p | TRIM44 |
| hsa-mir-122-5p | TPP1 |
| hsa-mir-204-5p | TPP1 |
| hsa-mir-122-5p | NETO1 |
| hsa-mir-122-5p | AKIP1 |
| hsa-mir-122-5p | ATMIN |
| hsa-mir-122-5p | TMX3 |
| hsa-mir-204-5p | WEE1 |
| hsa-mir-122-5p | CCDC68 |
| hsa-mir-122-5p | RIMKLB |
| hsa-mir-122-5p | HSP90B1 |
| hsa-mir-204-5p | HSP90B1 |
| hsa-mir-122-5p | B2M |
| hsa-mir-122-5p | B2M |
| hsa-mir-122-5p | CASC4 |
| hsa-mir-122-5p | AP1G1 |
| hsa-mir-122-5p | SLFN5 |
| hsa-mir-122-5p | CATSPER2 |
| hsa-mir-122-5p | ANPEP |
| hsa-mir-204-5p | NAV2 |
| hsa-mir-122-5p | C18orf54 |
| hsa-mir-122-5p | NAB2 |
| hsa-mir-122-5p | PATL1 |
| hsa-mir-204-5p | GREM1 |
| hsa-mir-204-5p | GREM1 |
| hsa-mir-204-5p | GREM1 |
| hsa-mir-204-5p | MAP1A |
| hsa-mir-204-5p | RCCD1 |
| hsa-mir-122-5p | ACSF2 |
| hsa-mir-204-5p | URM1 |
| hsa-mir-122-5p | MIDN |
| hsa-mir-204-5p | CDT1 |
| hsa-mir-122-5p | ZNF641 |
| hsa-mir-204-5p | KMT2D |
| hsa-mir-122-5p | TUBA1A |
| hsa-mir-122-5p | PSCA |
| hsa-mir-204-5p | EEF2 |
| hsa-mir-122-5p | TSR1 |
| hsa-mir-122-5p | KLK13 |
| hsa-mir-122-5p | ANGPTL4 |
| hsa-mir-122-5p | PRDX2 |
| hsa-mir-122-5p | RAB26 |
| hsa-mir-122-5p | SRRM2 |
| hsa-mir-204-5p | DDB1 |
| hsa-mir-122-5p | SF1 |
| hsa-mir-122-5p | SCARA3 |
| hsa-mir-122-5p | SETD5 |
| hsa-mir-204-5p | SETD5 |
| hsa-mir-204-5p | MAPK1IP1L |
| hsa-mir-122-5p | DNAJC7 |
| hsa-mir-122-5p | IRF2BP2 |
| hsa-mir-204-5p | KIF5C |
| hsa-mir-204-5p | KIF5C |
| hsa-mir-204-5p | PDHB |
| hsa-mir-122-5p | PXK |
| hsa-mir-204-5p | IRF2 |
| hsa-mir-122-5p | SCN11A |
| hsa-mir-204-5p | ING5 |
| hsa-mir-122-5p | ATXN2L |
| hsa-mir-204-5p | POLR3D |
| hsa-mir-204-5p | MTCL1 |
| hsa-mir-122-5p | GBX2 |
| hsa-mir-204-5p | COL3A1 |
| hsa-mir-122-5p | ZNF30 |
| hsa-mir-122-5p | IL7R |
| hsa-mir-122-5p | NPNT |
| hsa-mir-122-5p | SEMA4C |
| hsa-mir-204-5p | TET2 |
| hsa-mir-204-5p | CHTF8 |
| hsa-mir-204-5p | CHTF8 |
| hsa-mir-122-5p | IL12A |
| hsa-mir-122-5p | COL4A3 |
| hsa-mir-122-5p | GOT1L1 |
| hsa-mir-122-5p | TBC1D10B |
| hsa-mir-204-5p | MMGT1 |
| hsa-mir-122-5p | MMGT1 |
| hsa-mir-122-5p | ZEB2 |
| hsa-mir-122-5p | GKN1 |
| hsa-mir-122-5p | C15orf40 |
| hsa-mir-122-5p | HIC2 |
| hsa-mir-122-5p | ZNF32 |
| hsa-mir-122-5p | UGP2 |
| hsa-mir-122-5p | PCDH7 |
| hsa-mir-122-5p | MUC3A |
| hsa-mir-204-5p | TOR1AIP2 |
| hsa-mir-122-5p | KLF13 |
| hsa-mir-122-5p | KLF13 |
| hsa-mir-122-5p | TMEM154 |
| hsa-mir-122-5p | ALCAM |
| hsa-mir-122-5p | HNRNPA3 |
| hsa-mir-122-5p | RNF150 |
| hsa-mir-204-5p | PWWP2A |
| hsa-mir-122-5p | USP47 |
| hsa-mir-122-5p | FAM161A |
| hsa-mir-204-5p | CRTAP |
| hsa-mir-122-5p | TMED10 |
| hsa-mir-122-5p | SMAD1 |
| hsa-mir-122-5p | SP7 |
| hsa-mir-122-5p | STAT2 |
| hsa-mir-122-5p | RNF34 |
| hsa-mir-122-5p | POLH |
| hsa-mir-204-5p | KIF5B |
| hsa-mir-122-5p | AKAP13 |
| hsa-mir-122-5p | USP32 |
| hsa-mir-122-5p | KIAA0232 |
| hsa-mir-122-5p | TMEM43 |
| hsa-mir-122-5p | OSCAR |
| hsa-mir-122-5p | OSCAR |
| hsa-mir-122-5p | OSCAR |
| hsa-mir-122-5p | OSCAR |
| hsa-mir-122-5p | OSCAR |
| hsa-mir-122-5p | OSCAR |
| hsa-mir-122-5p | OSCAR |
| hsa-mir-122-5p | OSCAR |
| hsa-mir-122-5p | OSCAR |
| hsa-mir-122-5p | OSCAR |
| hsa-mir-122-5p | TANC2 |
| hsa-mir-122-5p | PKIA |
| hsa-mir-122-5p | MTM1 |
| hsa-mir-122-5p | TRMT61B |
| hsa-mir-122-5p | INSR |
| hsa-mir-122-5p | C9orf16 |
| hsa-mir-122-5p | ZNF692 |
| hsa-mir-122-5p | SHCBP1 |
| hsa-mir-122-5p | CHD7 |
| hsa-mir-122-5p | ESCO2 |
| hsa-mir-122-5p | ASXL1 |
| hsa-mir-204-5p | ZNF562 |
| hsa-mir-122-5p | RSL1D1 |
| hsa-mir-122-5p | ZNF274 |
| hsa-mir-122-5p | PIK3CD |
| hsa-mir-122-5p | SPSB1 |
| hsa-mir-122-5p | ATF7IP |
| hsa-mir-122-5p | PWWP2B |
| hsa-mir-122-5p | MLLT3 |
| hsa-mir-204-5p | SCG2 |
| hsa-mir-122-5p | JMJD1C |
| hsa-mir-204-5p | JMJD1C |
| hsa-mir-122-5p | SMN1 |
| hsa-mir-122-5p | SMN1 |
| hsa-mir-122-5p | SMN1 |
| hsa-mir-122-5p | SMN1 |
| hsa-mir-122-5p | SMN1 |
| hsa-mir-122-5p | NME6 |
| hsa-mir-122-5p | CYCS |
| hsa-mir-122-5p | TEFM |
| hsa-mir-122-5p | ISG20 |
| hsa-mir-122-5p | ID4 |
| hsa-mir-122-5p | ZNF131 |
| hsa-mir-122-5p | TP53RK |
| hsa-mir-204-5p | GTPBP2 |
| hsa-mir-122-5p | MANEA |
| hsa-mir-122-5p | AFF1 |
| hsa-mir-204-5p | AFF1 |
| hsa-mir-204-5p | HCFC1 |
| hsa-mir-122-5p | DCP2 |
| hsa-mir-204-5p | DCP2 |
| hsa-mir-122-5p | RPL38 |
| hsa-mir-122-5p | CES2 |
| hsa-mir-122-5p | ANKRD13D |
| hsa-mir-204-5p | LCLAT1 |
| hsa-mir-122-5p | HECTD4 |
| hsa-mir-122-5p | FAM222B |
| hsa-mir-122-5p | BNC2 |
| hsa-mir-204-5p | COQ2 |
| hsa-mir-122-5p | KDM2A |
| hsa-mir-122-5p | RAPH1 |
| hsa-mir-204-5p | RAPH1 |
| hsa-mir-122-5p | VANGL1 |
| hsa-mir-122-5p | SLC2A14 |
| hsa-mir-122-5p | MMRN2 |
| hsa-mir-122-5p | CHD2 |
| hsa-mir-122-5p | NMNAT1 |
| hsa-mir-122-5p | HEG1 |
| hsa-mir-204-5p | HEG1 |
| hsa-mir-204-5p | STAT5B |
| hsa-mir-122-5p | RNF213 |
| hsa-mir-122-5p | NET1 |
| hsa-mir-204-5p | HOXB2 |
| hsa-mir-122-5p | RBM4 |
| hsa-mir-122-5p | XXYLT1 |
| hsa-mir-122-5p | UBXN2A |
| hsa-mir-122-5p | FBXO45 |
| hsa-mir-204-5p | MGA |
| hsa-mir-122-5p | C12orf66 |
| hsa-mir-204-5p | PITPNA |
| hsa-mir-204-5p | ZBTB4 |
| hsa-mir-204-5p | ZBTB4 |
| hsa-mir-122-5p | ZHX3 |
| hsa-mir-122-5p | ANKRD36C |
| hsa-mir-122-5p | AKIRIN1 |
| hsa-mir-122-5p | ANGEL2 |
| hsa-mir-122-5p | FZD4 |
| hsa-mir-122-5p | GLMN |
| hsa-mir-122-5p | DHX36 |
| hsa-mir-122-5p | DHX36 |
| hsa-mir-122-5p | CADM2 |
| hsa-mir-122-5p | CADM2 |
| hsa-mir-122-5p | DDIT3 |
| hsa-mir-122-5p | ARHGAP1 |
| hsa-mir-122-5p | GOLGA8A |
| hsa-mir-122-5p | PHYKPL |
| hsa-mir-122-5p | SMAD2 |
| hsa-mir-204-5p | LPL |
| hsa-mir-122-5p | CCDC14 |
| hsa-mir-204-5p | DENND2C |
| hsa-mir-122-5p | B3GALT6 |
| hsa-mir-122-5p | JAKMIP2 |
| hsa-mir-122-5p | HSF5 |
| hsa-mir-122-5p | ENTHD1 |
| hsa-mir-122-5p | RTTN |
| hsa-mir-122-5p | SLC35G1 |
| hsa-mir-122-5p | EID2B |
| hsa-mir-122-5p | LPCAT4 |
| hsa-mir-122-5p | SLCO3A1 |
| hsa-mir-122-5p | PRR15 |
| hsa-mir-122-5p | MYO1D |
| hsa-mir-122-5p | CDK5R1 |
| hsa-mir-122-5p | WSB2 |
| hsa-mir-204-5p | SOX11 |
| hsa-mir-122-5p | CHD9 |
| hsa-mir-204-5p | TIMM22 |
| hsa-mir-204-5p | TIMM22 |
| hsa-mir-204-5p | TIMM22 |
| hsa-mir-122-5p | TGIF1 |
| hsa-mir-204-5p | ST8SIA3 |
| hsa-mir-122-5p | RPLP2 |
| hsa-mir-122-5p | CSTF2T |
| hsa-mir-122-5p | AGTRAP |
| hsa-mir-122-5p | ZNF518A |
| hsa-mir-122-5p | ZNF619 |
| hsa-mir-122-5p | ZBTB41 |
| hsa-mir-204-5p | PDE4DIP |
| hsa-mir-122-5p | DDX10 |
| hsa-mir-122-5p | DALRD3 |
| hsa-mir-122-5p | LCORL |
| hsa-mir-204-5p | PARD6G |
| hsa-mir-122-5p | GALNT11 |
| hsa-mir-204-5p | WDR6 |
| hsa-mir-122-5p | GEN1 |
| hsa-mir-204-5p | GEN1 |
| hsa-mir-122-5p | DNAJC22 |
| hsa-mir-122-5p | OTOS |
| hsa-mir-122-5p | ZNF713 |
| hsa-mir-122-5p | SUZ12 |
| hsa-mir-122-5p | KCTD12 |
| hsa-mir-122-5p | ZBTB7A |
| hsa-mir-122-5p | AURKB |
| hsa-mir-122-5p | FAM133A |
| hsa-mir-122-5p | DAND5 |
| hsa-mir-122-5p | PTPN11 |
| hsa-mir-122-5p | PACS2 |
| hsa-mir-204-5p | ZBTB18 |
| hsa-mir-122-5p | ARL14 |
| hsa-mir-122-5p | FLJ37453 |
| hsa-mir-122-5p | PDXDC1 |
| hsa-mir-122-5p | PDXDC1 |
| hsa-mir-122-5p | PDXDC1 |
| hsa-mir-204-5p | SEPHS2 |
| hsa-mir-122-5p | TMEM86B |
| hsa-mir-122-5p | EXOC3 |
| hsa-mir-122-5p | ZNF816 |
| hsa-mir-204-5p | CCDC66 |
| hsa-mir-204-5p | GAS1 |
| hsa-mir-204-5p | RNF182 |
| hsa-mir-122-5p | RNF182 |
| hsa-mir-204-5p | YOD1 |
| hsa-mir-122-5p | C3orf22 |
| hsa-mir-122-5p | HOXC9 |
| hsa-mir-122-5p | OXTR |
| hsa-mir-122-5p | SLC26A11 |
| hsa-mir-122-5p | TMEM102 |
| hsa-mir-122-5p | TMEM102 |
| hsa-mir-122-5p | SGSH |
| hsa-mir-122-5p | GPR135 |
| hsa-mir-122-5p | TNFSF15 |
| hsa-mir-122-5p | RELL1 |
| hsa-mir-122-5p | C5orf24 |
| hsa-mir-122-5p | CHST15 |
| hsa-mir-122-5p | TNRC18 |
| hsa-mir-122-5p | ZNF708 |
| hsa-mir-122-5p | EXT1 |
| hsa-mir-122-5p | YBEY |
| hsa-mir-122-5p | PLCXD1 |
| hsa-mir-122-5p | EXOC7 |
| hsa-mir-122-5p | CEP97 |
| hsa-mir-122-5p | SATB1 |
| hsa-mir-122-5p | TRAK1 |
| hsa-mir-122-5p | TTC3 |
| hsa-mir-204-5p | TTC3 |
| hsa-mir-122-5p | PAPPA |
| hsa-mir-204-5p | GSTA5 |
| hsa-mir-204-5p | C16orf72 |
| hsa-mir-122-5p | RPL35A |
| hsa-mir-122-5p | EWSR1 |
| hsa-mir-204-5p | PTTG1IP |
| hsa-mir-122-5p | BCOR |
| hsa-mir-122-5p | KIAA2026 |
| hsa-mir-122-5p | ASB7 |
| hsa-mir-122-5p | TANGO2 |
| hsa-mir-122-5p | SFXN4 |
| hsa-mir-122-5p | IQGAP3 |
| hsa-mir-122-5p | PTP4A2 |
| hsa-mir-122-5p | DENND5A |
| hsa-mir-204-5p | DENND5A |
| hsa-mir-122-5p | EIF3C |
| hsa-mir-204-5p | IRAK1 |
| hsa-mir-122-5p | PCDH9 |
| hsa-mir-122-5p | POU6F1 |
| hsa-mir-122-5p | MAP7D2 |
| hsa-mir-122-5p | SS18L1 |
| hsa-mir-122-5p | SNN |
| hsa-mir-122-5p | NELL2 |
| hsa-mir-122-5p | ZBTB40 |
| hsa-mir-122-5p | FAM110C |
| hsa-mir-122-5p | SUMO3 |
| hsa-mir-122-5p | JAG2 |
| hsa-mir-122-5p | MUC6 |
| hsa-mir-122-5p | MUC6 |
| hsa-mir-122-5p | MUC6 |
| hsa-mir-122-5p | MUC6 |
| hsa-mir-204-5p | AP3M1 |
| hsa-mir-204-5p | SEMA4B |
| hsa-mir-122-5p | FAF1 |
| hsa-mir-122-5p | PRPF39 |
| hsa-mir-122-5p | UBALD2 |
| hsa-mir-122-5p | ARL15 |
| hsa-mir-122-5p | CDK10 |
| hsa-mir-122-5p | SP140L |
| hsa-mir-122-5p | METTL7A |
| hsa-mir-122-5p | IFNLR1 |
| hsa-mir-122-5p | LMLN |
| hsa-mir-122-5p | BRWD1 |
| hsa-mir-204-5p | BRWD1 |
| hsa-mir-122-5p | MYBL1 |
| hsa-mir-122-5p | ADARB2 |
| hsa-mir-122-5p | IFIT1 |
| hsa-mir-204-5p | MORF4L1 |
| hsa-mir-122-5p | ARL17A |
| hsa-mir-122-5p | ARL17A |
| hsa-mir-122-5p | ARL17A |
| hsa-mir-122-5p | ARL17A |
| hsa-mir-122-5p | RNPC3 |
| hsa-mir-204-5p | RASA3 |
| hsa-mir-204-5p | RASA3 |
| hsa-mir-204-5p | NDUFA13 |
| hsa-mir-122-5p | CCDC84 |
| hsa-mir-122-5p | CCDC84 |
| hsa-mir-122-5p | TOR3A |
| hsa-mir-122-5p | PPP1CC |
| hsa-mir-122-5p | GLDN |
| hsa-mir-204-5p | RPS23 |
| hsa-mir-122-5p | PCLO |
| hsa-mir-204-5p | UBE2H |
| hsa-mir-122-5p | LYRM7 |
| hsa-mir-122-5p | TMEM216 |
| hsa-mir-122-5p | TEAD1 |
| hsa-mir-122-5p | FNBP1 |
| hsa-mir-204-5p | DCC |
| hsa-mir-122-5p | TAF9B |
| hsa-mir-204-5p | CHP1 |
| hsa-mir-122-5p | PTMA |
| hsa-mir-122-5p | FANCA |
| hsa-mir-122-5p | SECISBP2 |
| hsa-mir-122-5p | SEMA4D |
| hsa-mir-204-5p | LIN28B |
| hsa-mir-122-5p | FANCM |
| hsa-mir-122-5p | ZNF70 |
| hsa-mir-122-5p | CARD9 |
| hsa-mir-122-5p | DMBT1 |
| hsa-mir-122-5p | AGRN |
| hsa-mir-122-5p | ZC3H6 |
| hsa-mir-122-5p | DCUN1D3 |
| hsa-mir-122-5p | AGAP4 |
| hsa-mir-122-5p | ZP3 |
| hsa-mir-122-5p | C19orf54 |
| hsa-mir-122-5p | NBR1 |
| hsa-mir-122-5p | RALGAPA2 |
| hsa-mir-204-5p | SMIM15 |
| hsa-mir-122-5p | FAM221A |
| hsa-mir-122-5p | TMEM201 |
| hsa-mir-122-5p | ZDHHC11 |
| hsa-mir-122-5p | RPL14 |
| hsa-mir-204-5p | MSL1 |
| hsa-mir-122-5p | TRMT2B |
| hsa-mir-122-5p | ZNF292 |
| hsa-mir-122-5p | ZNF567 |
| hsa-mir-122-5p | ARID2 |
| hsa-mir-122-5p | TMEM63A |
| hsa-mir-204-5p | LCOR |
| hsa-mir-122-5p | ZNF107 |
| hsa-mir-122-5p | ZNF493 |
| hsa-mir-204-5p | PTPN1 |
| hsa-mir-122-5p | EPHB4 |
| hsa-mir-122-5p | ZNF124 |
| hsa-mir-122-5p | TSC22D2 |
| hsa-mir-122-5p | NCOR2 |
| hsa-mir-122-5p | LAMA2 |
| hsa-mir-122-5p | XRCC2 |
| hsa-mir-122-5p | SLC22A25 |
| hsa-mir-122-5p | RABL6 |
| hsa-mir-122-5p | TRAPPC4 |
| hsa-mir-122-5p | TRAPPC4 |
| hsa-mir-122-5p | AMZ2 |
| hsa-mir-122-5p | NF1 |
| hsa-mir-204-5p | VKORC1L1 |
| hsa-mir-122-5p | COL27A1 |
| hsa-mir-122-5p | ZNF700 |
| hsa-mir-122-5p | MVB12B |
| hsa-mir-122-5p | ANKRD36B |
| hsa-mir-122-5p | ARHGEF12 |
| hsa-mir-122-5p | SRGAP1 |
| hsa-mir-122-5p | FAM3C |
| hsa-mir-122-5p | CASP4 |
| hsa-mir-122-5p | ZNF100 |
| hsa-mir-122-5p | ZNF100 |
| hsa-mir-204-5p | ZNF100 |
| hsa-mir-204-5p | ZNF100 |
| hsa-mir-204-5p | IGF2R |
| hsa-mir-204-5p | DYNC1H1 |
| hsa-mir-122-5p | PCBP2 |
| hsa-mir-204-5p | ZGPAT |
| hsa-mir-122-5p | PGAP1 |
| hsa-mir-122-5p | NOL4L |
| hsa-mir-122-5p | SLC2A7 |
| hsa-mir-122-5p | ZNF165 |
| hsa-mir-122-5p | PELI1 |
| hsa-mir-122-5p | ZNF675 |
| hsa-mir-122-5p | DIO3 |
| hsa-mir-122-5p | IPP |
| hsa-mir-204-5p | MAP3K5 |
| hsa-mir-122-5p | SPN |
| hsa-mir-122-5p | MIB2 |
| hsa-mir-122-5p | RAB40C |
| hsa-mir-204-5p | CDC42SE1 |
| hsa-mir-122-5p | PARVA |
| hsa-mir-122-5p | CCDC180 |
| hsa-mir-122-5p | ZNF44 |
| hsa-mir-122-5p | NKIRAS1 |
| hsa-mir-122-5p | SPG7 |
| hsa-mir-122-5p | MPZL1 |
| hsa-mir-122-5p | SNHG12 |
| hsa-mir-122-5p | TUBA3C |
| hsa-mir-204-5p | RPS4X |
| hsa-mir-122-5p | ZNF273 |
| hsa-mir-122-5p | NUP62CL |
| hsa-mir-122-5p | SFI1 |
| hsa-mir-204-5p | ITSN2 |
| hsa-mir-122-5p | ZNF583 |
| hsa-mir-122-5p | ZNF28 |
| hsa-mir-122-5p | KCNRG |
| hsa-mir-122-5p | STK39 |
| hsa-mir-204-5p | ZNF652 |
| hsa-mir-122-5p | SLC5A3 |
| hsa-mir-204-5p | FAM169A |
| hsa-mir-122-5p | CNOT7 |
| hsa-mir-122-5p | MTOR |
| hsa-mir-122-5p | PNP |
| hsa-mir-122-5p | SFT2D1 |
| hsa-mir-122-5p | ZNF277 |
| hsa-mir-122-5p | LTN1 |
| hsa-mir-122-5p | CCDC152 |
| hsa-mir-122-5p | SMC5 |
| hsa-mir-122-5p | L1CAM |
| hsa-mir-122-5p | SREBF2 |
| hsa-mir-122-5p | RPL39 |
| hsa-mir-122-5p | ATG9A |
| hsa-mir-122-5p | NOS1AP |
| hsa-mir-122-5p | DMD |
| hsa-mir-204-5p | PJA2 |
| hsa-mir-122-5p | RORB |
| hsa-mir-122-5p | SNHG5 |
| hsa-mir-122-5p | GIGYF2 |
| hsa-mir-204-5p | GIGYF2 |
| hsa-mir-122-5p | RUFY2 |
| hsa-mir-122-5p | BRD2 |
| hsa-mir-122-5p | BRD2 |
| hsa-mir-122-5p | BRD2 |
| hsa-mir-122-5p | BRD2 |
| hsa-mir-122-5p | BRD2 |
| hsa-mir-122-5p | BRD2 |
| hsa-mir-122-5p | BRD2 |
| hsa-mir-204-5p | PBX2 |
| hsa-mir-204-5p | PBX2 |
| hsa-mir-204-5p | PBX2 |
| hsa-mir-204-5p | PBX2 |
| hsa-mir-204-5p | PBX2 |
| hsa-mir-204-5p | PBX2 |
| hsa-mir-204-5p | PBX2 |
| hsa-mir-122-5p | PBX2 |
| hsa-mir-122-5p | PBX2 |
| hsa-mir-122-5p | PBX2 |
| hsa-mir-122-5p | PBX2 |
| hsa-mir-122-5p | PBX2 |
| hsa-mir-122-5p | PBX2 |
| hsa-mir-122-5p | PBX2 |
| hsa-mir-122-5p | MRPL38 |
| hsa-mir-204-5p | SDHD |
| hsa-mir-122-5p | GPANK1 |
| hsa-mir-122-5p | GPANK1 |
| hsa-mir-122-5p | GPANK1 |
| hsa-mir-122-5p | GPANK1 |
| hsa-mir-122-5p | GPANK1 |
| hsa-mir-122-5p | GPANK1 |
| hsa-mir-122-5p | GPANK1 |
| hsa-mir-122-5p | ZNF805 |
| hsa-mir-204-5p | HLA-E |
| hsa-mir-204-5p | HLA-E |
| hsa-mir-204-5p | HLA-E |
| hsa-mir-204-5p | HLA-E |
| hsa-mir-204-5p | HLA-E |
| hsa-mir-204-5p | HLA-E |
| hsa-mir-204-5p | HLA-E |
| hsa-mir-204-5p | PPP1R11 |
| hsa-mir-204-5p | PPP1R11 |
| hsa-mir-204-5p | PPP1R11 |
| hsa-mir-204-5p | PPP1R11 |
| hsa-mir-204-5p | PPP1R11 |
| hsa-mir-204-5p | PPP1R11 |
| hsa-mir-204-5p | PPP1R11 |
| hsa-mir-204-5p | PPP1R11 |
| hsa-mir-204-5p | TRIM27 |
| hsa-mir-204-5p | TRIM27 |
| hsa-mir-204-5p | TRIM27 |
| hsa-mir-204-5p | TRIM27 |
| hsa-mir-204-5p | TRIM27 |
| hsa-mir-204-5p | TRIM27 |
| hsa-mir-204-5p | TRIM27 |
| hsa-mir-122-5p | TCTN1 |
| hsa-mir-204-5p | MZT1 |
| hsa-mir-204-5p | TRIM13 |
| hsa-mir-122-5p | PRSS1 |
| hsa-mir-122-5p | PRSS1 |
| hsa-mir-122-5p | SPIRE2 |
| hsa-mir-122-5p | VIT |
| hsa-mir-122-5p | E2F4 |
| hsa-mir-122-5p | PDE7A |
| hsa-mir-122-5p | IPO7 |
| hsa-mir-122-5p | TMEM256 |
| hsa-mir-122-5p | TMEM256 |
| hsa-mir-122-5p | ITPRIPL2 |
| hsa-mir-204-5p | ITPRIPL2 |
| hsa-mir-122-5p | CRYZL1 |
| hsa-mir-204-5p | C5orf51 |
| hsa-mir-204-5p | RNPS1 |
| hsa-mir-122-5p | RAB12 |
| hsa-mir-204-5p | RAB12 |
| hsa-mir-122-5p | ANKRD28 |
| hsa-mir-122-5p | ZNF580 |
| hsa-mir-204-5p | ZNF580 |
| hsa-mir-122-5p | ZNF611 |
| hsa-mir-122-5p | DENND1B |
| hsa-mir-122-5p | ZNF254 |
| hsa-mir-122-5p | GSTM2 |
| hsa-mir-122-5p | DNASE1 |
| hsa-mir-122-5p | IRF9 |
| hsa-mir-122-5p | IRF9 |
| hsa-mir-122-5p | TAX1BP3 |
| hsa-mir-122-5p | GANC |
| hsa-mir-122-5p | TTLL3 |
| hsa-mir-122-5p | C3orf56 |
| hsa-mir-204-5p | BBIP1 |
| hsa-mir-204-5p | HNRNPUL2 |
| hsa-mir-122-5p | MIR99AHG |
| hsa-mir-204-5p | FNIP1 |
| hsa-mir-122-5p | PPP3R1 |
| hsa-mir-122-5p | PPP2R2A |
| hsa-mir-122-5p | CCNL2 |
| hsa-mir-122-5p | EXOSC6 |
| hsa-mir-122-5p | SLC26A6 |
| hsa-mir-122-5p | RUSC1-AS1 |
| hsa-mir-122-5p | TEX41 |
| hsa-mir-122-5p | SAPCD1 |
| hsa-mir-122-5p | SAPCD1 |
| hsa-mir-122-5p | SAPCD1 |
| hsa-mir-122-5p | SAPCD1 |
| hsa-mir-122-5p | SAPCD1 |
| hsa-mir-122-5p | SAPCD1 |
| hsa-mir-122-5p | TMA7 |
| hsa-mir-122-5p | PPP1R3E |
| hsa-mir-122-5p | OR10G9 |
| hsa-mir-122-5p | LRRC37A2 |
| hsa-mir-122-5p | LRRC37A2 |
| hsa-mir-122-5p | LRRC37A2 |
| hsa-mir-204-5p | NME1 |
| hsa-mir-122-5p | CDRT4 |
| hsa-mir-122-5p | NSUN6 |
| hsa-mir-122-5p | CRCP |
| hsa-mir-204-5p | ARFGAP3 |
| hsa-mir-204-5p | PEG10 |
| hsa-mir-122-5p | MICAL3 |
| hsa-mir-122-5p | RBM12 |
| hsa-mir-122-5p | N4BP2L2 |
| hsa-mir-122-5p | NEAT1 |
| hsa-mir-204-5p | CEBPA |
| hsa-mir-122-5p | PDCD6 |
| hsa-mir-122-5p | CDK3 |
| hsa-mir-204-5p | TRNP1 |
| hsa-mir-204-5p | ATXN7L3B |
| hsa-mir-122-5p | PRKDC |
| hsa-mir-122-5p | PIGY |
| hsa-mir-122-5p | EID3 |
| hsa-mir-122-5p | POLR2M |
| hsa-mir-204-5p | MTRNR2L8 |
| hsa-mir-122-5p | ZNF10 |
| hsa-mir-122-5p | CUX1 |
| hsa-mir-122-5p | PRSS58 |
| hsa-mir-122-5p | PRSS58 |
| hsa-mir-122-5p | CEP95 |
| hsa-mir-122-5p | ANKRD20A1 |
| hsa-mir-122-5p | FLJ42627 |
| hsa-mir-122-5p | SRSF8 |
| hsa-mir-204-5p | DYNLL2 |
| hsa-mir-122-5p | OTUD7B |
| hsa-mir-122-5p | RNF115 |
| hsa-mir-122-5p | SEC22B |
| hsa-mir-122-5p | SRGAP2 |
| hsa-mir-122-5p | POLR2J2 |
| hsa-mir-122-5p | POLR2J2 |
| hsa-mir-122-5p | ZNF224 |
| hsa-mir-122-5p | NBPF12 |
| hsa-mir-122-5p | SLC6A14 |
| hsa-mir-122-5p | TMEM185A |
| hsa-mir-122-5p | TAF15 |
| hsa-mir-122-5p | TAF15 |
| hsa-mir-122-5p | NBPF10 |
| hsa-mir-204-5p | GTF2H5 |
| hsa-mir-122-5p | CD24 |
| hsa-mir-122-5p | DCP1A |
| hsa-mir-122-5p | CWC25 |
| hsa-mir-122-5p | CWC25 |
| hsa-mir-204-5p | LHX1 |
| hsa-mir-204-5p | LHX1 |
| hsa-mir-122-5p | ZNF280B |
| hsa-mir-122-5p | ZNF280B |
| hsa-mir-204-5p | ZNF280B |
| hsa-mir-204-5p | ZNF280B |
| hsa-mir-122-5p | MLLT6 |
| hsa-mir-122-5p | MLLT6 |
| hsa-mir-204-5p | PIP4K2B |
| hsa-mir-204-5p | PIP4K2B |
| hsa-mir-204-5p | MARCKS |
| hsa-mir-122-5p | TIMM22 |
| hsa-mir-122-5p | TIMM22 |
| hsa-mir-122-5p | TIMM22 |
| hsa-mir-122-5p | CISD3 |
| hsa-mir-122-5p | CISD3 |
| hsa-mir-122-5p | MYO19 |
| hsa-mir-122-5p | MYO19 |
| hsa-mir-122-5p | C17orf78 |
| hsa-mir-122-5p | C17orf78 |
| hsa-mir-122-5p | ACACA |
| hsa-mir-122-5p | ACACA |
